# Catalytic transfer zincation using ammonium cations

**DOI:** 10.1039/d5dt01816a

**Published:** 2025-08-14

**Authors:** Justyna Łosiewicz, Milan Kumar Bisai, Gary S. Nichol, Stuart A. Macgregor, Michael J. Ingleson

**Affiliations:** a EaStCHEM School of Chemistry, University of Edinburgh Edinburgh EH9 3FJ UK mingleso@ed.ac.uk; b EaStCHEM School of Chemistry, University of St Andrews St Andrews KY16 9ST UK sam38@st-andrews.ac.uk

## Abstract

C–H zincation is an efficient route to useful organozinc compounds. To date, most approaches effect aryl C–H zincation with selectivity controlled by substrate p*K*_a_. Herein, we report a heteroaryl C–H zincation method that uses an easy to access (β-diketiminate)Zn–Me complex and [(R_3_N)H][Anion] and proceeds under electronic control. This catalytic process is anion dependent; the less coordinating anion [B(C_6_F_5_)_4_]^−^ proved superior to [CHB_11_H_5_Br_6_]^−^, with the latter forming the intimate ion pair [(β-diketiminate)Zn(CHB_11_H_5_Br_6_)]. The Zn–Me complex and the [(R_3_N)H]^+^ salt can also initiate catalytic C–H borylation. A key requirement for a viable transfer zincation is a low barrier protonolysis reaction between the zinc–alkyl and [(R_3_N)H]^+^. This study found significant differences between the zinc–methyl and zinc–ethyl systems which stem from increased steric crowding in the zinc–ethyl congener. For the latter, the S_E_2(open) protonolysis transition state is forced to proceed through a sub-optimal non-linear Zn⋯C^α^⋯H_NR_3__ orientation which increases the energy of this barrier (*vs.* that for the Zn–Me analogue). While the scope of this initial process is limited, this work demonstrates that catalytic electrophilic transfer zincation is feasible and also highlights the sensitivity of a key transition state to small changes in the steric environment.

## Abstract 2

This work reports the first catalytic electrophilic transfer zincation in which the use of the right alkyl group is essential. This enables a zinc-catalysed glovebox-free borylation with (H–BBN)_2_.

## Graphical abstract

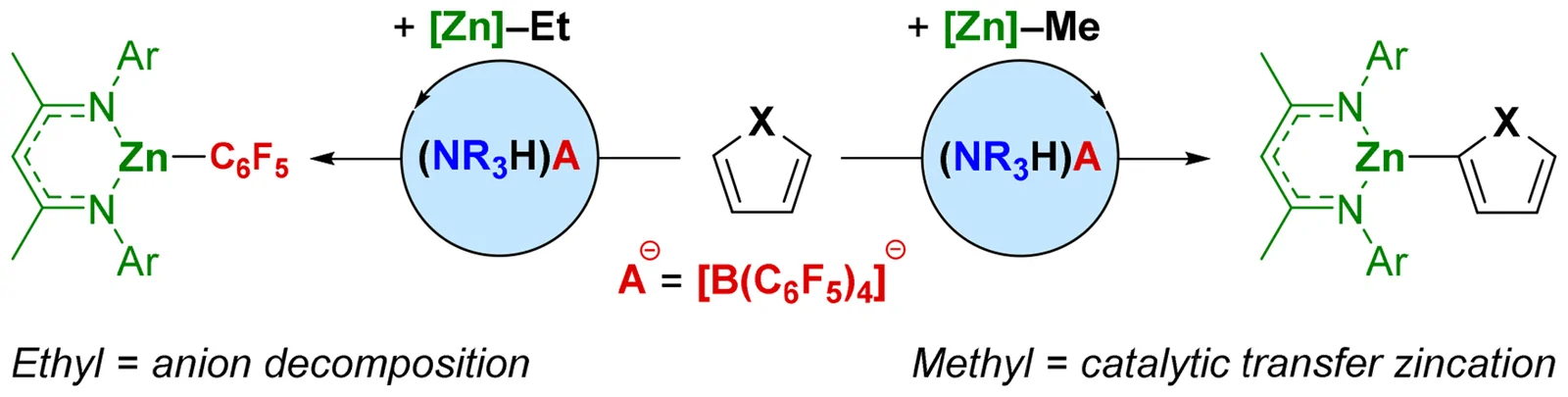

## Introduction

Organozinc reagents are ubiquitous in synthesis, in part due to the utility of the Negishi cross-coupling reaction.^1^ Classical routes to aryl-zinc complexes involve the metalation of aryl-halides, or the deprotonation of weakly acidic (hetero)arenes by strong organometallic Brønsted bases.^2,3^ The former requires aryl-X precursors, while the latter forms reactive intermediates (*e.g.*, aryl-lithiums)^4^ that are subsequently trapped by a zinc electrophile. More recently, it has been demonstrated that C–H bonds can be converted directly into C–Zn bonds.^5,6^ This is generally achieved using bimetallic reagents containing zincate units ([Y_3_Zn]^−^)^7^ that are produced by combining a zinc electrophile and an anionic Brønsted base. The first zincates utilised for C–H zincation were derived from Zn(alkyl)_2_ and M(NR_2_) (Fig. 1a),^8–10^ with the bimetallic nature of these essential for C–H zincation.^7,11^ Subsequent studies led to the development of multiple bimetallic systems that are effective for the C–H zincation of (hetero)arenes.^5,7^ This includes more tolerant systems that are not derived from dialkyl-zinc precursors, *e.g.*, (TMP)_*n*_Zn (TMP = 2,2,6,6-tetramethylpiperidide, *n* = 1 or 2) partnered with metal halides or metal alkoxides (Fig. 1a).^12–14^ While this approach is highly notable, it requires stoichiometric strong base (*e.g.*, Na-/Li-(TMP)) and metalation selectivity is controlled by C–H acidity (in the absence of other factors *e.g.*, directing groups).^15^ Alternative approaches that effect C–H zincation with selectivity dictated by (hetero)arene electronics and/or proceed catalytically are under-developed. Both are desirable to complement the well-established zincate-mediated approach to heteroarene C–H zincation.

**Fig. 1:**
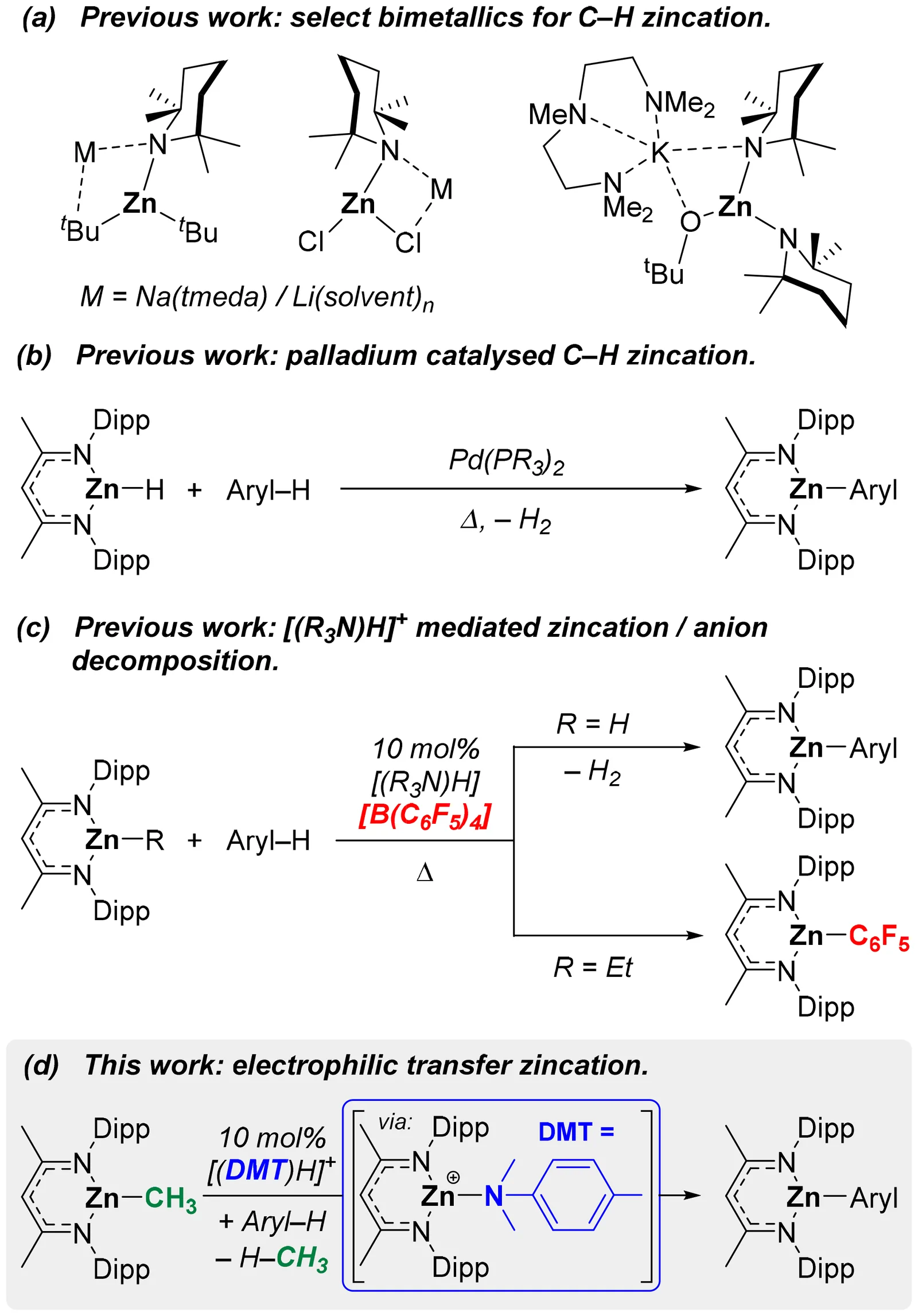
(a) Select zincates that effect C–H zincation; (b) catalytic C–H zincation using a Pd catalyst; (c) catalytic C–H zincation (top) or anion decomposition (bottom) dependent on identity of Zn–Y (Y = H or Et), using sub-stoichiometric R_3_N/[(R_3_N)H]^+^; (d) this work on using a Zn–Me complex for catalytic transfer zincation.

In contrast to stoichiometric methods, Crimmin and co-workers reported catalytic arene C–H zincation using a Pd(PR_3_)_2_ catalyst and (β-diketiminate)ZnH (Fig. 1b).^16^ While a key advance, this process led to organozinc products where selectivity again was controlled by substrate p*K*_a_, analogous to C–H zincation using zincates. More recently, we demonstrated that combining ^Dipp^NacNacZnH (^Dipp^NacNac = {(2,6-^i^Pr_2_C_6_H_3_)N(CH_3_)C}_2_CH) and sub-stoichiometric ammonium salts enabled the C–H zincation of heteroarenes (Fig. 1c, top).^17^ This produced organozinc products with selectivity controlled by heteroarene electronics, *e.g.*, indoles were zincated at C3. This was attributed to zincation being an S_E_Ar-type process using a cationic zinc electrophile. The ammonium cation by-product, [(R_3_N)H]^+^, from the S_E_Ar step enables turnover by reacting with ^Dipp^NacNacZnH. This evolves H_2_ and forms further equivalents of the key zinc electrophile, [^Dipp^NacNacZn(NR_3_)]^+^ (*e.g.*, inset Fig. 1d).

As part of a previous study, electrophilic transfer zincation was attempted.^17^ This is the transformation of a zinc alkyl into a zinc-heteroarene *via* an S_E_Ar type mechanism. However, using ^Dipp^NacNacZnEt and [(R_3_N)H][Anion] transfer zincation was not successful. Instead, anion decomposition occurred (Fig. 1c, bottom). Our studies indicated a higher barrier to the protonolysis of ^Dipp^NacNacZnEt with [(R_3_N)H]^+^ relative to ^Dipp^NacNacZnH, consistent with the absence of C–H zincation using the former. In contrast, catalytic electrophilic transfer metalation was achieved using ^Dipp^NacNacAlMe_2_, with this complex having a lower barrier to metal-alkyl protonolysis using [(R_3_N)H]^+^ relative to the Zn–Et congener.^17^ Given that NacNacZn–alkyl complexes are significantly simpler to synthesise than NacNacZnH,^18,19^ the development of an electrophilic transfer zincation process remained of interest. A key challenge to achieving this goal is identifying a system with a low barrier to Zn–alkyl protonolysis using [(R_3_N)H]^+^ salts.

Herein, we present a catalytic in R_3_N/[(R_3_N)H]^+^ electrophilic transfer zincation to form aryl-zinc complexes using the ^Dipp^NacNacZnMe analogue of the Zn–ethyl complex that previously proved unsuccessful in transfer zincation. We have also identified the origin of the disparity between the Zn–Et/-Me systems and utilised this system to catalyse the borylation of heteroarenes with 9-borabicyclo-[3.3.1]-nonane, (H–BBN)_2_.

## Results and discussion

### Initial computational study

This study commenced by investigating computationally if ^Dipp^NacNacZnMe, 1-Me, undergoes protonolysis with an ammonium cation with a lower barrier compared to the Zn–Et derivative (Fig. 2). All calculations were performed at the B3PW91(def2-TZVP, D3(BJ), PhCl)//B3PW91(Zn: SDD; S: SDD(d); other atoms: 6-31G**) level of theory which was chosen based on its performance in the ^Dipp^NacNacZnH mediated C–H zincation.^17^ [^Dipp^NacNacZn–DMT]^+^ ([1-DMT]^+^) (DMT = *N*,*N*-dimethyl-4-toluidine) is taken as the starting point of the cycle, with this complex chosen as it was effective in both C–H zincation and C–H borylation, the latter with a range of hydroboranes.^20,21^ As the C–H zincation using [1-DMT]^+^ to form ^Dipp^NacNacZn-thienyl, 3a, and [(DMT)H]^+^ is significantly endergonic (+14.8 kcal mol^−1^) the protonolysis step between 1-Me and [DMT(H)]^+^ requires a low barrier for this catalytic transfer zincation to be viable. Initially, we explored a ligand-assisted protonolysis pathway as this had the lowest barrier using the Zn–H congener (Fig. 2 left, pathway A). However, while both TS1^P-ZnMe^ and TS2^P-ZnMe^ are kinetically accessible (at +20.3 and +21.9 kcal mol^−1^, respectively) the subsequent transition state for methane loss *via*INT2^P-ZnMe^ was too high in energy (+33.6 kcal mol^−1^) which excluded pathway A. This high overall barrier is consistent with our previous study, and is attributed to a reduced orbital overlap in TS3^P-ZnMe^ between a directional sp^3^ orbital of the methyl group in the Zn–Me unit and the H 1s orbital in the NacNac C_γ_–H moiety. An alternative more feasible pathway subsequently was identified (Fig. 2 right, pathway B). This involved direct proton transfer from [DMT(H)]^+^ to the Zn–Me unit in ^Dipp^NacNacZnMe *via* transition state TS4^P-ZnMe^ (at +24.8 kcal mol^−1^). This resulted in the formation of a methane σ-complex INT3^P-ZnMe^ (at +14.0 kcal mol^−1^). The subsequent step, methane dissociation *via*TS5^P-ZnMe^ (at +21.0 kcal mol^−1^), forms INT4^P-ZnMe^, where [NacNacZn]^+^ is stabilised by interacting with two isopropyl groups from the dipp substituents. DMT binding forms another equivalent of [1-DMT]^+^ and completes the cycle. Overall, the transfer zincation cycle has a Δ*G* = −12.3 kcal mol^−1^, with the protonolysis phase being highly exergonic. Notably, protonolysis pathway B has an overall free energy span comparable to the protonolysis of ^Dipp^NacNacZnH by [(DMT)H]^+^. As ^Dipp^NacNacZnH/[(DMT)H]^+^ effects heteroarene C–H zincation at 60 °C, these calculations predict that catalytic transfer zincation using 1-Me/[(DMT)H]^+^ should be feasible.

**Fig. 2:**
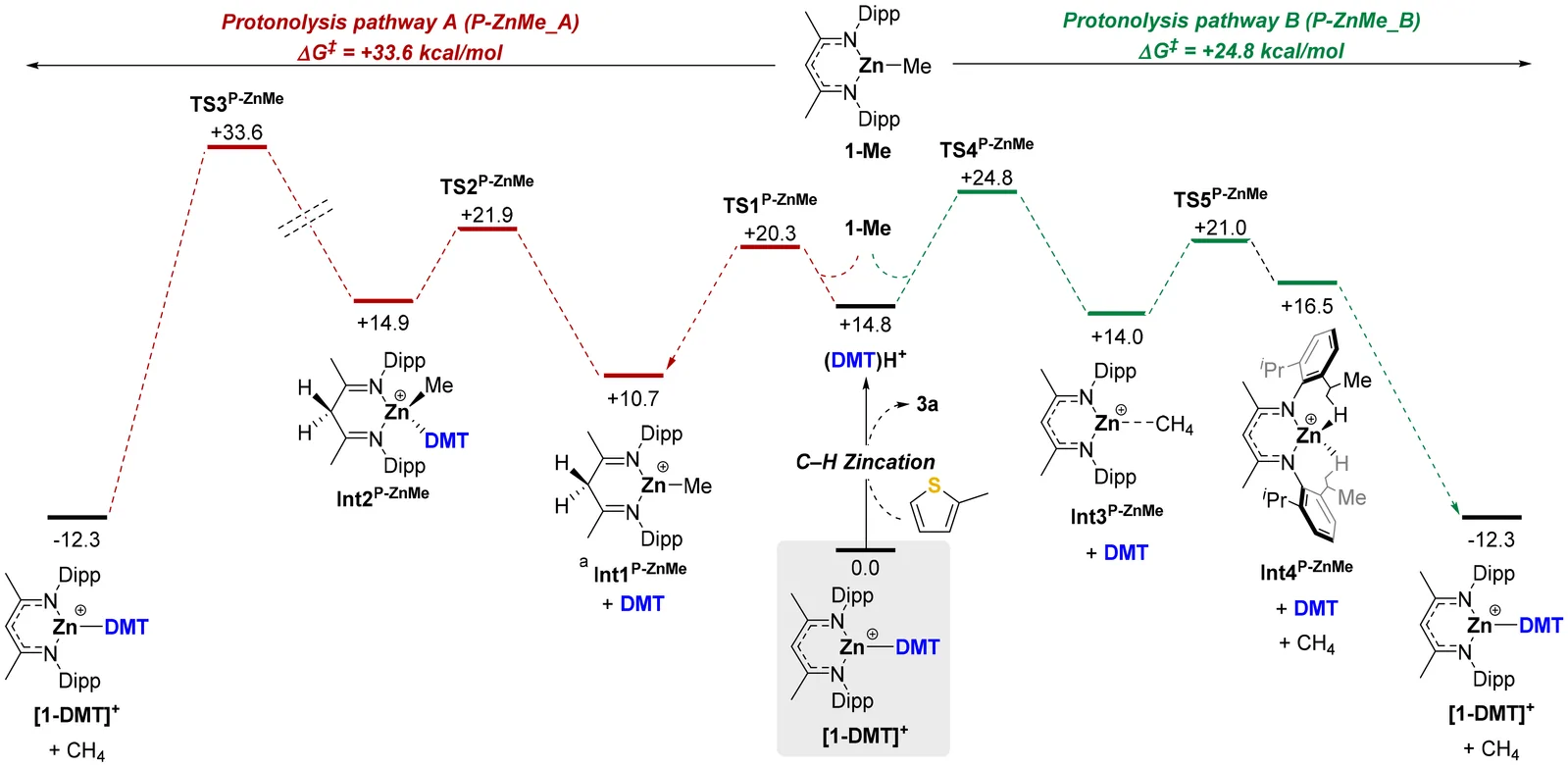
Computed free energy reaction profile (kcal mol^−1^) at 25 °C for the C–H zincation of 2-methyl-thiophene focused on the two explored protonolysis pathways: a ligand assisted pathway (A, left) and a direct Zn–C protonolysis proceeding *via* a methane σ-complex (pathway B, right). ^*a*^ TS1^P-ZnMe^ leads initially to a H-bonded DMT adduct at +13.9 kcal mol^−1^ that is omitted for brevity (see SI for details).

### C–H zincation studies

C–H zincation initially was investigated using one equivalent of 1-Me, two equivalents of 2-methyl-thiophene and 10 mol% of [(DMT)H][B(C_6_F_5_)_4_]. Note, 1-Me can be prepared from ^Dipp^NacNacH and ZnMe_2_ in a single step with excellent yield (>95%). At 100 °C (Table 1, entry 1) C–H zincation proceeded with a conversion of 63% after 24 h. If no Brønsted acid was present C–H zincation did not occur (entry 2). The outcome using [DMT(H)][B(C_6_F_5_)_4_] was improved at 110 °C (entry 3), however, increasing the temperature to 140 °C resulted in minimal (<10%) C–H zincation due to anion decomposition forming ^Dipp^NacNacZn–C_6_F_5_ (by ^19^F NMR spectroscopy).

**Table 1:** Select optimisation reactions of C–H zincation of 2-methyl-thiophene^a^

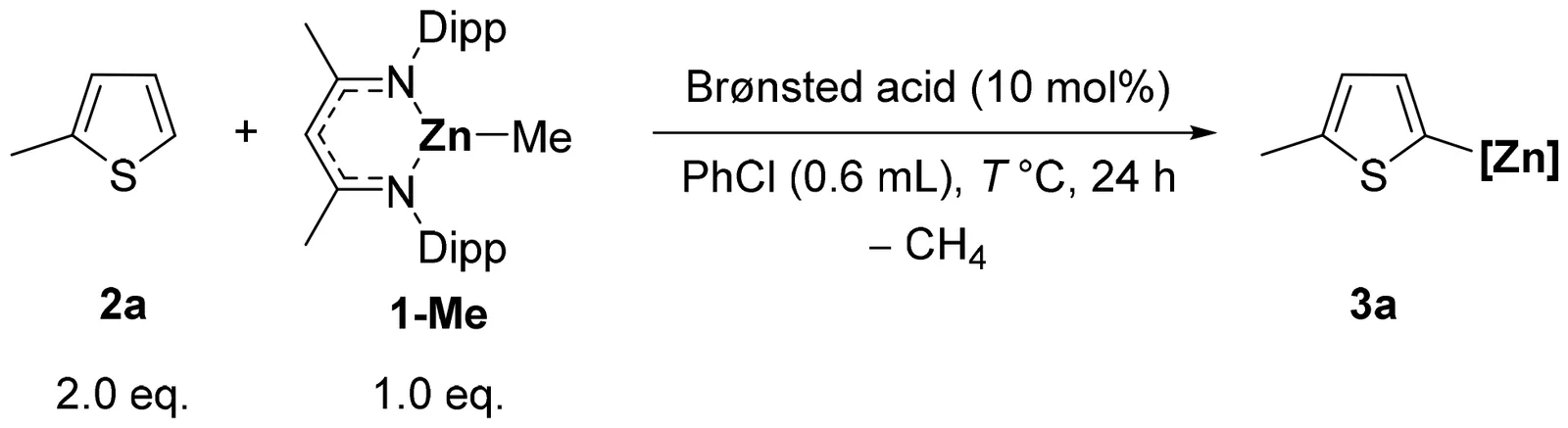			
Entry	Brønsted acid	*T* (°C)	Con.^b^ (%)
1	[(DMT)H][B(C_6_F_5_)_4_]	100	63
2	—	110	0
3	[(DMT)H][B(C_6_F_5_)_4_]	110	80
4	[(DMT)H][B{C_6_H_3_(CF_3_)_2_}_4_]	100	39
5	[(Et_3_N)H][B{C_6_H_3_(CF_3_)_2_}_4_]	100	46
6	[(2,4-Br_2_-C_6_H_3_NMe_2_)H][B(C_6_F_5_)_4_]	100	43
7	[(2,4,6-(CH_3_)_3_C_6_H_2_NMe_2_)H][B(C_6_F_5_)_4_]	110	62
8	[(DMT)H][CHB_11_H_5_Br_6_]	110	28
9	[(DMT)H][CHB_11_H_5_Br_6_]	140	56
**10** c	**[(DMT)H][B(C**_**6**_ **F**_**5**_ **)**_**4**_ **]**	**110**	**87**

a 2-Methyl-thiophene (2.0 eq.), 1-Me (1.0 eq.), and Brønsted acid (0.1 eq.) in PhCl (0.6 mL), [0.16 M].

b Conversion by ^1^H NMR spectroscopy based on the NacNac C_γ_–H in the product *versus* in the starting material.

c Reaction run at double concentration [0.32 M].

Changing the anion to [B{C_6_H_3_(CF_3_)_2_}_4_]^−^ (entry 4) resulted in a poorer outcome due to anion decomposition occurring at 100 °C (by ^19^F NMR spectroscopy). While a triethylammonium salt containing the same anion did not show any signs of anion decomposition at 100 °C, it gave a lower conversion than using [(DMT)H][B(C_6_F_5_)_4_] (entry 1 *vs.* 5). Using a more acidic ammonium cation, [(2,4-Br_2_-C_6_H_3_NMe_2_)H]^+^, did not improve the conversion (entry 6). Similarly, the use of a bulkier salt, [(2,4,6-(CH_3_)_3_-C_6_H_2_NMe_2_)H][B(C_6_F_5_)_4_], also led to a poorer outcome (entry 7) and some anion decomposition was observed. Given the repeated observation of anion decomposition at the temperatures necessary for transfer zincation the use of a more robust weakly coordinating anion was explored. The hexa-brominated carborane anion was chosen based on its high stability to strong Lewis and Brønsted acids, including those present during catalytic S_E_Ar cycles.^22–24^ The [DMT(H)]^+^ salt of [CHB_11_H_5_Br_6_]^−^ was synthesised and used with 1-Me/2-methyl-thiophene at 110 °C and 140 °C (entries 8 and 9). While no anion decomposition was observed (by ^11^B NMR spectroscopy) only moderate conversion was achieved even after 24 h at 140 °C. Ultimately, the optimal conditions identified were using [(DMT)H][B(C_6_F_5_)_4_] at 110 °C and increasing the concentration (entry 10), this afforded a conversion of 87% (for full optimisation see Table S1).

The origin of the poorer outcomes using [CHB_11_H_5_Br_6_]^−^ was investigated by assessing the coordinating ability of this anion towards the [^Dipp^NacNacZn]^+^ cation. 1-Me was reacted with [Ph_3_C][CHB_11_H_5_Br_6_] in a 1 : 1 ratio. This led to the formation of a single new compound (by ^1^H NMR spectroscopy) in 95% yield (*vs.* an internal standard) and Ph_3_CMe as the expected by-product. The same compound also was formed from the reaction of ^Dipp^NacNacZnH and [Ph_3_C][CHB_11_H_5_Br_6_]. Crystals suitable for single crystal X-ray diffraction were grown from PhCl/pentane, with this revealing the compound to be [1-CHB_11_H_5_Br_6_], an intimate ion pair (Fig. 3). In this structure the [CHB_11_H_5_Br_6_]^−^ anion interacts in a bidentate manner with the zinc centre. This is in contrast to the [B(C_6_F_5_)_4_]^−^ salts of related [NacNacZn]^+^ cations which form solvent-separated systems in which zinc is bound to an arene or a haloarene.^25^ A compound with a similar binding motif, A, was reported by Dorta and co-workers (Fig. 3a, inset).^26^[1-CHB_11_H_5_Br_6_] exhibits longer Zn–Br distances than compound A (2.519(8)/2.571(1) Å *vs.* 2.404(9)/2.435(1) Å), which suggests a weaker interaction between [^Dipp^NacNacZn]^+^ and the [CHB_11_H_5_Br_6_]^−^ anion. When one equivalent of DMT was added to [1-CHB_11_H_5_Br_6_] an equilibrium between [^Dipp^NacNacZn–DMT][CHB_11_H_5_Br_6_] ([1-DMT][CHB_11_H_5_Br_6_]) and [1-CHB_11_H_5_Br_6_] was observed by NMR spectroscopy (Fig. 3a and Fig. S56). Only after excess DMT was added (5 eq.) was all [1-CHB_11_H_5_Br_6_] consumed to form [1-DMT][CHB_11_H_5_Br_6_] (by NMR spectroscopy). The NMR data for [1-DMT][CHB_11_H_5_Br_6_] were closely comparable to those previously reported for the [1-DMT][B(C_6_F_5_)_4_] salt, indicating formation of a solvent-separated ion pair. Combined, these experiments revealed that the [CHB_11_H_5_Br_6_]^−^ anion and DMT have comparable binding affinities towards [NacNacZn]^+^, in contrast to [B(C_6_F_5_)_4_]^−^ which is less coordinating. Given the first step of the C–H zincation is the displacement of DMT (or anion) from zinc by the incoming heteroarene, the presence of a more coordinating anion will retard this process. This is consistent with the catalytic results (entry 3 *vs.* 8) and is analogous to the previous work with the [OTf]^−^ anion.^17^ Therefore, despite the greater thermal stability of [CHB_11_H_5_Br_6_]^−^ under these conditions all further studies utilised the [B(C_6_F_5_)_4_]^−^ anion.

**Fig. 3:**
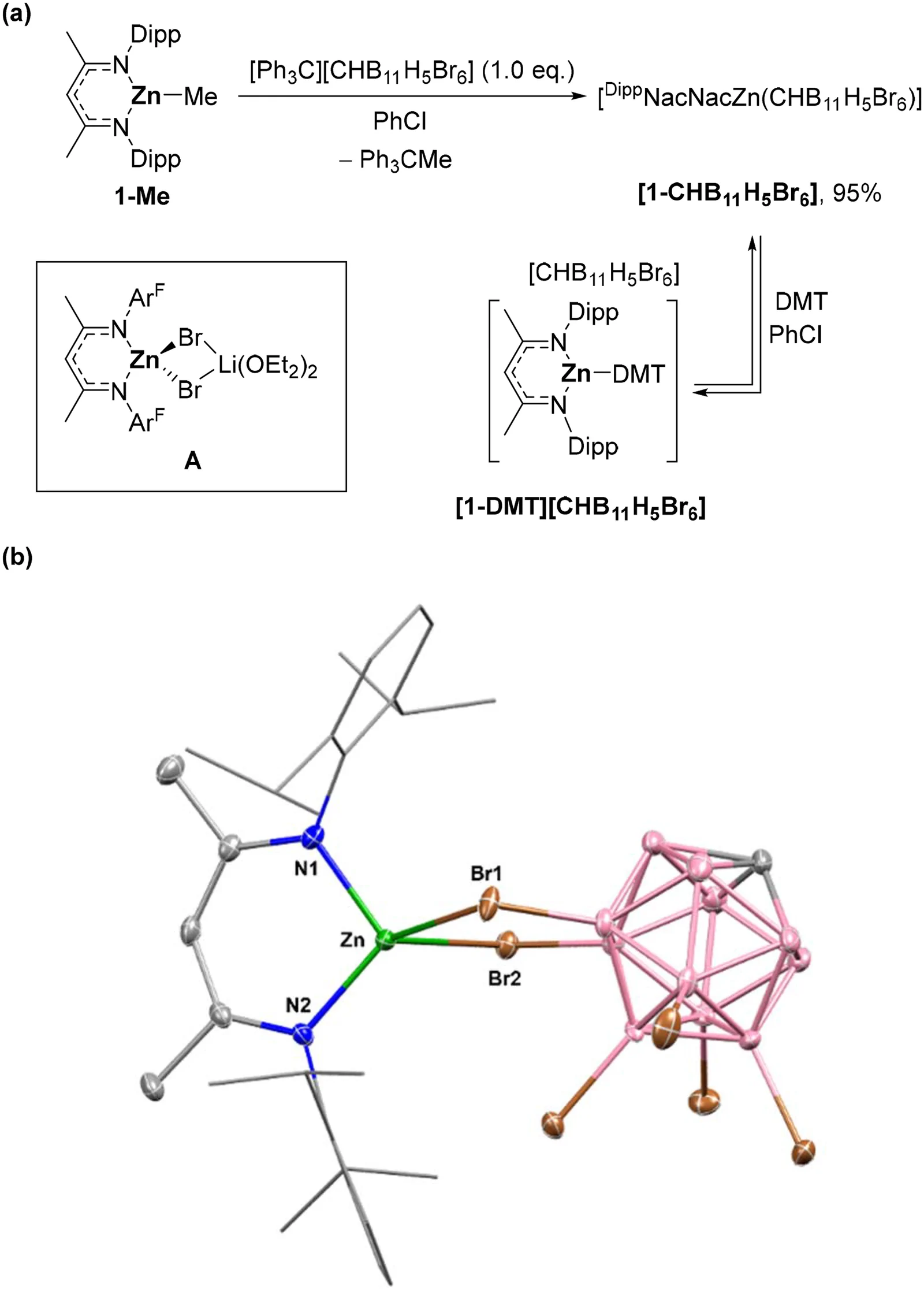
(a) The formation of [1-CHB_11_H_5_Br_6_] and its subsequent reaction with DMT; inset: structure of ArNacNacZn(μ-Br)_2_Li(Et_2_O)_2_ (Ar = 2,6-F_2_C_6_H_3_). (b) Solid-state structure of [1-CHB_11_H_5_Br_6_]; ellipsoids are at 50% probability and H atoms are omitted for clarity. Selected bond distances (Å): Zn–N1 = 1.933(3), Zn–N2 = 1.925(5), Zn–Br1 = 2.519(8), Zn–Br2 = 2.571(1).

### Substrate scope

The scope of electrophilic transfer zincation was assessed next (Chart 1). This revealed that the C–H zincation using ^Dipp^NacNacZnMe was limited in comparison to C–H zincation using ^Dipp^NacNacZnH. This is consistent with the zincation of 2-methyl-thiophene using 1-Me requiring harsher conditions relative to that using the Zn–H congener (24 h at 110 °C *vs.* 15 h at 60 °C). This indicates that the overall barrier for catalytic transfer zincation using 1-Me is higher relative to the overall barrier to catalytic C–H zincation using the Zn–H derivative. Nevertheless, using 1-Me several thiophenes (3a–3c) underwent zincation at the α-position with good yields (87–99%), while benzofuran, 3d, was also amenable to transfer zincation with a high yield (90%). Zincation of the less nucleophilic heteroarene, 2-bromo-thiophene, as well as zincation of 2-ethyl-furan and *N*-methyl-indole also proceeded but with poor yields (*ca.* 20%).

**Chart 1:**
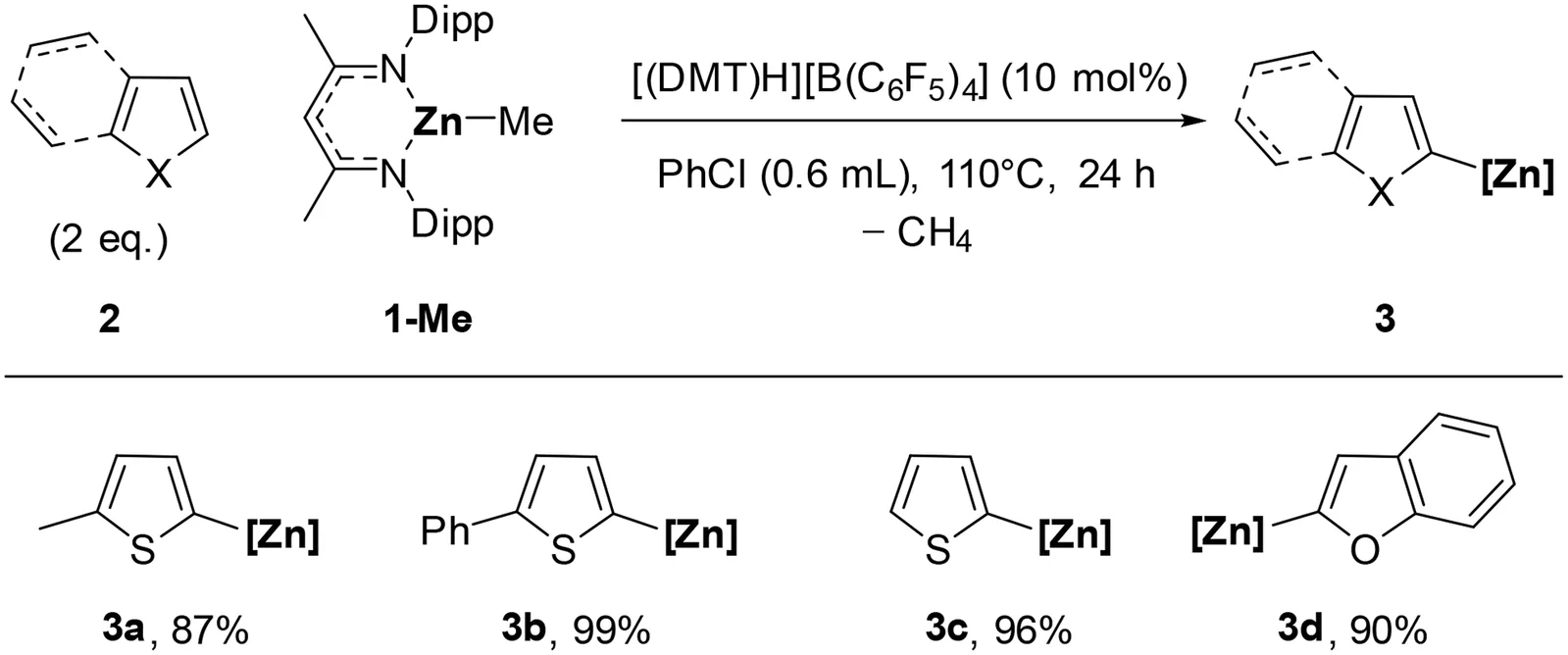
Substrate scope for catalytic transfer zincation.

### Mechanistic studies

Given that the catalytic C–H zincation using 1-Me required heating to high temperatures (≥100 °C) the free energy profile for the transfer zincation of 2a using 1-Me was recalculated at the optimised reaction temperature of 110 °C (Fig. 4). Only minor changes with respect to the profile calculated at 25 °C were observed, *i.e.*, the C–H zincation phase was slightly less endergonic at 110 °C, while there was only a modest increase in the energy barriers at the higher temperature. In the 110 °C energy profile transition states TS2^CHZn^ and TS4^P-ZnMe^ have the highest energies (+25.1 kcal mol^−1^ and +26.6 kcal mol^−1^, respectively). These are sufficiently close in energy that either could be the actual rate-determining step (RDS). To gain further insight into the RDS, the kinetic isotope effect (KIE) from deuteration at the C5-position of 2-methyl-thiophene was measured in independent reactions under the optimised catalytic conditions. A *k*_H_/*k*_D_ of 0.92 for the C–H zincation of 2-methyl-thiophene using 1-Me was determined (see Fig. S57). This value can be explained by:

(i) TS2^CHZn^ being the RDS, with this transition state involving a change in hybridisation at the deuterated carbon centre reflected in the increase of Zn–C–H angle (∠ = 75.2° in INT1^CHZn^ and ∠ = 95.0° in INT2^CHZn^). As the reaction approaches TS2^CHZn^, the hybridisation changes from sp^2^ towards sp^3^ thus the out-of-plane C–H bend stiffens resulting in an inverse KIE;^27^

(ii) Or, the measured KIE is from a combination of one or more inverse equilibrium isotope effects (EIE) arising from a change in hybridisation and then a normal KIE for TS4^P-ZnMe^, if this is the RDS.^27^

**Fig. 4:**
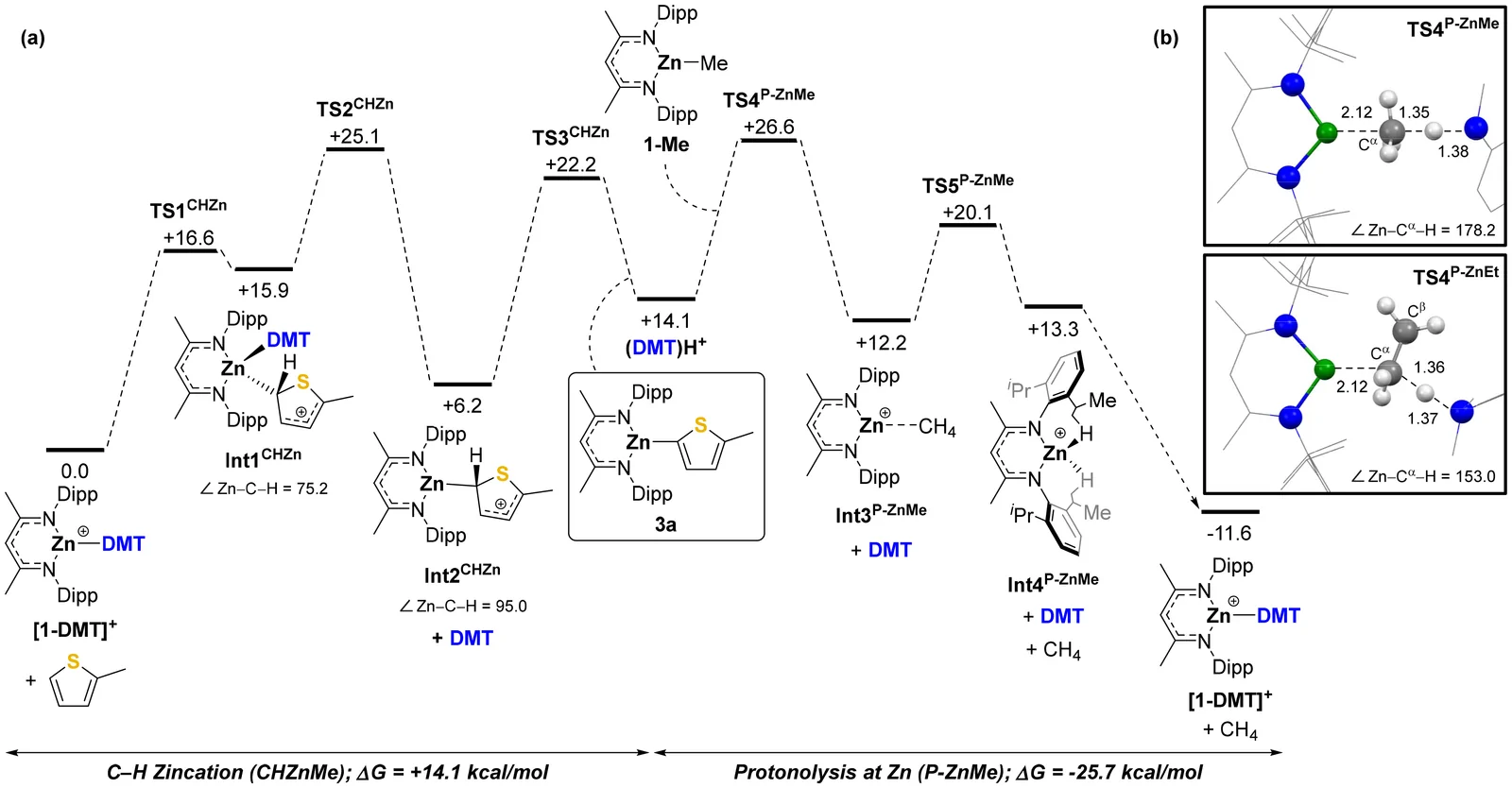
(a) Computed free energy reaction profile (kcal mol^−1^) at 110 °C for the catalytic C–H zincation of 2-methyl-thiophene; (b) images of selected stationary points (H atoms of NacNac ligand and DMT apart from the N–H bond omitted for clarity).

To investigate this further, the energy profile was calculated with 5-*d*-2-methyl-thiophene (see Fig. S62) and theoretical KIEs were determined. Regarding TS4^P-ZnMe^ the KIE for this isolated step at 110 °C was 2.51. In contrast, the calculated KIE for the step proceeding through TS2^CHZn^ was 0.88. The latter is in very close agreement with the experimentally observed KIE. However, considering option (ii), the overall KIE of the process could involve one or more inverse EIEs combined with the KIE for TS4^P-ZnMe^_._ While the EIEs for the formation of INT1^CHZn^ and INT2^CHZn^ were 1.14 and 1.00, respectively, the EIE for formation of 3a and [DMT(H)]^+^ was 0.59. However, combining EIEs with the KIE for TS4^P-ZnMe^ gives a total KIE at 110 °C of 1.69 (see SI for details) which does not match the measured KIE of 0.92.

Overall, the KIE experiments are more consistent with TS2^CHZn^ being the RDS. This indicates a small discrepancy between the calculated energies and the real energies associated with TS2^CHZn^ and/or TS4^P-ZnMe^. The energy of TS2^CHZn^ was found to be more temperature dependent (21.7 kcal mol^−1^ at 25 °C *vs.* 25.1 kcal mol^−1^ at 110 °C) than TS4^P-ZnMe^ (24.8 kcal mol^−1^ at 25 °C *vs.* 26.6 kcal mol^−1^ at 110 °C). Uncertainty in the estimated entropic contributions could therefore contribute to this small discrepancy.

We were also interested in understanding the origin of the reactivity difference between ^Dipp^NacNacZnMe (1-Me) and ^Dipp^NacNacZnEt (1-Et), with the latter not effective for electrophilic transfer zincation. Therefore, the mechanism for C–H zincation of 2-methyl-thiophene was computed for 1-Et at 110 °C (see Fig. S66). This revealed a higher energy for TS4^P−ZnEt^ (+29.3 kcal mol^−1^) compared to TS4^P-ZnMe^ (+26.6 kcal mol^−1^). TS4^P−ZnR^ involves protonation of the Zn–R unit in both cases. While the distances in the Zn⋯C^α^⋯H_NR_3__ unit in TS4^P−ZnR^ for the Zn–Et and Zn–Me congeners are effectively identical the angles are not (inset Fig. 4b). The proton transfer from [DMT(H)]^+^ to 1-Me occurs *via* an effectively linear Zn–C^α^–H_NR_3__ arrangement in TS4^P-ZnMe^ (∠ = 178.2°) resulting in an approximately trigonal bipyramidal geometry at C^α^. This is close to ideal for an electrophilic aliphatic substitution *via* backside attack (an invertive S_E_2(open) mechanism). In contrast, in TS4^P−ZnEt^ the Zn⋯C^α^⋯H_NR_3__ unit is significantly distorted away from a linear arrangement. The presence of the methyl substituent in the Zn–Et congener forces a deviation away from linearity in this transition state to minimise unfavourable interactions with the DMT moiety. This results in a decrease in the Zn–C^α^–H_NR_3__ angle to 153.0°. Such compression reduces the orbital overlap in the S_E_2(open) transition state which is consistent with the higher protonolysis barrier calculated. This difference in methyl *vs.* ethyl is presumably due to the sterically crowded pocket around the Zn–alkyl moiety generated by the two dipp groups restricting the accessible orientations of the ethyl group and the incoming [(DMT)H]^+^. This steric effect is indicated by the calculated Zn–C^α^–C^β^ angle in 1-Et being 118.0°, with similar Zn–C^α^–C^β^ angles observed in the solid-state structures of related ^Dipp^NacNacZn–alkyl complexes.^28,29^ Therefore, for the Zn–Et congener the incoming [(DMT)H]^+^ is forced into an unfavoured approach vector leading to TS4^P−ZnEt^. Furthermore, the greater energy difference between TS2^CHZn^ and TS4^P−ZnEt^ (*δ*Δ*G* = 4.2 kcal mol^−1^) relative to that for the Zn–Me congener (*δ*Δ*G* = 1.5 kcal mol^−1^), suggests the RDS for the C–H zincation using ^Dipp^NacNacZnEt will be the protonolysis step. This is consistent with the higher barrier for Zn–Et protonolysis resulting in anion decomposition proceeding instead of C–H zincation. In this case, the calculations appear to agree well with the experimental observations.

### C–H borylation studies

Previous work demonstrated that C–H zincation can be sequenced with σ-bond metathesis using the hydroborane 9-borabicyclo-[3.3.1]-nonane, (H–BBN)_2_,^21^ to enable zinc catalysed C–H borylation. The original procedure used sub-stoichiometric ^Dipp^NacNacZnH, 1-H, and a [(R_3_N)H]^+^ salt. This methodology was therefore extended to the more readily synthesised derivative ^Dipp^NacNacZnMe. 2-Methyl-thiophene initially was tested using the optimised conditions from our previous borylation study, with 1-Me used in place of 1-H (Chart 2). After 24 h at 80 °C, C–H borylation proceeded forming 4a in excellent yield (98%). Notably, substrates that performed poorly in electrophilic transfer zincation using 1-Me proved more amenable to C–H borylation. For example, 4e was formed in good yield (81%), despite 2-bromo-thiophene giving a poor outcome in electrophilic transfer zincation. Other heteroarenes, such as furan and 2-ethyl-furan, also were amenable to C–H borylation to form 4f and 4g, respectively. Lastly, *N*-methyl-indole underwent borylation to form 4h in moderate yield at C3, with this selectivity confirming C–H functionalisation again is occurring *via* an S_E_Ar mechanism. The superior outcomes for borylation relative to transfer zincation using 1-Me are attributed to the fact that ^Dipp^NacNacZnH (1-H) is formed during catalytic borylation (*e.g.*, from σ-bond metathesis of 3a with the hydroborane) and 1-H has a lower overall barrier to C–H zincation. Nevertheless, to showcase the improved operational simplicity of using 1-Me (*vs.*1-H) as an initiator in catalytic borylation, 2-methyl-thiophene was borylated in excellent yield (95%) in a glovebox-free procedure that makes and utilises 1-Me*in situ* (Chart 2).

**Chart 2:**
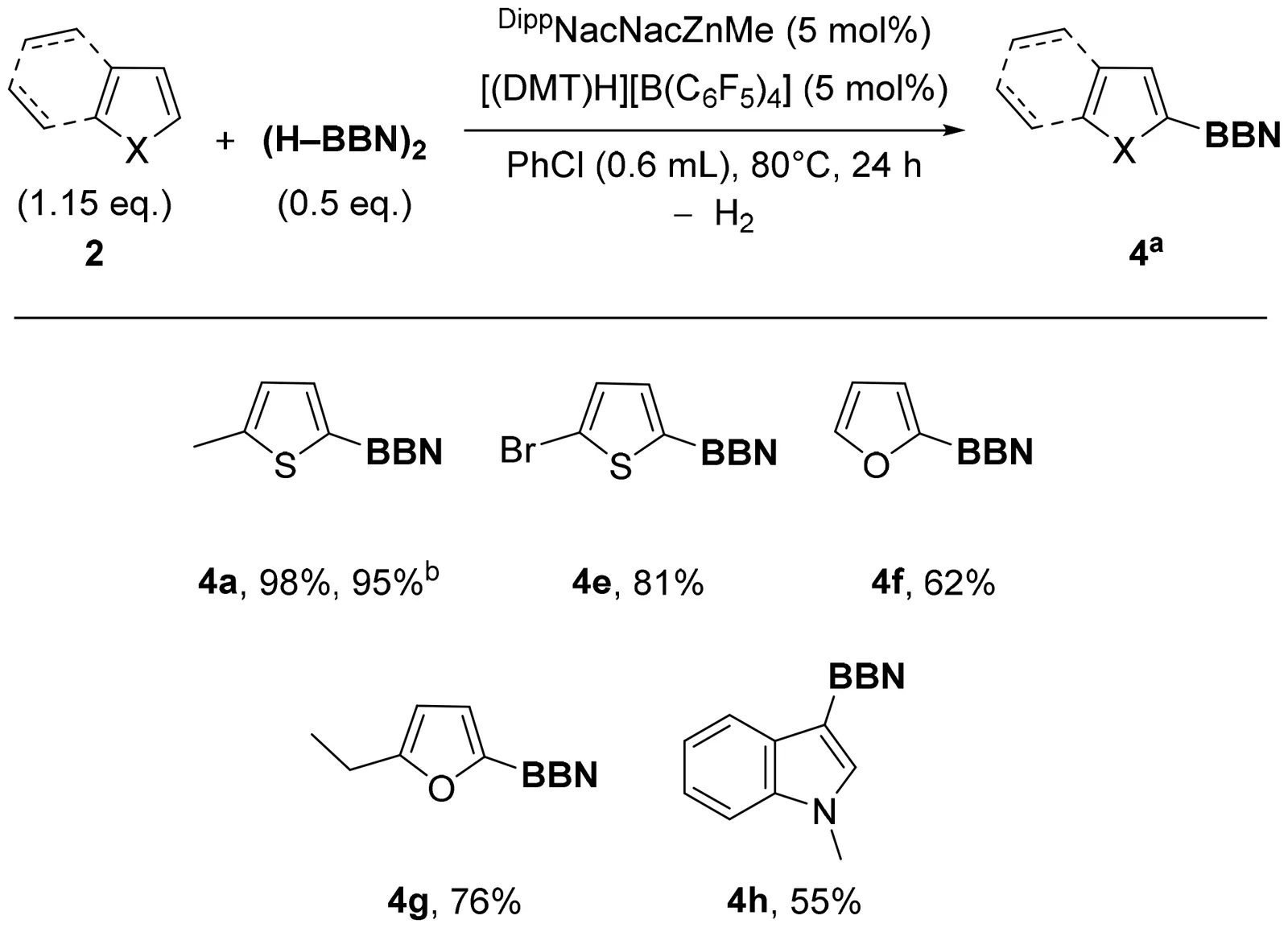
Substrate scope for zinc catalysed borylation. ^*a*^Yield *versus* an internal standard. ^*b*^Glove-box free procedure (see SI for details).

Finally, we probed how the active catalytic species, [^Dipp^NacNacZn–DMT]^+^, was accessed from 1-Me under the catalytic borylation conditions. Two possibilities were considered: (i) protonolysis of 1-Me by [(DMT)H]^+^; (ii) 1-Me undergoing σ-bond metathesis with the hydroborane, and then the zinc hydride product, 1-H, undergoing protonolysis with [(DMT)H]^+^. Stoichiometric experiments revealed that with 1-Me both protonolysis and σ-bond metathesis proceed at room temperature (Fig. 5, see SI for details). However, under the catalytic borylation conditions, (H–BBN)_2_ is used in stoichiometric quantity while ^Dipp^NacNacZnMe and [(DMT)H][B(C_6_F_5_)_4_] are present in sub-stoichiometric quantities (5 mol%). During catalysis 3.3% of MeBBN is formed (yield *vs.* internal standard) which implies initiation proceeding *via* Zn–Me/B–H σ-bond metathesis dominates. The remaining 1.7% of 1-Me presumably reacts *via* protonolysis with [(DMT)H]^+^ to form methane and [^Dipp^NacNacZn–DMT]^+^. Following the first C–H borylation cycle, ^Dipp^NacNacZnH is formed which reacts on further by facile protonolysis providing a lower energy pathway for further C–H zincation/borylation cycles, relative to ^Dipp^NacNacZnMe.

**Fig. 5:**
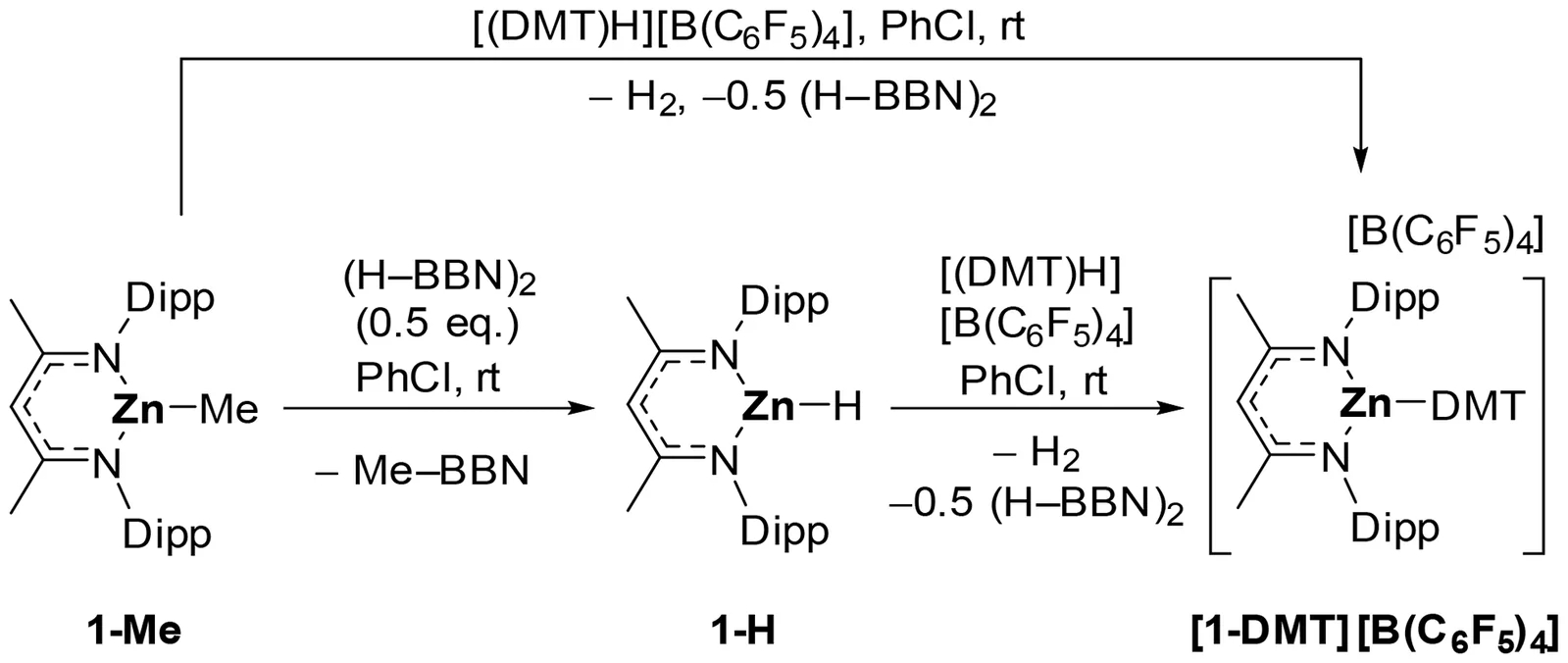
Formation of [1-DMT]^+^*via* two routes starting from 1-Me: direct protonolysis with [(DMT)H][B(C_6_F_5_)_4_] (top) or initial reaction with (H–BBN)_2_ (0.5 eq.) forming 1-H followed by subsequent reaction with the ammonium salt.

## Conclusions

To conclude, a minor change in structure from a NacNacZn–ethyl to a NacNacZn–methyl complex is sufficient to switch on catalytic electrophilic transfer zincation using sub-stoichiometric [(R_3_N)H]^+^ salts. While the scope of this process is limited it is, to our knowledge, the first reported electrophilic transfer zincation. This process can be sequenced with a σ-bond metathesis step using a hydroborane to enable a zinc-catalysed C–H borylation. Here, the simpler synthetic accessibility of NacNacZn–Me derivatives (relative to zinc-hydride analogues) allows for a glovebox-free protocol to be developed where the zinc–methyl complex is made and used *in situ*. Mechanistic and computational studies were performed which revealed that protonolysis transition state energies are highly sensitive to small changes in steric bulk. This is attributed to the flanking bulky aryl substituents on the NacNac ligand restricting the accessible space around the zinc–alkyl moiety. This significantly impacts the degree of linearity of the S_E_2(open) type transition state for Zn–alkyl protonolysis by [(R_3_N)H]^+^. Combined, this all highlights that switching between methyl and ethyl is not always futile.

## Author contributions

JL, MKB, MJI and SAM conceived the research concept and aims and analyzed all data. JL performed the majority of the synthetic work and the majority of the analytical components of this project. GSN collected and solved all the crystal structures. JL performed all the calculations. Combined, all authors drafted, reviewed and edited the manuscript.

## Conflicts of interest

There are no conflicts to declare.

## Supplementary Material

DT-054-D5DT01816A-s001

DT-054-D5DT01816A-s002

DT-054-D5DT01816A-s003

## Data Availability

The data supporting this article has been uploaded as part of the SI: NMR spectra for all new compounds, *in situ* NMR spectra for catalytic and mechanistic reactions and Cartesian coordinates for all calculated structures. See DOI: https://doi.org/10.1039/d5dt01816a. CCDC 2454352 contains the supplementary crystallographic data for this paper.^30^
