# Supplementary material for: Catalytic transfer zincation using ammonium cations
Source: Dalton Trans. 2025 Aug 14;54(45):16697–703. doi: 10.1039/d5dt01816a (PMC13725691; doi:10.1039/d5dt01816a)
Supplement: Supplementary file 1 [file DT-054-D5DT01816A-s002.pdf]

Supplementary Information (SI) for Dalton Transactions.
This journal is © The Royal Society of Chemistry 2025

Supporting Information for:

Catalytic Transfer Zincation Using Ammonium Cations

Justyna Łosiewicz[a], Milan Kumar Bisai[a], Gary S. Nichol[a], Stuart A. Macgregor[b]* and Michael J. Ingleson[a]*

[a] EaStCHEM School of Chemistry, University of Edinburgh, Edinburgh, EH9 3FJ, UK.

[b] EaStCHEM School of Chemistry, University of St Andrews, St. Andrews, KY16 9ST, UK.

Contents

S1. General Considerations S2

S2. Synthesis of [Base(H)][Anion] S3

S3. Synthesis of Zn-aryl compounds S10

S4. Synthesis of aryl-BBN compounds S23

S5. Mechanistic Studies S37

S6. Crystallographic data S53

S7. Computational details S54

S8. References S89

S1. General Considerations

Unless otherwise mentioned, all the experiments were carried out under an inert atmosphere using either standard Schlenk techniques or in a MBraun glovebox (<0.1 ppm H_2O/O_2). Chlorobenzene, C_6D_6 and 1,2-difluorobenzene were distilled over CaH_2 and stored over activated 3 Å molecular sieves. C_6D_5Br, CD_3CN, $CDCl_3$ and CD_2Cl_2 were dried over activated 3 Å molecular sieves. All other solvents were obtained from an Inert PureSolv MD5 SPS and further dried over activated 3 Å molecular sieves. Unless otherwise stated all chemicals were purchased from commercial sources and used as received. ^{Dipp}NacNacH[1], [where ^{Dipp}NacNac = {2,6-*i*$Pr_2C_6H_3$NC(Me)}$_2$CH], ^{Dipp}NacNacZnH[2], [DMT(H)][$B(C_6F_5)_4$][3], (where, DMT = *N,N*-dimethyl-4-toluidine), [DMT(H)][B{$C_6H_3(CF_3)_2$}$_4$][4], [Et_3N(H)][B{$C_6H_3(CF_3)_2$}$_4$][5], [(2,4-$Br_2C_6H_3NMe_2$(H)][$B(C_6F_5)_4$][4], Cs[$CHB_{11}H_5Br_6$][6], [Ph_3C][$CHB_{11}H_5Br_6$][6] and 5-*d*-2-methylthiophene[7] were prepared as per reported literature procedures.

^1H, $^1H\{^{11}B\}$, $^{13}C\{^1H\}$, ^{11}B, $^{11}B\{^1H\}$ and ^{19}F NMR spectra were recorded on Bruker Advance III 500MHz or Bruker PRO 500 MHz spectrometers and referenced to the solvent in use for ^1H, $^1H\{^{11}B\}$ and $^{13}C\{^1H\}$, while ^{11}B, $^{11}B\{^1H\}$ and ^{19}F shifts are referenced relative to external $BF_3{\cdot}Et_2O$ and C_6F_6, respectively. Chemical shifts are reported as dimensionless δ values in ppm, coupling constants J are given in Hertz (Hz). The multiplicity of the signals is indicated as "br. s", "s", "d", "t" "q" "pent", "sept" or "m" for broad singlet, singlet, doublet, triplet, quartet, pentet, septet or multiplet, respectively. Background signals in ^{11}B and $^{11}B\{^1H\}$ NMR spectra arise to a significant degree from glass components of the NMR tubes used as well as probes used in the spectrometers. Unless otherwise stated, NMR spectroscopy was undertaken at room temperature (~27°C). Carbon atoms directly bonded to B are not observed in $^{13}C\{^1H\}$ NMR spectra due to quadrupolar broadening effects. Data analysis was carried out using MestReNova.

Mass spectrometry was performed by the Scottish Instrumentation and Resource Centre for Advanced Mass Spectrometry (SIRCAMS) at the University of Edinburgh. Mass spectrometry was performed using electrospray ionisation (ESI) techniques in either positive or negative (or both) modes depending on the sample. Data analysis was carried out using Data Analysis (Bruker).

S2. Synthesis of [Base(H)][Anion]

S2.1. Synthesis of **[(2,4,6-$(CH_3)_3C_6H_2NMe_2$)H][B(C_6F_5)$_4$]**

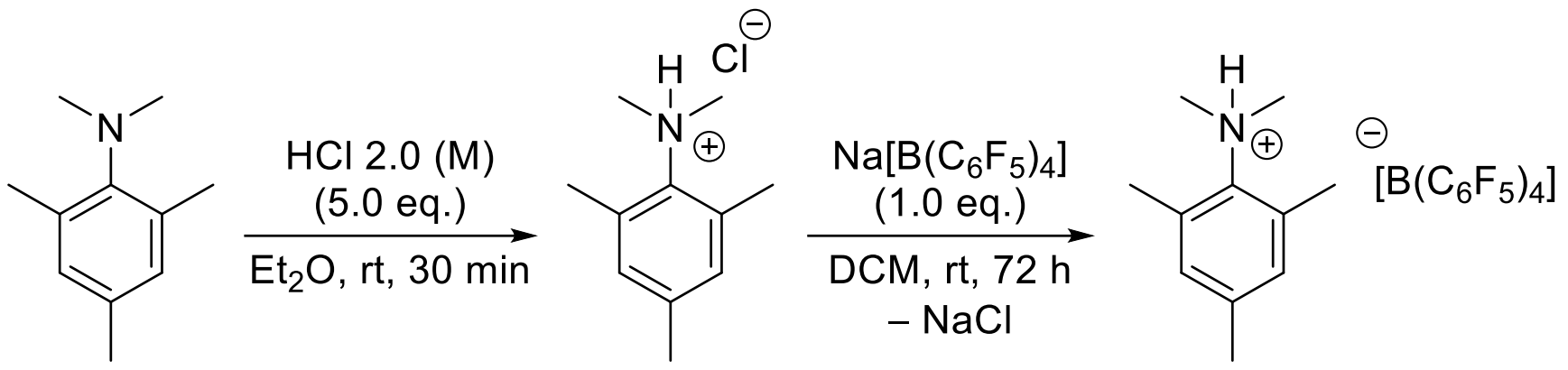

HCl (2 M in Et_2O, 700 µL, 1.4 mmol, 5.0 eq.) was slowly added to a solution of 2,4,6-$(CH_3)_3C_6H_2NMe_2$ (50 µL, 0.28 mmol, 1.0 eq.) in Et_2O (20 mL) prepared in an ampule at room temperature. The reaction was stirred for 30 minutes before all volatiles were removed *in vacuo* affording a white powder. Sodium tetrakis(pentafluorophenyl)borate (195 mg, 0.28 mmol, 1.0 eq.) followed by DCM (20 mL) were added to the flask and stirred for 72 hours at room temperature. The mixture was then filtered to remove NaCl and the volatiles removed *in vacuo* yielding an off-white powder. Yield: 68% (159 mg).

^{1}H NMR (500 MHz, CD_3CN): δ 8.07 (t, J = 36.9 Hz, 1H, *NH*), 7.05 (s, 1H, *Ar*), 7.04 (s, 1H, *Ar*), 3.30 (d, J = 5.2 Hz, 6H, N*(CH₃)₂*), 2.53 (s, 3H, o-*CH₃*), 2.43 (s, 3H, o-*CH₃*), 2.27 (s, 3H, p-*CH₃*).

^{13}C {^{1}H} NMR (126 MHz, CD_3CN): δ 149.1 [d, $^{1}J_{C\text{-}F}$ = 241.8 Hz, B(C_6F_5)$_4$], 141.5 (*Ar*), 139.3 [d, $^{1}J_{C\text{-}F}$ = 244.3 Hz, B(C_6F_5)$_4$], 137.5 (*Ar*), 137.3 [d, $^{1}J_{C\text{-}F}$ = 246.7 Hz, B(C_6F_5)$_4$], 134.5 (*Ar*), 131.8 (*Ar*), 131.4 (*Ar*), 131.3 (*Ar*), 125.1 (*C*–B) 46.3 (s, N$(CH_3)_2$), 20.5 (s, p-CH_3), 20.4 (s, o-CH_3), 18.5 (s, o-CH_3).

^{11}B NMR (160 MHz, CD_3CN): δ -16.7 (s).

^{19}F NMR (471 MHz, CD_3CN): δ -130.0 – -137.6 (m), -163.4 (t, J = 21.4 Hz), -167.5 (t, J = 21.2 Hz).

Mass Spectrometry: Calculated [M^+] = 164.1434, Observed [M^+] = 164.1432; Calculated [M^-] = 678.9779, Observed [M^-] = 678.9774.

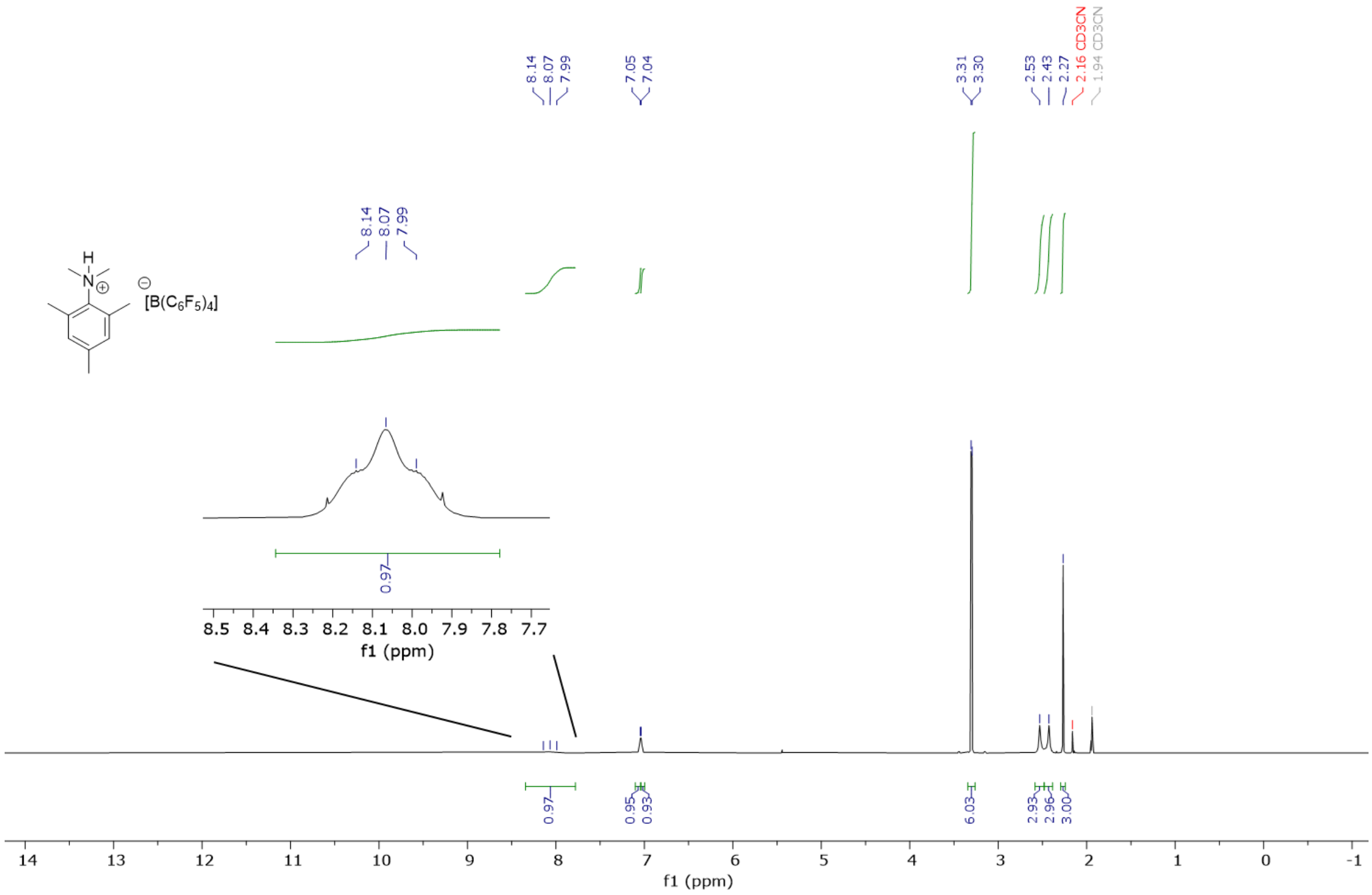

Figure S1: ^{1}H NMR spectrum of [(2,4,6-$(CH_3)_3C_6H_2NMe_2$)H][$B(C_6F_5)_4$] in CD_3CN.

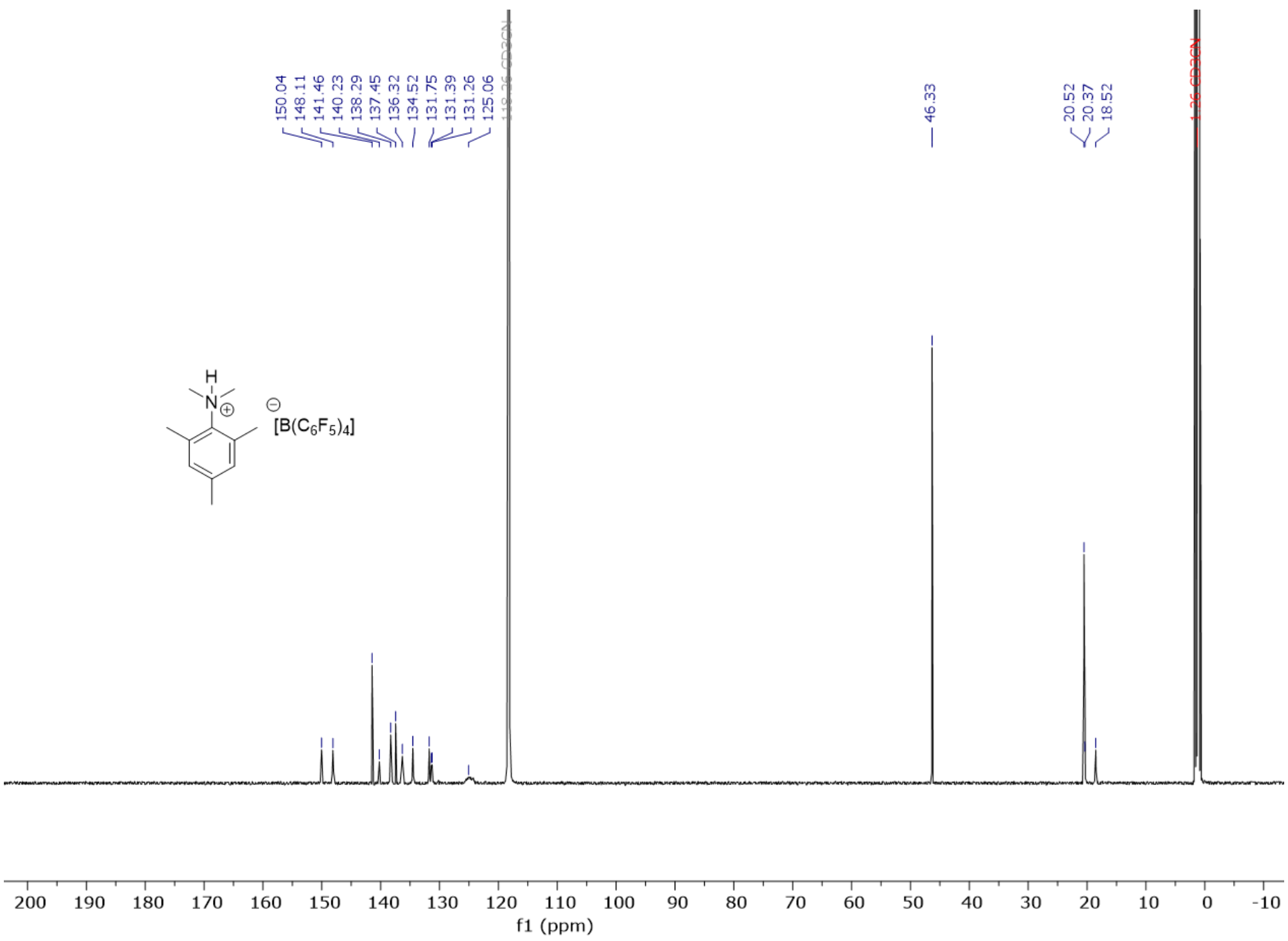

Figure S2: $^{13}C\{^{1}H\}$ NMR spectrum of [(2,4,6-$(CH_3)_3C_6H_2NMe_2$)H][$B(C_6F_5)_4$] in CD_3CN.

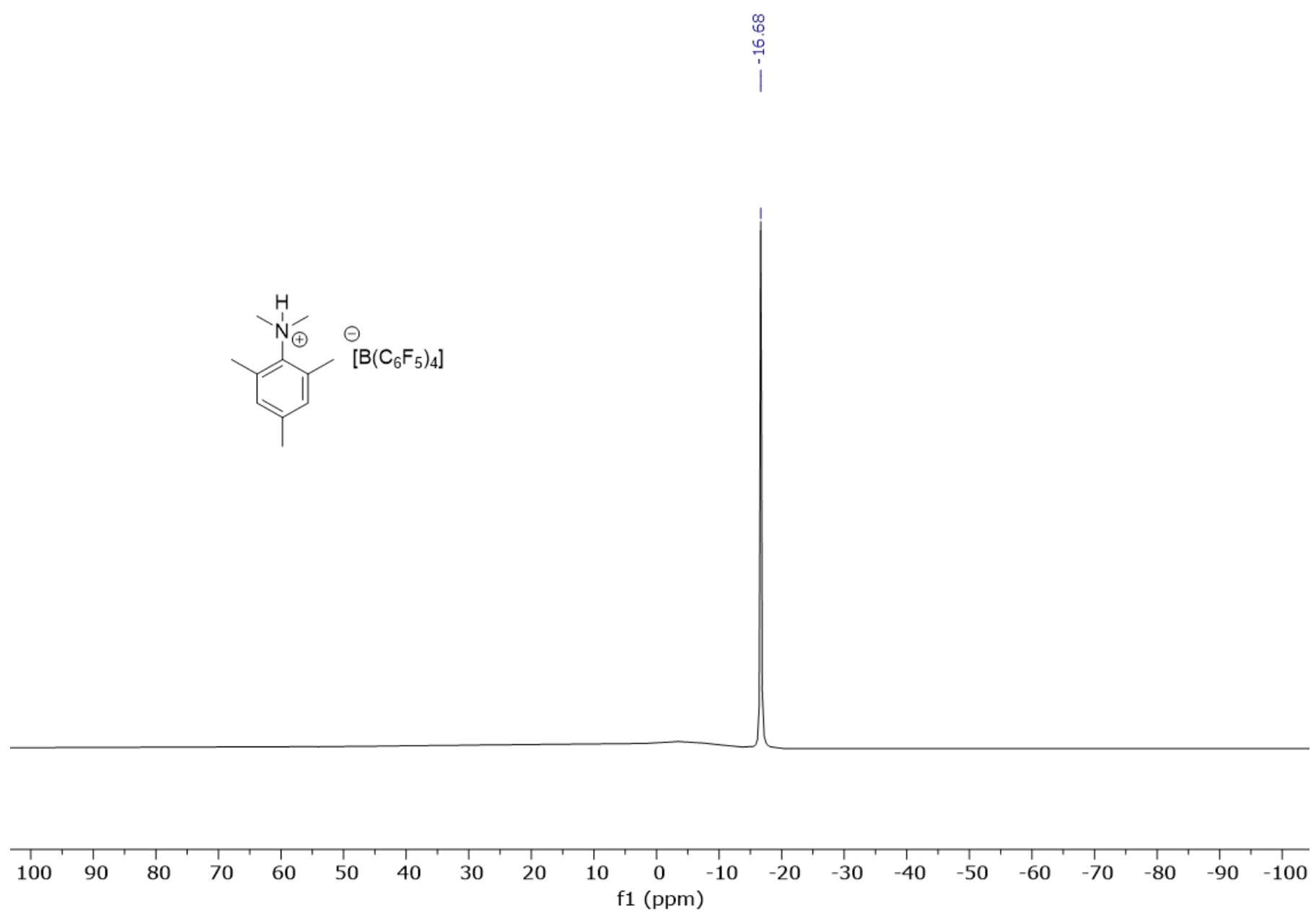

Figure S3: ^{11}B NMR spectrum of [(2,4,6-$(CH_3)_3C_6H_2NMe_2$)H][B$(C_6F_5)_4$] in CD_3CN.

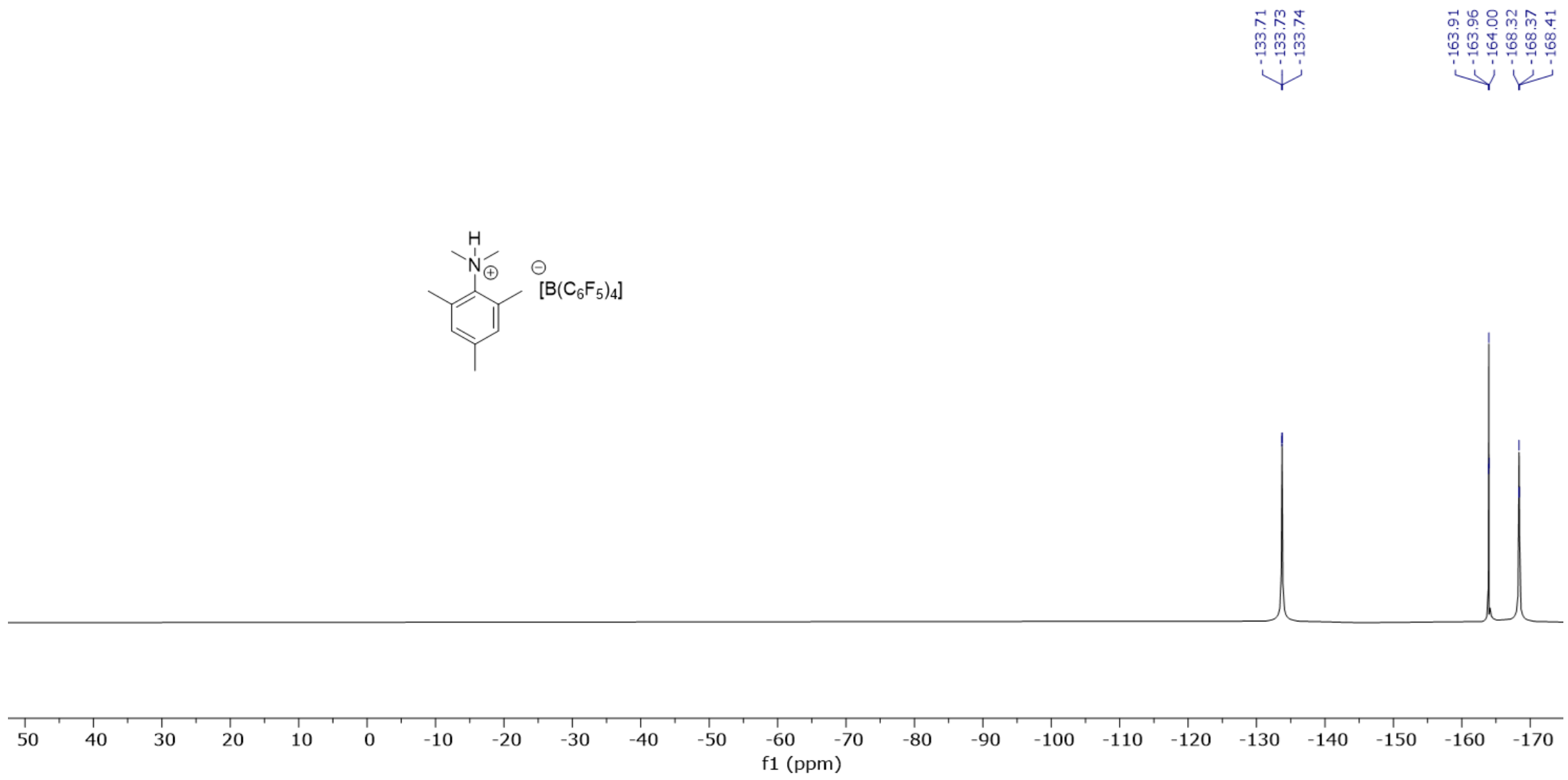

Figure S4: ^{19}F NMR spectrum of [(2,4,6-$(CH_3)_3C_6H_2NMe_2$)H][B$(C_6F_5)_4$] in CD_3CN.

S2.2. Synthesis of **[(DMT)H][$CHB_{11}H_5Br_6$]**

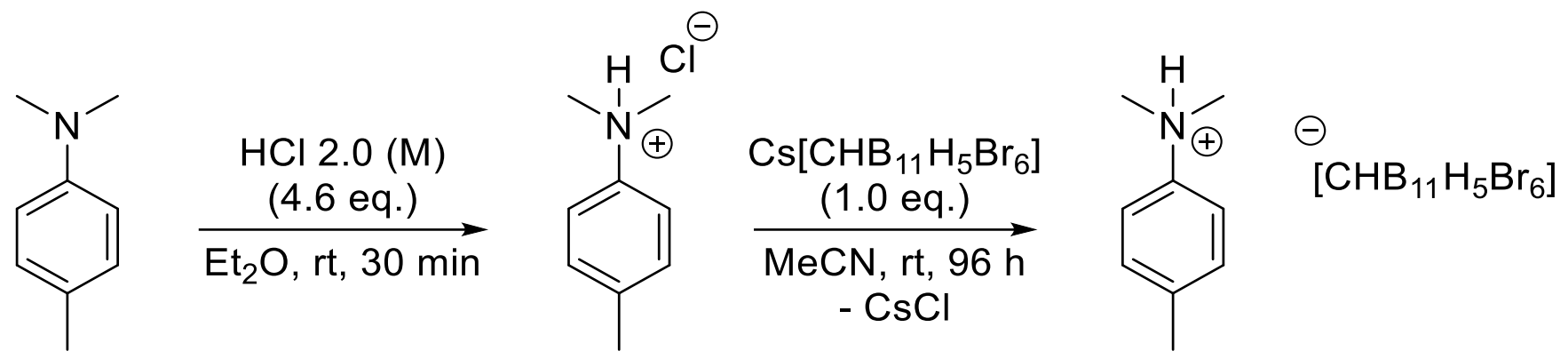

HCl (2 M in Et_2O, 400 µL, 0.8 mmol, 4.6 eq.) was slowly added to a solution of *N,N*-dimethyl-4-toluidine (DMT, 25 µL, 0.17 mmol, 1.0 eq.) in Et_2O (4 mL) prepared in an ampule at room temperature. The reaction was stirred for 30 minutes before all volatiles were removed *in vacuo* affording a white powder. $Cs(CHB_{11}H_5Br_6)$ (132 mg, 0.17 mmol, 1.0 eq.) followed by MeCN (12 mL) were added to the flask and stirred for 96 hours at room temperature. The mixture was then filtered, resulting in a clear yellow solution. The volatiles removed *in vacuo* overnight yielding a brown solid. DCM (5 mL) was added and the resulting solution was filtered to remove CsCl. Volatiles removed *in vacuo* yielding an off-white powder. Yield: 21% (28 mg).

^{1}H NMR (500 MHz, $CDCl_3$): δ 10.76 (br. s, 1H, *NH*), 7.54 (d, *J* = 8.6 Hz, 2H, ^{DMT}Ar), 7.36 (d, *J* = 8.2 Hz, 2H, ^{DMT}Ar), 3.32 (s, 6H, N$(CH_3)_2$), 2.59 (s, 1H, $^{carborane}CH$), 2.40 (s, 3H, $^{DMT}CH_3$). B–H peaks could not be resolved due to broadening and overlap.

^{1}H{^{11}B} NMR (500 MHz, $CDCl_3$): δ 10.76 (br. s, 1H, *NH*), 7.54 (d, *J* = 8.6 Hz, 2H, ^{DMT}Ar), 7.36 (d, *J* = 8.2 Hz, 2H, ^{DMT}Ar), 3.32 (s, 6H, N$(CH_3)_2$), 2.59 (s, 1H, $^{carborane}CH$), 2.40 (s, 3H, $^{DMT}CH_3$). B–H peaks could not be resolved due to broadening and overlap.

^{13}C{^{1}H} NMR (126 MHz, $CDCl_3$): δ 141.6 (^{DMT}Ar), 139.5 (^{DMT}Ar), 131.6 (^{DMT}Ar), 120.11 (^{DMT}Ar), 48.2 (N$(CH_3)_2$), 41.6 ($^{carborane}CH$), 21.3 (CH_3).

^{11}B NMR (160 MHz, $CDCl_3$): δ -1.6 (s), -9.8 (s), -20.1 (d, *J* = 169.5 Hz).

^{11}B{^{1}H} NMR (160 MHz, $CDCl_3$): δ -1.6 (s), -9.8 (s), -20.1 (s).

Mass Spectrometry: Calculated [M^+] = 136.1121, Observed [M^+] = 136.1122; Calculated [M^-] = 616.6613, Observed [M^-] = 616.6603.

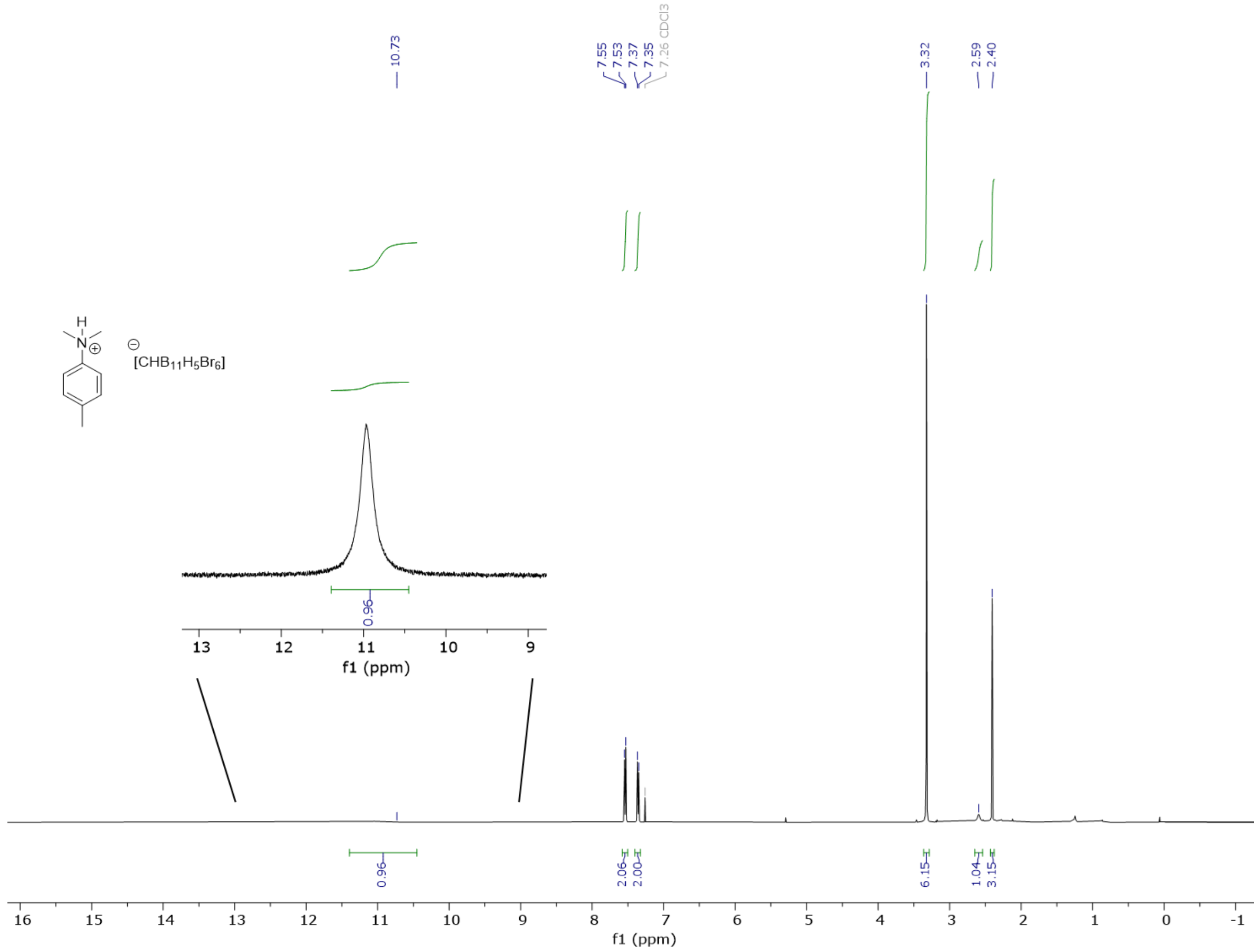

Figure S5: ^{1}H NMR spectrum of $[(DMT)H][CHB_{11}H_5Br_6]$ in $CDCl_3$.

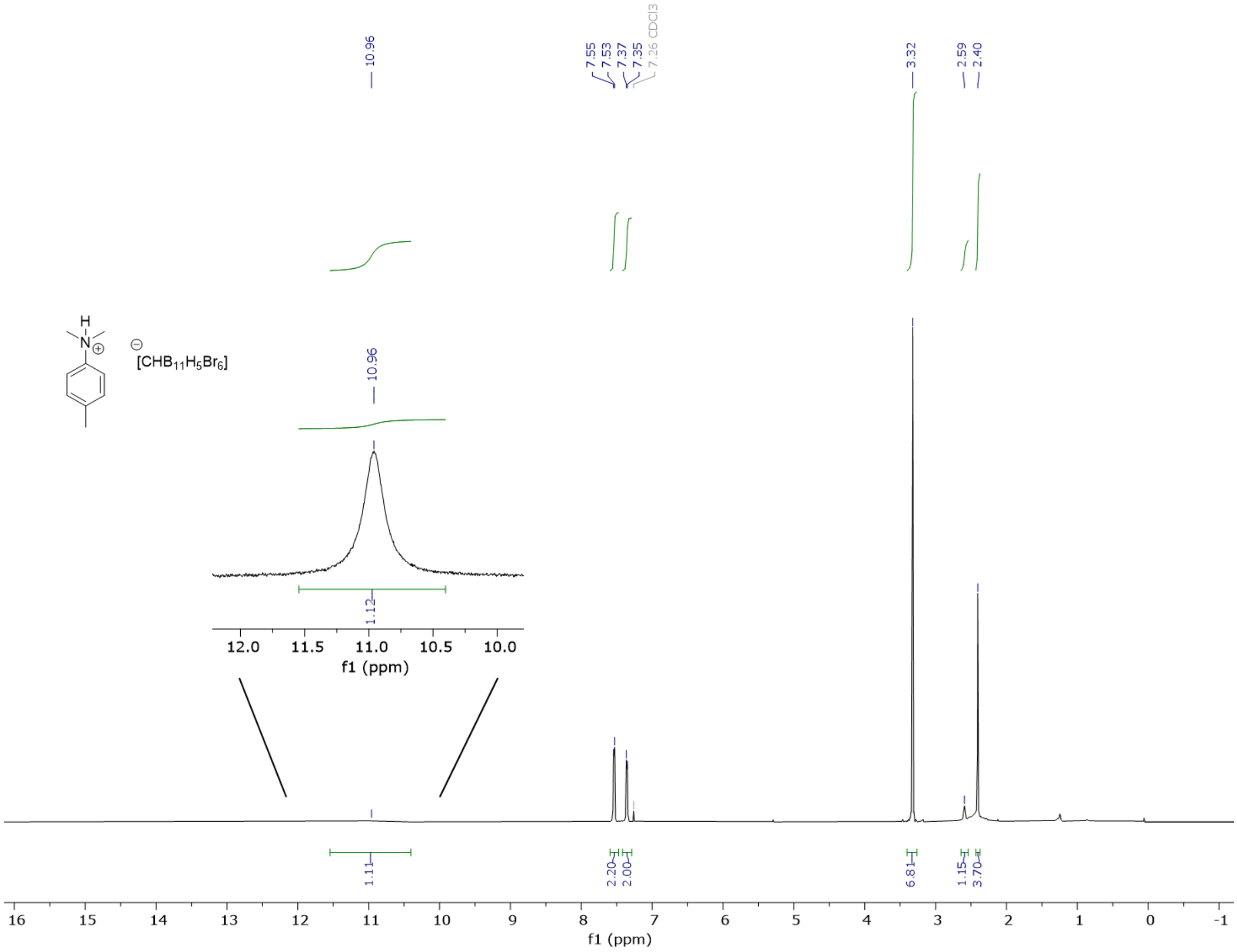

Figure S6: $^{1}H\{^{11}B\}$ NMR spectrum of $[(DMT)H][CHB_{11}H_5Br_6]$ in $CDCl_3$.

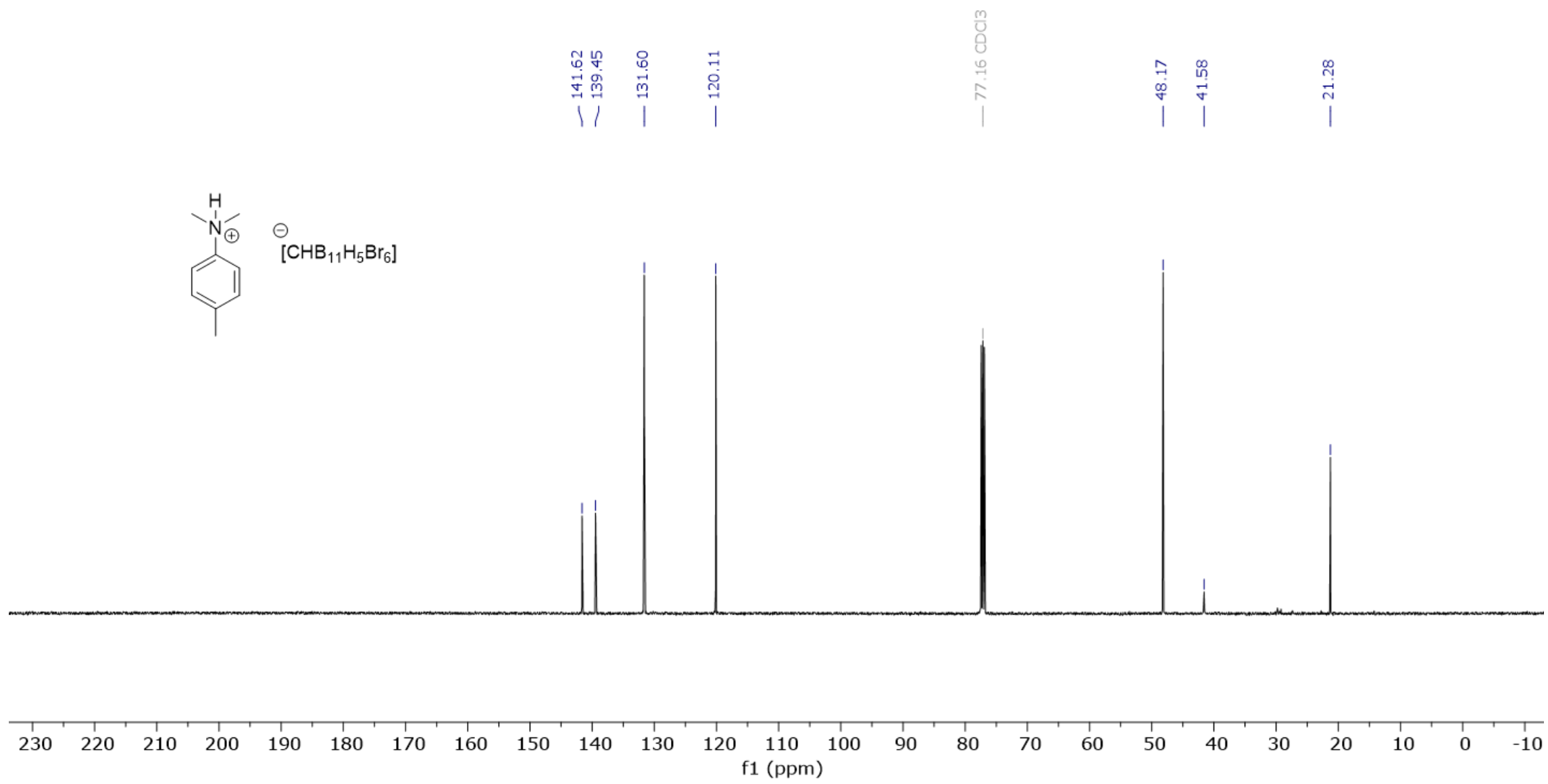

Figure S7: $^{13}C\{^{1}H\}$ NMR spectrum of $[(DMT)H][CHB_{11}H_5Br_6]$ in $CDCl_3$.

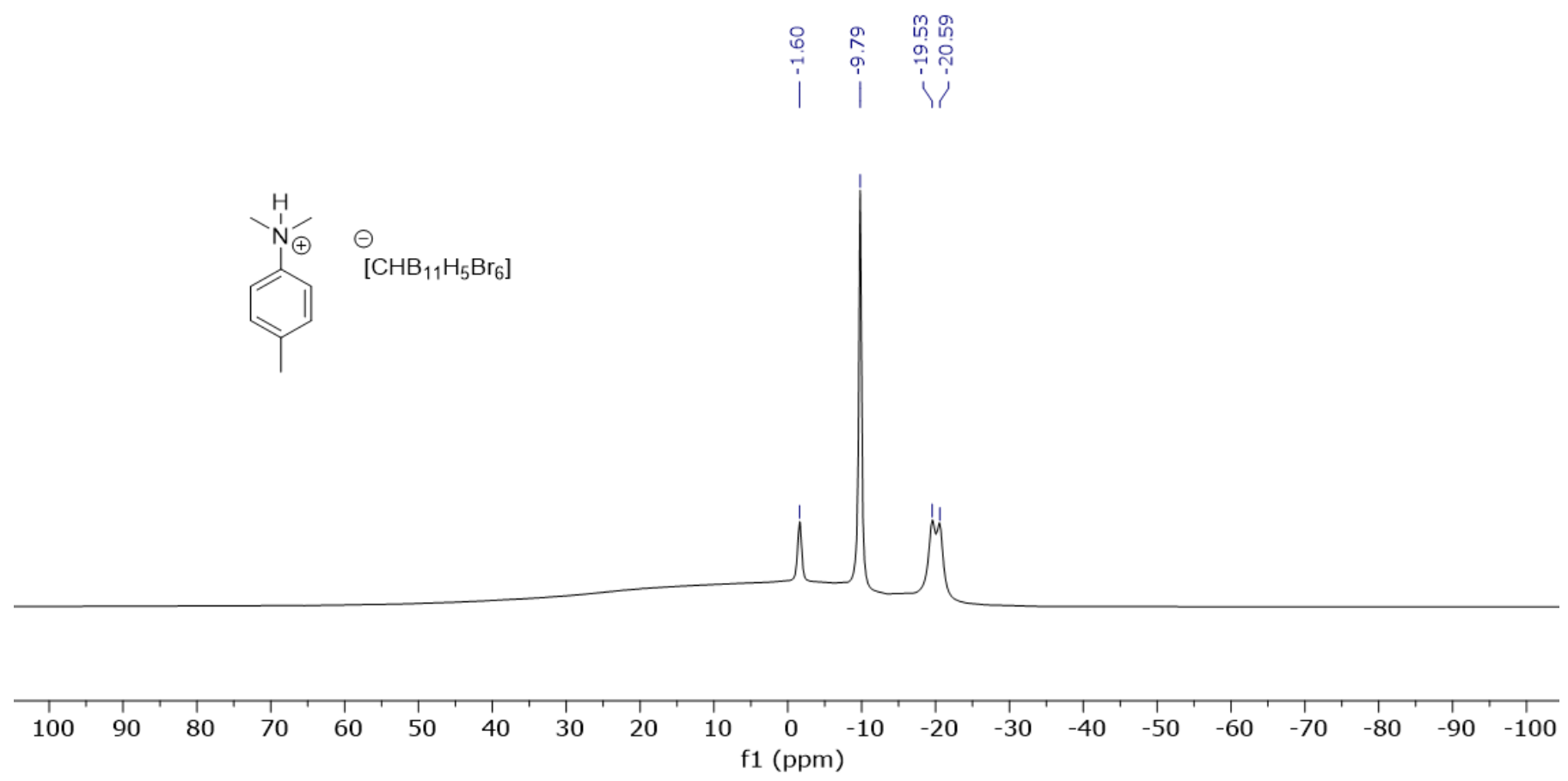

Figure S8: ^{11}B NMR spectrum of [(DMT)H][$CHB_{11}H_5Br_6$] in CD_2Cl_2.

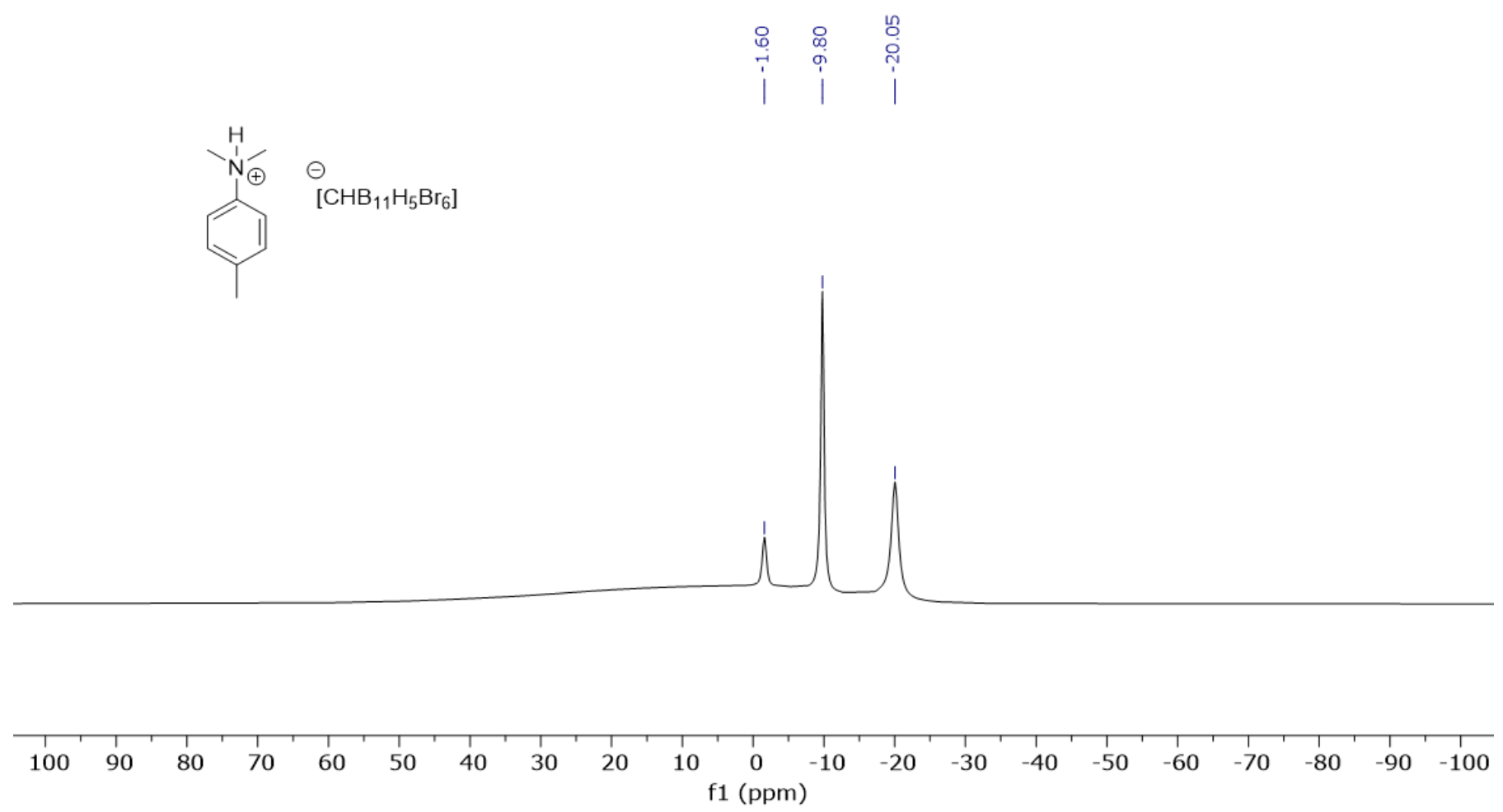

Figure S9: $^{11}B\{^1H\}$ NMR spectrum of [(DMT)H][$CHB_{11}H_5Br_6$] in $CDCl_3$.

S3. Synthesis of Zn-aryl compounds

S3.1. Synthesis of ^{Dipp}NacNacZnMe

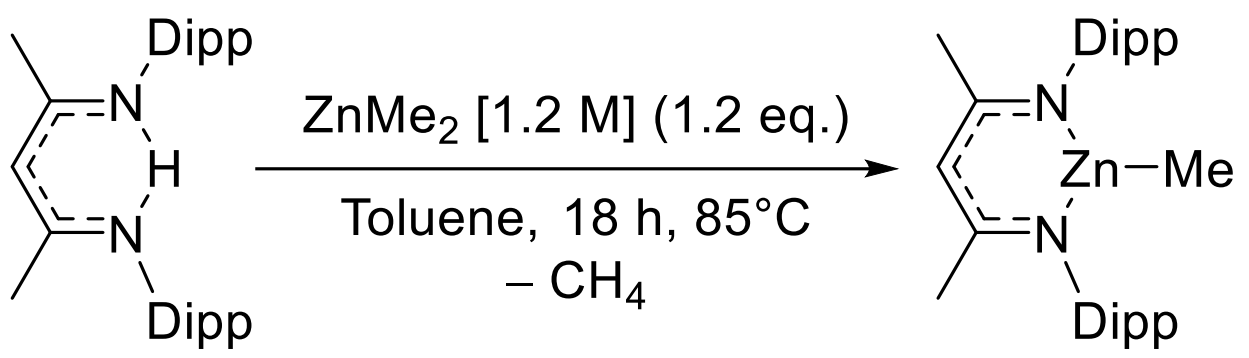

Synthesis according to a modified literature procedure.[8] $ZnMe_2$ (1.2 M in toluene, 8.8 mL, 10.6 mmol, 1.2 eq.) was added at ambient temperature to a solution of ^{Dipp}NacNacH (3.67 g, 8.8 mmol, 1 eq.) in toluene. The solution was heated at 85°C for 18 h when all gas evolution stopped. The volatiles were removed *in vacuo* yielding a white powder. Yield: 96% (4.1 g).

^{1}H NMR (500 MHz, C_6D_6): δ 7.13 – 7.11 (m, 6H, *Ar*), 5.02 (s, 1H, C_γ–*H*), 3.20 (sept, J = 6.9 Hz, 4H, *CH*(CH_3)$_2$), 1.71 (s, 6H, CH(NCCH_3)$_2$), 1.24 (d, J = 6.9 Hz, 12H, CH(CH_3)$_2$), 1.17 (d, J = 6.9 Hz, 12H, CH(CH_3)$_2$), -0.67 (s, 3H, ZnCH_3).

^{13}C{^{1}H} NMR (126 MHz, C_6D_6): δ 167.6 (s, CH(N*C*CH_3)$_2$), 144.7 (s, Ar-*C*N), 141.7 (s, Ar-*C*CH(CH_3)$_2$), 126.1 (s, p-Ar-*CH*), 123.9 (s, m-Ar-*CH*), 95.6 (s, C_γ–*H*), 28.5 (s, *CH*(CH_3)$_2$), 24.4 (s, CH(CH_3)$_2$), 23.4 (s, CH(NCCH_3)$_2$), -16.4 (ZnCH_3).

Analytical data are consistent with that previously reported.[8]

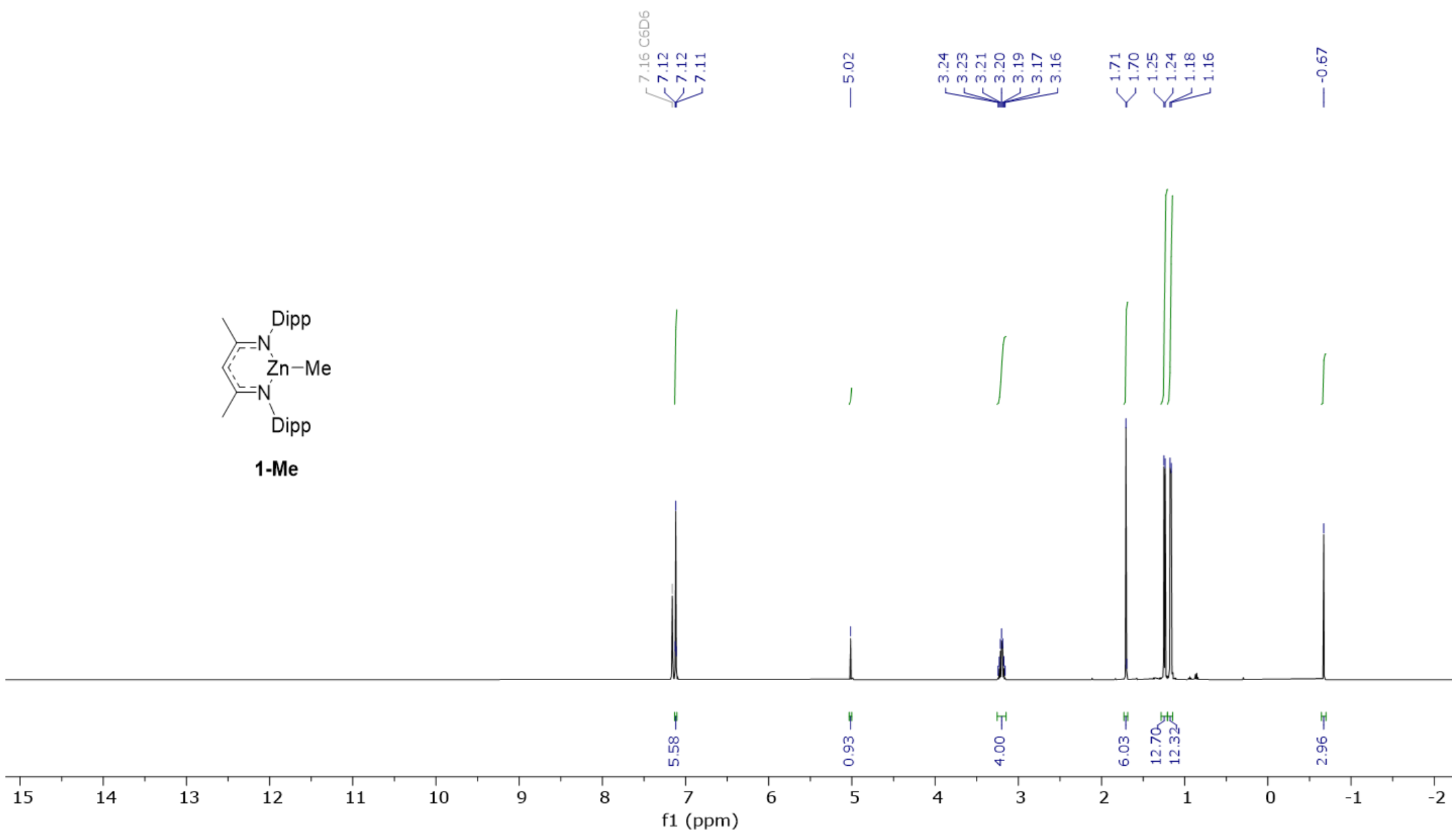

Figure S10: ^{1}H NMR spectrum of ^{Dipp}NacNacZnMe, compound **1-Me**, in C_6D_6.

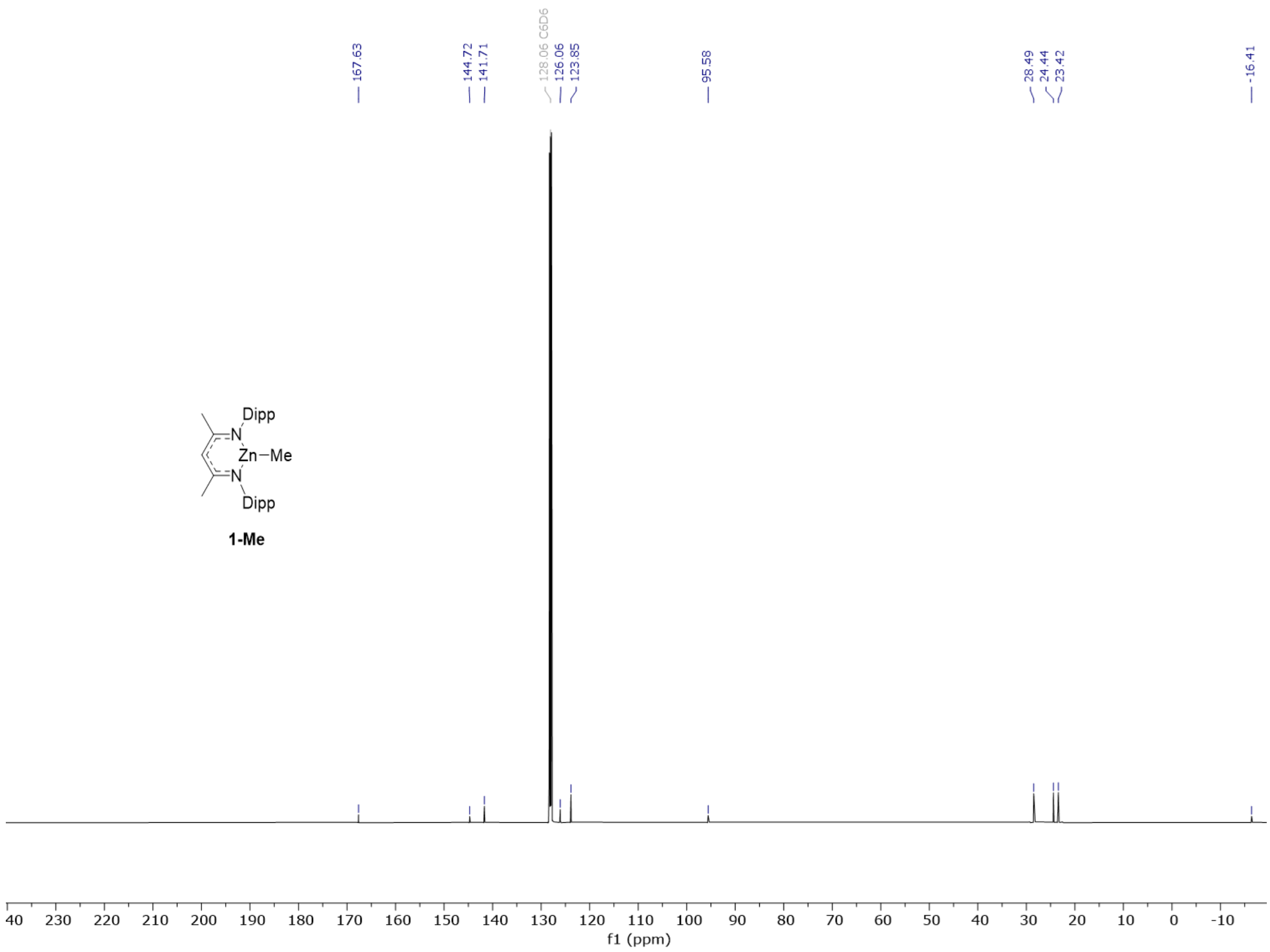

Figure S11: $^{13}C\{^{1}H\}$ NMR spectrum of ^{Dipp}NacNacZnMe, compound **1-Me**, in C_6D_6.

S3.2. General procedure 1: Optimization of the catalytic C−H zincation of 2-methyl-thiophene

In a glovebox, ^{Dipp}NacNacZnMe (50.0 mg, 0.10 mmol) and [(Base)H][Anion] (0.01 mmol) charged in a J. Young's NMR tube were dissolved in PhCl (0.6 mL). Subsequently, 2-methyl-thiophene (20 μL, 0.2 mmol, 2.0 equiv.) was added to the reaction mixture and heated at a specified temperature for 24 h unless stated otherwise. Upon completion, the reaction mixture was vented and the *in-situ* conversion was determined by the integration of diagnostic ^{1}H (NacNac C_γ–H in the product versus unreacted NacNac C_γ–H in the starting material) resonances.

Table S1. Optimisation of C−H zincation of 2-methyl-thiophene[a].

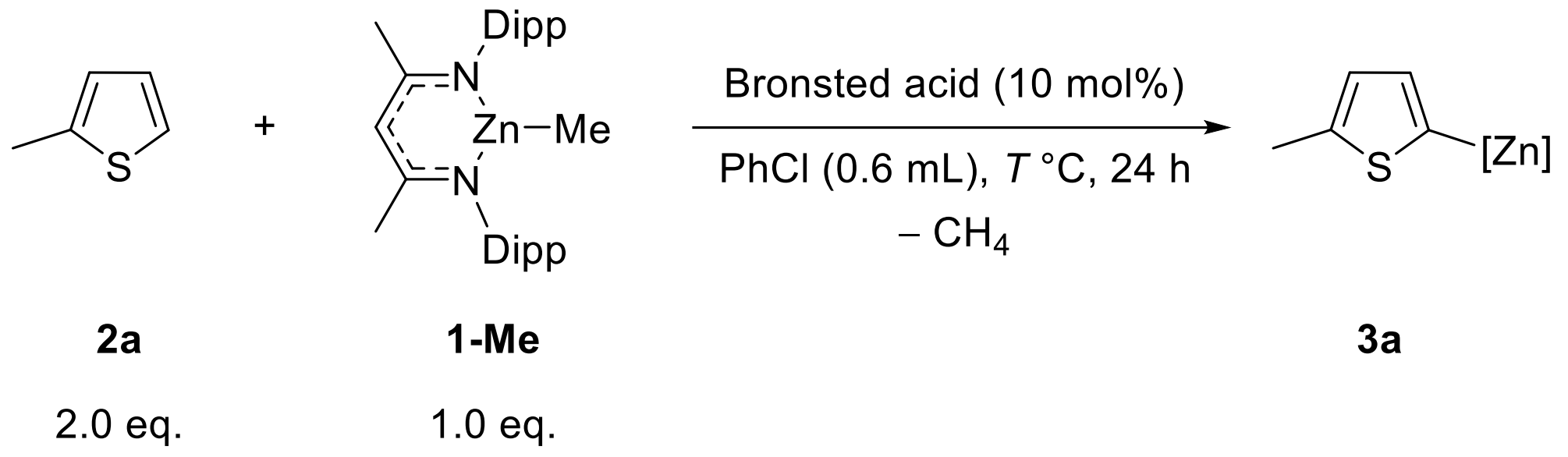

Entry	Brønsted Acid	T (ºC)	Con. (%)[b]
**1**	$[(DMT)H][B(C_6F_5)_4]$	100	63.0
**2**[c]	$[(DMT)H][B(C_6F_5)_4]$	100	60.0
**3**[d]	$[(DMT)H][B(C_6F_5)_4]$	100	63.0
**4**[e]	$[(DMT)H][B(C_6F_5)_4]$	100	75.0
**5**	$[(2,4\text{-}Br_2C_6H_3NMe_2)H][B(C_6F_5)_4]$	100	43.0
**6**	$[(DMT)H][B\{C_6H_3(CF_3)_2\}_4]$	100	39.0[f]
**7**	$[(Et_3N)H][B\{C_6H_3(CF_3)_2\}_4]$	100	46.0
**8**	$[(DMT)H][B(C_6F_5)_4]$	110	80.0
**9**	$[(2,4,6\text{-}(CH_3)_3C_6H_2NMe_2)H][B(C_6F_5)_4]$	110	62.0[f]
**10**	$[(DMT)H][CHB_{11}H_5Br_6]$	110	28.0
**11**	$[(DMT)H][CHB_{11}H_5Br_6]$	140	56.0
**12**[g]	$[(DMT)H][B(C_6F_5)_4]$	110	87.0
**13**	-	110	0

[a] 2-methyl-thiophene (2.0 equiv.), ^{Dipp}NacNacZnMe (1.0 equiv), and Brønsted acid (0.1 equiv) in PhCl (0.6 mL), [0.16 M]. [b] conversion by ^{1}H NMR spectroscopy NacNac C_γ–H in the product versus NacNac C_γ–H in the starting material. [c] reaction carried out in 1,2-dichlorobenzene. [d] reaction carried out in 1,2-difluorobenzene. [e] reaction carried out for 48 h. [f] significant anion decomposition observed followed by ^{19}F NMR spectroscopy. [g] reaction run at double concentration [0.32 M].

S3.3. Attempted C–H zincation with [(DMT)H][$B(C_6F_5)_4$] at a higher temperature

In a glovebox, ^{Dipp}NacNacZnMe (50.0 mg, 0.10 mmol) and [(DMT)H][$B(C_6F_5)_4$] (0.01 mmol) charged in a J. Young's NMR tube were dissolved in PhCl (0.6 mL). Subsequently, 2-methyl-thiophene (20 µL, 0.2 mmol, 2.0 equiv.) was added to the reaction mixture and the reaction mixture was heated at 140°C for 6 h. Formation of the ^{Dipp}NacNacZn−C_6F_5 was observed in ca. 40% conversion by the integration of diagnostic ^{1}H NMR resonances. ^{11}B and ^{19}F NMR spectra further confirmed almost complete decomposition of $[B(C_6F_5)_4]^-$ anion.

Analytical data are consistent with that previously reported.[4]

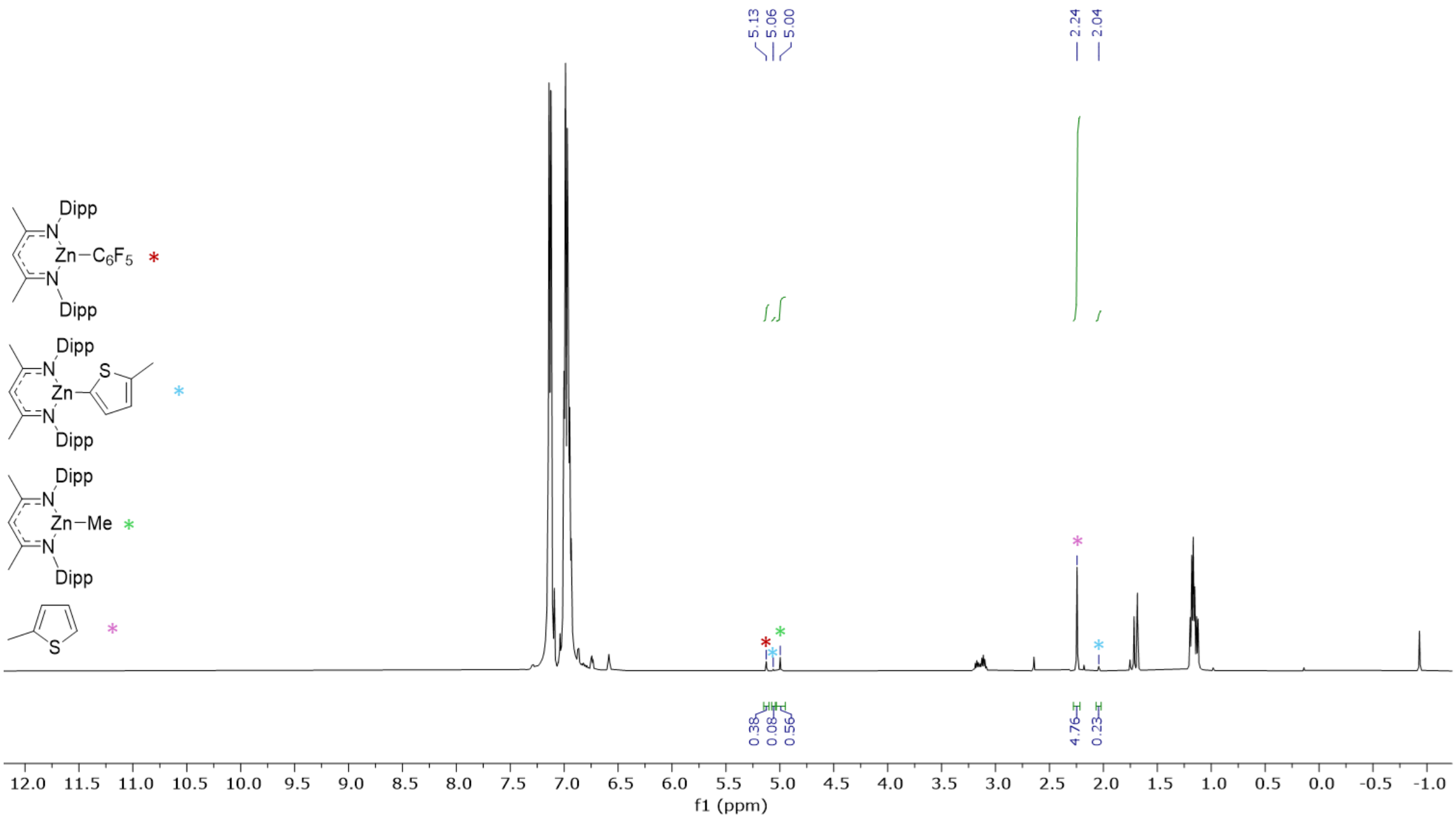

Figure S12: Anion decomposition to ^{Dipp}NacNacZn−C_6F_5 observed for C–H zincation of 2-methyl-thiophene in PhCl by *in-situ* ^{1}H NMR spectroscopy.

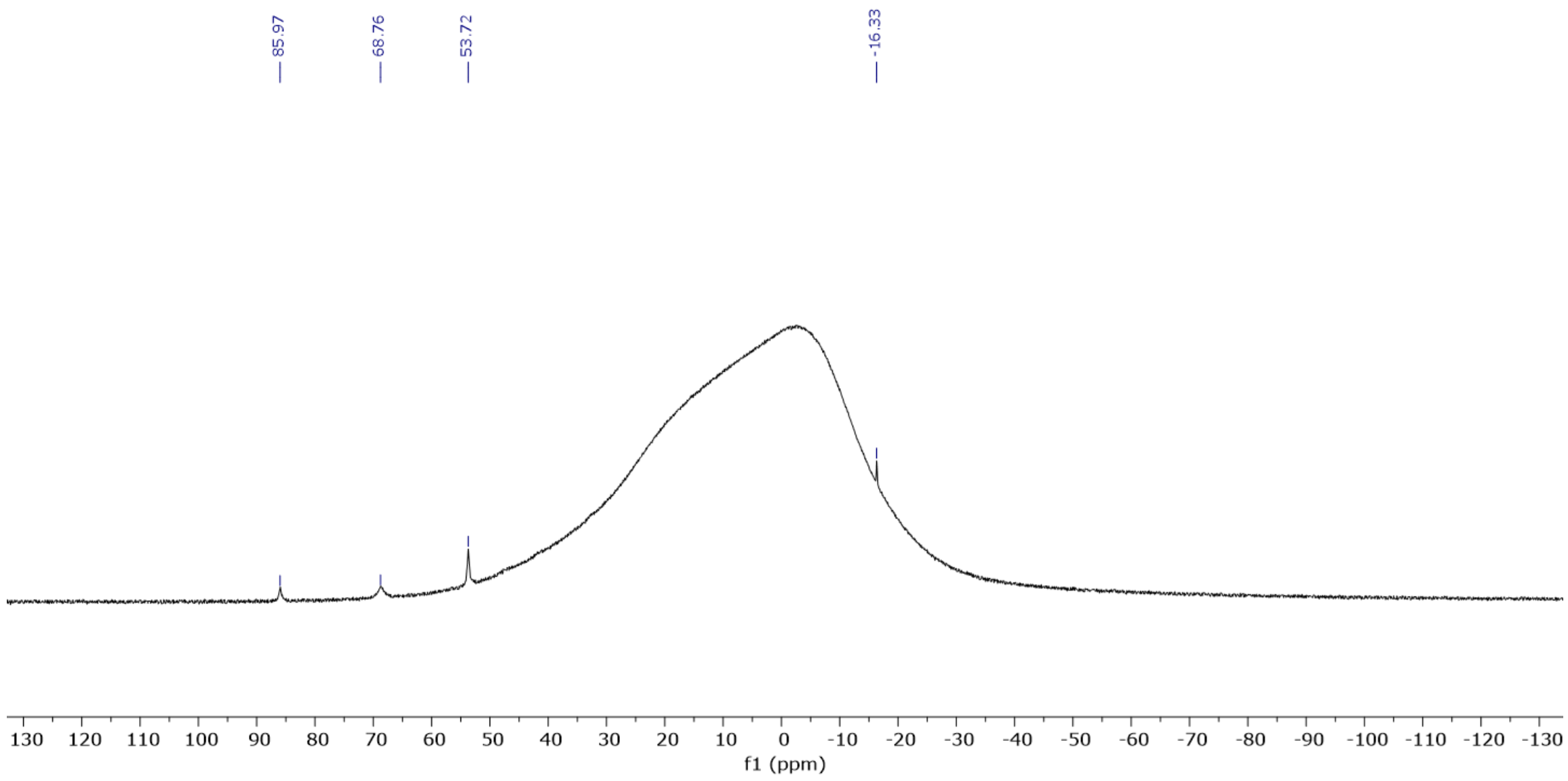

Figure S13: Anion decomposition to ^{Dipp}NacNacZn−C_6F_5 observed for C–H zincation of 2-methyl-thiophene in PhCl by *in-situ* ^{11}B NMR spectroscopy.

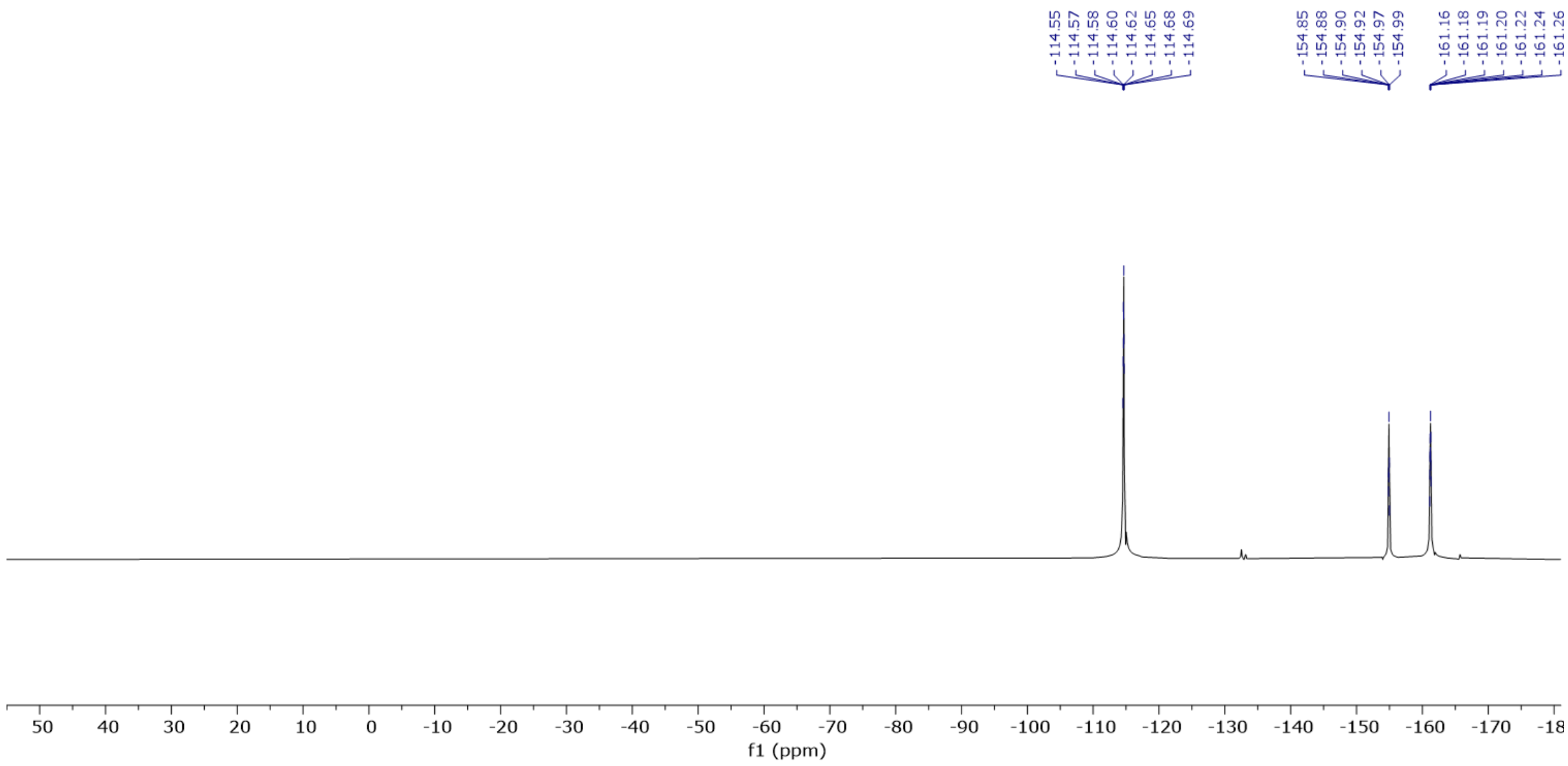

Figure S14: Anion decomposition to ^{Dipp}NacNacZn−C_6F_5 observed for C–H zincation of 2-methyl-thiophene in PhCl by *in-situ* ^{19}F NMR spectroscopy.

S3.4. General procedure 2: Catalytic C–H zincation of (hetero)arenes

In a glovebox, ^{Dipp}NacNacZnMe (100.0 mg, 0.20 mmol) and [(DMT)H][B(C_6F_5)$_4$] (16 mg, 0.02 mmol) charged in a J. Young's NMR tube were dissolved in PhCl (0.6 mL). Subsequently, the corresponding substrate (0.40 mmol, 2.0 equiv. w.r.t. ^{Dipp}NacNacZnMe) was added to the reaction mixture and heated at 110°C for 24 h. Upon completion, the reaction mixture was vented and dibromomethane (28.0 µL, 0.4 mmol) was added to the reaction mixture as an internal standard to determine *in-situ* yield by the integration of diagnostic ^{1}H (e.g., NacNac C_γ–H) resonances. In cases where the diagnostic peak in the ^{1}H NMR spectrum was obscured by chlorobenzene solvent, the reaction mixture was dried, redissolved in C_6D_6 and dibromomethane (28.0 µL, 0.4 mmol) was added to determine the *in-situ* yield.

To identify the product, the reaction mixture was dried and the residue was redissolved in C_6D_6 to obtain NMR spectrum from the crude reaction mixture. In each case, analytical data were consistent with that previously reported.

S3.4.1. Synthesis of ^{Dipp}NacNacZn-(5-methyl-thiophene-2-yl), **3a**

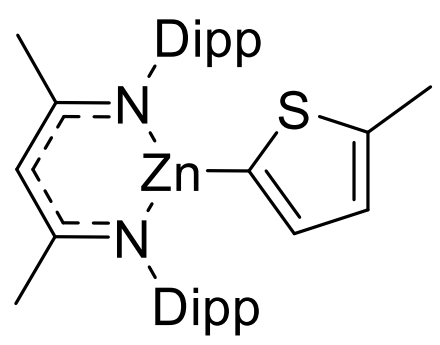

3a

As per general procedure 1, using 2-methyl-thiophene (20.0 µL, 0.40 mmol, 2.0 equiv.) and heating at 100°C for 24 h. *In-situ* yield by integration of diagnostic ^{1}H resonances (87% yield by ^{1}H NMR spectroscopy).

^{1}H NMR (500 MHz, C_6D_6): δ 7.25 – 7.22 (m, 2H, *Ar*), 7.20 – 7.18 (m, 4H, *Ar*), 6.61 (dd, *J* = 3.2, 1.1 Hz, 1H, ^{Thiophene}*CH*), 6.15 (d, *J* = 3.2 Hz, 1H, ^{Thiophene}*CH*), 5.05 (s, 1H, C_γ–*H*), 3.20 (sept., *J* = 6.8 Hz, 4H, *CH*(CH_3)$_2$), 2.08 (d, *J* = 1.1 Hz, 3H, ^{Thiophene}*CH*$_3$), 1.76 (s, 6H, CH(NC*CH$_3$*)$_2$), 1.26 (d, *J* = 6.9 Hz, 12H, CH(*CH$_3$*)$_2$), 1.17 (d, *J* = 6.9 Hz, 12H, CH(*CH$_3$*)$_2$).

^{13}C{^{1}H} NMR (126 MHz, C_6D_6): δ 168.5 (CH(N*C*CH$_3$)$_2$), 144.4 (*Ar*), 144.1 (*Ar*), 141.8 (*Ar*), 140.0 (*Ar*), 137.1 (*Ar*), 126.6 (*Ar*), 126.5 (*Ar*), 124.2 (*Ar*), 95.6 (C_γ–*H*), 28.7 (*CH*(CH$_3$)$_2$), 24.7 (CH(*CH$_3$*)$_2$), 23.6 (CH(*CH$_3$*)$_2$), 23.3 (CH(NC*CH$_3$*)$_2$), 14.8 (^{Thiophene}*CH$_3$*).

Analytical data are consistent with that previously reported.[4]

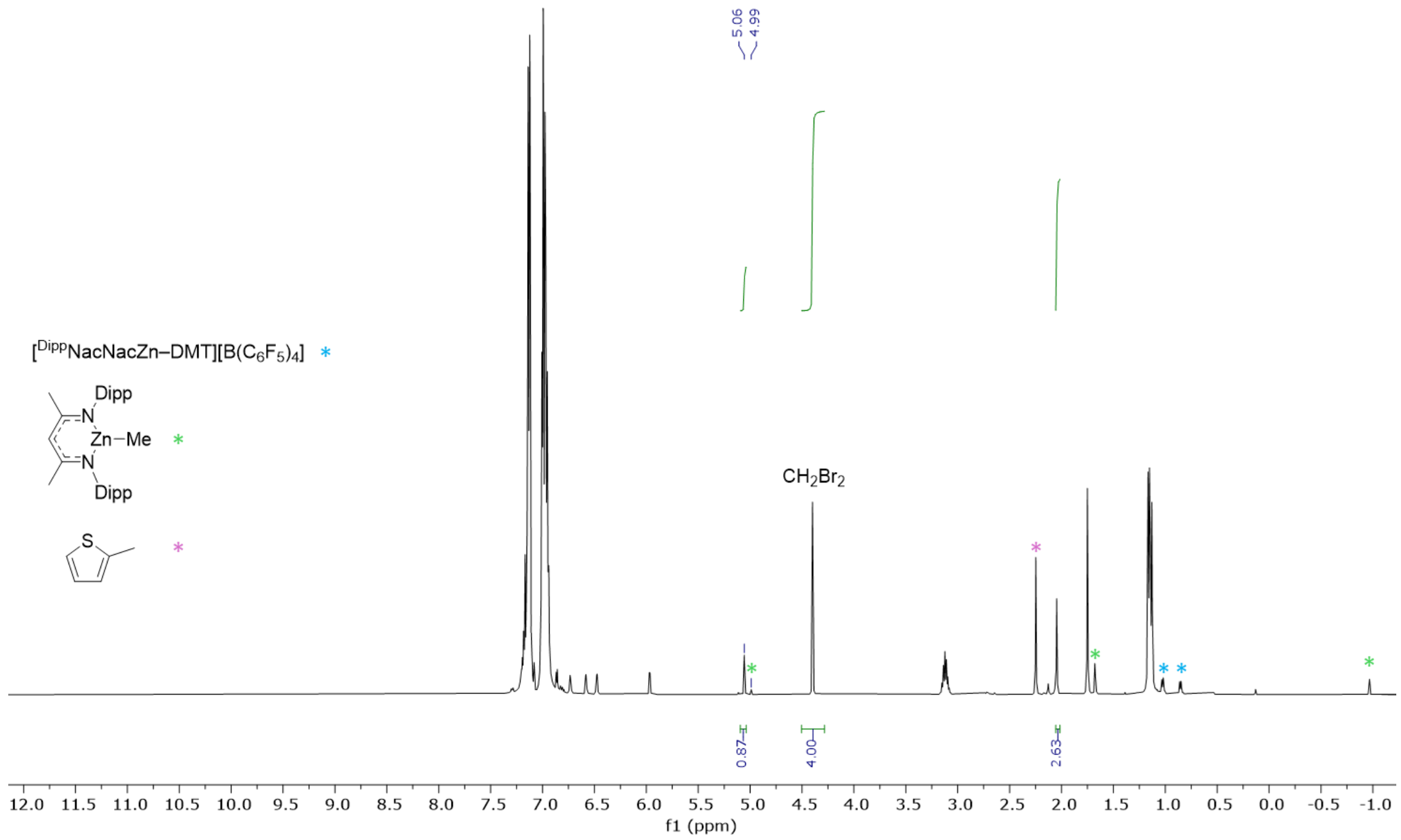

Figure S15: C–H zincation of 2-methyl-thiophene in PhCl by *in-situ* ^{1}H NMR spectroscopy.

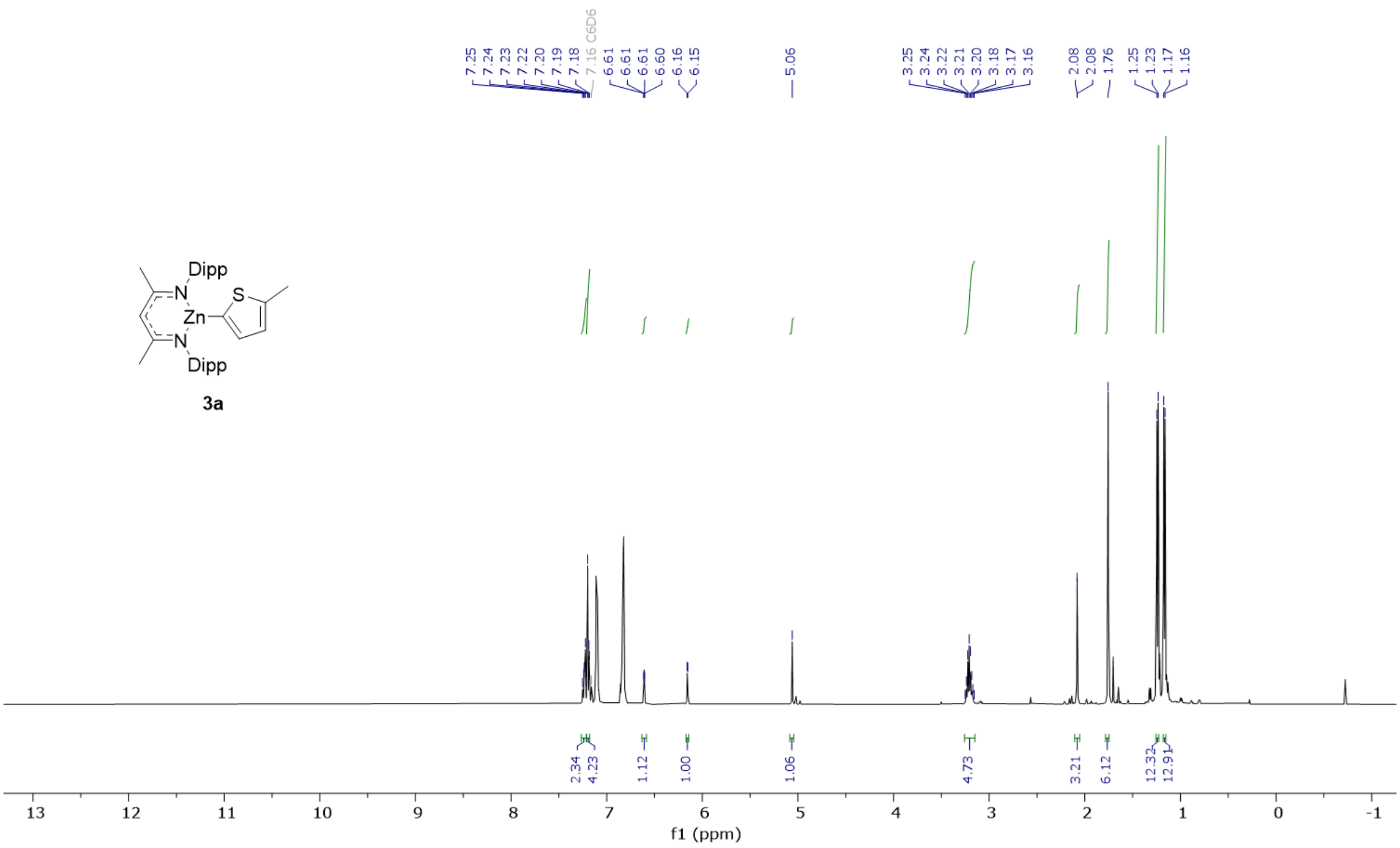

Figure S16: ^{1}H NMR spectrum of crude compound **3a** in C_6D_6.

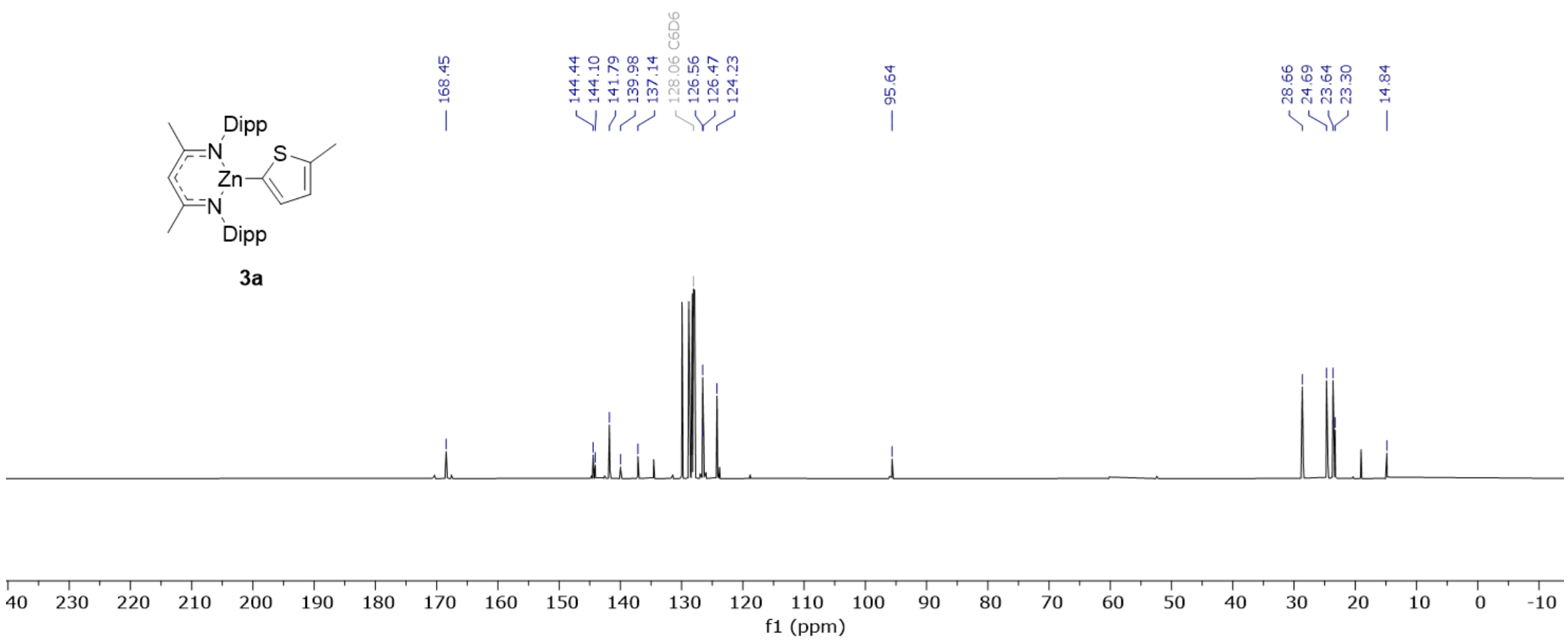

Figure S17: $^{13}C\{^{1}H\}$ NMR spectrum of crude compound **3a** in C_6D_6.

S3.4.2. Synthesis of ^{Dipp}NacNacZn-(5-phenylthiophene-2-yl), **3b**

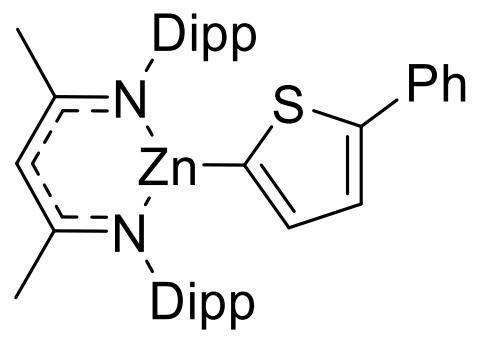

3b

As per general procedure 1, using 2-phenyl-thiophene (64.0 mg, 0.40 mmol, 2.0 equiv.) and heating at 100°C for 24 h. *In-situ* yield by integration of diagnostic ^{1}H resonances (99% yield by ^{1}H NMR spectroscopy).

^{1}H NMR (500 MHz, C_6D_6): δ 7.39 – 7.34 (m, 2H, *Ar*), 7.24 – 7.18 (m, 2H, *Ar*), 7.18 – 7.13 (m, 4H, *Ar*), 7.10 (d, J = 3.3 Hz, 1H, ^{Thiophene}*CH*), 7.01 (dd, J = 3.6 Hz, J = 1.0 Hz, 1H, *Ar*), 6.96 (dt, J = 7.9 Hz, J = 1.6 Hz, 1H, *Ar*), 6.23 (d, J = 3.3 Hz, 1H, ^{Thiophene}*CH*), 5.01 (s, 1H, C_γ–*H*), 3.16 (sept., J = 7.0 Hz, 4H, *CH*(CH_3)$_2$), 1.70 (s, 6H, CH(NCCH_3)$_2$), 1.19 (d, J = 6.9 Hz, 12H, CH(CH_3)$_2$), 1.12 (d, J = 6.9 Hz, 12H, CH(CH_3)$_2$).

^{13}C{^{1}H} NMR (126 MHz, C_6D_6): δ 168.6 (CH(N*C*CH_3)$_2$), 144.4 (*Ar*), 141.8 (*Ar*), 134.9 (*Ar*), 129.9 (*Ar*), 129.2 (*Ar*), 128.8 (*Ar*), 127.6 (*Ar*), 126.6 (*Ar*), 126.3 (*Ar*), 126.1 (*Ar*), 124.3 (*Ar*), 123.4 (*Ar*), 95.8 (C_γ–*H*), 28.7 (*CH*(CH_3)$_2$), 24.7 (CH(CH_3)$_2$), 23.6 (CH(CH_3)$_2$), 23.3 (CH(NCCH_3)$_2$).

Analytical data are consistent with that previously reported.[4]

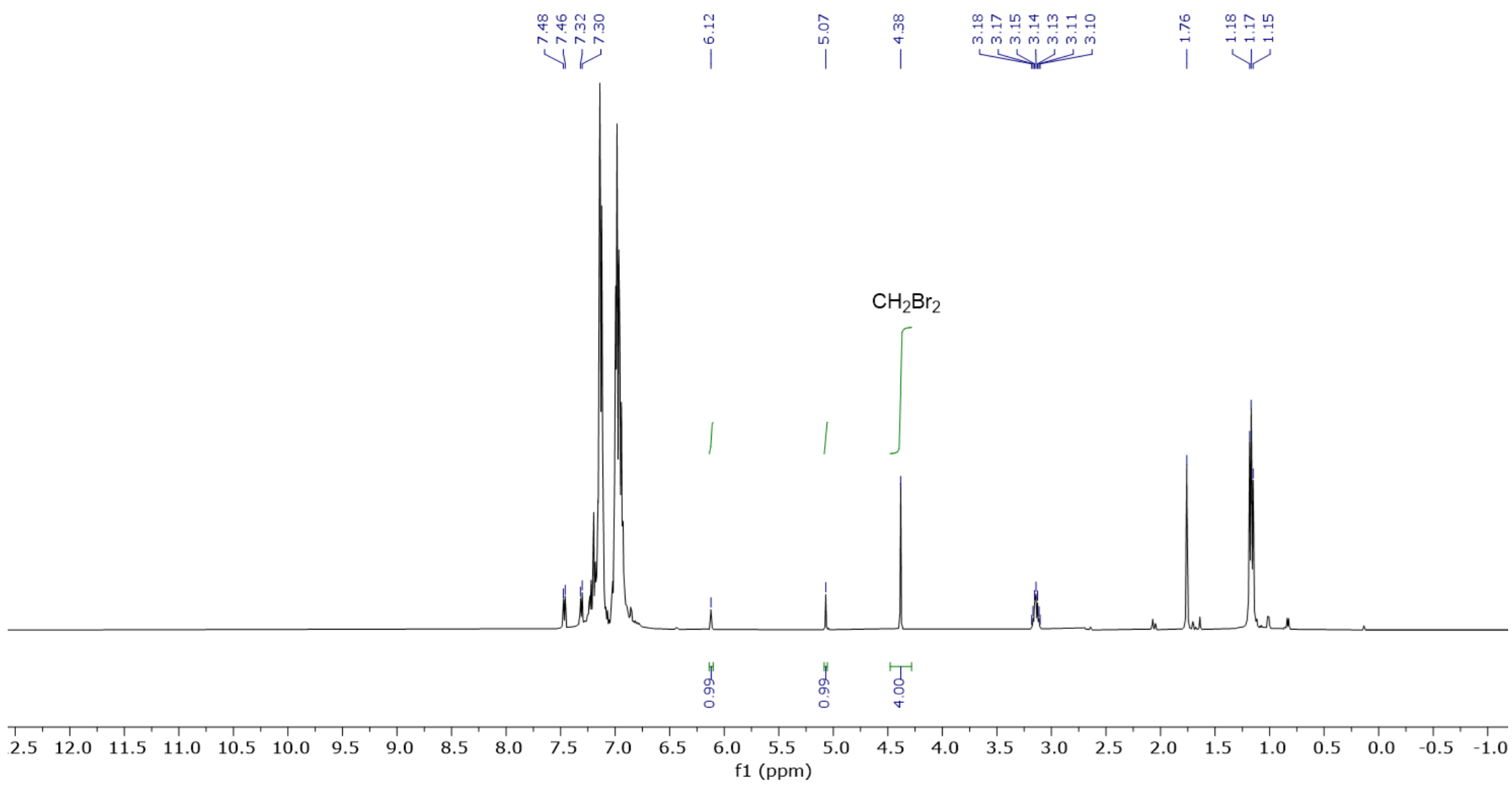

Figure S18: C–H zincation of 2-phenyl-thiophene in PhCl by *in-situ* ^{1}H NMR spectroscopy.

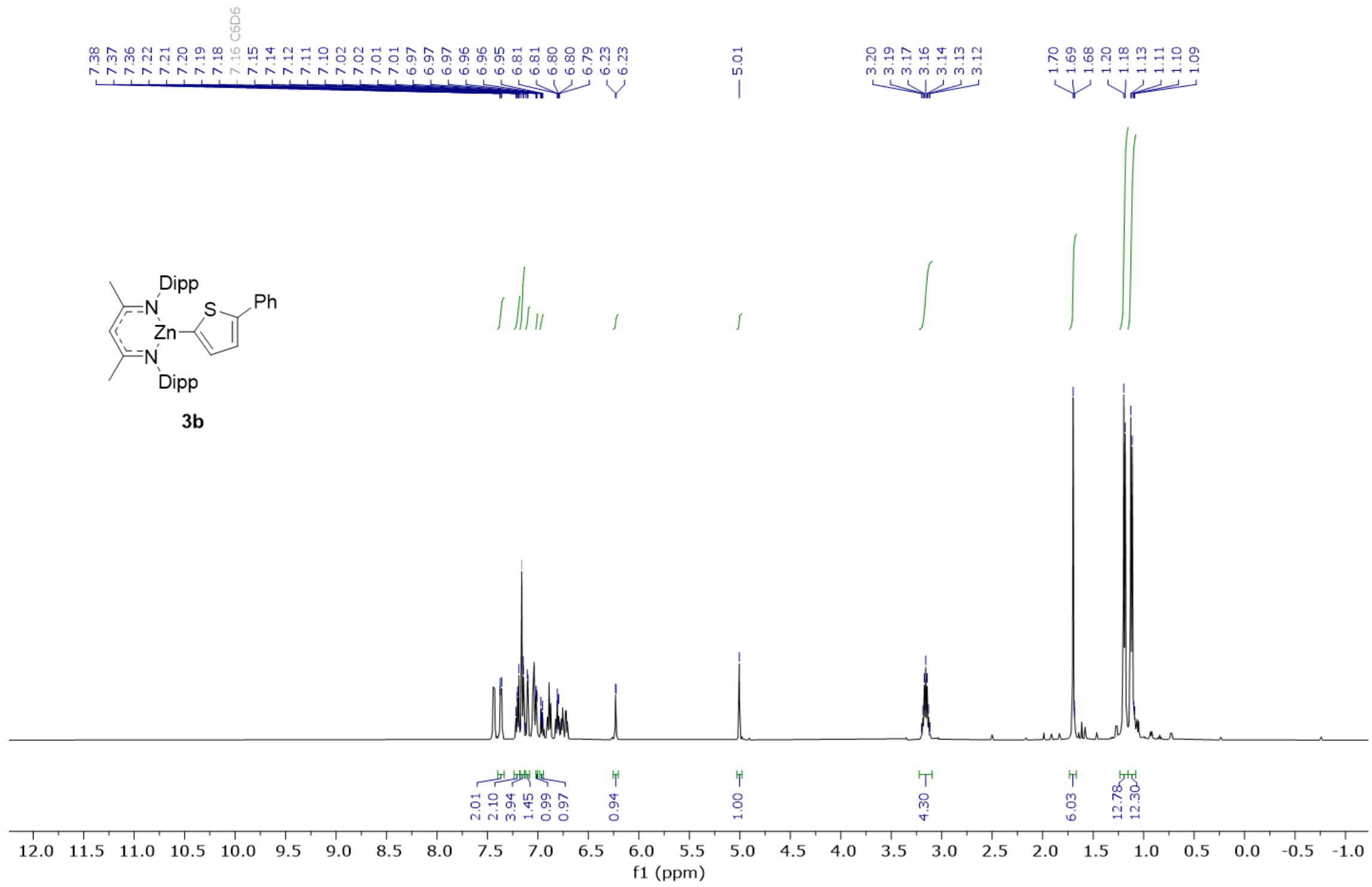

Figure S19: ^{1}H NMR spectrum of crude compound **3b** in C_6D_6.

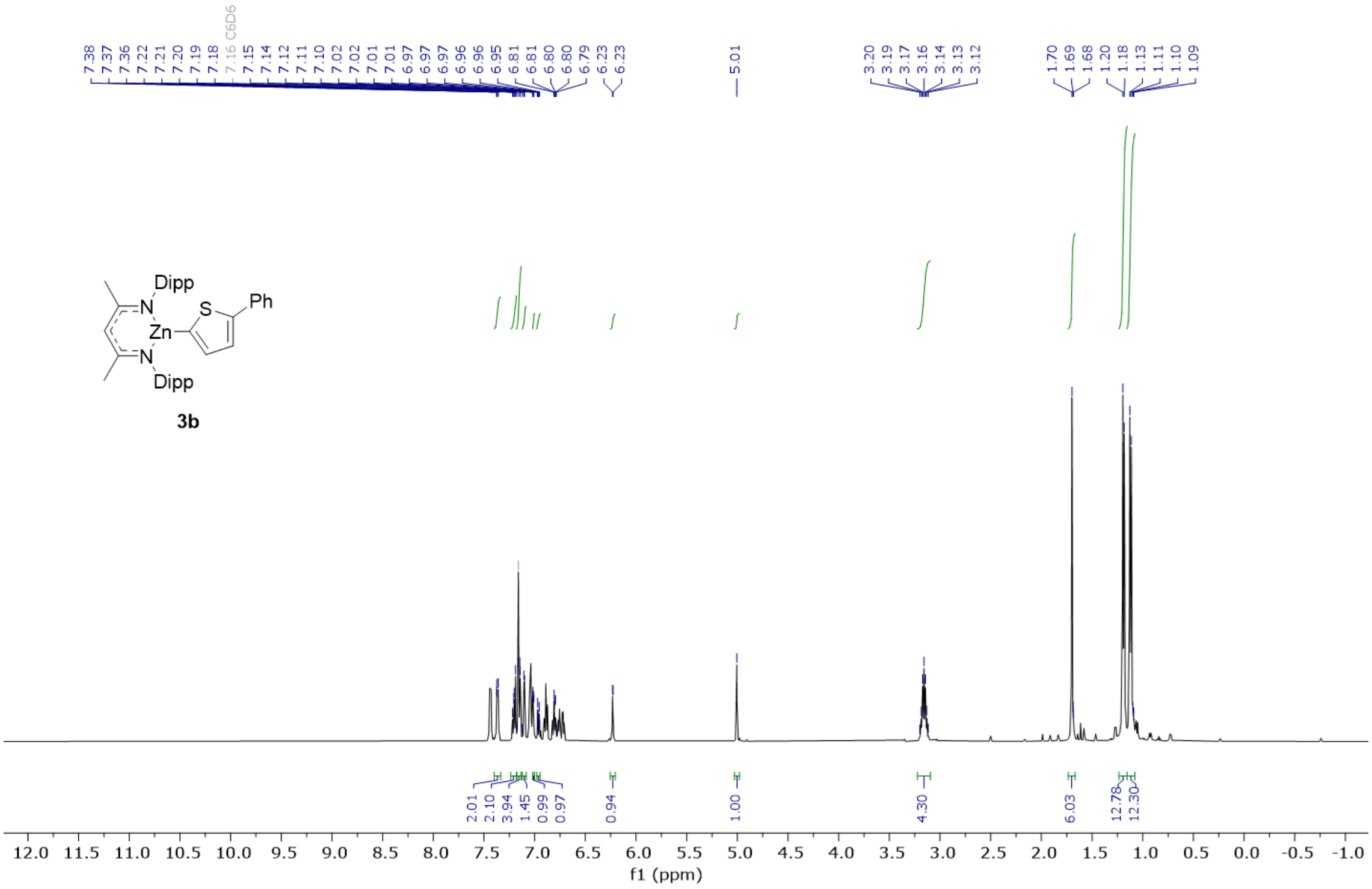

Figure S20: $^{13}C\{^{1}H\}$ NMR spectrum of crude compound **3b** in C_6D_6.

S3.4.3. Synthesis of ^{Dipp}NacNacZn-(thiophene-2-yl), **3c**

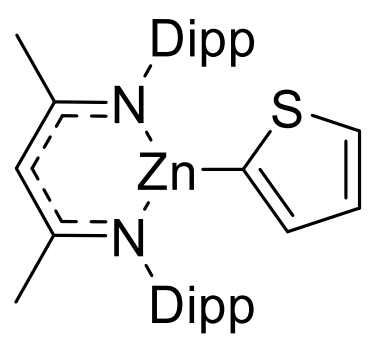

3c

As per general procedure 1, using thiophene (32 µL, 0.40 mmol, 2.0 equiv.) and heating at 110°C for 24 h. *In-situ* yield by integration of diagnostic ^{1}H resonances (96% yield by ^{1}H NMR spectroscopy in C_6D_6).

^{1}H NMR (500 MHz, C_6D_6): δ 7.23 – 7.18 (m, 4H, *Ar* & 1H, ^{Thiophene}*CH*), 6.92 (dd, *J* = 4.6 Hz, *J* = 3.2 Hz, 1H, ^{Thiophene}*CH*), 6.33 (d, *J* = 3.0 Hz, 1H, ^{Thiophene}*CH*), 5.06 (s, 1H, C_{γ}–*H*), 3.18 (sept., *J* = 6.9 Hz, 4H, *CH*(CH_3)$_2$), 1.76 (s, 6H, CH(NCCH_3)$_2$), 1.17 (t, *J* = 6.8 Hz, 24H, CH(CH_3)$_2$).

$^{13}C\{^{1}H\}$ NMR (126 MHz, C_6D_6): δ 168.5 (CH(N*C*CH$_3$)$_2$), 144.4 (*Ar*), 141.8 (*Ar*), 141.0 (*Ar*), 136.6 (*Ar*), 128.8 (*Ar*), 127.3 (*Ar*), 126.5 (*Ar*), 124.3 (*Ar*), 95.7 (C_{γ}–*H*), 28.7 (*CH*(CH$_3$)$_2$), 24.6 (CH(CH_3)$_2$), 23.7 (CH(CH_3)$_2$), 23.3 (CH(NCCH_3)$_2$).

Analytical data are consistent with that previously reported.[2]

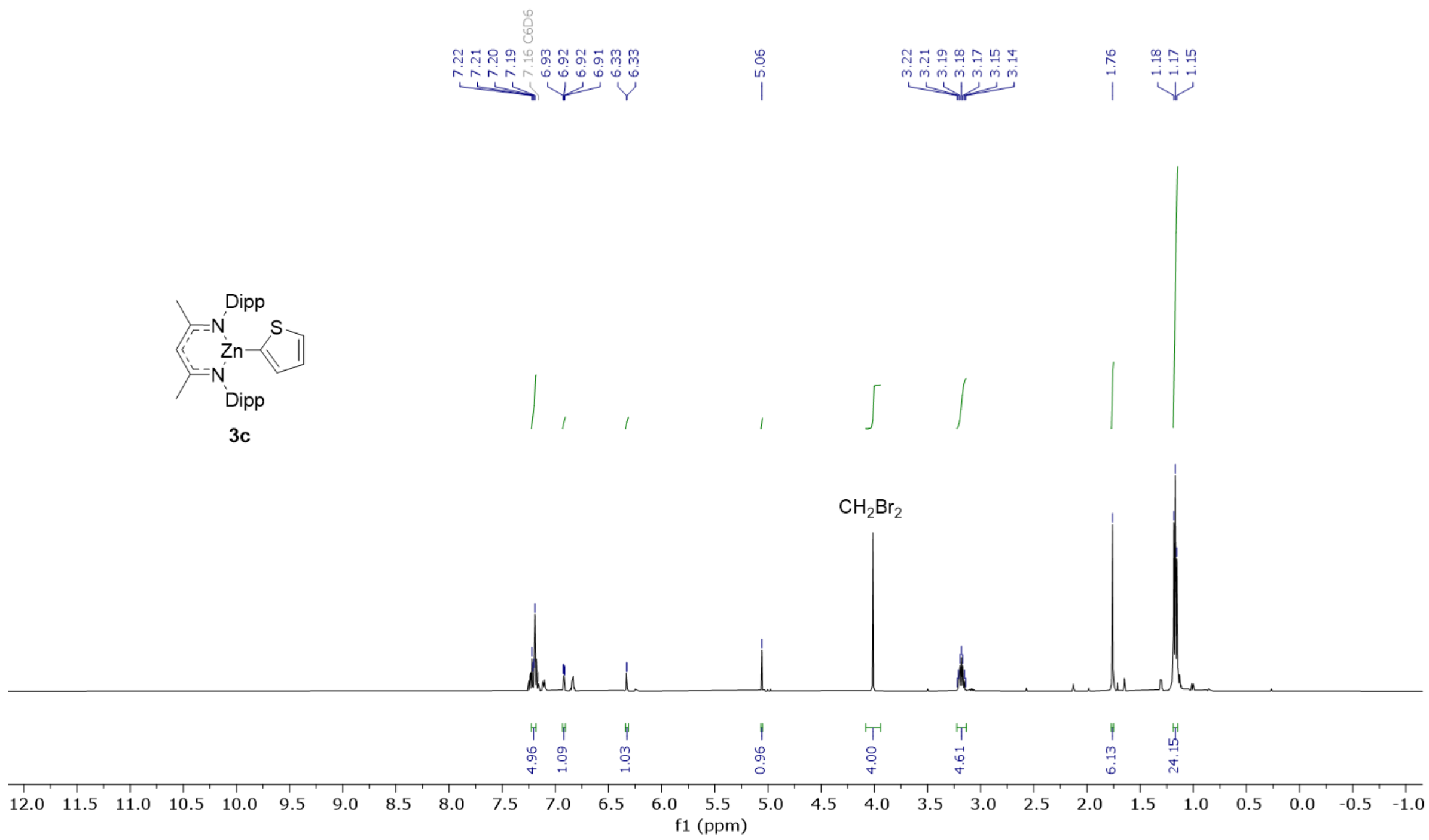

Figure S21: ^{1}H NMR spectrum of crude compound **3c** in C_6D_6 with CH_2Br_2 (28.0 µL, 0.4 mmol) added as an internal standard.

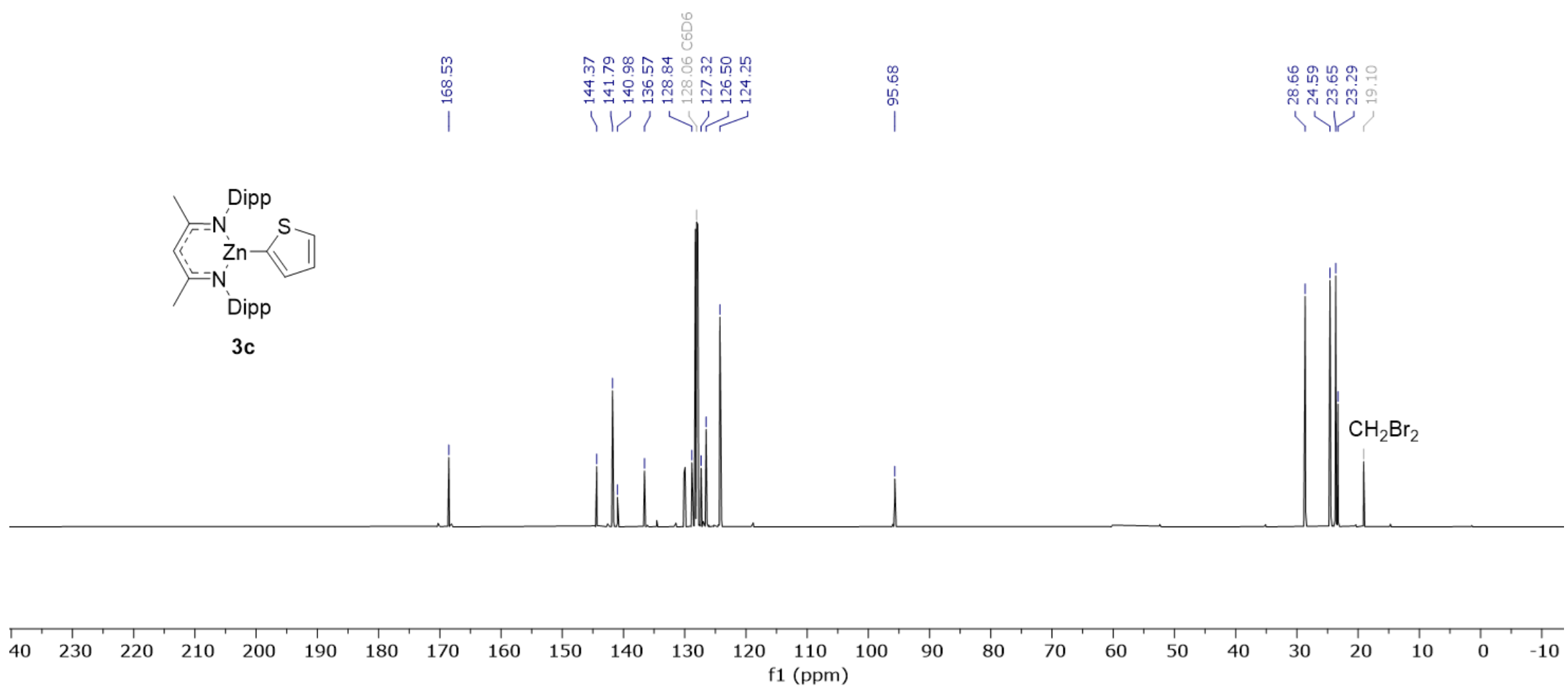

Figure S22: $^{13}C\{^{1}H\}$ NMR spectrum of crude compound **3c** in C_6D_6.

S3.4.4. Synthesis of ^{Dipp}NacNacZn-(2-benzofuranyl), **3d**

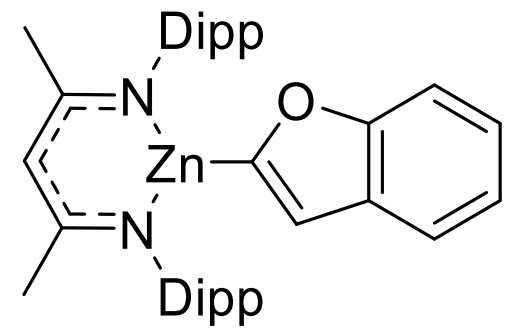

3d

As per general procedure 1, using benzofuran (45 µL, 0.40 mmol, 2.0 equiv.) and heating at 110°C for 24 h. *In-situ* yield by integration of diagnostic ^{1}H resonances (90% yield by ^{1}H NMR spectroscopy).

^{1}H NMR (500 MHz, C_6D_6): δ 7.28 (d, J = 8.0, 1H, ^{Benzfuran}C*H*), 7.22 (dd, J = 8.5 Hz, J = 6.8 Hz, 2H, *Ar*), 7.09 (dd, J = 7.6 Hz, J = 2.0 Hz, 4H, *Ar*), 6.95 (td, J = 7.9 Hz, J = 1.3 Hz, 1H, ^{Benzfuran}*CH*), 6.89 (td, J = 7.5 Hz, J = 1.1 Hz, 1H, ^{Benzfuran}*CH*), 6.10 (s, 1H, ^{Benzfuran}*CH*), 5.76 (dd, J = 7.8 Hz, J = 1.6 Hz, 1H, ^{Benzfuran}*CH*), 5.05 (s, 1H, C_γ–*H*), 3.16 (sept., J = 6.8 Hz, 4H, *CH*(CH_3)$_2$), 1.74 (s, 6H, CH(NCCH_3)$_2$), 1.13 (d, J = 7.0 Hz, 12H, CH(CH_3)$_2$), 1.07 (d, J = 6.8 Hz, 12H, CH(CH_3)$_2$).

^{13}C{^{1}H} NMR (126 MHz, C_6D_6): δ 173.3 (Zn*C*), 168.5 (CH(N*C*CH$_3$)$_2$), 159.0 (^{Benzfuran}*CH*), 155.6 (^{Benzfuran}*CH*), 151.7 (^{Benzfuran}*CH)*, 144.9 (*Ar*), 142.0 (*Ar*), 128.8 (*Ar*), 126.5 (*Ar*), 124.4 (^{Benzfuran}*CH)*, 123.8 (*Ar*), 123.2 (^{Benzfuran}*CH)*, 121.8 (^{Benzfuran}*CH)*, 110.7 (^{Benzfuran}*CH)*, 95.8 (C_γ–*H*), 28.6 (*CH*(CH_3)$_2$), 24.5 (CH(CH_3)$_2$), 23.5 (CH(CH_3)$_2$), 23.4 (CH(NCCH_3)$_2$).

Analytical data are consistent with that previously reported.[2]

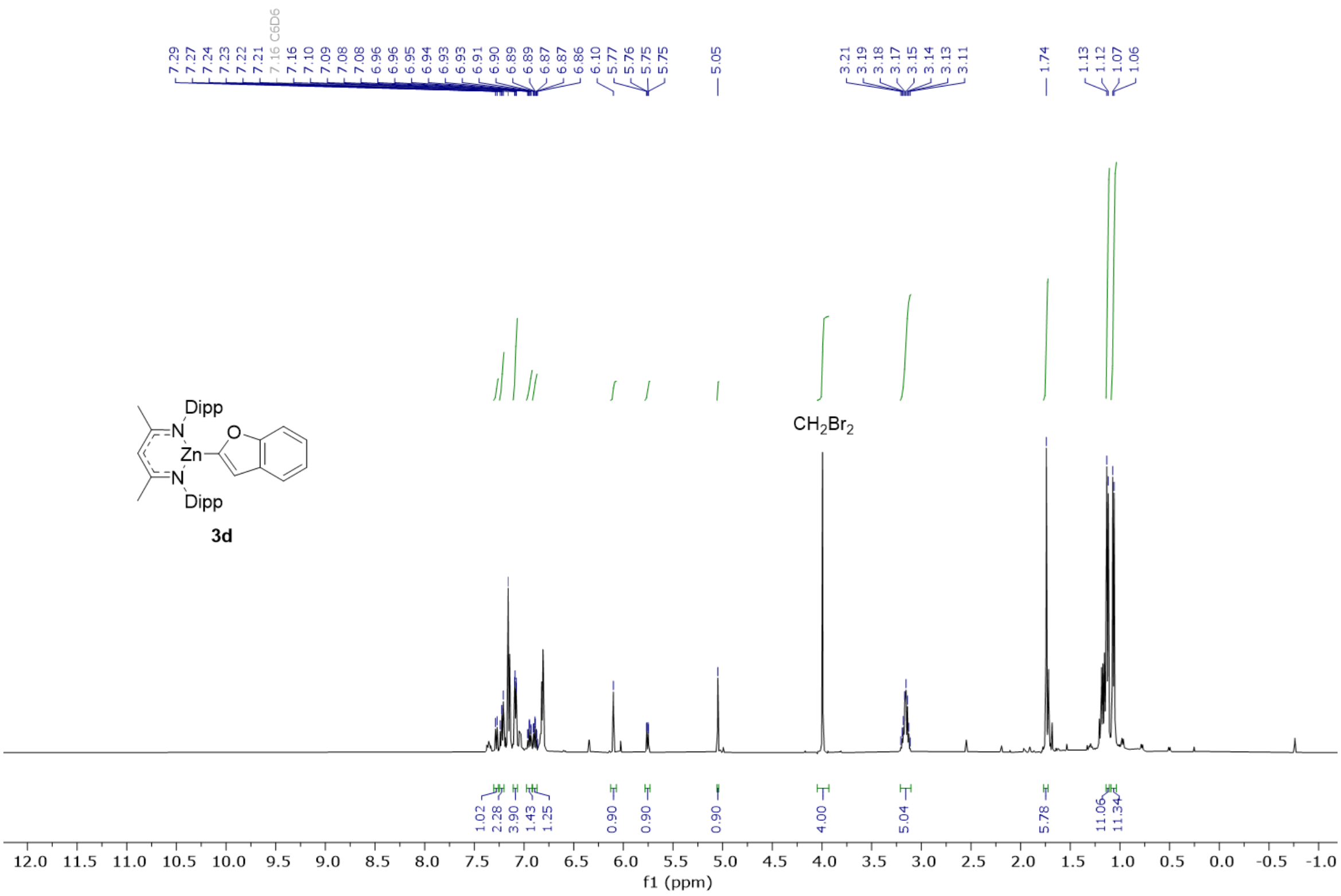

Figure S23: ^{1}H NMR spectrum of crude compound **3d** in C_6D_6 with CH_2Br_2 (28.0 µL, 0.4 mmol) added as an internal standard.

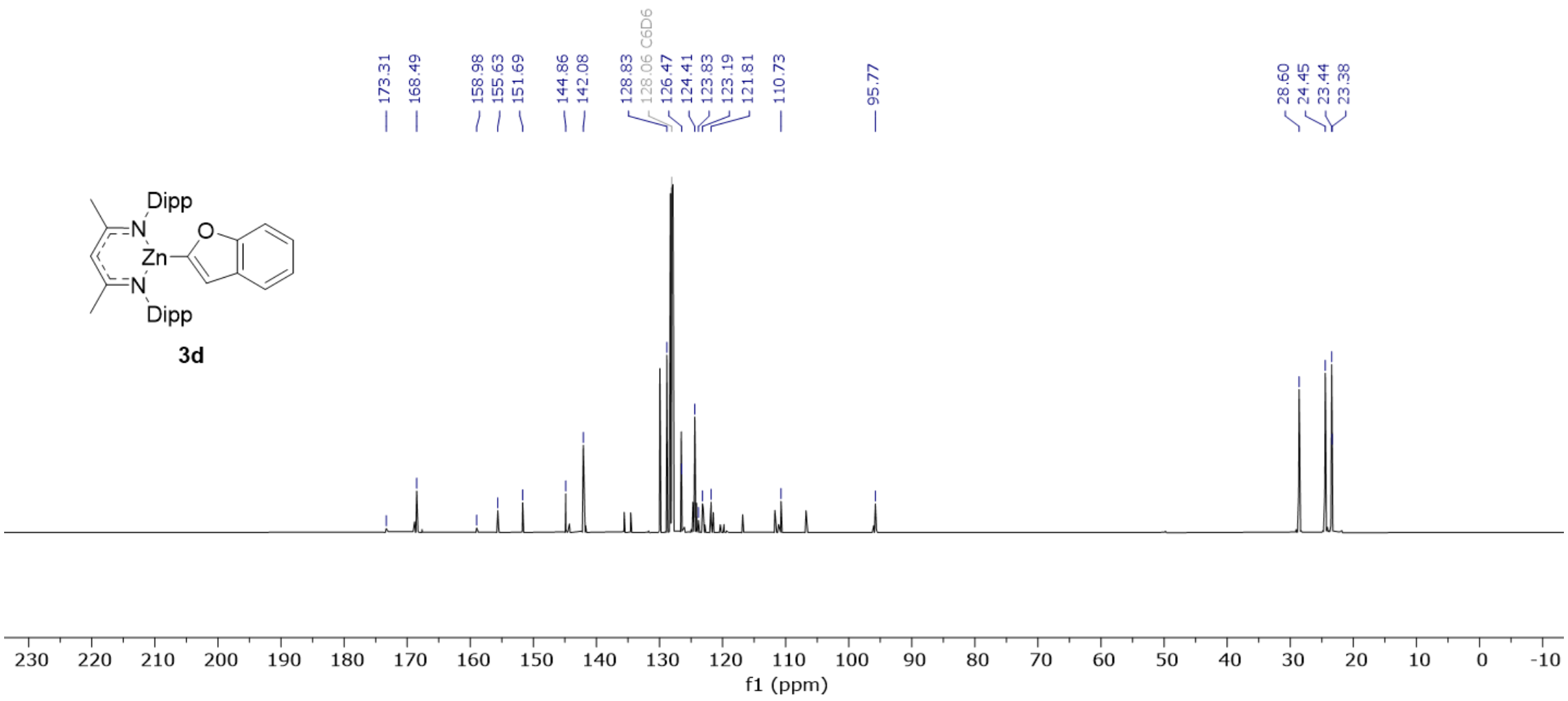

Figure S24: $^{13}C\{^{1}H\}$ NMR spectrum of crude compound **3d** in C_6D_6.

S3.5. Unsuccessful substrates for catalytic C–H zincation

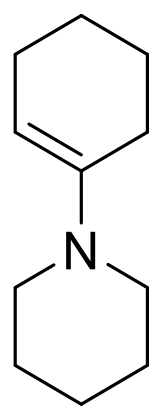

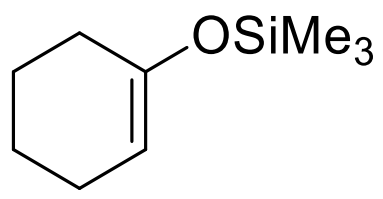

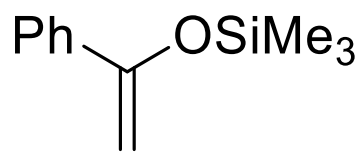

Please note that 2-ethyl-furan, 2-bromo-thiophene and 1-methyl-indole underwent catalytic transfer zincation under optimized conditions albeit in low yields (<20%). Therefore, sufficiently clean NMR spectra allowing unambiguous identification of the zincated products could not be obtained thus specific yields are not reported.

S4. Synthesis of aryl-BBN compounds

S4.1. General procedure 2: Zinc catalysed C–H mono-borylation of (hetero)arenes

In a glovebox, $(H-BBN)_2$ (30.0 mg, 0.12 mmol of dimer, 0.5 equiv.), ^{Dipp}NacNacZnMe (6.0 mg, 0.012 mmol, 5 mol%) and $[(DMT)H][B(C_6F_5)_4]$ (10 mg, 0.012 mmol, 5 mol%) charged in a J. Young’s NMR tube were dissolved in PhCl (0.6 mL). Subsequently, the corresponding heteroarene (0.28 mmol, 1.15 equiv.) was added and the reaction mixture heated at 80°C for 24 h. Upon completion, dibromomethane (17 μL, 0.24 mmol) was added to the reaction mixture as an internal standard to determine *in-situ* yield by the integration of diagnostic ^1H resonances. In cases where the diagnostic peak in ^1H NMR spectrum is obscured by chlorobenzene solvent, the reaction mixture was dried and redissolved in C_6D_6 (unless stated otherwise) to determine the *in-situ* yield upon addition of dibromomethane (17 μL, 0.24 mmol) as an internal standard.

To identify the product, the reaction mixture was dried and the residue was redissolved in C_6D_6 (unless stated otherwise) to obtain NMR spectrum from the crude reaction mixture.

Please note, formation of black solids and $O(BBN)_2$ in minor amounts was observed during the catalysis.

S4.1.1. Synthesis of 2-(9-borabicyclo[3.3.1]nonan-9-yl)-5-methyl-thiophene, **4a**

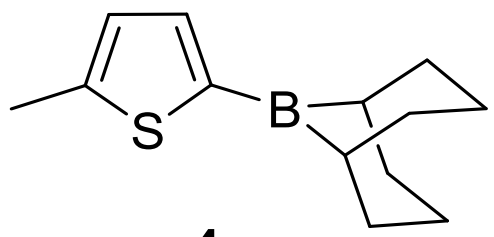

4a

As per general procedure 2, using 2-methyl-thiophene (28 µL, 0.29 mmol, 1.18 equiv.) and heating at 80°C for 24 h. *In-situ* yield by integration of diagnostic ^{1}H resonances (98% yield by ^{1}H NMR spectroscopy).

^{1}H NMR (500 MHz, PhCl): δ 7.60 (d, J =3.5 Hz, 1H, $^{Thienyl}CH$), 6.82 (d, J = 3.6 Hz, 1H, $^{Thienyl}CH$), 2.36 (s, 3H, $^{Thienyl}CH_3$), 2.11 – 2.05 (m, 2H, BBN), 1.97 – 1.91 (m, 6H, BBN), 1.86 – 1.77 (m, 6H, BBN).

^{11}B NMR (160 MHz, PhCl): δ 72.2.

Analytical data are consistent with that previously reported.[9]

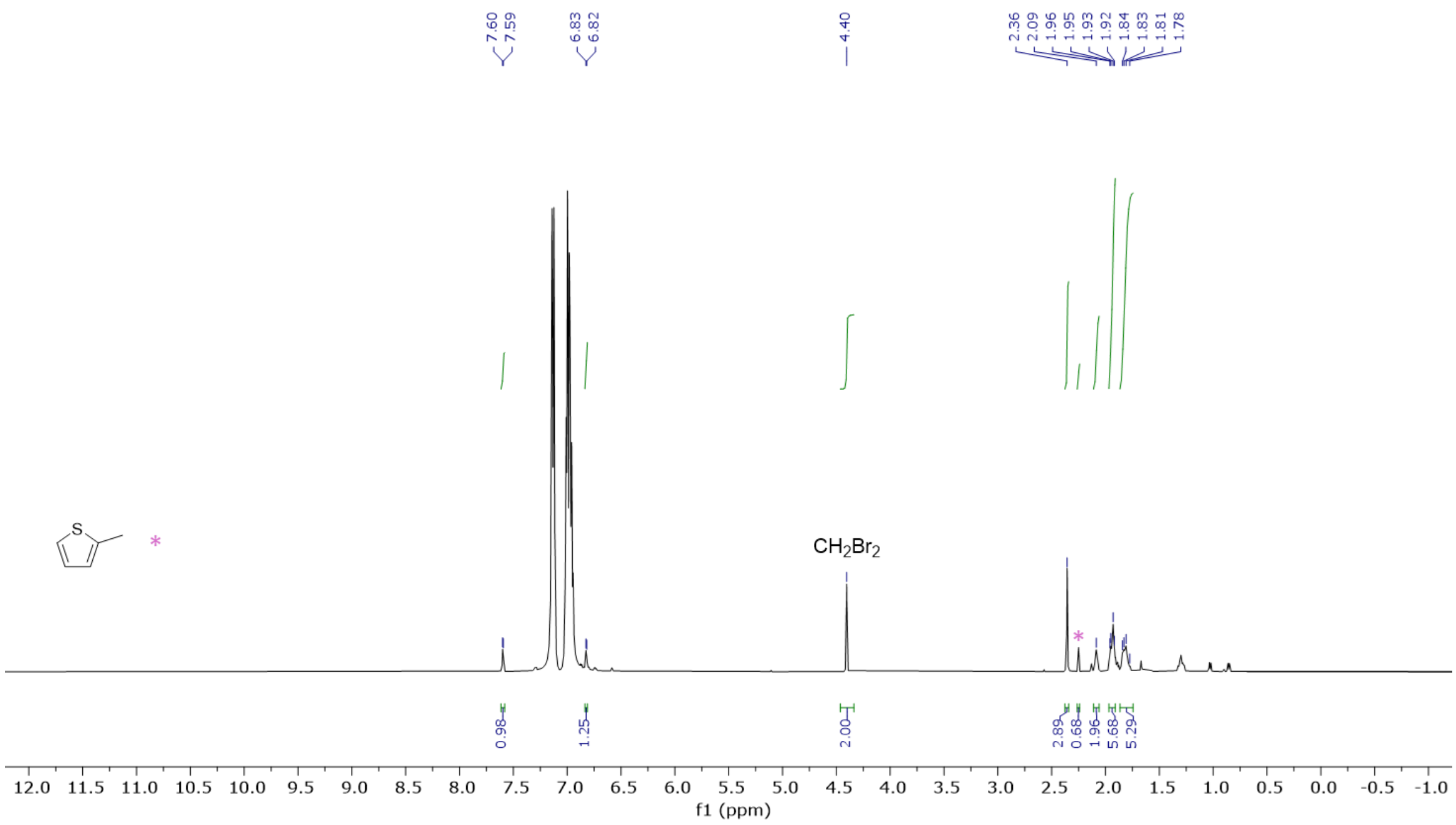

Figure S25: C–H borylation of 2-methyl-thiophene in PhCl by *in-situ* ^{1}H NMR spectroscopy.

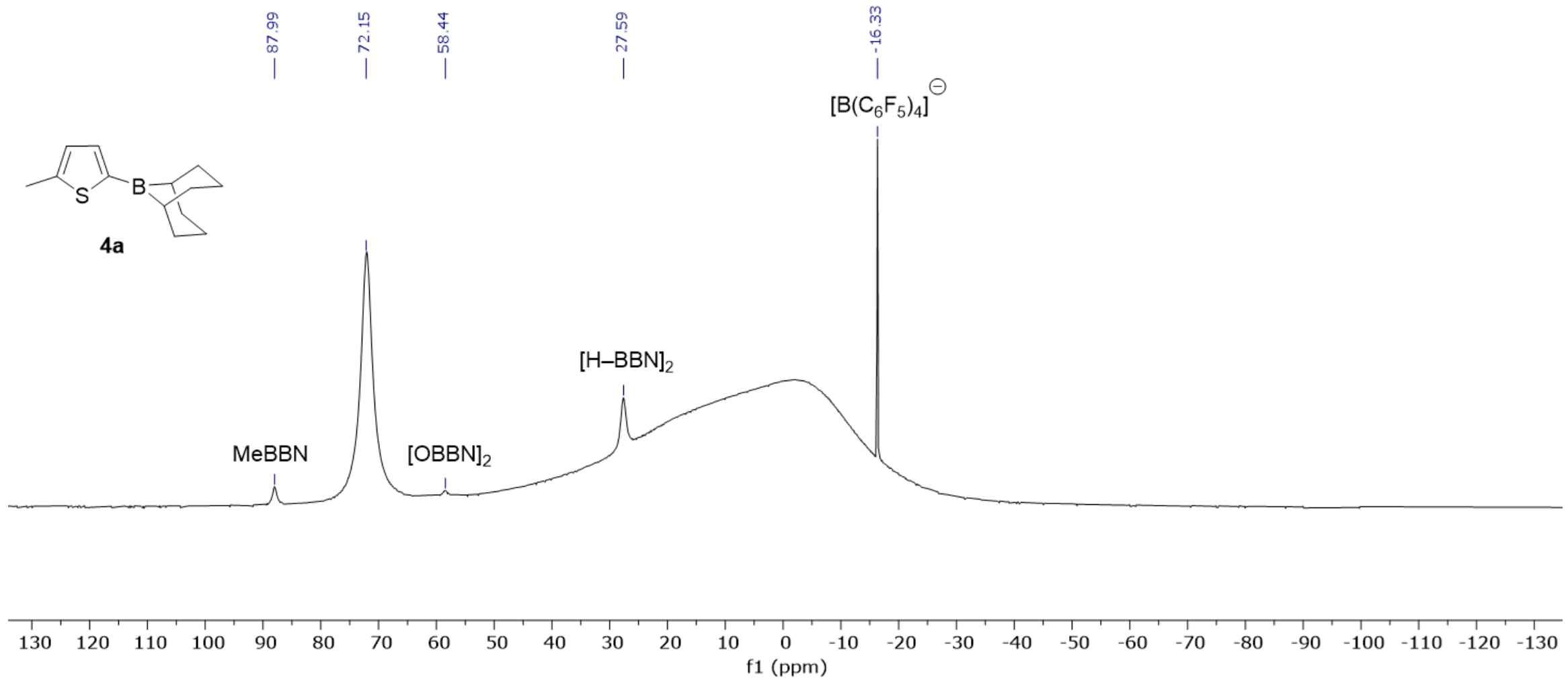

Figure S26: ^{11}B NMR spectrum of the crude reaction mixture in PhCl.

S4.1.2. Synthesis of 2-(9-borabicyclo[3.3.1]nonan-9-yl)-5-bromo-thiophene, **4e**

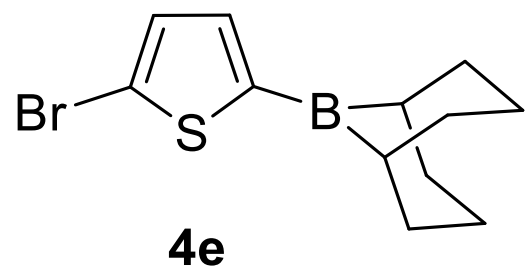

4e

As per general procedure 2, using 2-bromo-thiophene (28.0 µL, 0.29 mmol, 1.15 equiv.) and heating at 80°C for 24 h. *In-situ* yield by integration of diagnostic ^{1}H resonances (81% yield by ^{1}H NMR spectroscopy).

^{1}H NMR (500 MHz, C_6D_6): δ 7.24 (d, J = 3.7 Hz, 1H, ^{Thienyl}*CH*), 6.92 (d, J = 3.7 Hz, 1H, ^{Thienyl}*CH*), 2.01 – 1.97 (m, 2H, BBN), 1.94 – 1.85 (m, 8H, BBN), 1.78 – 1.71 (m, 4H, BBN).

^{13}C{^{1}H} NMR (126 MHz, C_6D_6): δ 139.0, 133.1, 125.1, 34.4, 30.1, 23.8.

^{11}B NMR (160 MHz, C_6D_6): δ 73.0.

Analytical data are consistent with that previously reported.[9]

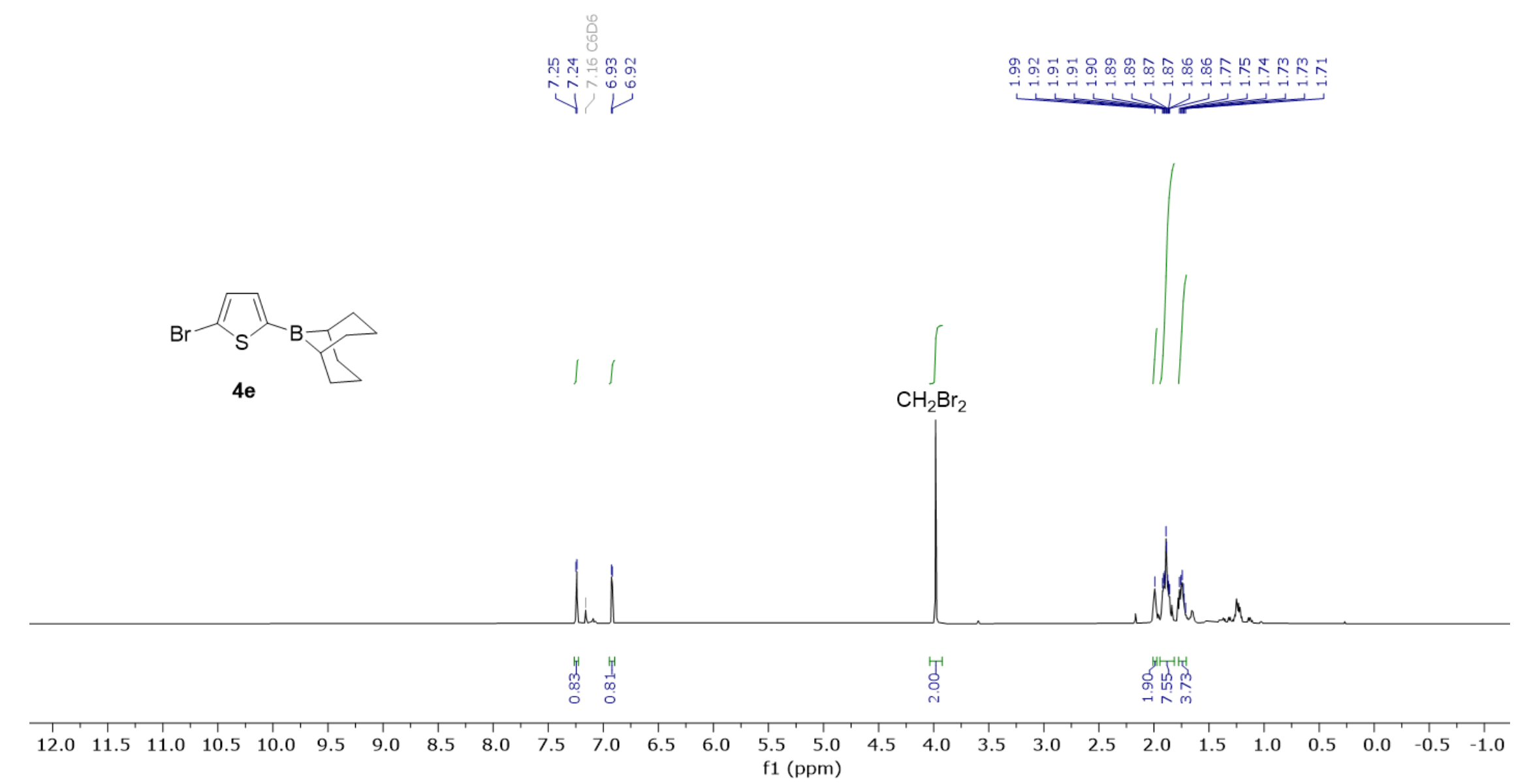

Figure S27: ^{1}H NMR spectrum of crude compound **4e** in C_6D_6 with CH_2Br_2 (17.0 µL, 0.24 mmol) added as an internal standard.

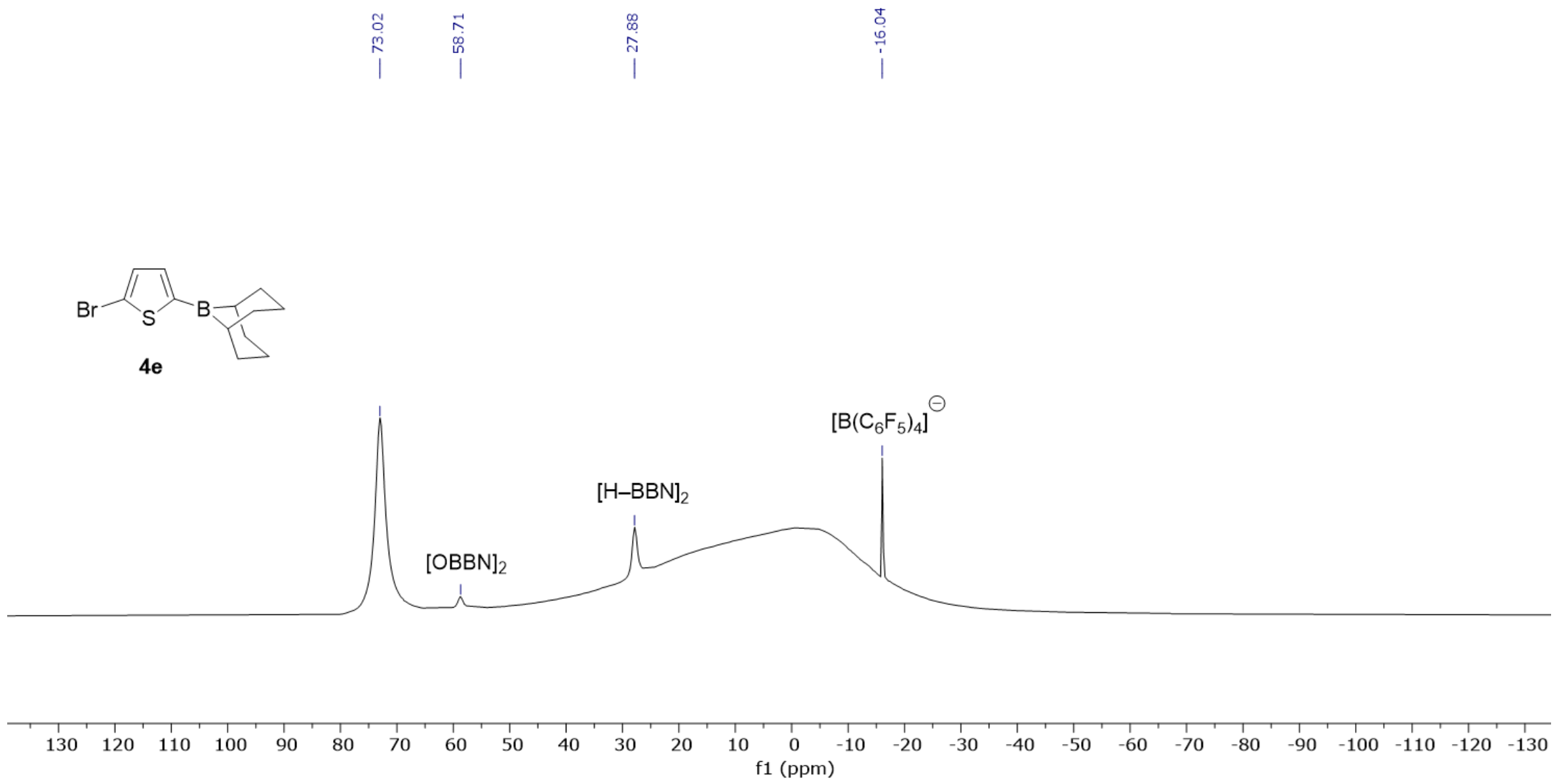

Figure S28: ^{11}B NMR spectrum of crude compound **4e** in C_6D_6.

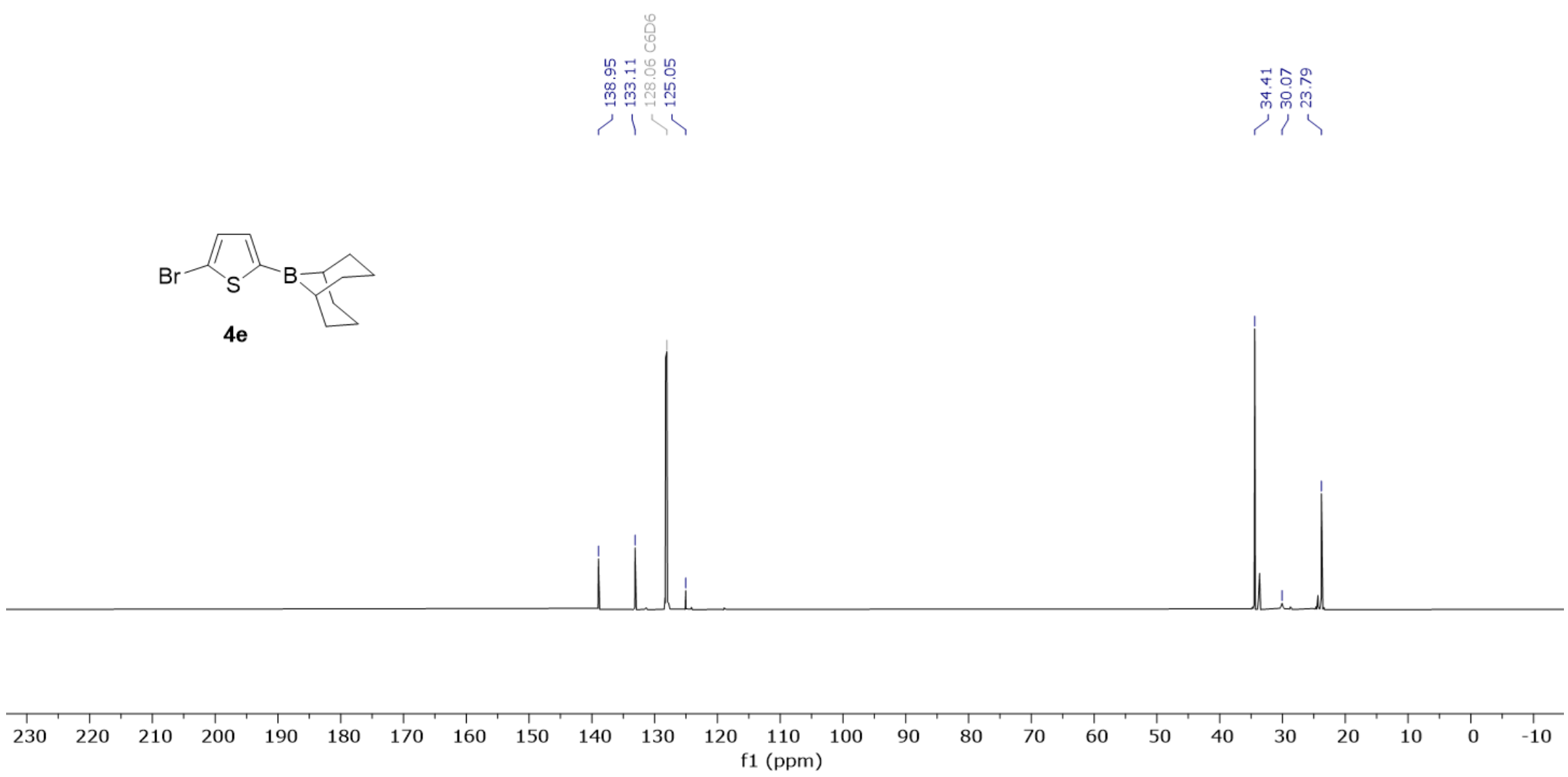

Figure S29: $^{13}C\{^{1}H\}$ NMR spectrum of crude compound **4e** in C_6D_6.

S4.1.3. Synthesis of 2-(9-borabicyclo[3.3.1]nonan-9-yl)-furan, **4f**

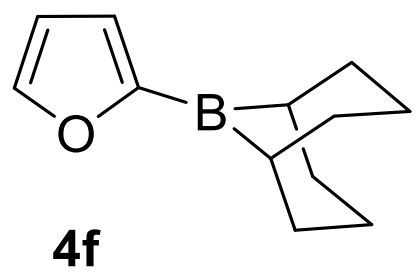

4f

As per general procedure 2, using furan (21.0 µL, 0.29 mmol, 1.15 equiv.) and heating at 80°C for 24 h. *In-situ* yield by integration of diagnostic ^{1}H resonances (62% yield by ^{1}H NMR spectroscopy).

^{1}H NMR (500 MHz, C_6D_6): δ 7.42 (d, J = 1.0 Hz, 1H, ^{Furanyl}*CH*), 7.20 (d, J = 3.2 Hz, 1H, ^{Furanyl}*CH*), 6.19 (dd, J = 3.1 Hz, J = 1.8 Hz, 1H, ^{Furanyl}*CH*), 2.30 (br. s, 2H, BBN), 1.98 – 1.93 (m, 6H, BBN), 1.90 – 1.86 (m, 6H, BBN).

^{13}C{^{1}H} NMR (126 MHz, C_6D_6): δ 150.3, 127.4, 111.8, 34.3, 28.3, 23.8.

^{11}B NMR (160 MHz, C_6D_6): δ 71.1.

Mass Spectrometry: Calculated [M(Cl)$^{-}$] = 223.10610, Observed [M(Cl)$^{-}$] = 223.10630.

Analytical data are consistent with that previously reported.[9]

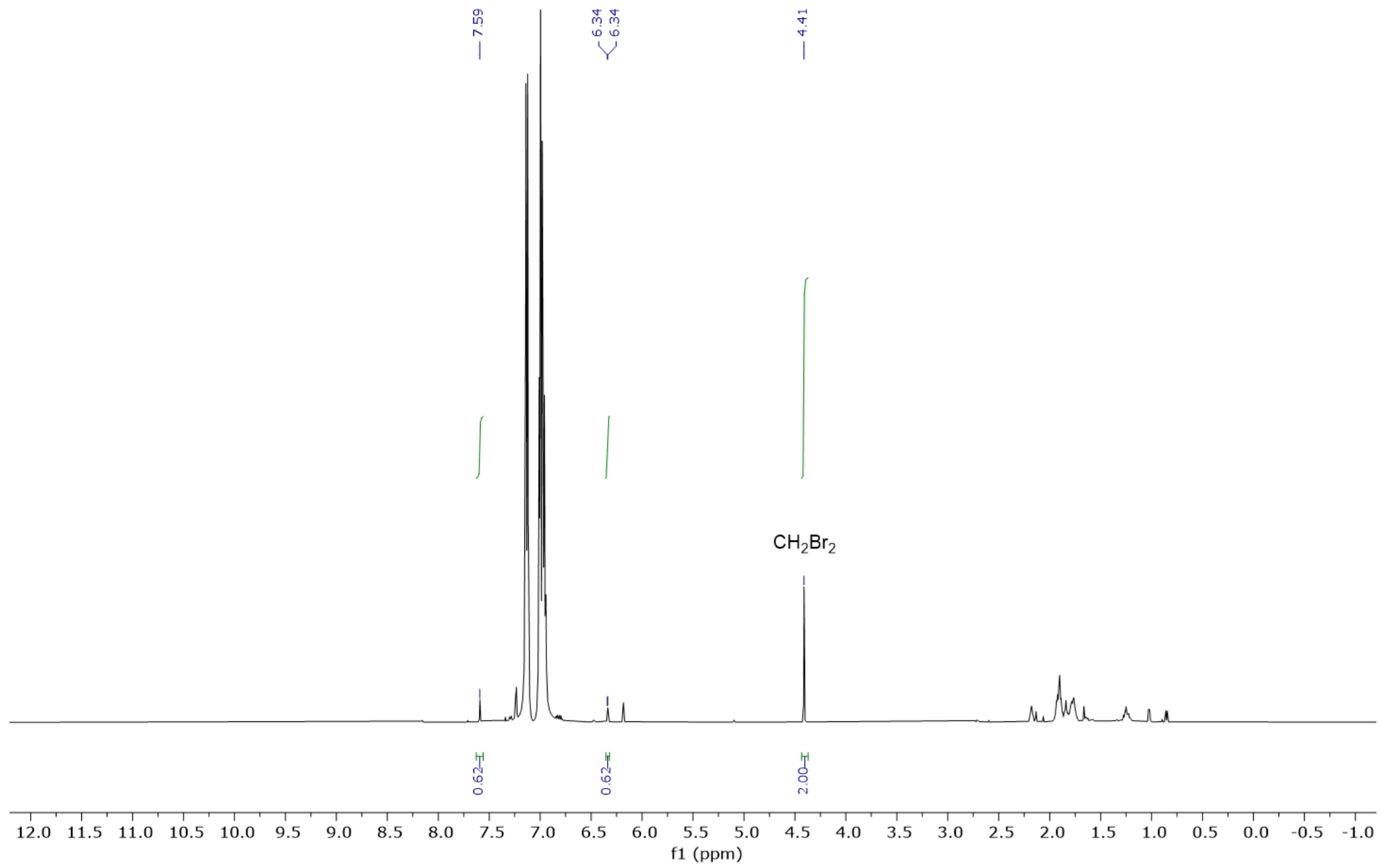

Figure S30: C–H borylation of furan in PhCl by *in-situ* ^{1}H NMR spectroscopy.

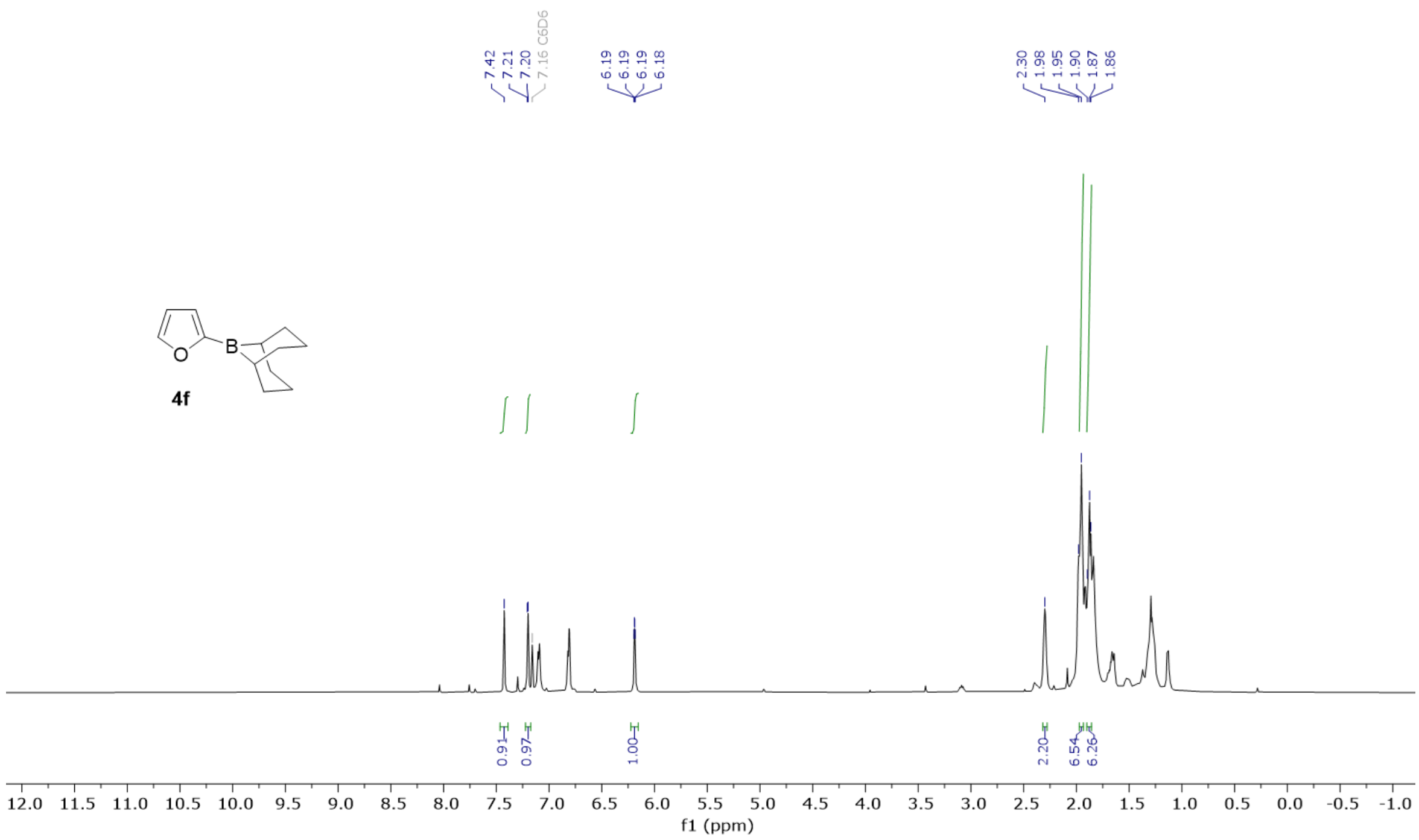

Figure S31: ^{1}H NMR spectrum of crude compound **4f** in C_6D_6.

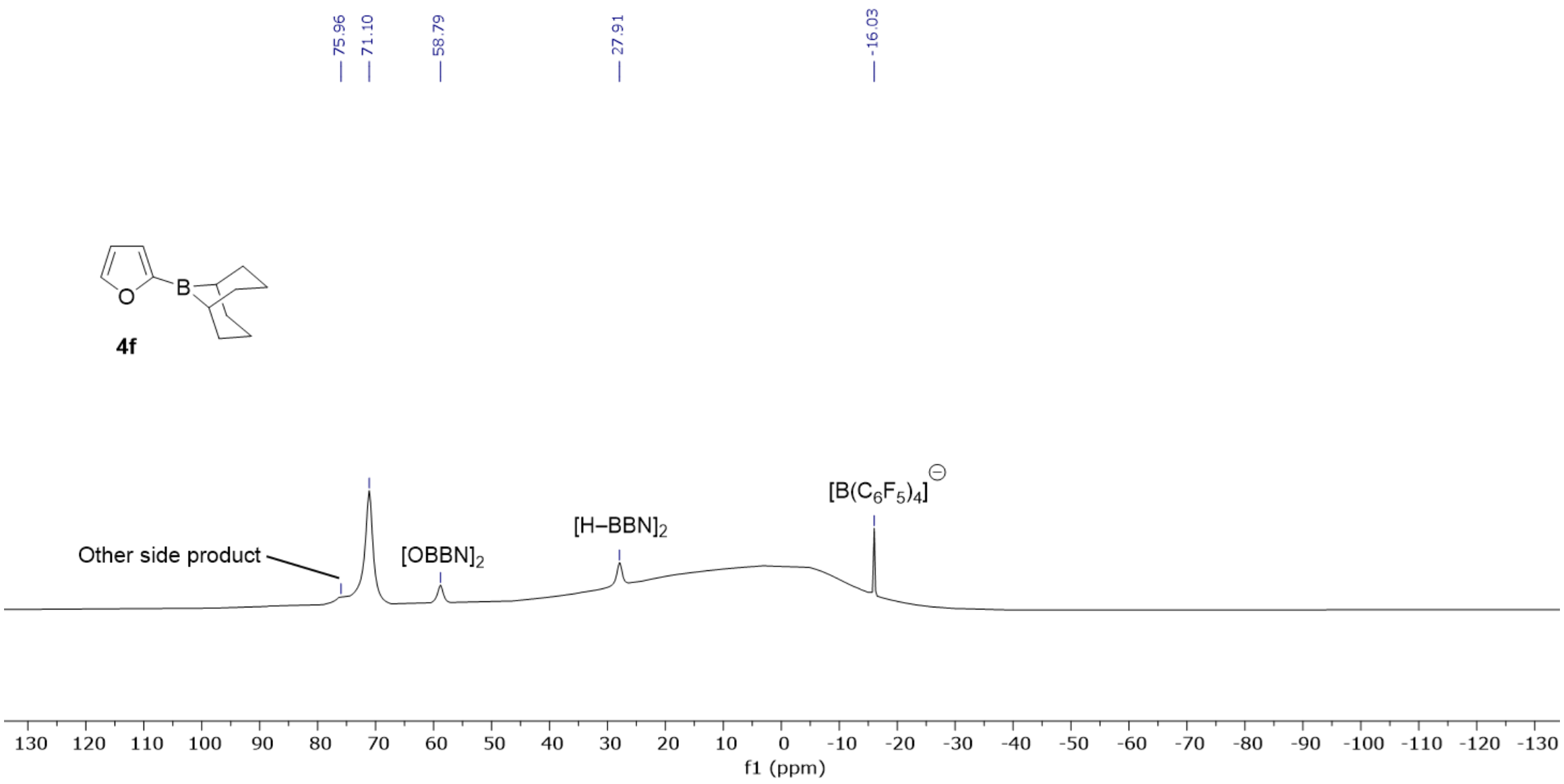

Figure S32: ^{11}B NMR spectrum of crude compound **4f** in C_6D_6.

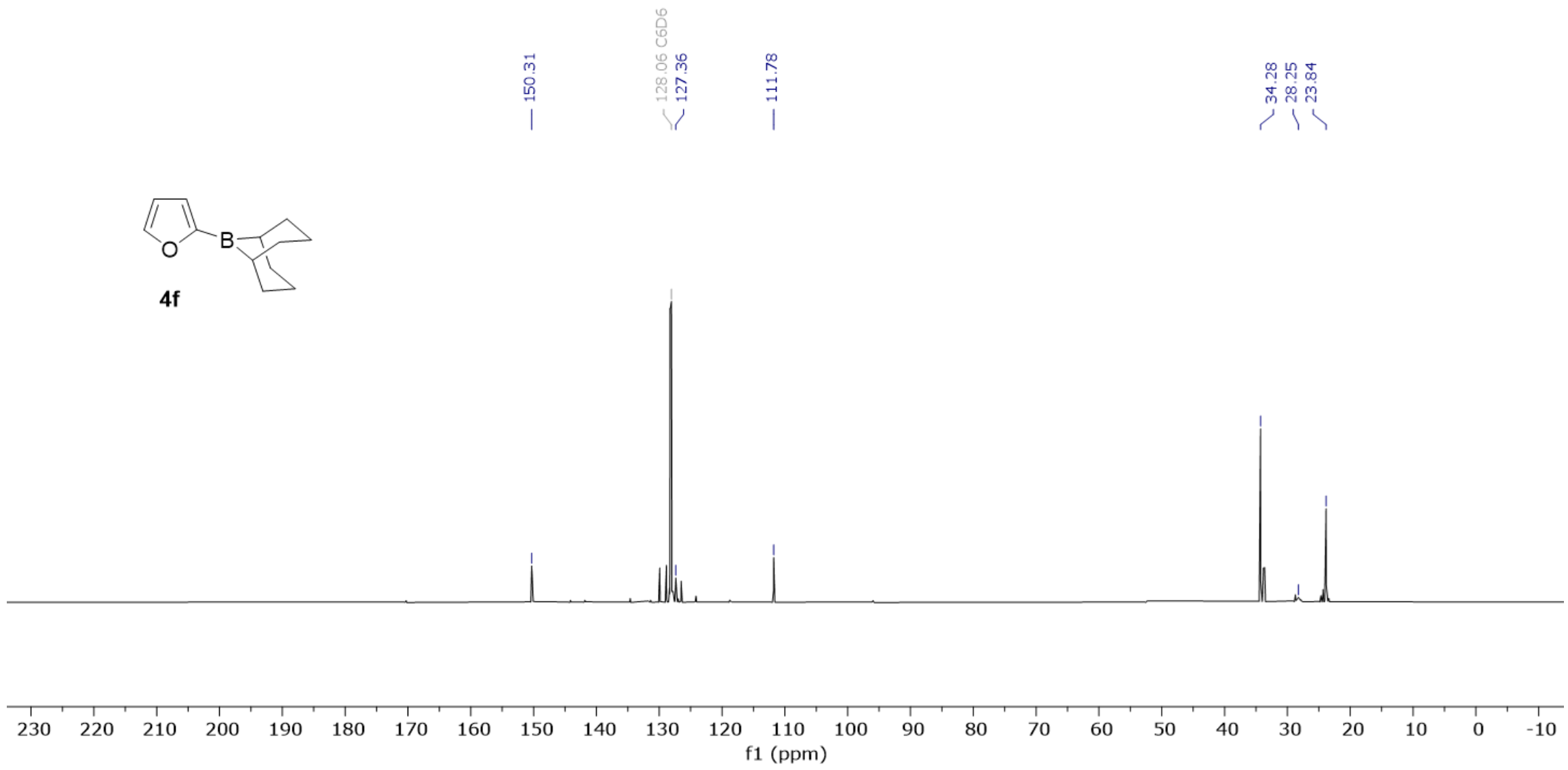

Figure S33: $^{13}C\{^1H\}$ NMR spectrum of crude compound **4f** in C_6D_6.

S4.1.4. Synthesis of 2-(9-borabicyclo[3.3.1]nonan-9-yl)-5-ethyl-furan, **4g**

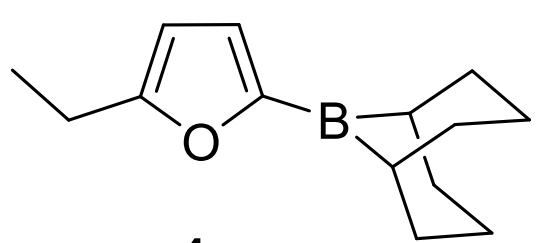

4g

As per general procedure 2, using 2-ethyl-furan (31.0 μL, 0.29 mmol, 1.15 equiv.) and heating at 80°C for 24 h. *In-situ* yield by integration of diagnostic ^{1}H resonances (76% yield by ^{1}H

NMR spectroscopy). As this compound has not been reported before, volatiles were removed in vacuo and the residue was extracted in dry *n*-hexane (ca. 2 mL). The solution was filtered, dried under vacuo and submitted for NMR characterisation in C_6D_6.

^{1}H NMR (500 MHz, C_6D_6): δ 7.28 (d, J = 3.3 Hz, 1H, ^{Furanyl}*CH*), 5.99 (d, J = 3.3 Hz, 1H, ^{Furanyl}*CH*), 2.48 (q, J = 7.6 Hz, 2H, ^{Furanyl}*CH_2*CH_3), 2.35 (br. s, 2H, BBN), 2.04 – 1.97 (m, 6H, BBN), 1.96 – 1.86 (m, 6H, BBN), 1.01 (t, J = 7.6 Hz, 3H, ^{Furanyl}CH_2*CH_3*).

^{13}C{^{1}H} NMR (126 MHz, C_6D_6): δ 166.6 (^{Furanyl}*CH*), 129.2 (^{Furanyl}*CH*), 107.4 (^{Furanyl}*CH*), 34.4 (*BBN*), 33.8 (*BBN*), 27.9 (*BBN*), 23.9 (*BBN*), 23.6 (*BBN*), 22.2 (^{Furanyl}*CH_2*CH_3), 12.0 (^{Furanyl}CH_2*CH_3*).

^{11}B NMR (160 MHz, C_6D_6): δ 69.8.

Mass Spectrometry: Calculated [M(Na)$^+$] = 239.15832, Observed [M(Na)$^+$] = 239.15740.

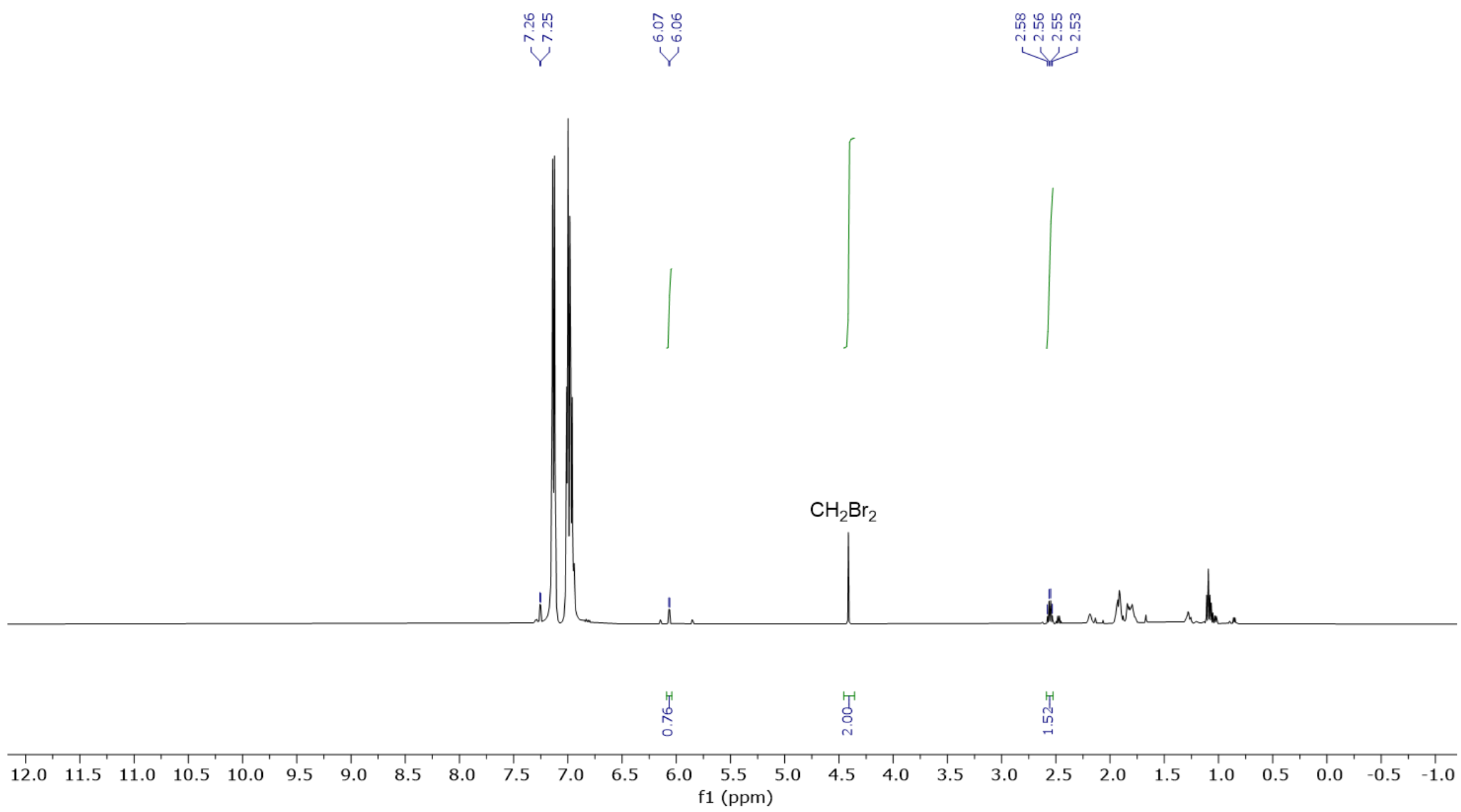

Figure S34: C–H borylation of 2-ethyl-furan in PhCl by *in-situ* ^{1}H NMR spectroscopy.

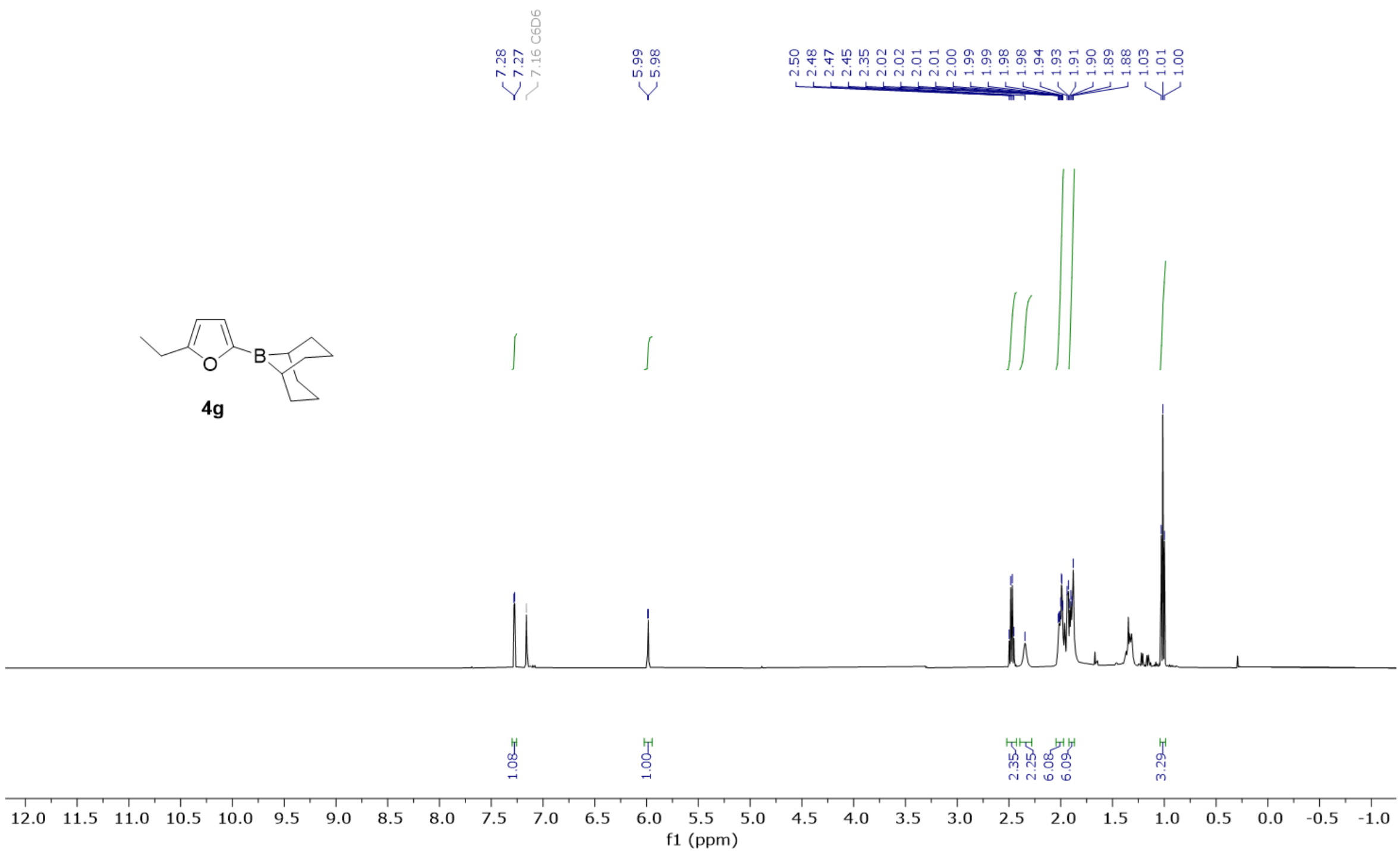

Figure S35: ^{1}H NMR spectrum of compound **4g** in C_6D_6.

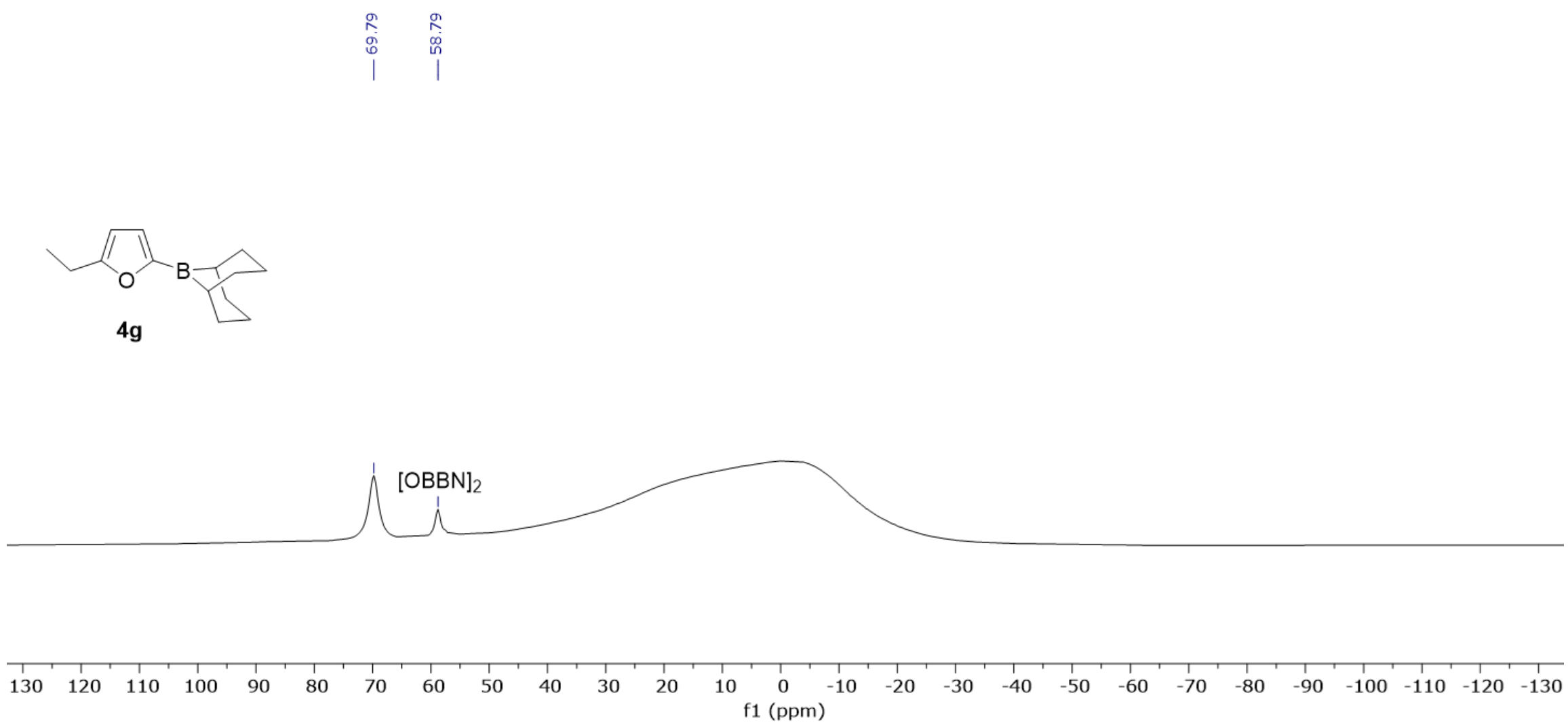

Figure S36: ^{11}B NMR spectrum of compound **4g** in C_6D_6.

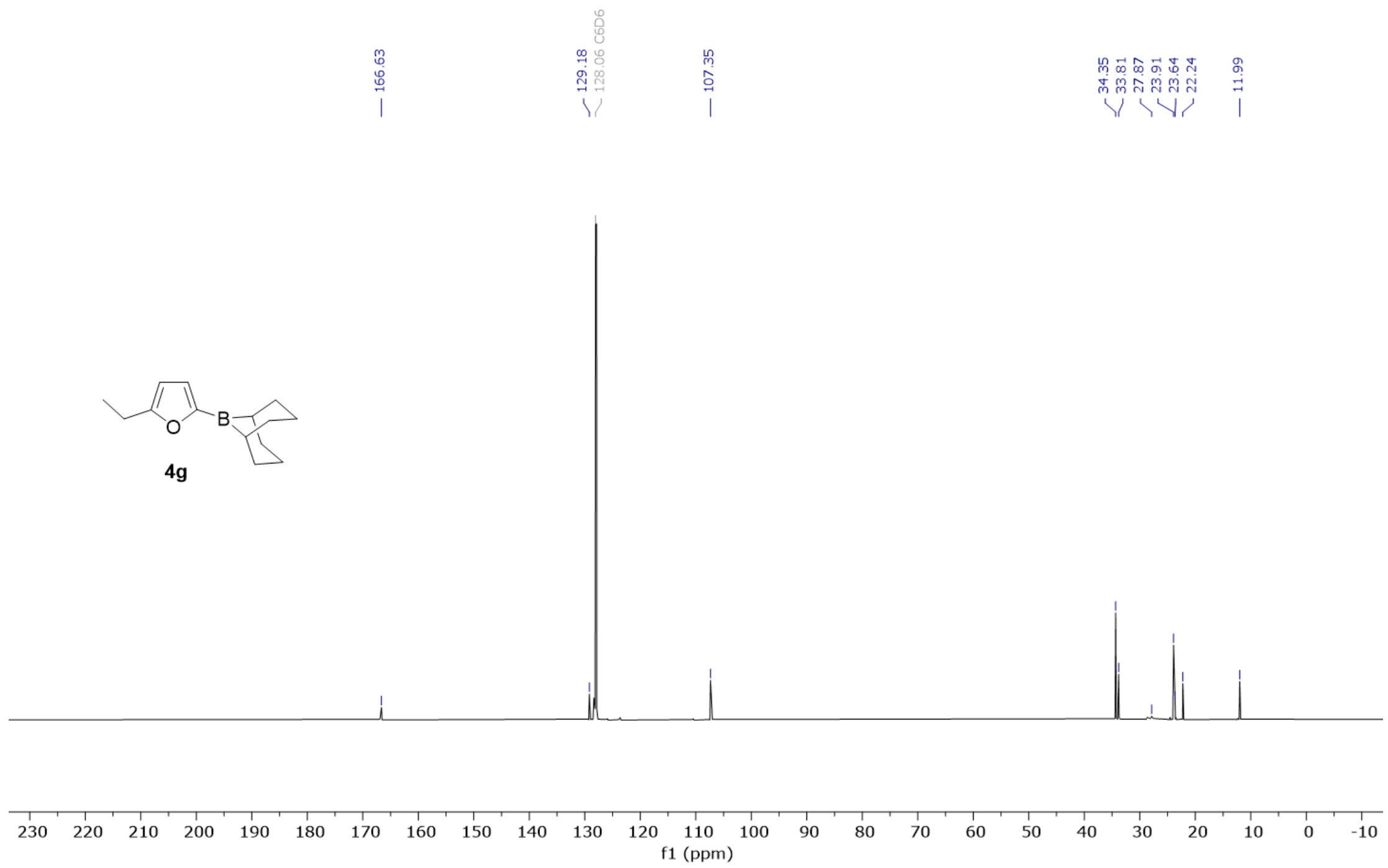

Figure S37: $^{13}C\{^{1}H\}$ NMR spectrum of compound **4g** in C_6D_6.

S4.1.5. Synthesis of 3-(9-borabicyclo[3.3.1]nonan-9-yl)-1-methyl-indole, **4h**

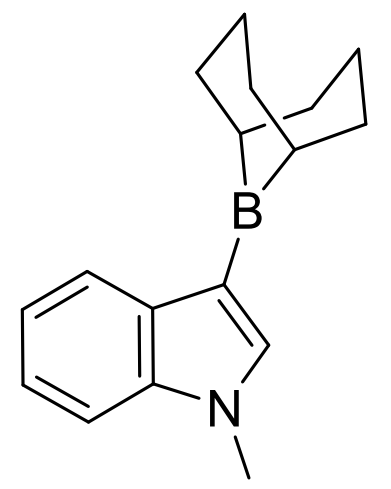

4h

As per general procedure 2, using 1-methyl-indole (36.0 µL, 0.29 mmol, 1.15 equiv.) and heating at 80°C for 24 h. *In-situ* yield by integration of diagnostic ^{1}H resonances in CH_2Cl_2 (55% yield by ^{1}H NMR spectroscopy). Please note formation of 1-methylindoline was also observed albeit in low yield (18% yield by ^{1}H NMR spectroscopy).[10]

^{1}H NMR (500 MHz, CD_2Cl_2): δ 8.09 (d, J = 7.1 Hz, 1H, ^{Indole}*CH*), 7.78 (s, 1H, ^{Indole}*CH*), 7.41 (d, J = 8.2 Hz, 1H, ^{Indole}*CH*), 7.32 – 7.26 (m, 1H, ^{Indole}*CH*), 7.26 – 7.21 (m, 1H, ^{Indole}*CH*), 3.85 (s, 3H, N–CH_3), 2.40 (br. s, 2H, BBN), 2.13 – 2.00 (m, 6H, BBN), 2.00 – 1.89 (m, 4H, BBN), 1.41 (s, 2H, BBN).

$^{13}C\{^1H\}$ NMR (126 MHz, CD_2Cl_2): δ 141.8, 139.9, 133.6, 123.0, 122.3, 121.3, 110.1, 34.4, 33.9, 29.5 (br. s), 24.1.

^{11}B NMR (160 MHz, CD_2Cl_2): δ 72.6.

Analytical data are consistent with that previously reported.[11]

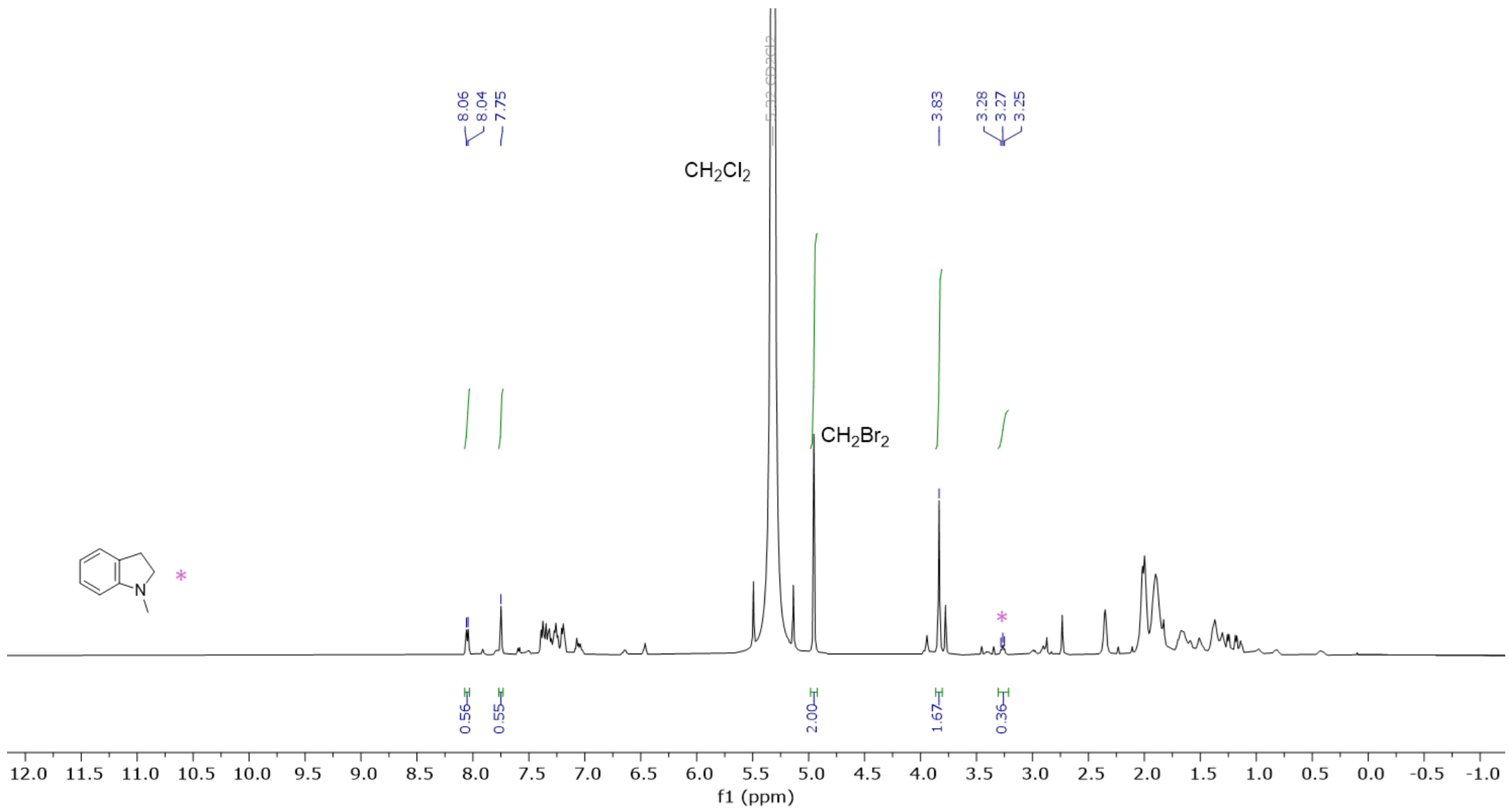

Figure S38: C–H borylation of 1-methyl-indole in CH_2Cl_2 by *in-situ* ^{1}H NMR spectroscopy.

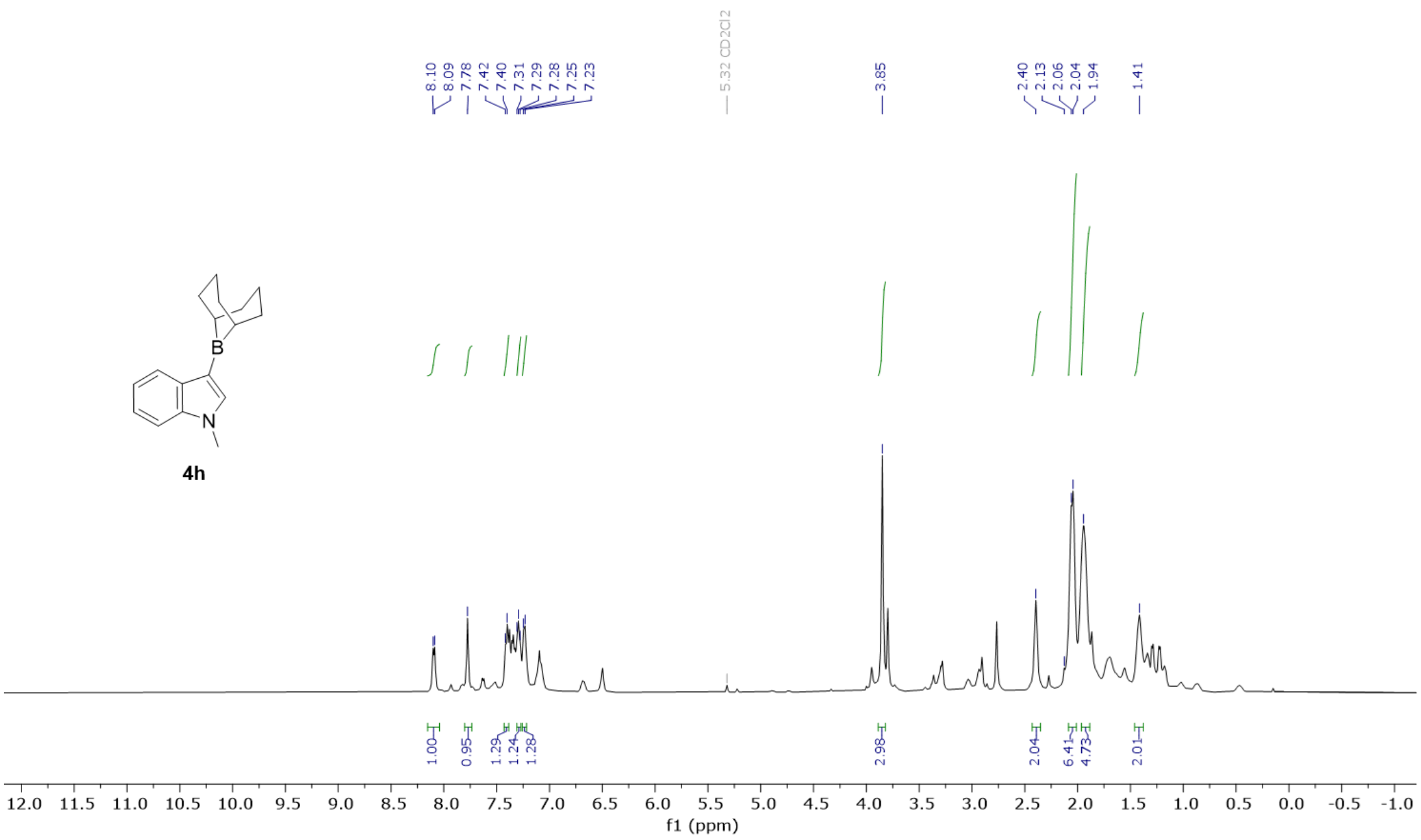

Figure S39: ^{1}H NMR spectrum of crude compound **4h** in CD_2Cl_2.

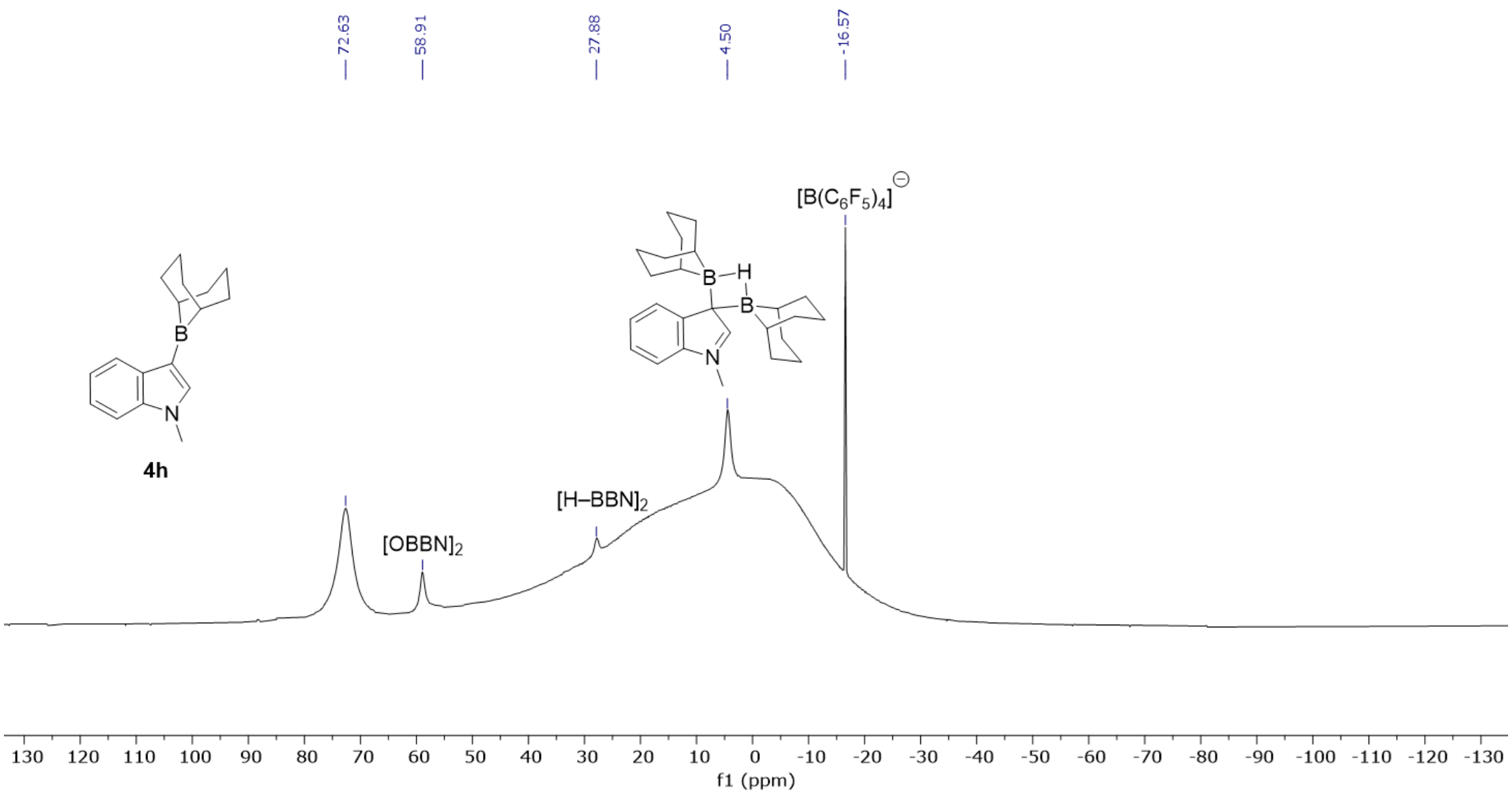

Figure S40: ^{11}B NMR spectrum of crude compound **4h** in CD_2Cl_2.

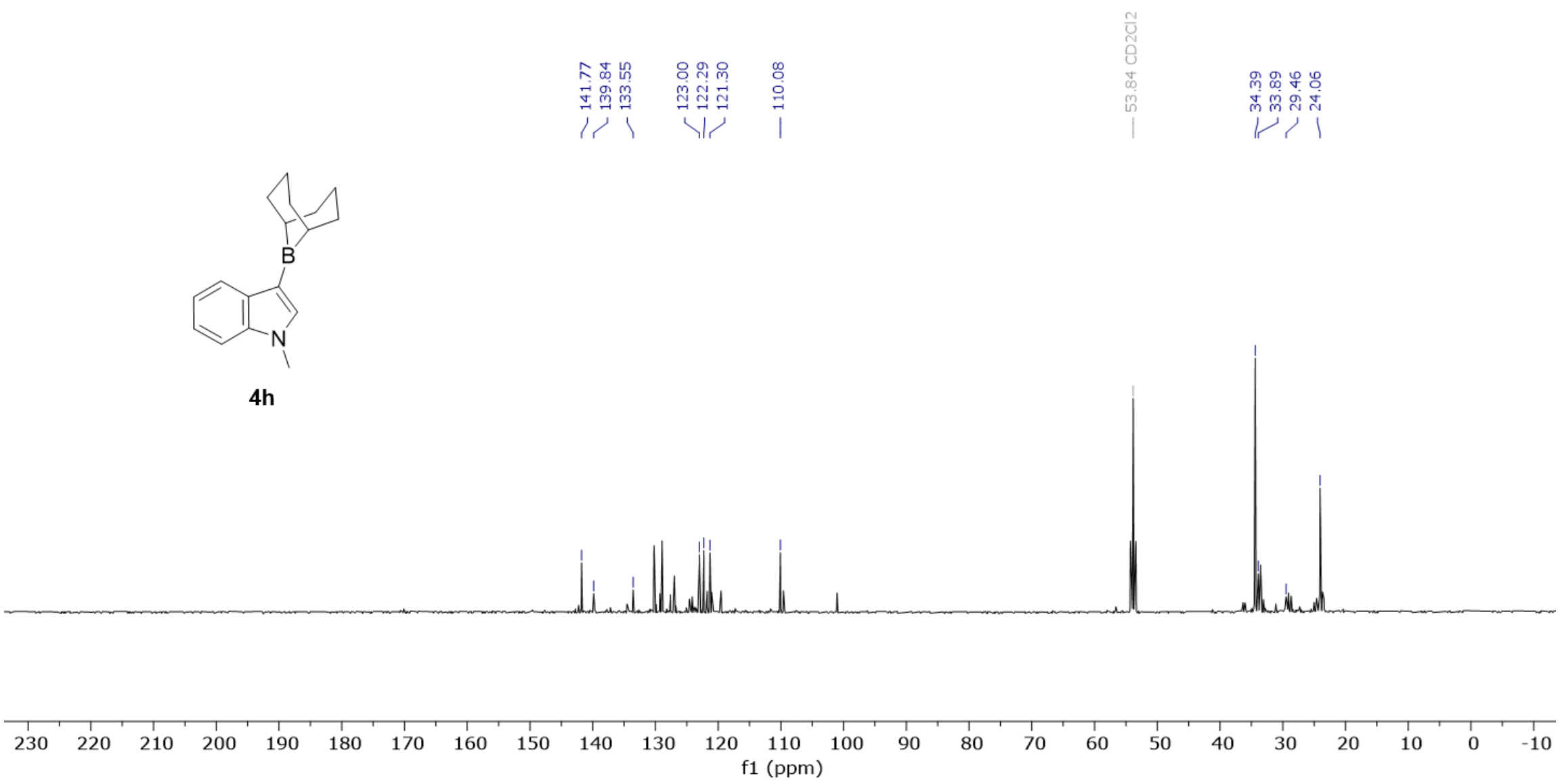

Figure S41: $^{13}C\{^{1}H\}$ NMR spectrum of crude compound **4h** in CD_2Cl_2.

S4.2. Glovebox-free zinc catalysed C–H mono-borylation of 2-methyl-thiophene, **2a**

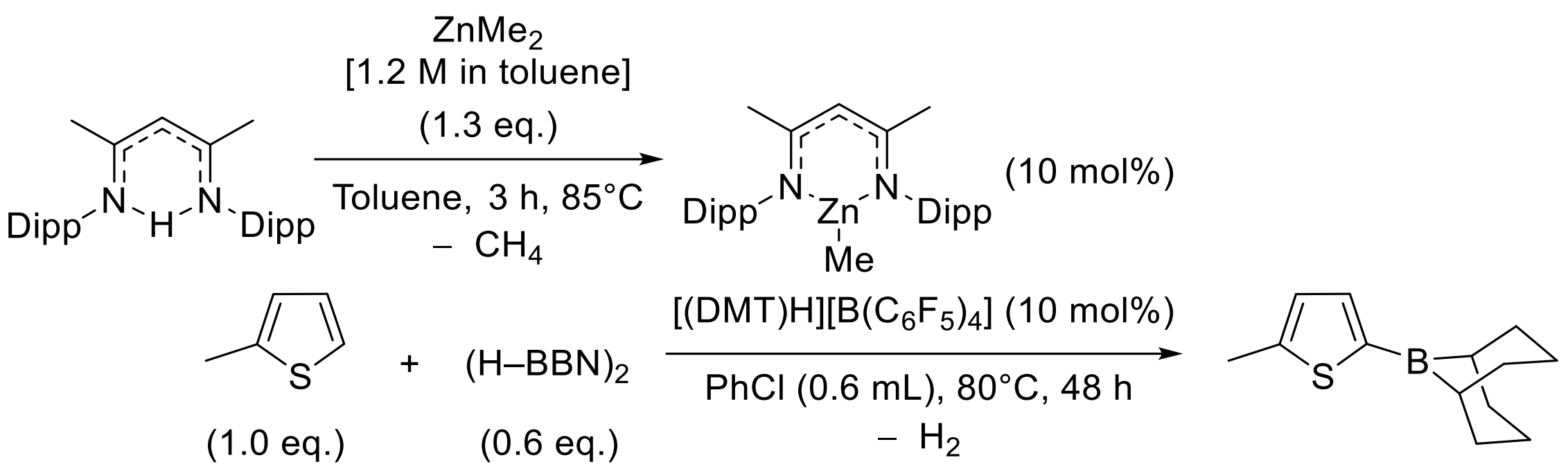

2a **4a, 95%**

$ZnMe_2$ (1.2 M in toluene, 0.26 mL, 0.31 mmol, 1.3 eq.) was added at ambient temperature to a solution of ^{Dipp}NacNacH (100.5 mg, 0.24 mmol, 1.0 eq.) in toluene. The solution was heated at 85°C for 3 h until gas evolution stopped. The resulting solution of ^{Dipp}NacNacZnMe (0.23 M in toluene, 0.1 mL, 0.02 mmol, 10 mol%) was added to an ampule with a solution of [(DMT)H][B(C_6F_5)$_4$] (19 mg, 0.02 mmol, 10 mol%) in PhCl (0.6 mL). Subsequently, 2-methyl-thiophene (20 µL, 0.21 mmol, 1.0 equiv.) and (H−BBN)$_2$ (0.4 M wrt monomer in hexanes, 0.6 mL, 0.12 mmol of dimer, 0.6 equiv.) were added and the reaction mixture heated at 80°C for 48 h. Upon completion, dibromomethane (14.5 µL, 0.21 mmol) was added to the reaction mixture as an internal standard to determine *in-situ* yield by the integration of diagnostic ^{1}H resonances (95%).

Analytical data are consistent with that shown previously (Section S4.1.1.).

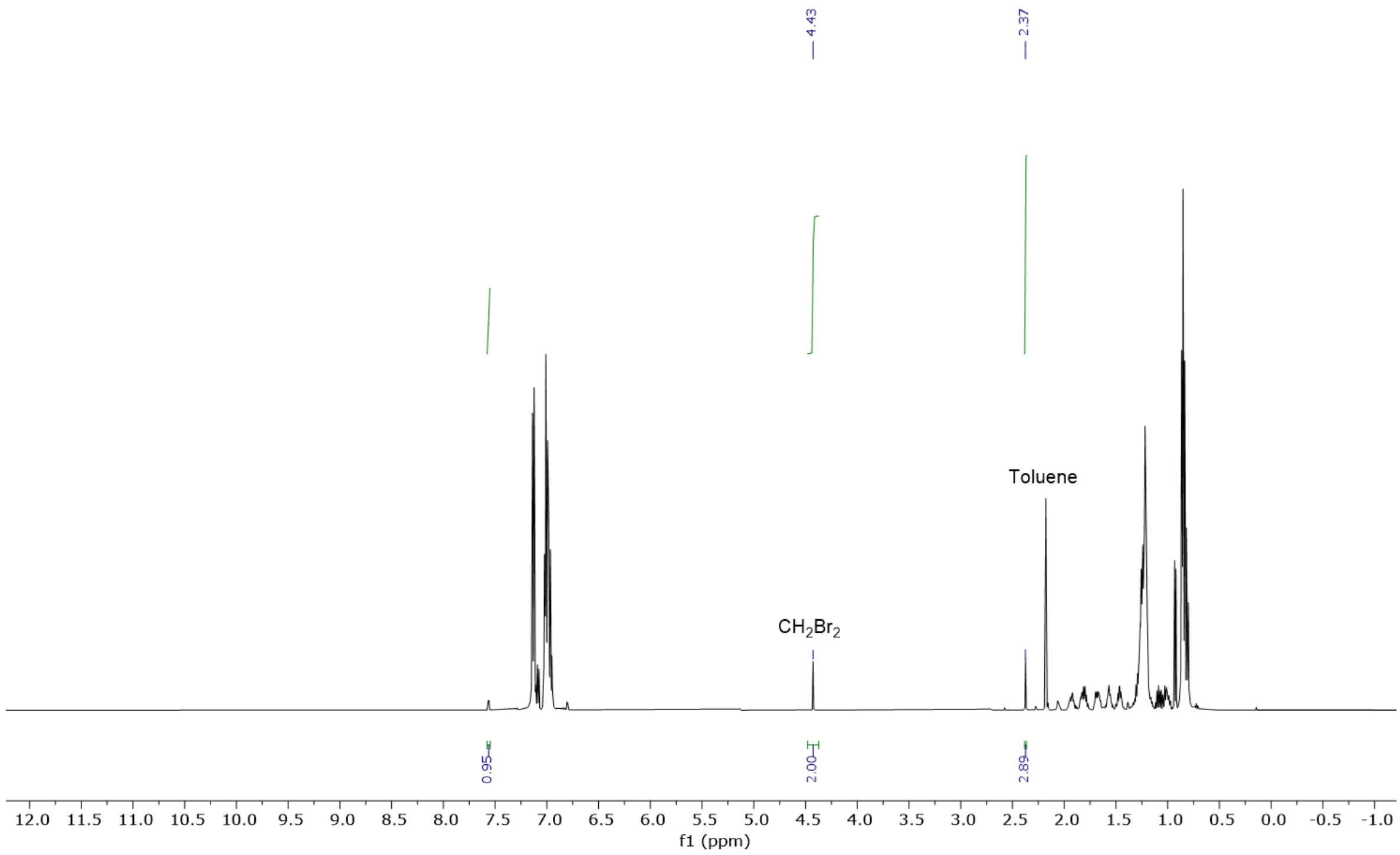

Figure S42: C–H borylation of 2-methyl-thiophene in PhCl by *in-situ* ^{1}H NMR spectroscopy.

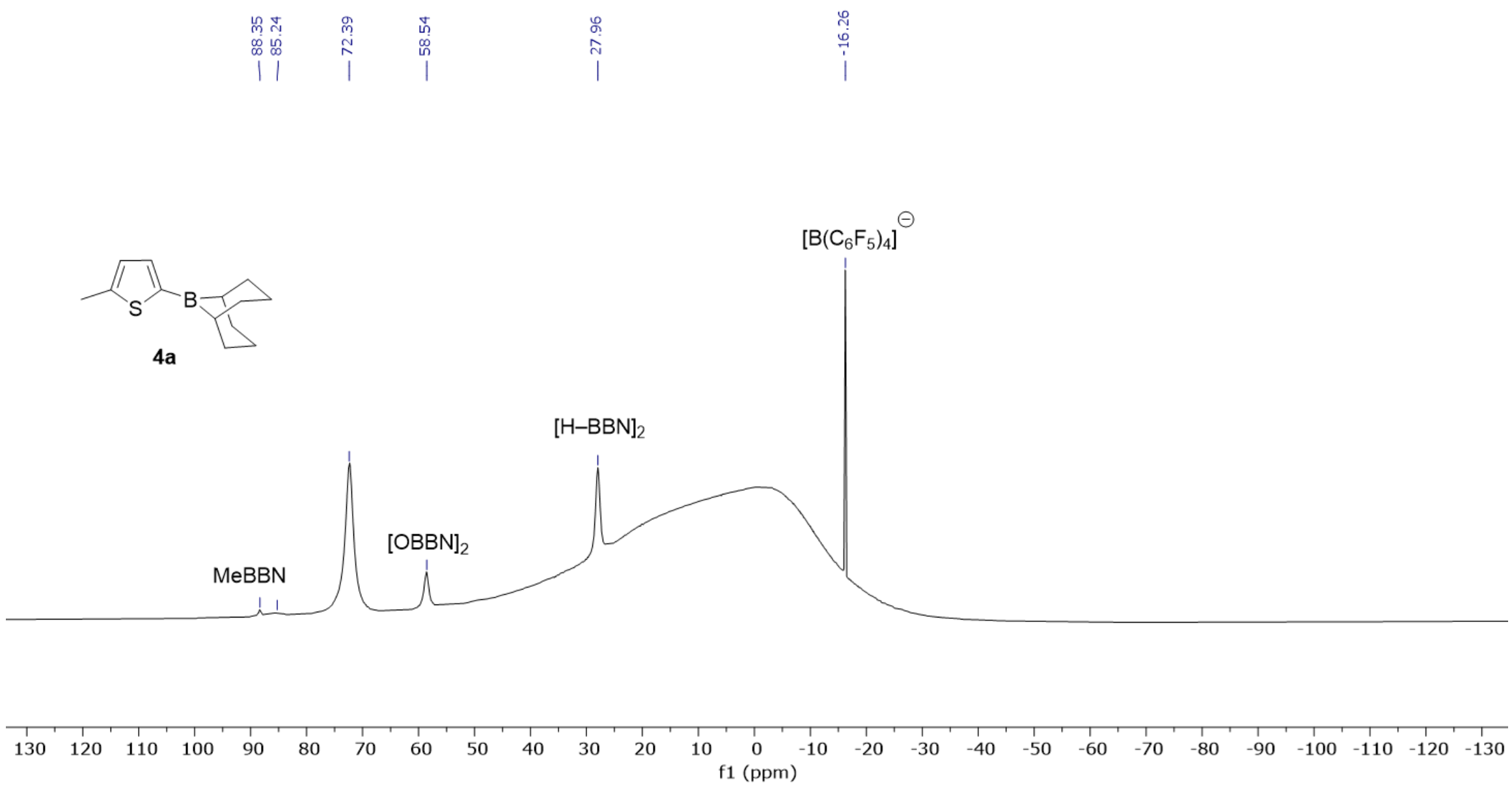

Figure S43: ^{11}B NMR spectrum of the crude reaction mixture in PhCl.

S5. Mechanistic Studies

S5.1. Reaction of ^{Dipp}NacNacZnMe with [(DMT)H][B(C_6F_5)$_4$] forming **[1-DMT][B(C_6F_5)$_4$]**

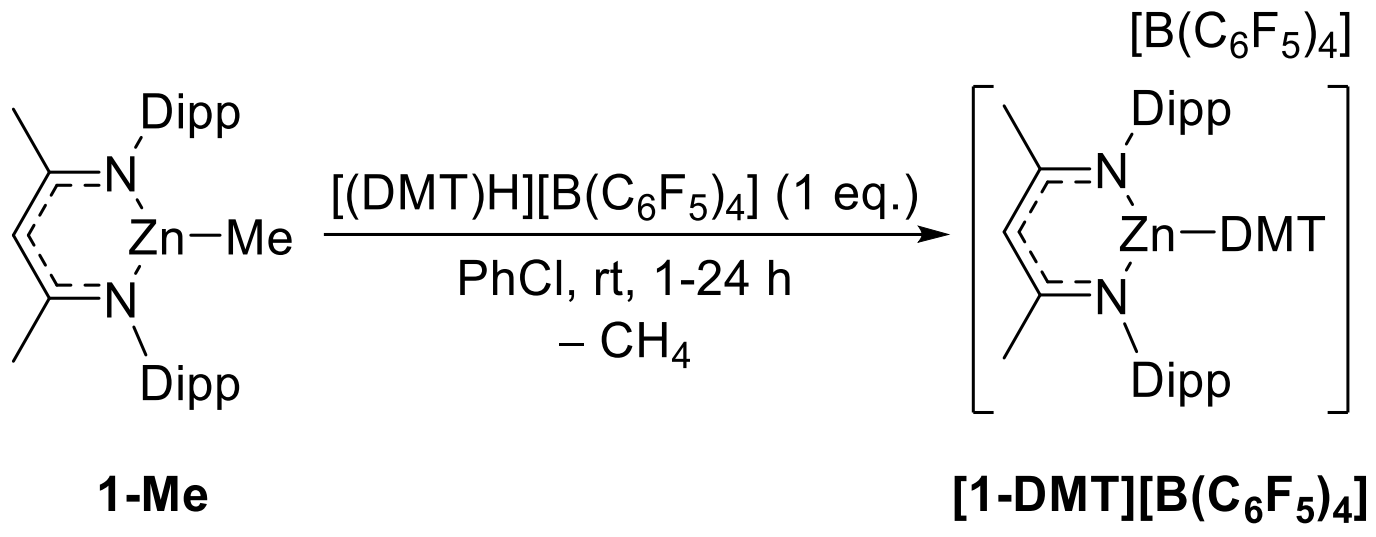

A J Young's NMR tube was charged with ^{Dipp}NacNacZnMe (6 mg, 0.01 mmol, 1.0 eq.) and [(DMT)H][B(C_6F_5)$_4$] (10 mg, 0.01 mmol, 1.0 eq.) in a glovebox before dissolution in PhCl (0.6 mL). The reaction mixture was sealed and mixed well by rotation (ca. 30 rpm) at rt for 24 h. Gas formation was observed and the J Young's NMR tube was vented regularly. The reaction was monitored via ^{1}H NMR spectroscopy and after 24 h at rt formation of a new compound assigned to [^{Dipp}NacNacZn–DMT][B(C_6F_5)$_4$] was identified (>85% conversion).

Analytical data are consistent with that previously reported.[4]

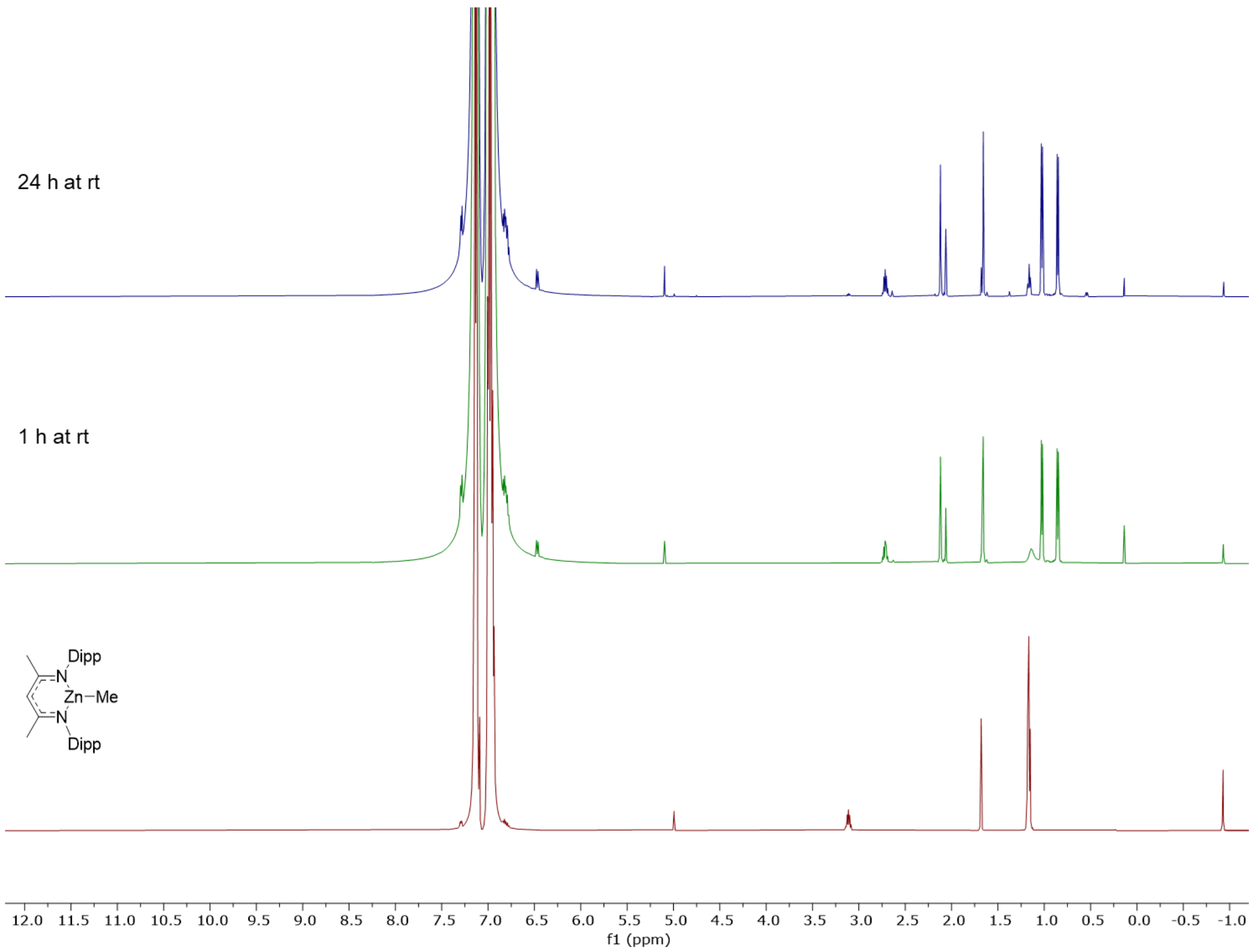

Figure S44: Stacked ^{1}H NMR spectra in PhCl of: ^{Dipp}NacNacZnMe (red), ^{Dipp}NacNacZnMe with [(DMT)H][B(C_6F_5)$_4$] after 1 h at rt (green) and ^{Dipp}NacNacZnMe with [(DMT)H][B(C_6F_5)$_4$] after 24 h at rt (blue).

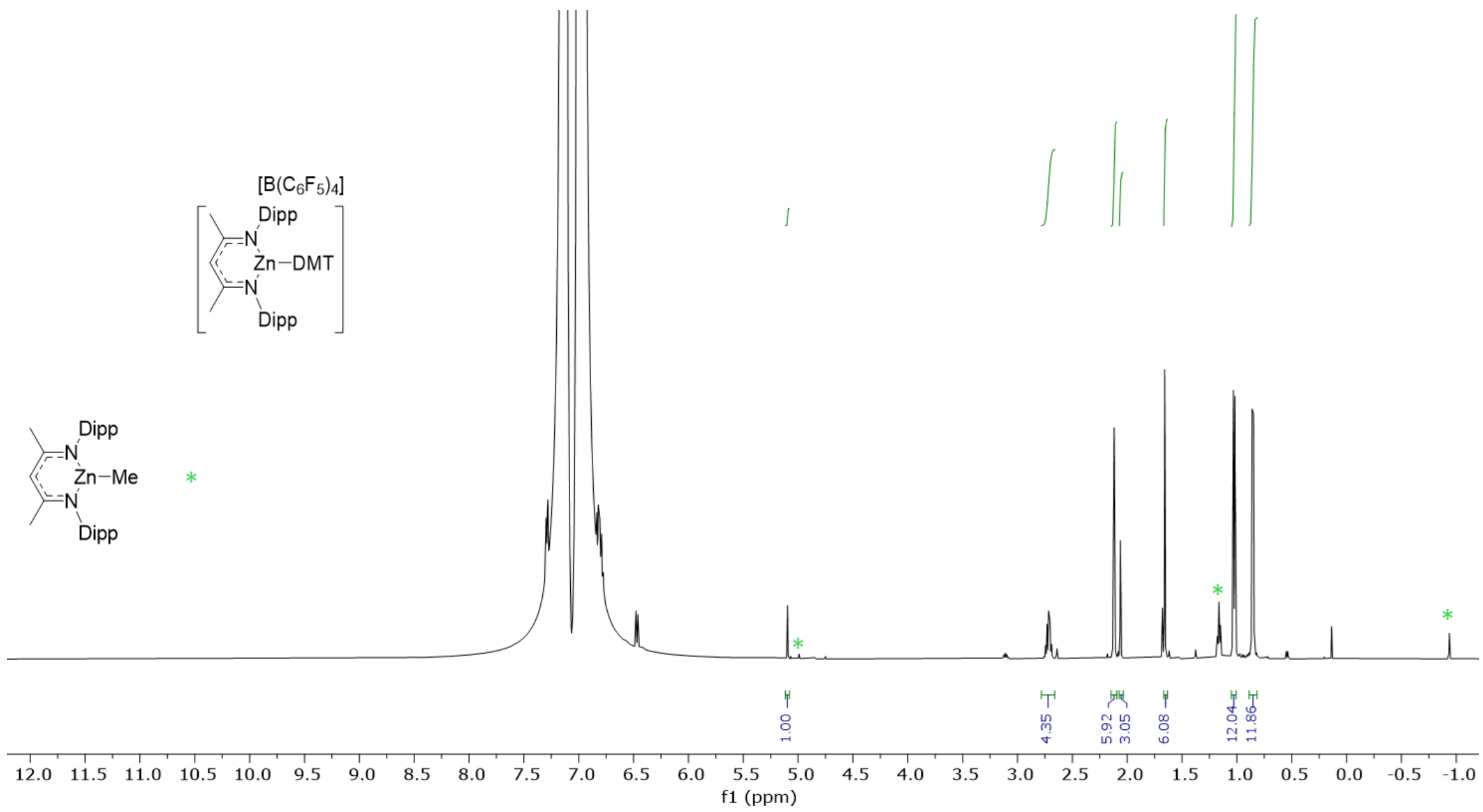

Figure S45: ^{1}H NMR spectrum of crude [^{Dipp}NacNacZn−DMT][B(C_6F_5)$_4$] in PhCl.

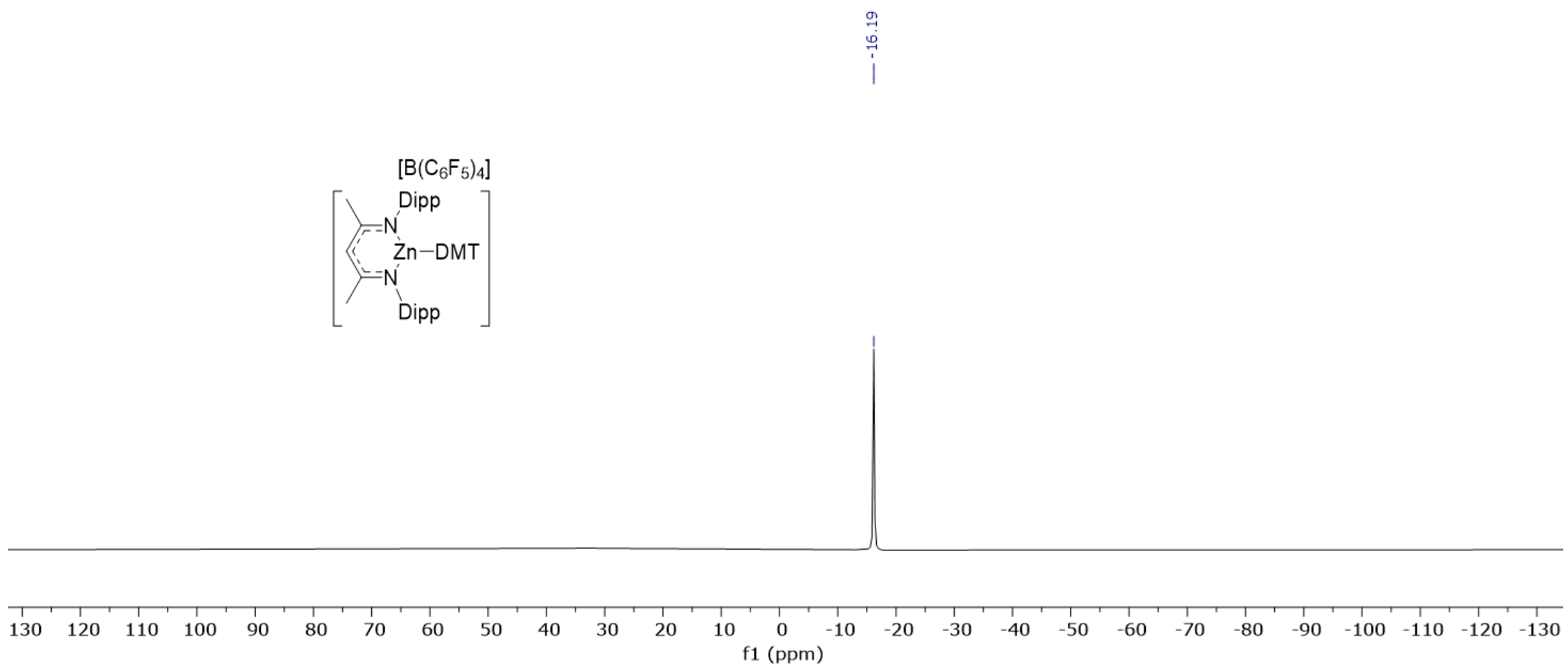

Figure S46: ^{11}B NMR spectrum of crude [^{Dipp}NacNacZn−DMT][$B(C_6F_5)_4$] in PhCl.

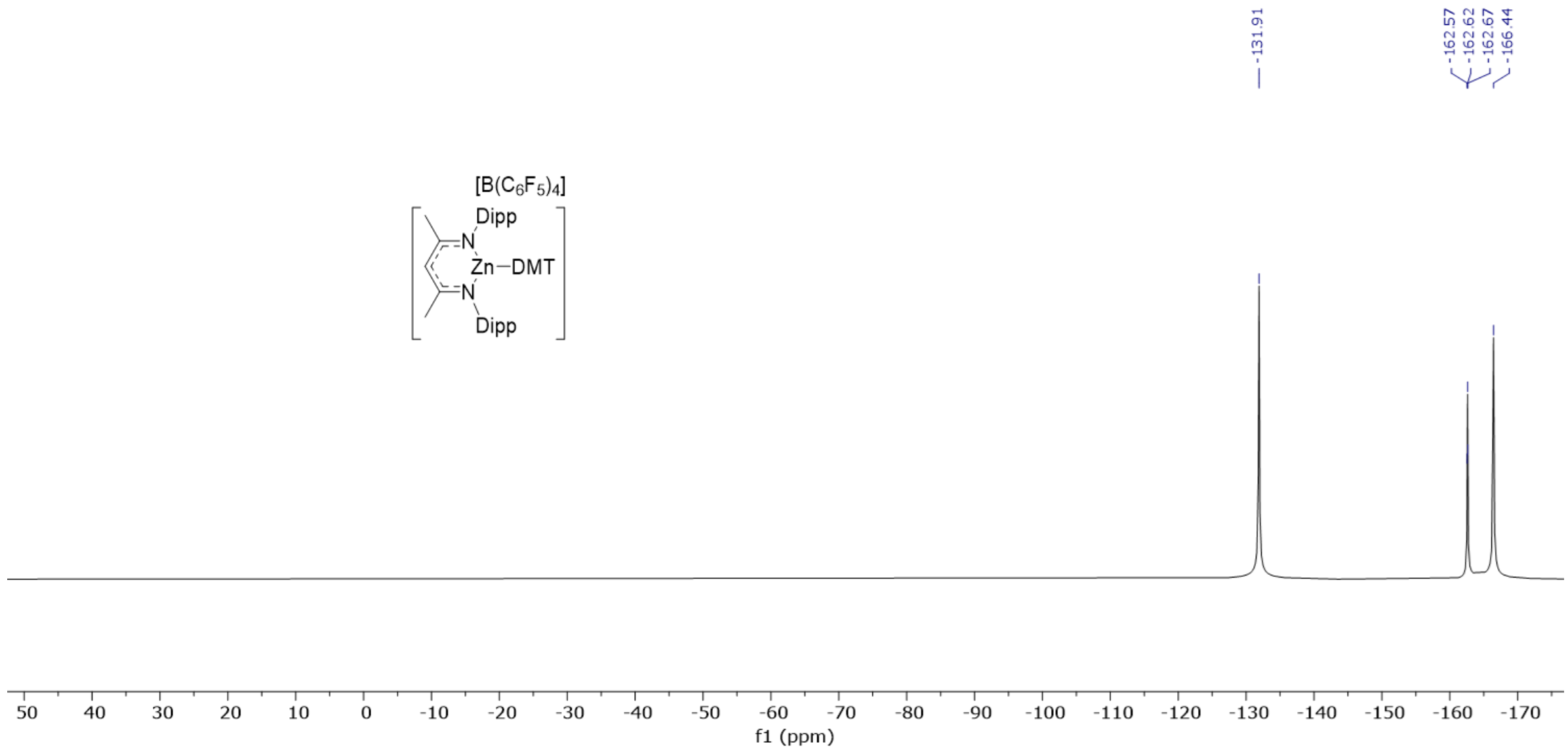

Figure S47: ^{19}F NMR spectrum of crude [^{Dipp}NacNacZn−DMT][$B(C_6F_5)_4$] in PhCl.

S5.2. Reactivity of **[^{Dipp}NacNacZn($CHB_{11}H_5Br_6$)]**

S5.2.1. Synthesis of **[^{Dipp}NacNacZn($CHB_{11}H_5Br_6$)]**

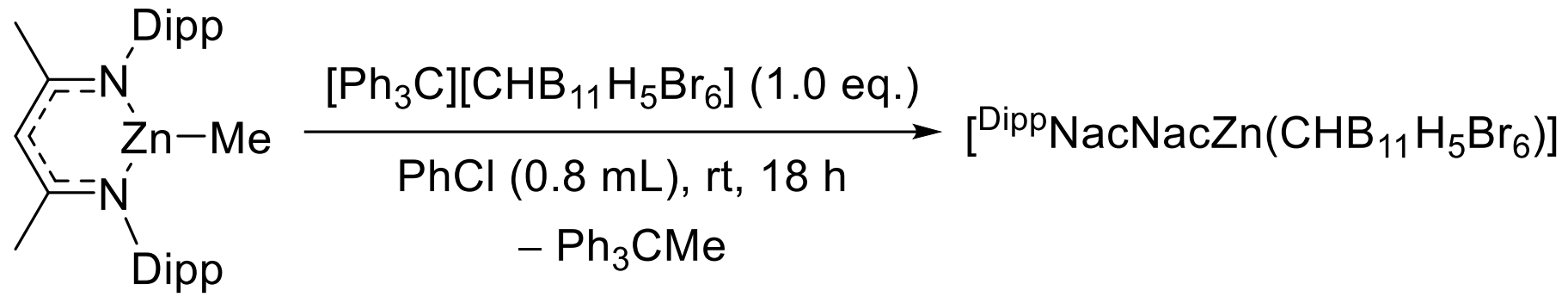

1-Me **[1-$CHB_{11}H_5Br_6$]**, 95%

Method A (yield determination): A J Young's NMR tube was charged with ^{Dipp}NacNacZnMe (11 mg, 0.02 mmol, 1.0 eq.) and [Ph_3C][$CHB_{11}H_5Br_6$] (19 mg, 0.02 mmol, 1.0 eq.) in a glovebox before dissolution in PhCl (0.8 mL). The reaction mixture was sealed and mixed by sonication (at 40 °C) for 5 min to break down solids followed by rotation (ca. 30 rpm) at rt for 18 h. A clear yellow solution was obtained. Dibromomethane (2 M in PhCl, 11 μL, 0.02 mmol) was added to the reaction mixture as an internal standard to determine the *in-situ* yield (95%) by the integration of diagnostic ^{1}H (e.g., NacNac C_γ–H) resonances.

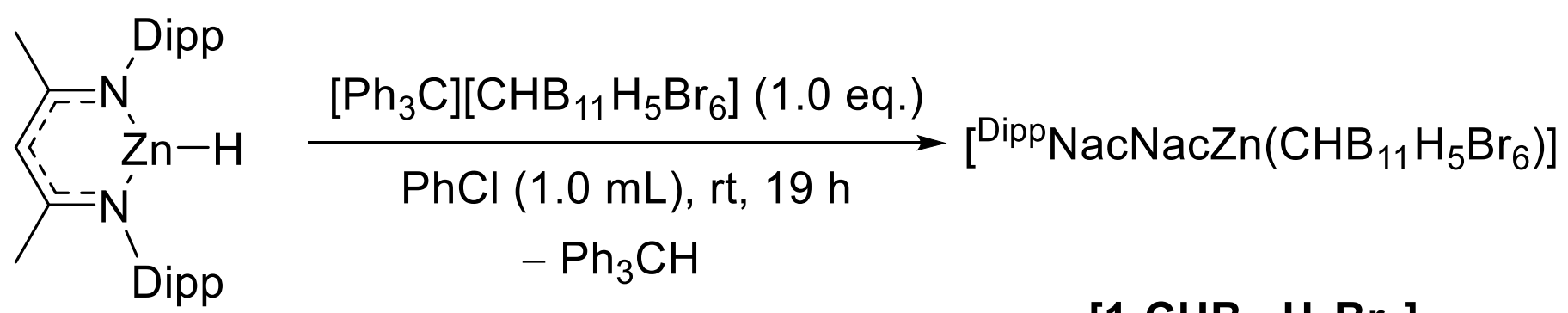

[1-$CHB_{11}H_5Br_6$]

Method B (crystals formation): A J Young's NMR tube was charged with NacNacZnH (0.019 g, 0.039 mmol. 1.0 equiv.) and [Ph_3C][$CHB_{11}H_5Br_6$] (0.034 g, 0.039 mmol. 1.0 equiv.) in a glovebox before dissolution in PhCl (1 mL). The reaction mixture was sealed and mixed by sonication (at 40 °C) for 30 min to break down solids followed by rotation (ca. 30 rpm) at rt for 19 h. A clear yellow solution was obtained and formation of **[1-$CHB_{11}H_5Br_6$]** was confirmed via ^{1}H NMR spectroscopy. The solution was concentrated and layered with pentane (*ca.* 5 mL). After 10 days at rt, the formation of white crystals was observed. Crystallisation from the reaction mixture afforded single crystals suitable for the X-ray diffraction.

^{1}H NMR (500 MHz, C_6D_5Br): δ 7.14 – 7.08 (m, 2H, p-Ar-*CH*), 7.07 – 7.02 (m, 4H, m-Ar-*CH*), 4.88 (s, 1H, C_γ–*H*), 2.94 (sept, J = 6.7 Hz, 4H, *CH*(CH_3)$_2$), 2.01 (br s, 1H, C*H*$B_{11}H_5Br_6$), 1.64 (s, 6H, CH(NC*CH$_3$*)$_2$), 1.33 (d, J = 6.8 Hz, 12H, CH(*CH$_3$*)$_2$), 1.03 (d, J = 6.7 Hz, 12H, CH(*CH$_3$*)$_2$). B–H peaks could not be resolved due to broadening and overlap.

^{1}H{^{11}B} NMR (500 MHz, C_6D_5Br): δ 7.14 – 7.08 (m, 2H, p-Ar-*CH*), 7.07 – 7.02 (m, 4H, m-Ar-*CH*), 4.88 (s, 1H, C_{γ}–*H*), 2.94 (sept, *J* = 6.7 Hz, 4H, *CH*(CH_3)$_2$), 2.56 – 2.06 (br, 5H, CHB_{11}*H*$_5Br_6$), 2.01 (br s, 1H, *CH*$B_{11}H_5Br_6$), 1.64 (s, 6H, CH(NC*CH*$_3$)$_2$), 1.33 (d, *J* = 6.8 Hz, 12H, CH(*CH*$_3$)$_2$), 1.03 (d, *J* = 6.7 Hz, 12H, CH(*CH*$_3$)$_2$).

^{13}C{^{1}H} NMR (126 MHz, C_6D_5Br): δ 171.6 (s, CH(N*C*CH_3)$_2$), 142.6 (s, Ar-*C*CH(CH_3)$_2$), 141.9 (s, Ar-*C*N), 127.6 (s, p-Ar-*CH*), 124.7 (s, m-Ar-*CH*), 96.0 (s, C_{γ}–*H*), 42.0 (s, *CH*$B_{11}H_5Br_6$), 29.0 (s, *CH*(CH_3)$_2$), 25.5 (s, CH(*CH*$_3$)$_2$), 24.7 (s, CH(NC*CH*$_3$)$_2$), 24.6 (s, CH(*CH*$_3$)$_2$).

^{11}B NMR (160 MHz, C_6D_5Br): δ -1.8 (s), -10.2 (s), -20.5 (br. s).

^{11}B{^{1}H} NMR (160 MHz, C_6D_5Br): δ -1.8 (s), -10.2 (s), -20.5 (s).

Mass Spectrometry: Calculated [M^+] = 481.2561, Observed [M^+] = 481.2545; Calculated [M^-] = 616.6610, Observed [M^-] = 616.6566.

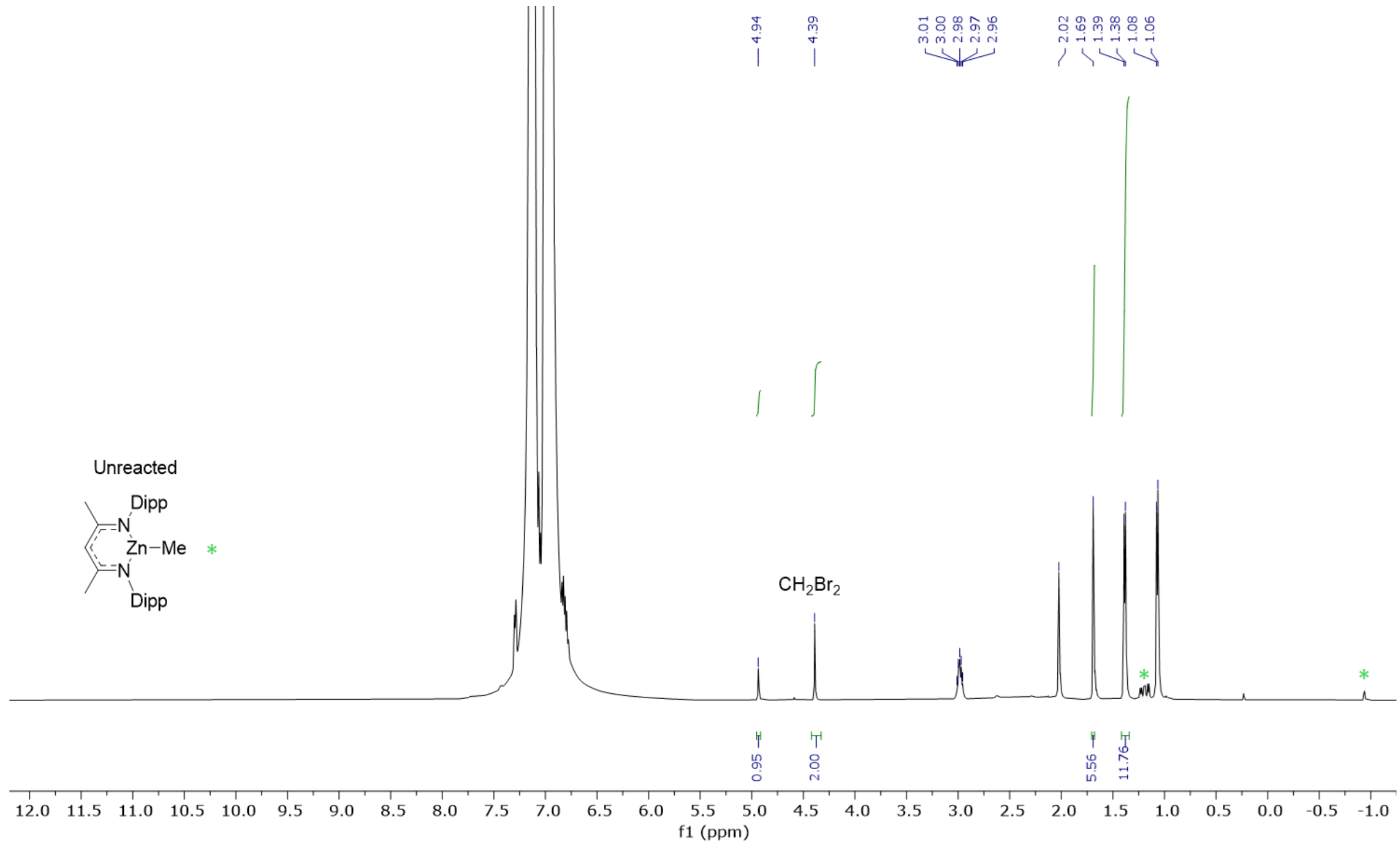

Figure S48: ^{1}H NMR spectrum of [^{Dipp}NacNacZn($CHB_{11}H_5Br_6$)] in PhCl with CH_2Br_2 (2 M in PhCl, 11 μL, 0.02 mmol) added as an internal standard.

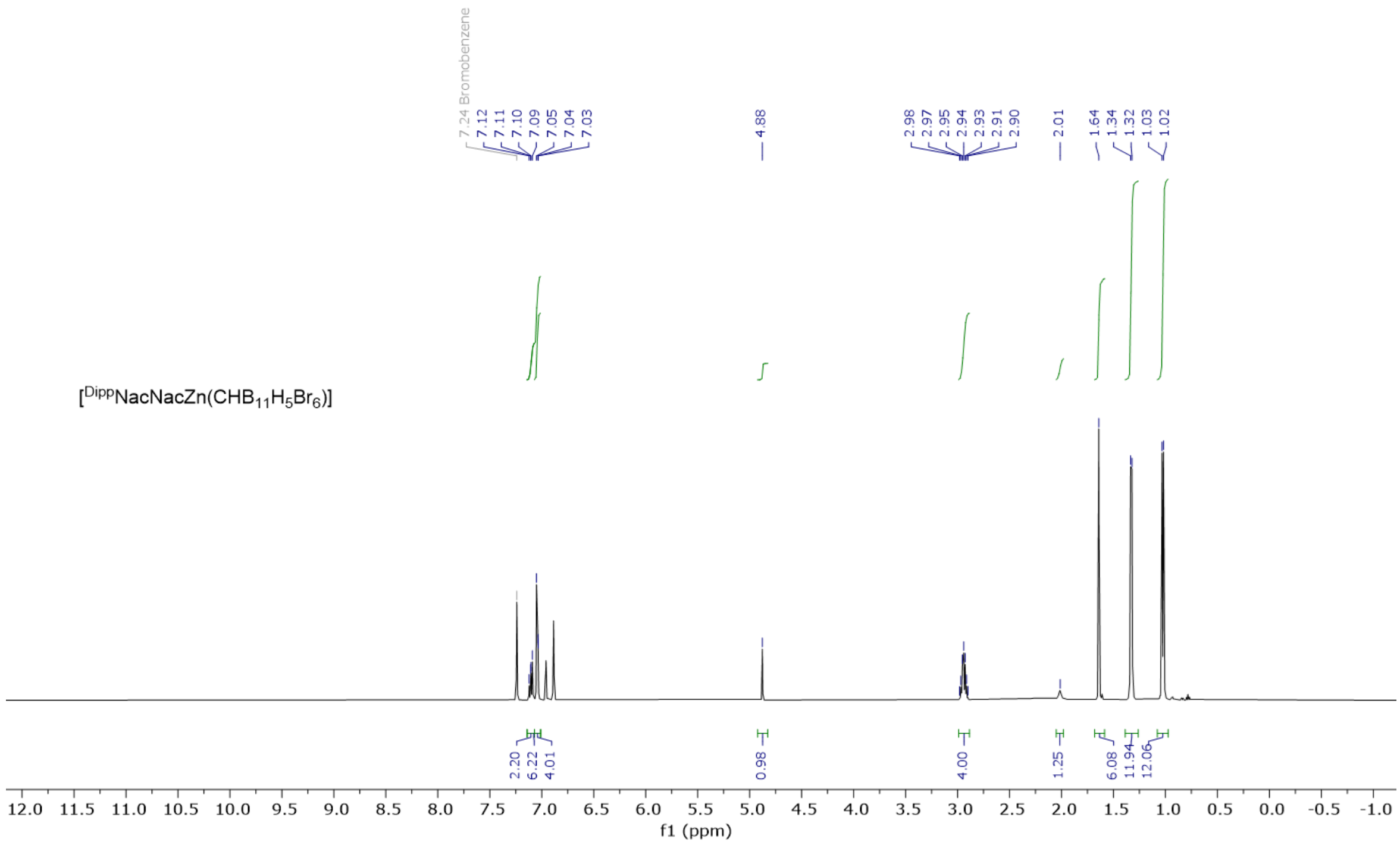

Figure S49: ^{1}H NMR spectrum of [^{Dipp}NacNacZn($CHB_{11}H_5Br_6$)] in C_6D_5Br.

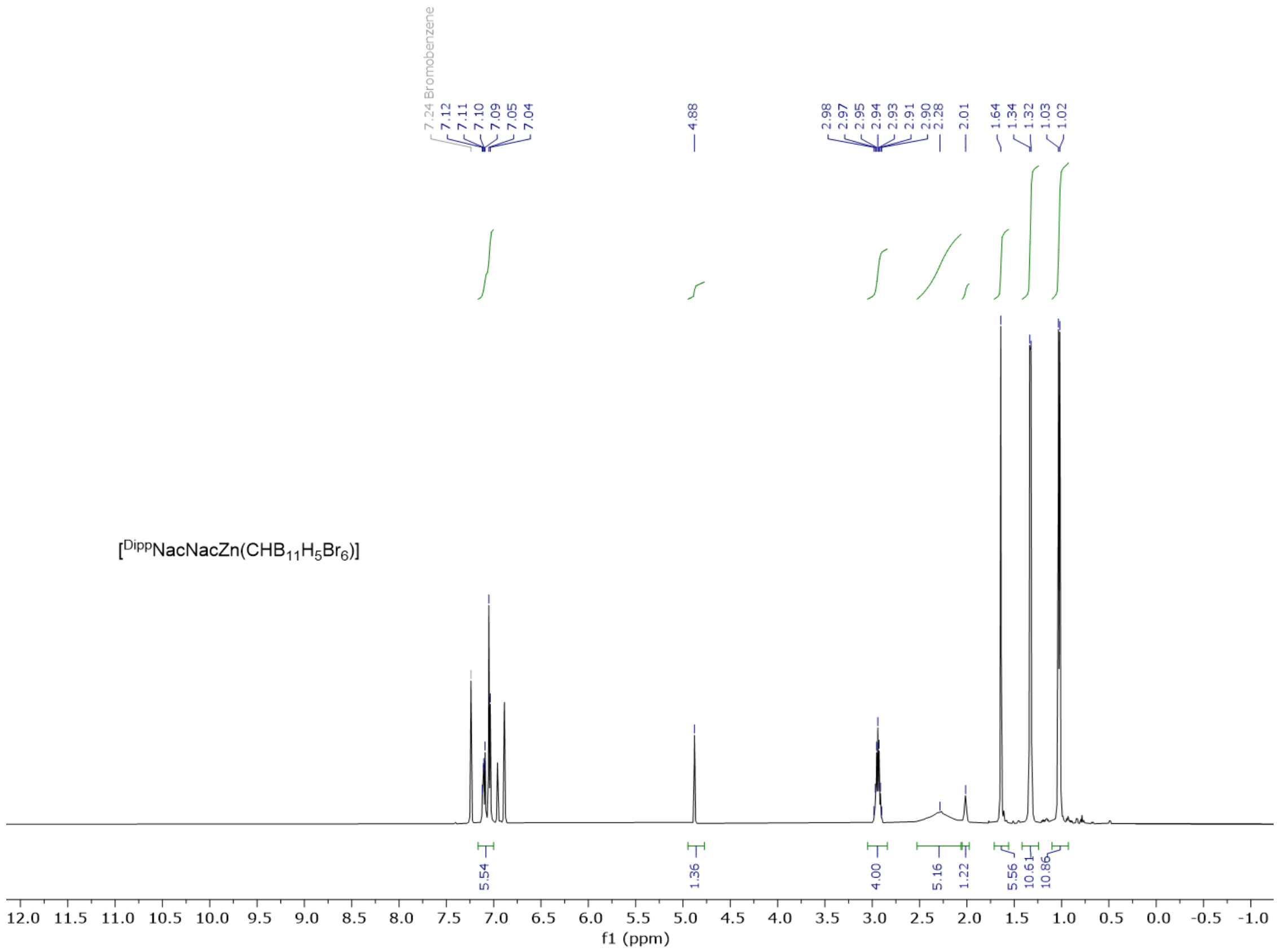

Figure S50: $^{1}H\{^{11}B\}$ NMR spectrum of [^{Dipp}NacNacZn($CHB_{11}H_5Br_6$)] in C_6D_5Br.

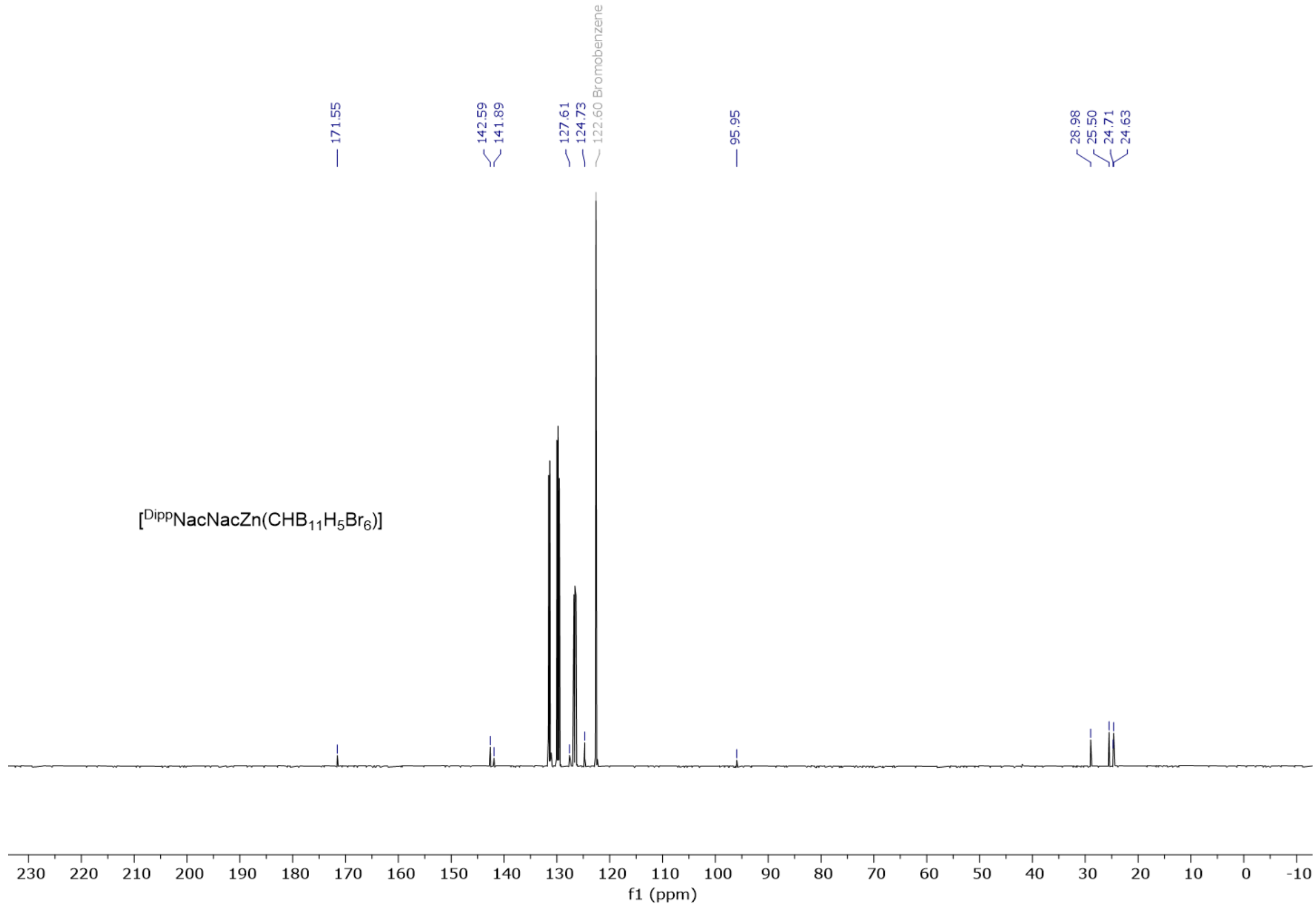

Figure S51: $^{13}C\{^{1}H\}$ NMR spectrum of [^{Dipp}NacNacZn($CHB_{11}H_5Br_6$)] in C_6D_5Br.

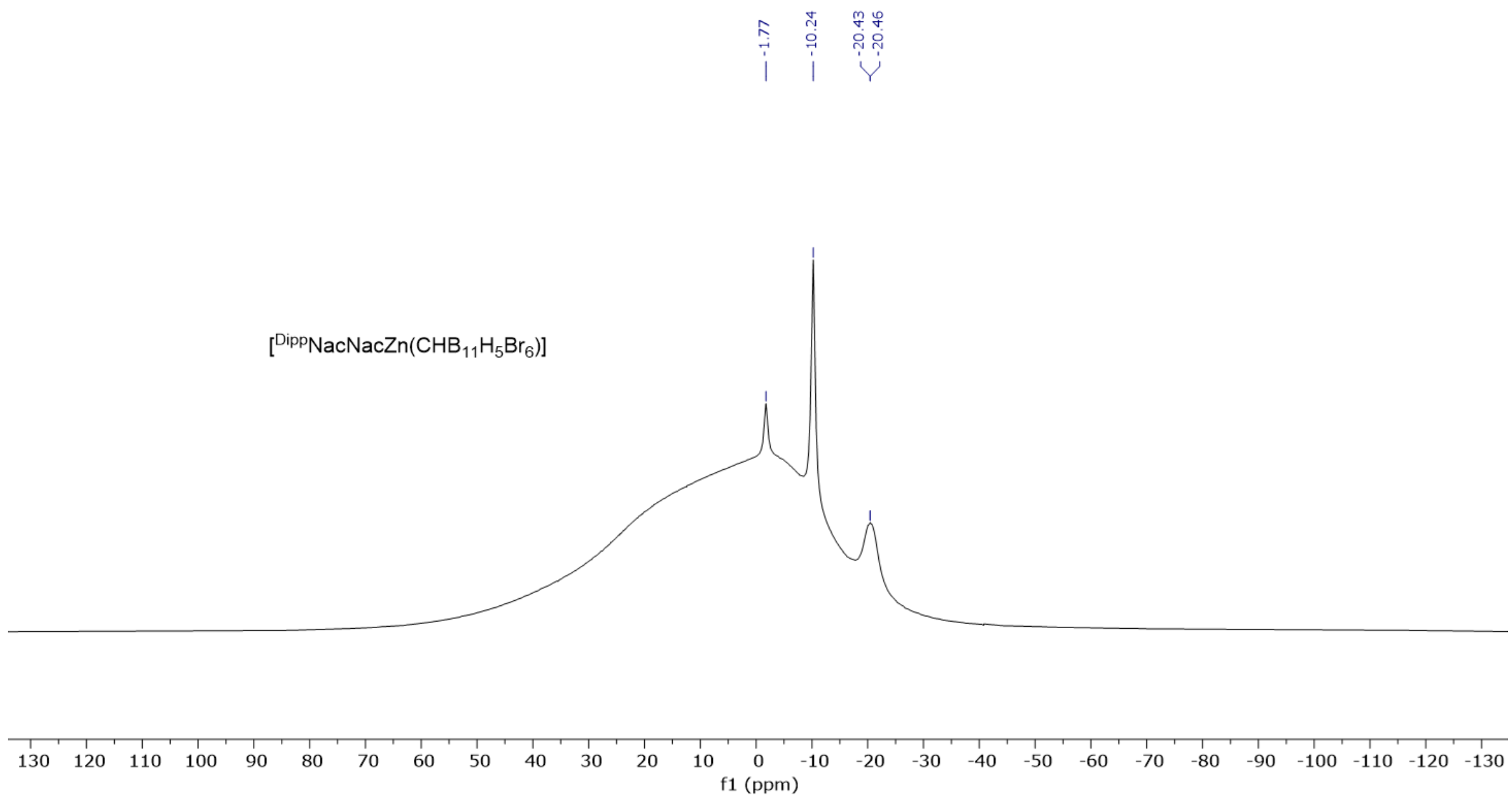

Figure S52: ^{11}B NMR spectrum of [^{Dipp}NacNacZn($CHB_{11}H_5Br_6$)] in C_6D_5Br.

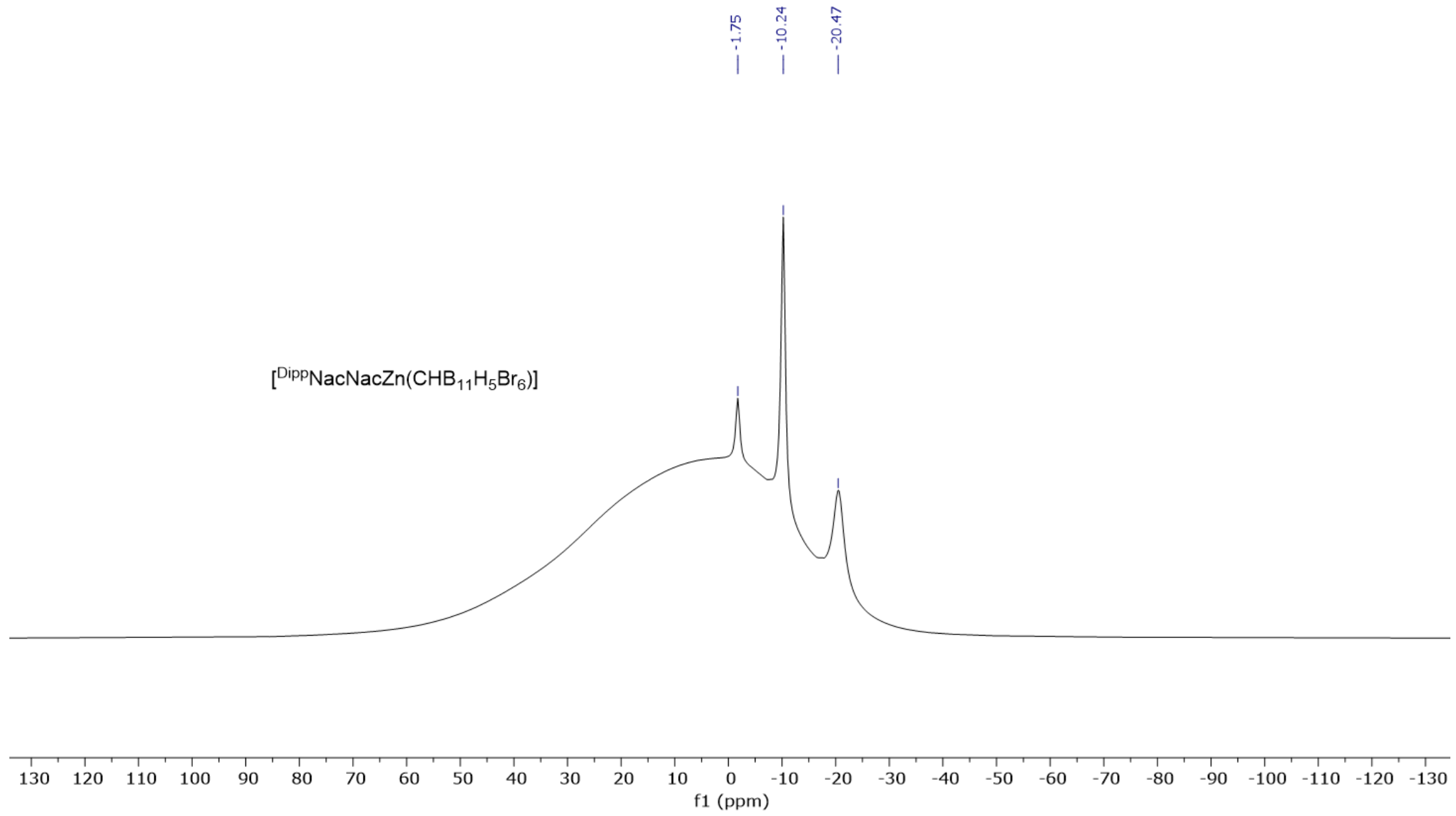

Figure S53: $^{11}B\{^{1}H\}$ NMR spectrum of [^{Dipp}NacNacZn($CHB_{11}H_5Br_6$)] in C_6D_5Br.

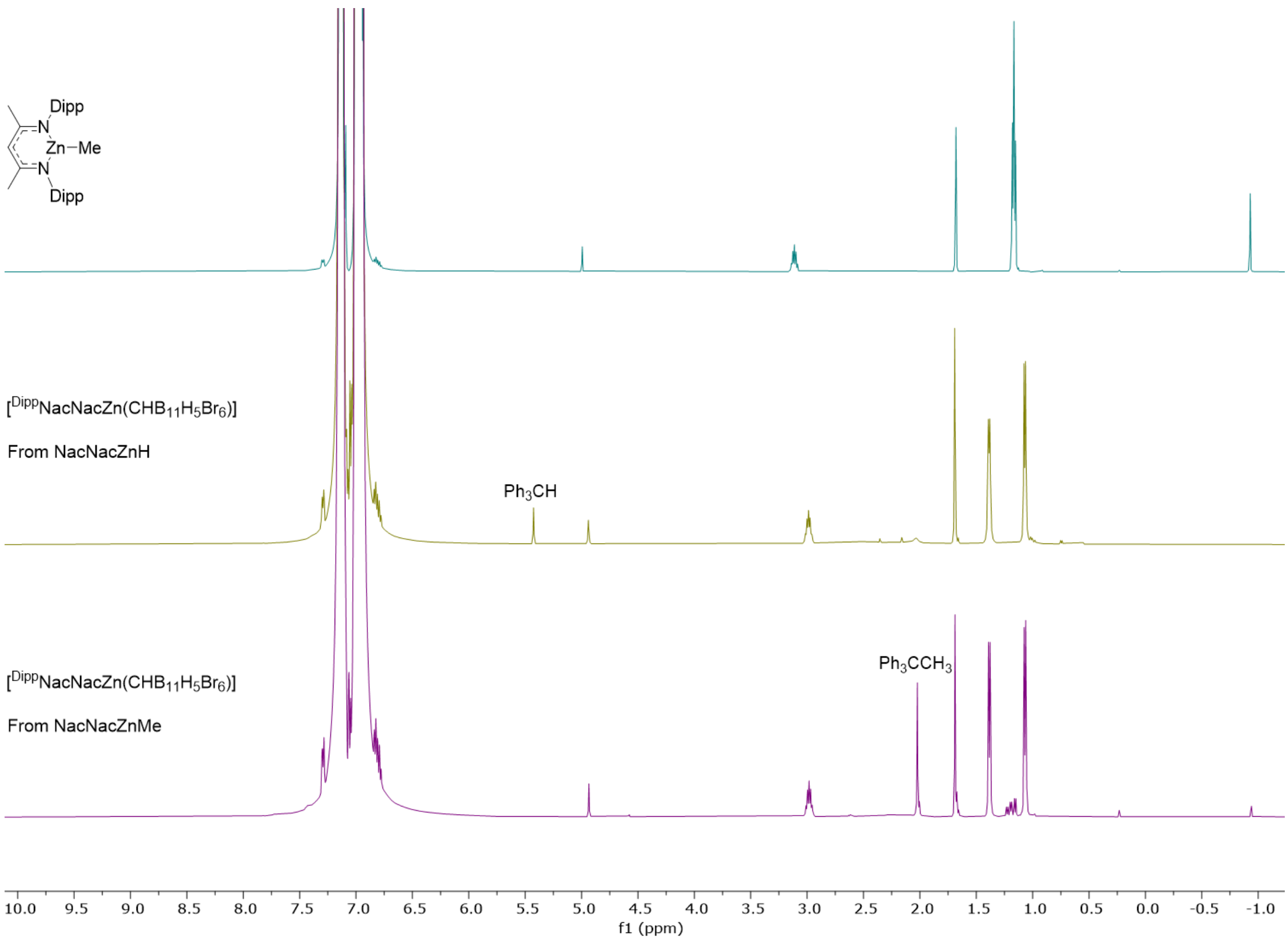

Figure S54: Stacked ^{1}H NMR spectra in PhCl of: ^{Dipp}NacNacZnMe, **1-Me** (top); [^{Dipp}NacNacZn($CHB_{11}H_5Br_6$)] synthesised from ^{Dipp}NacNacZnH (middle) and [^{Dipp}NacNacZn($CHB_{11}H_5Br_6$)] synthesised from ^{Dipp}NacNacZnMe (bottom).

S5.2.2. Decomposition study of **[^{Dipp}NacNacZn($CHB_{11}H_5Br_6$)]**

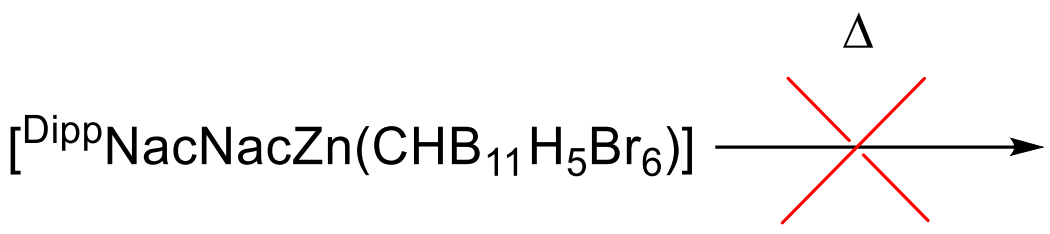

[1-$CHB_{11}H_5Br_6$]

[^{Dipp}NacNacZn($CHB_{11}H_5Br_6$)] was synthesised according to Method 1. The resulting solution was heated up to 110 °C. No decomposition was observed.

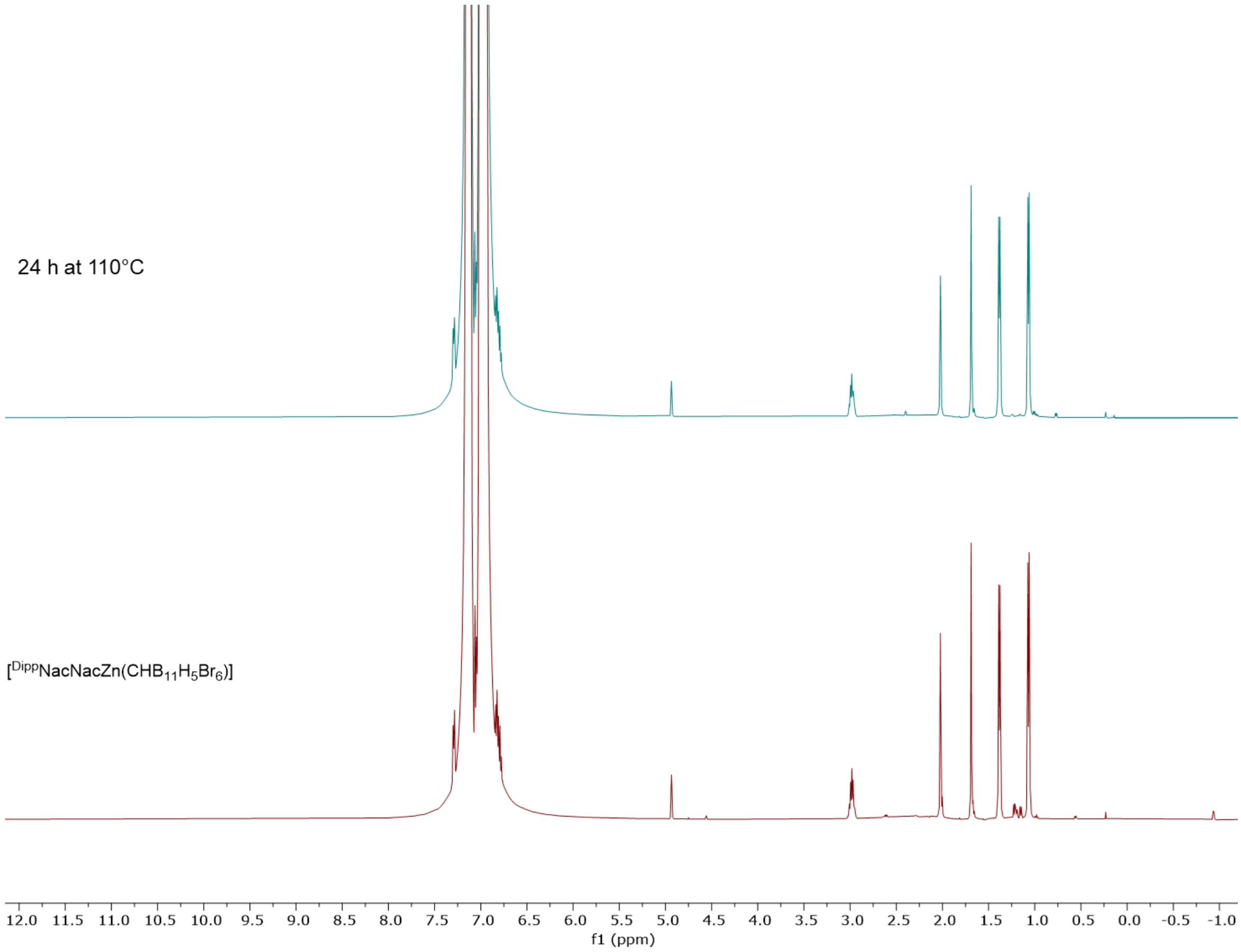

Figure S55: Stacked ^{1}H NMR spectra in PhCl of: [^{Dipp}NacNacZn($CHB_{11}H_5Br_6$)] before heating (bottom) and [^{Dipp}NacNacZn($CHB_{11}H_5Br_6$)] after 24 h at 110°C (top).

S5.2.3. Reaction of [^{Dipp}NacNacZn($CHB_{11}H_5Br_6$)] with DMT

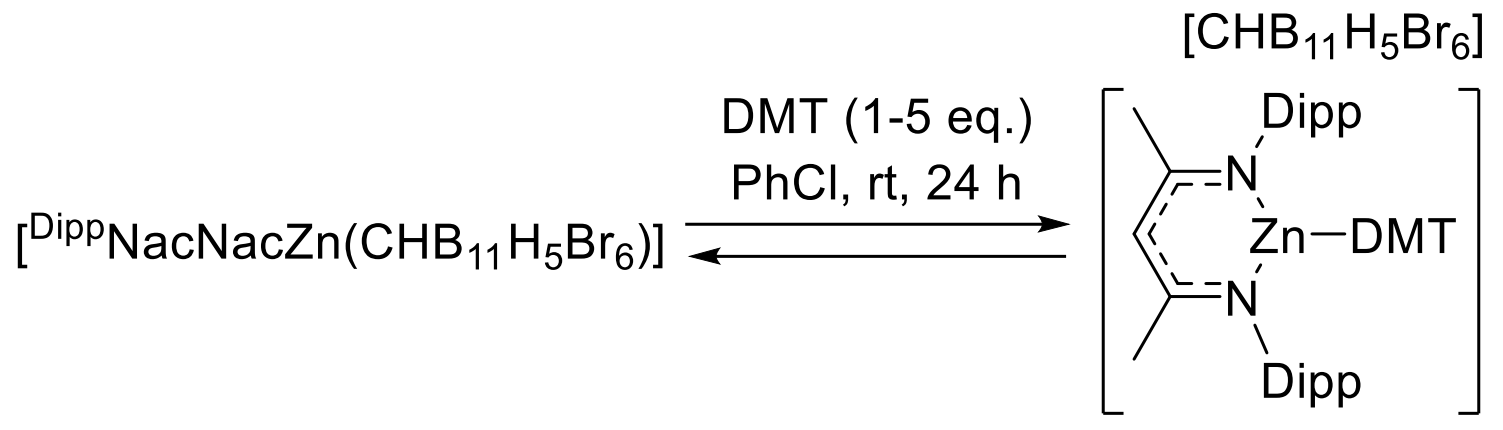

[1-$CHB_{11}H_5Br_6$] **[1-DMT][$CHB_{11}H_5Br_6$]**

[^{Dipp}NacNacZn($CHB_{11}H_5Br_6$)] was synthesised according to Method A. DMT (0.22 M in PhCl, 100 µL, 0.02 mmol, 1 eq.) was added to the solution of [^{Dipp}NacNacZn($CHB_{11}H_5Br_6$)]. A new compound formed at room temperature which was assigned to [^{Dipp}NacNacZn–DMT][$CHB_{11}H_5Br_6$]. The solution also contained free DMT. Prolonged heating at 60°C did not alter the ratio indicating the equilibrium position had been reached. More DMT (12.5 µL, 0.09 mmol, 5 eq. overall) was added which resulted in formation of a single product by ^{1}H NMR spectroscopy after 30 minutes at rt.

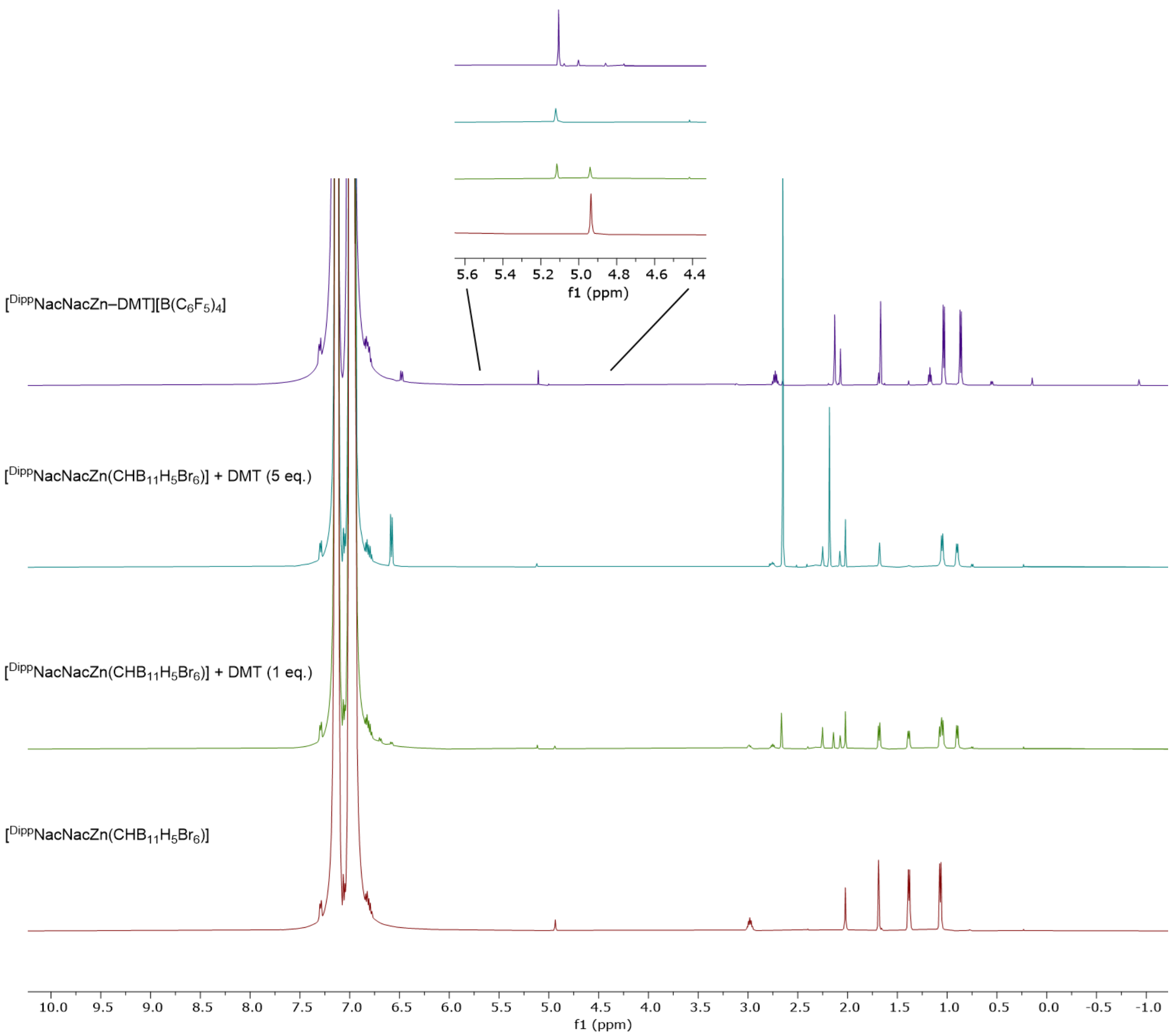

Figure S56: Stacked ^{1}H NMR spectra in PhCl of: [^{Dipp}NacNacZn(CHB$_{11}$H$_5$Br$_6$)] with no DMT added (red); [^{Dipp}NacNacZn(CHB$_{11}$H$_5$Br$_6$)] with DMT (1 eq.) (green); [^{Dipp}NacNacZn(CHB$_{11}$H$_5$Br$_6$)] with DMT (5 eq.) (blue) and *in-situ* [^{Dipp}NacNacZn–DMT][B(C$_6$F$_5$)$_4$] synthesised from the reaction of ^{Dipp}NacNacZnMe and [(DMT)H][B(C$_6$F$_5$)$_4$] (purple).

S5.3. Determination of experimental kinetic isotope effect (KIE) for C–H zincation (all experiments run in duplicates)

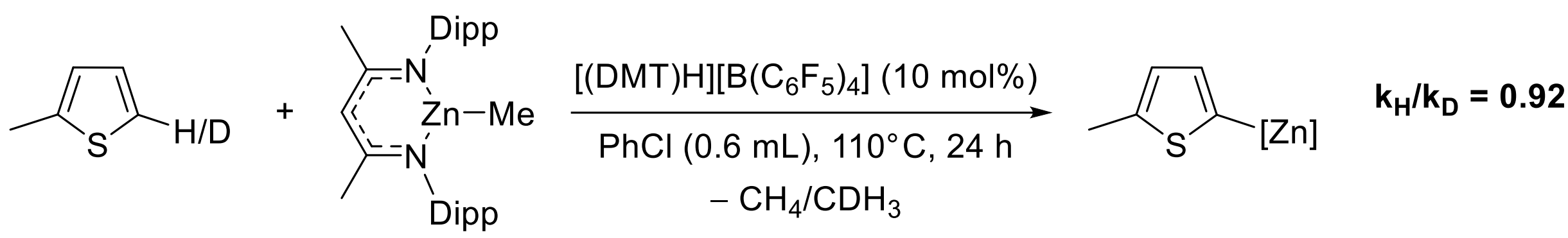

In a glovebox, ^{Dipp}NacNacZnMe (100.0 mg, 0.20 mmol) and [(DMT)H][B(C_6F_5)$_4$] (16 mg, 0.02 mmol) charged in a J. Young's NMR tube were dissolved in PhCl (0.6 mL). Subsequently, 2-methyl-thiophene (40 µL, 0.40 mmol, 2.0 equiv. w.r.t. ^{Dipp}NacNacZnMe) or 5-*d*-2-methyl-thiophene (40.5 mg, 0.40 mmol, 2.0 equiv. w.r.t. ^{Dipp}NacNacZnMe) was added to the reaction mixture and heated at 110°C. Reaction progress was monitored via ^{1}H NMR spectroscopy in 2 h time intervals up to 10 h. Conversion to the product was determined by the integration of the diagnostic ^{1}H NMR resonances (comparing NacNac backbone -CH_3 resonances). Upon completion (24 h at 110°C), the reaction mixture was vented and dibromomethane (28.0 µL, 0.4 mmol) was added to the reaction mixture as an internal standard to confirm the *in-situ* yield by the integration of diagnostic ^{1}H (NacNac backbone -CH_3) resonances.

With the average taken from two different runs for each experiment, concentration of the product [**3a**] (with 2-methyl-thiophene) or [***d*-3a**] (with 5-*d*-2-methyl-thiophene) versus time (h) was plotted and the KIE calculated to be 0.92 [equation (1)].

Time (h)	[**3a**] (mM)	[***d*-3a**] (mM)
2	0.13375	0.11802
4	0.17309	0.15736
6	0.19827	0.18568
8	0.21401	0.20456
10	0.22817	0.21873

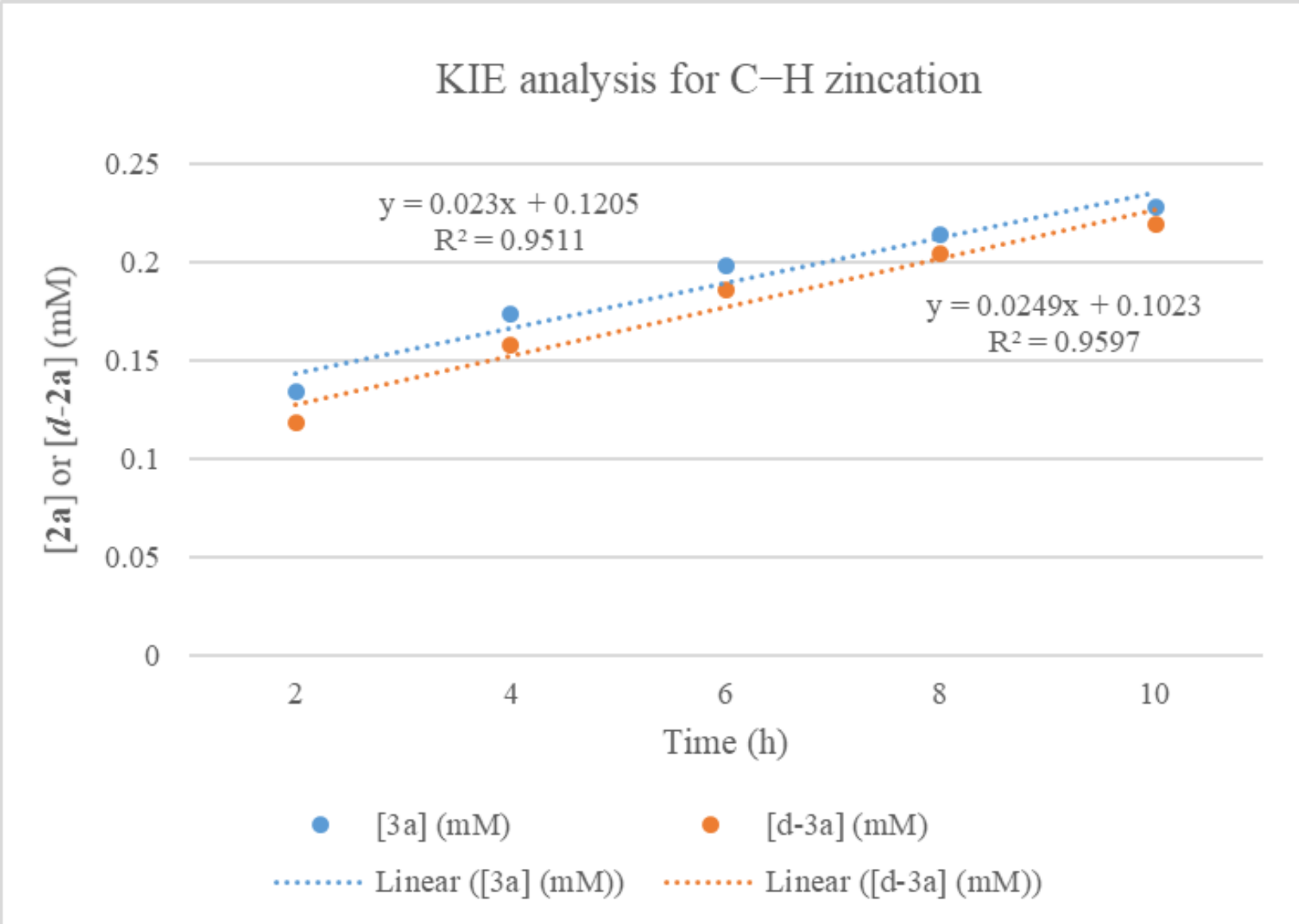

Figure S57: Concentration of **3a** vs ***d*-3a** as a function of time for the C–H zincation.

$$\frac{k_H}{k_D} = \frac{0.023}{0.025} = 0.92 \quad (1)$$

S5.4. Reaction of ^{Dipp}NacNacZnMe with (H−BBN)$_2$

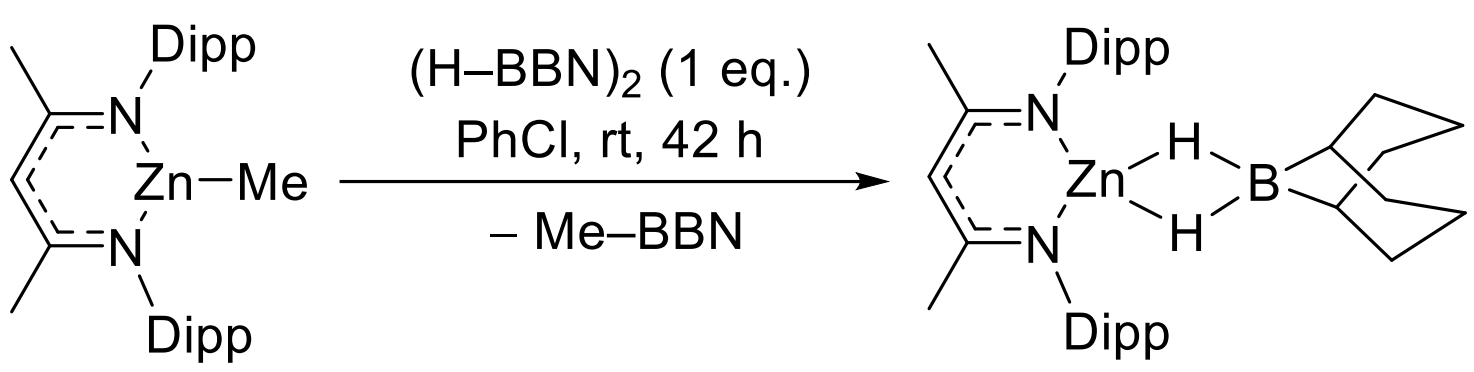

1-Me **1-H$_2$BBN**

A J Young's NMR tube was charged with ^{Dipp}NacNacZnMe (24 mg, 0.05 mmol, 1.0 eq.) and (H−BBN)$_2$ (6 mg, 0.025 mmol, 0.5 eq.) in a glovebox before dissolution in PhCl (0.6 mL). The reaction mixture was sealed and mixed well by rotation (ca. 30 rpm) at rt for 24 h. The reaction was monitored via ^{1}H NMR spectroscopy and formation of a new compound assigned to ^{Dipp}NacNacZn–(μ-H)$_2$–BBN was identified alongside formation of MeBBN. However, conversion was determined to be 50% due to the presence of unreacted ^{Dipp}NacNacZnMe. Further (H−BBN)$_2$ (6 mg, 0.025 mmol, 0.5 eq.; 1 eq. overall) was added in a glovebox. The

reaction mixture was sealed again and mixed well by rotation (ca. 30 rpm) at rt for ca. 18 h. The reaction was deemed completed as a single compound assigned to ^{Dipp}NacNacZn−(μ-H)$_2$–BBN was identified.

Analytical data are consistent with that previously reported.[9]

^{Dipp}NacNacZn−(μ-H)$_2$–BBN has previously been shown to undergo protonolysis with [(DMT)H][B(C_6F_5)$_4$] to [^{Dipp}NacNacZn−DMT][B(C_6F_5)$_4$] and H_2 at room temperature.

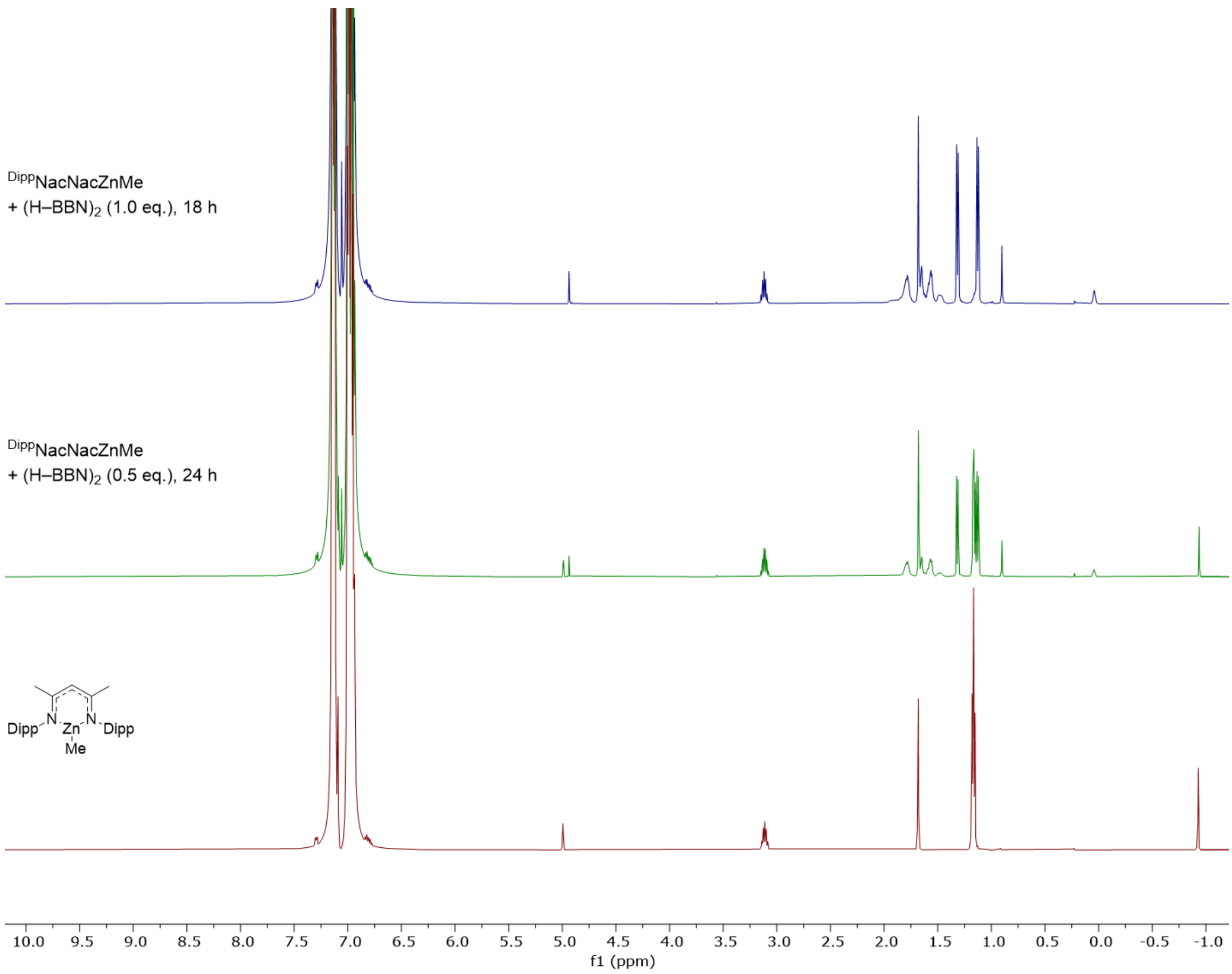

Figure S58: Stacked ^{1}H NMR spectra in PhCl of: ^{Dipp}NacNacZn−(μ-H)$_2$–BBN synthesised from ^{Dipp}NacNacZnMe and (H−BBN)$_2$ (1 eq.) (top); incomplete synthesis of ^{Dipp}NacNacZn−(μ-H)$_2$–BBN from ^{Dipp}NacNacZnMe and (H−BBN)$_2$ (0.5 eq.) due to too low loading of hydroborane (middle) and ^{Dipp}NacNacZnMe, **1-Me** (bottom).

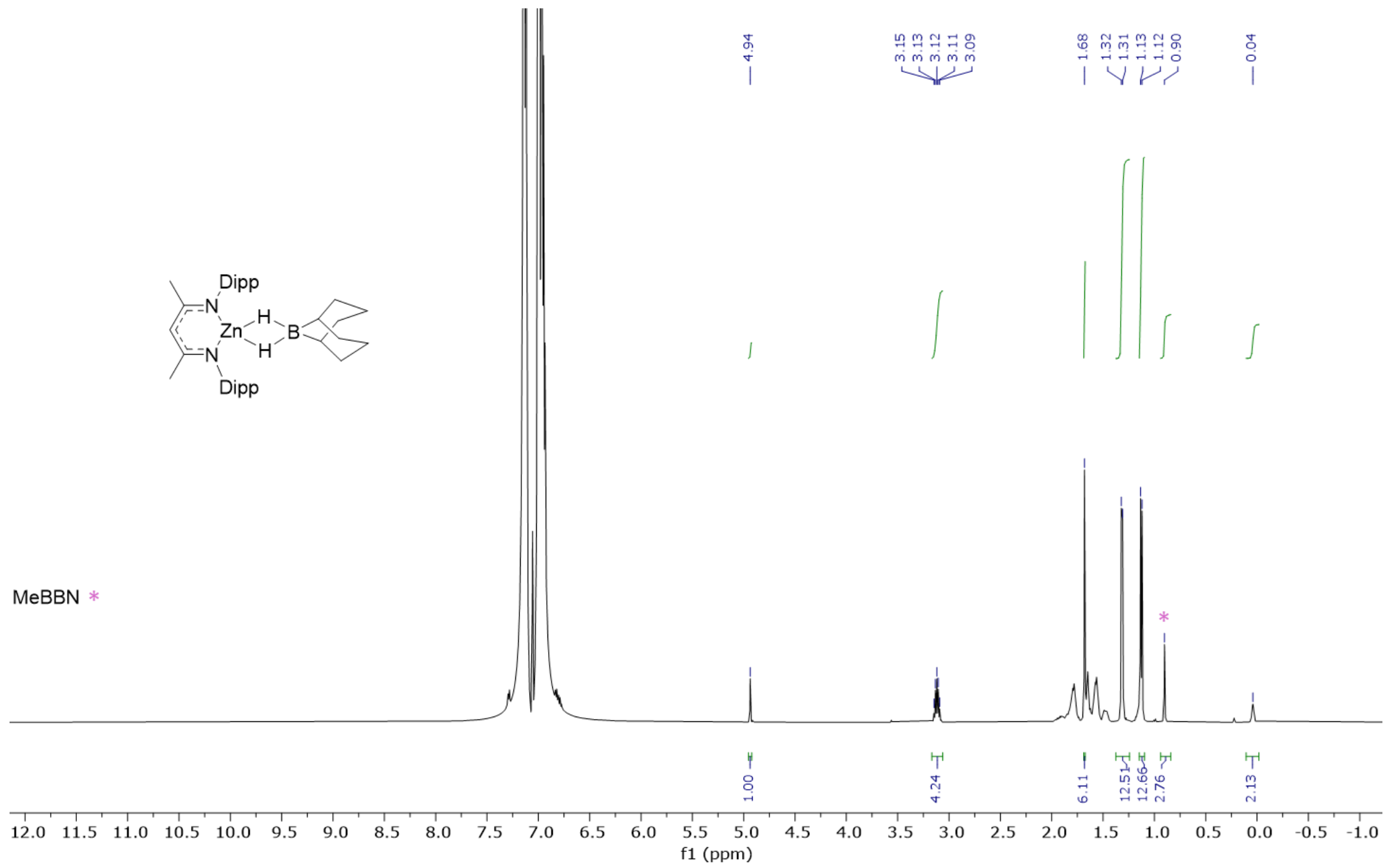

Figure S59: ^{1}H NMR spectrum of crude ^{Dipp}NacNacZn−(μ-H)$_2$–BBN in PhCl.

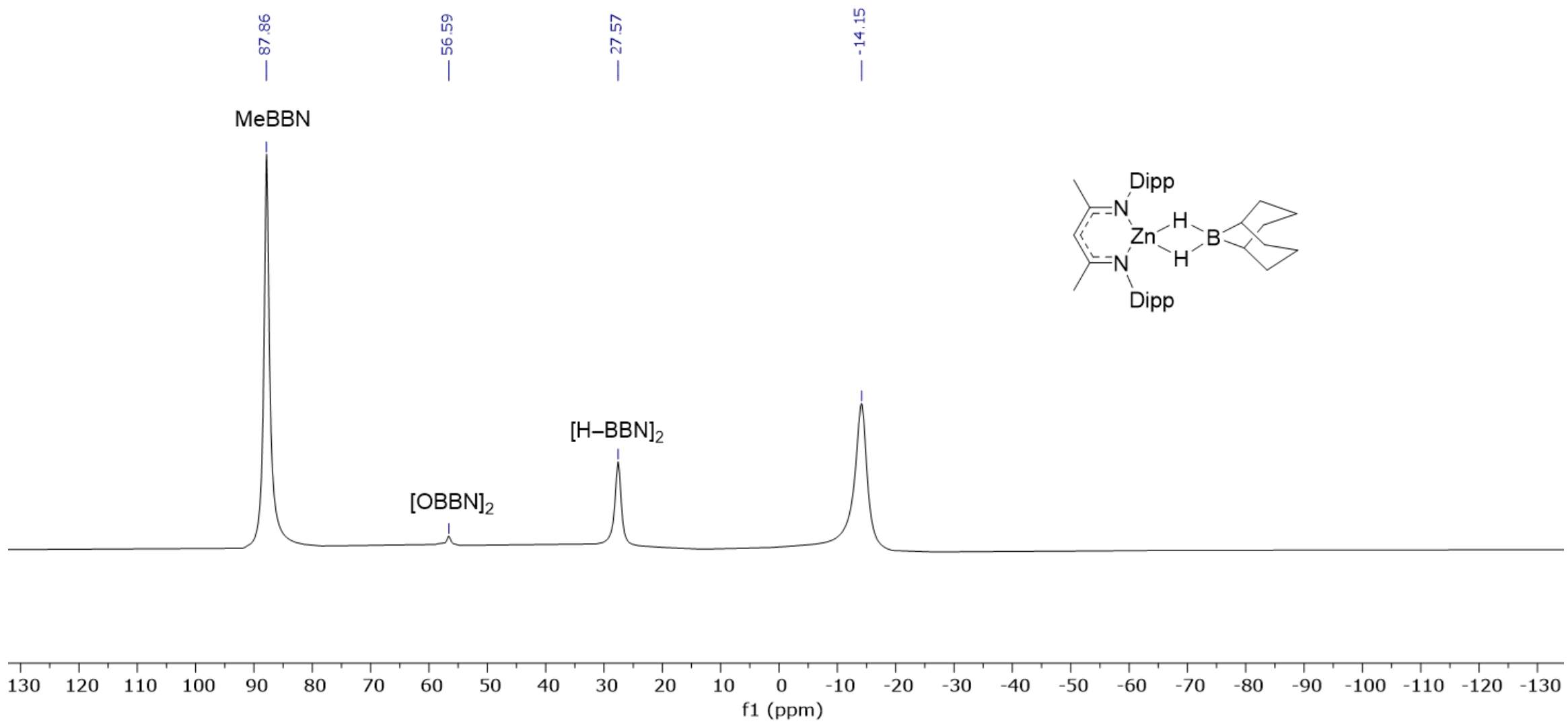

Figure S60: ^{11}B NMR spectrum of crude ^{Dipp}NacNacZn−(μ-H)$_2$–BBN in PhCl.

S5.5. MeBBN formation during C−H borylation

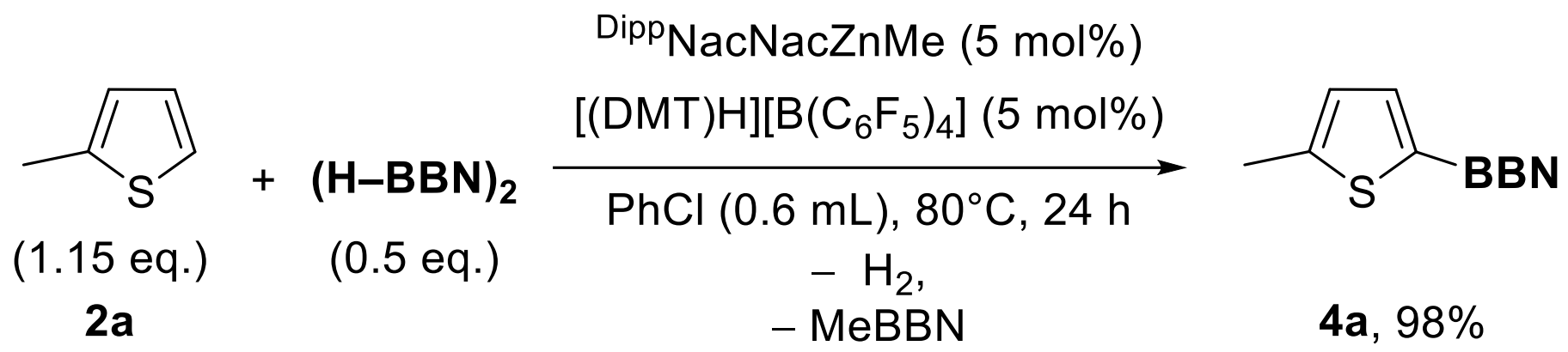

In order to understand how the catalytic cycle is initiated from ^{Dipp}NacNacZnMe, the presence of MeBBN under the optimised catalytic conditions was quantified via *in-situ* ^{1}H NMR spectroscopy of the catalytic C−H borylation of 2-methyl-thiophene (using the B–C*H*$_3$ resonance). Upon reaction completion, MeBBN (3.3% yield by ^{1}H NMR spectroscopy) was found in the crude reaction mixture.

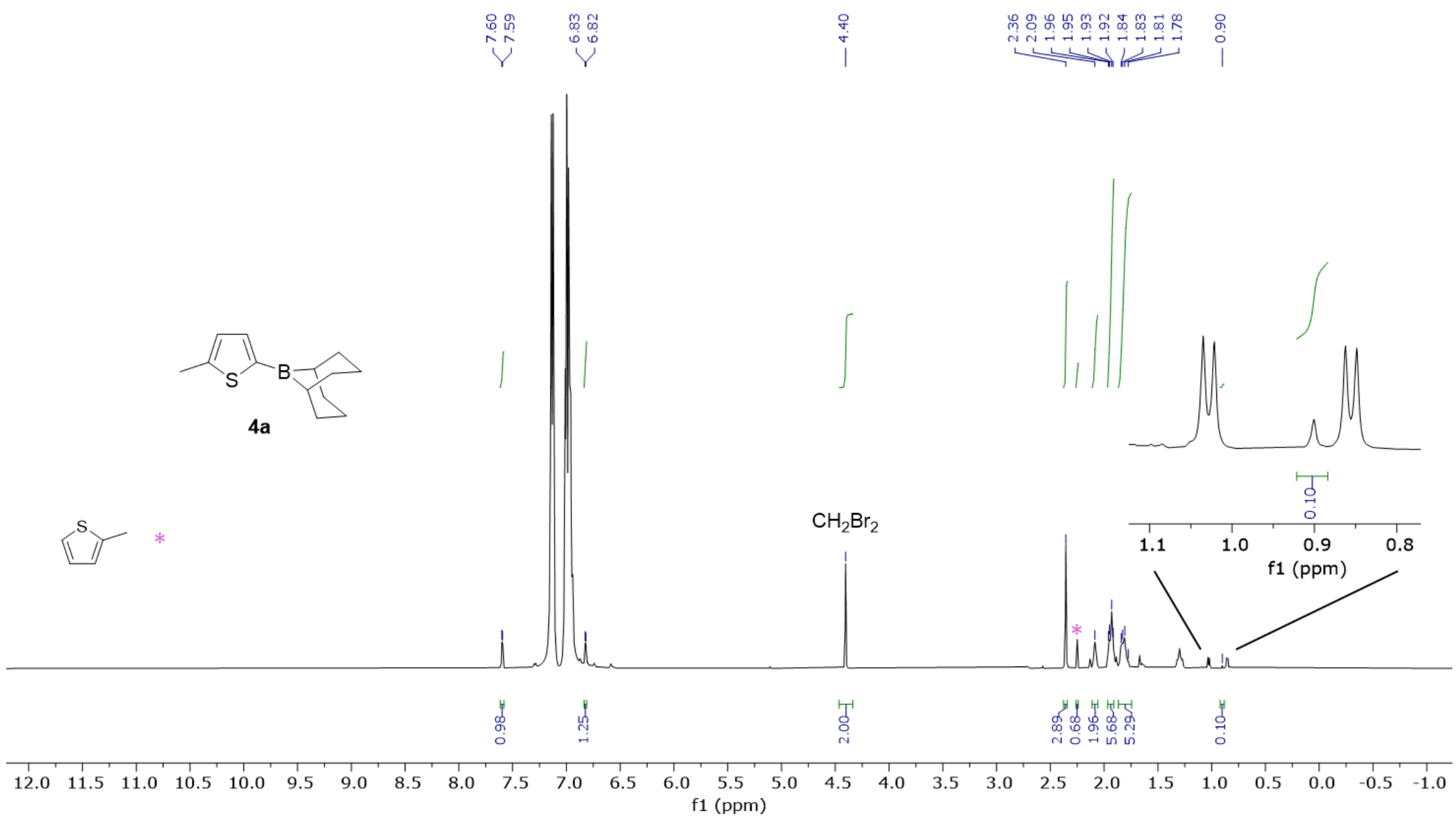

Figure S61: C–H borylation of 2-methyl-thiophene in PhCl by *in-situ* ^{1}H NMR spectroscopy. Expansion shows the B–Me region.

S6. Crystallographic data

S6.1. Crystal structure of [DippNacNacZn−$CHB_{11}H_5Br_6$], **[1−$CHB_{11}H_5Br_6$]**

CCDC Deposition Number: 2454352

Experimental. Single colourless plate-shaped crystals of [DippNacNacZn−$CHB_{11}H_5Br_6$] recrystallised from pentane by slow evaporation. A suitable crystal with dimensions 0.37 × 0.14 × 0.04 mm^3 was selected and mounted on a MITIGEN holder in Paratone oil on a Bruker D8 VENTURE diffractometer. The crystal was kept at a steady T = 100.0 K during data collection. The structure was solved with the Superflip (Palatinus & Chapuis, 2007; Palatinus & van der Lee, 2008; Palatinus et al., 2012) solution program using iterative methods and by using Olex2 1.5-beta (Dolomanov et al., 2009) as the graphical interface. The model was refined with ShelXL 2018/3 (Sheldrick, 2015) using full matrix least squares minimisation on $\boldsymbol{F}^2$.

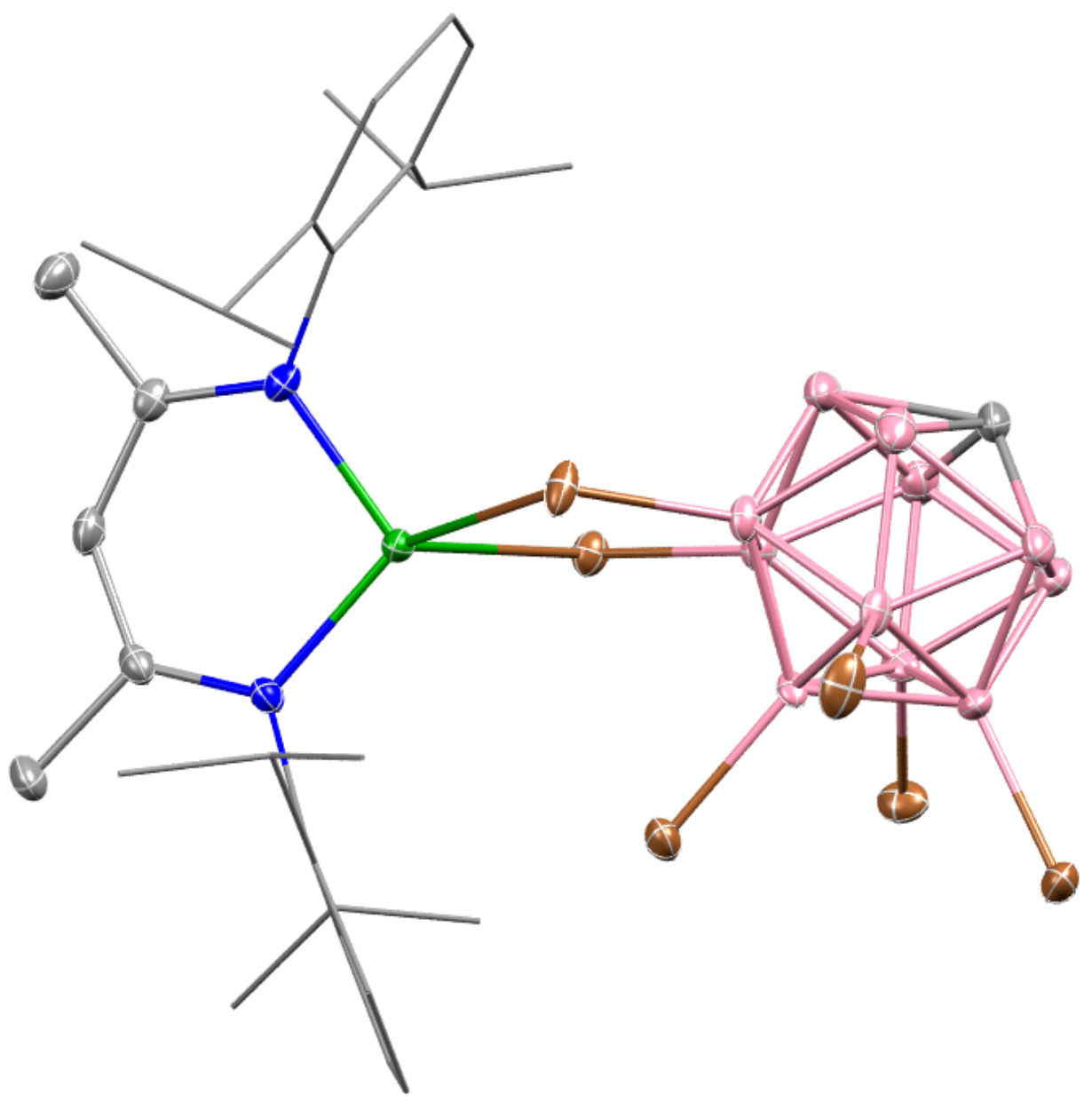

**Compound**	**[1−$CHB_{11}H_5Br_6$]**
Formula	$C_{30}H_{47}B_{11}Br_6N_2Zn$
$D_{calc.}$/ g cm^{-3}	1.700
μ/mm^{-1}	6.179
Formula Weight	1099.43
Colour	colourless
Shape	plate-shaped
Size/mm^3	0.37×0.14×0.04
T/K	100.0
Crystal System	triclinic
Space Group	P-1
a/Å	11.6147(14)
b/Å	19.821(2)
c/Å	21.632(3)
α/°	64.066(4)
β/°	76.749(4)
γ/°	75.510(4)
V/Å^3	4294.8(9)
Z	4
Z'	2
Wavelength/Å	0.71073
Radiation type	MoK$_\alpha$
Θ_{min}/°	2.126
Θ_{max}/°	28.478
Measured Refl's.	289795
Indep't Refl's	29987
Refl's I≥2σ(I)	24719
R_{int}	0.0563
Parameters	922
Restraints	0
Largest Peak	1.333
Deepest Hole	-0.721
GooF	1.022
wR_2 (all data)	0.0868
wR_2	0.0802
R_1 (all data)	0.0509
R_1	0.0347

S7. Computational details

DFT calculations were run with Gaussian 16 (Revision A.03).[12] Geometry optimizations and thermodynamic corrections were performed with the B3PW91 functional[13] with Zn and S centres described by Stuttgart RECPs and associated basis sets[14] and 6-31G** basis sets for all other atoms.[15,16] A set of d-orbital polarization functions was added to S ($\zeta^d = 0.503$).[17] This level of theory was selected based on its performance in previous studies of similar systems.[4,9] All stationary points were fully characterized via analytical frequency calculations as either minima (all positive frequencies) or transition states (one negative frequency) and the latter were characterized via IRC calculations and subsequent geometry optimizations to confirm the adjacent minima. Electronic energies were recomputed with the B3PW91 functional using def2-TZVP basis sets (BS2),[18,19] a correction for dispersion (BJD3)[20] and chlorobenzene solvent (PCM approach).[21] The thermochemical corrections from the B3PW91 frequency calculations were then added to give the free energies quoted in the text.

Additional functional testing was performed with the BP86,[22,23] BLYP,[22,24] B3LYP,[13] PBE,[25] PBE0,[26] B97D3,[27] B97D,[27] M06,[28] wB97x-D[29] and TPSS[30] functionals. Electronic energies were recomputed with each functional using def2-TZVP basis sets (BS2), a correction for dispersion (BJD3), where applicable (no dispersion was added to functionals: B97D, B97D3, M06 and wB97x-D), and chlorobenzene solvent (PCM approach). The thermochemical corrections from the B3PW91 frequency calculations were then added to give the free energies. Details of functional testing and all computed structures are provided below, the latter also as a separate XYZ file.

S7.1. Functional testing

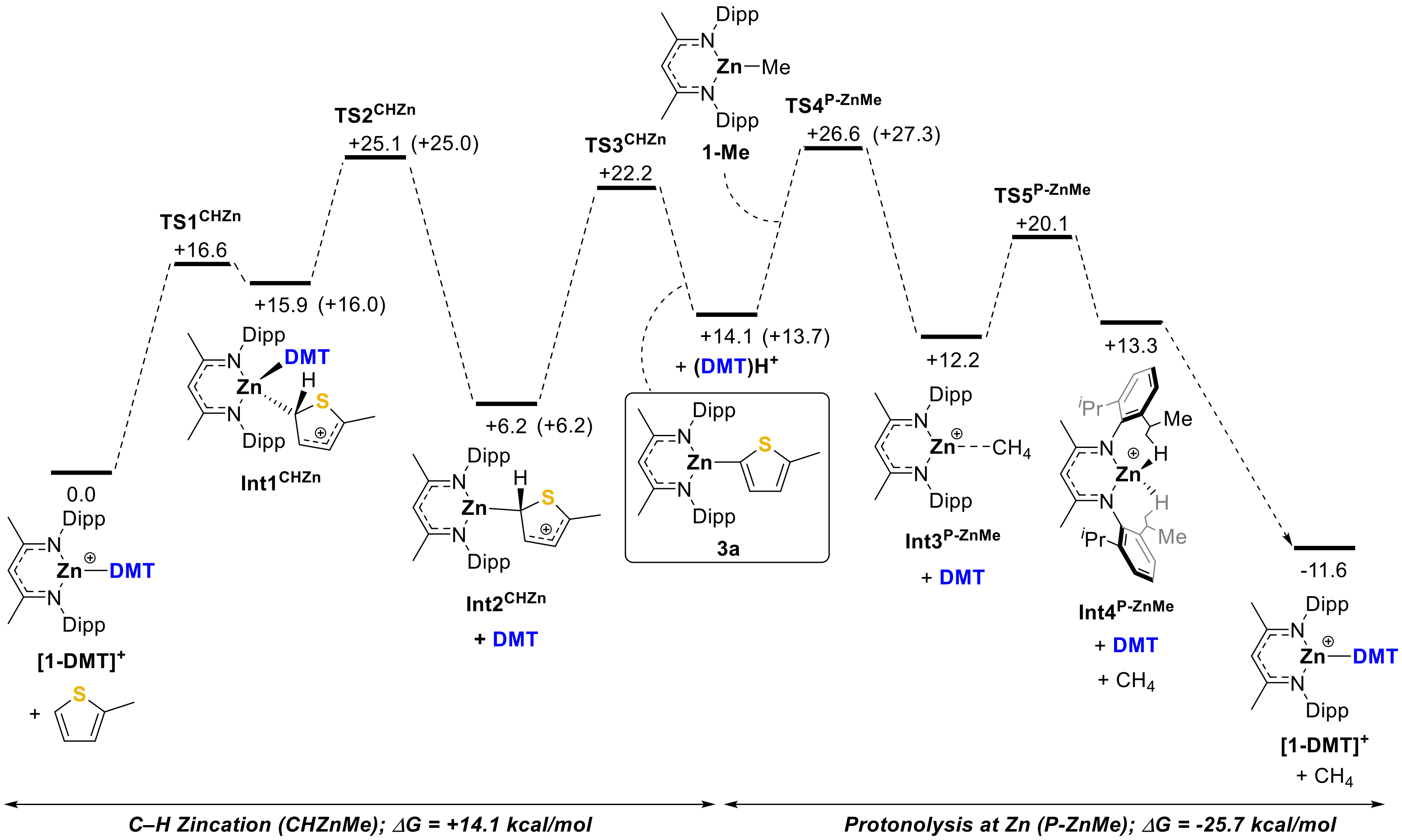

Figure S62: Computed free energy profile (kcal/mol) for the catalytic C–H zincation of 2-methyl-thiophene with ^{Dipp}NacNacZnMe at 110°C [Method: B3PW91(def2-TZVP, BJD3, PhCl)//B3PW91(Zn: SDD; S: SDD(d); other atoms: 6-31G**)]. Selected Gibbs free energies (kcal/mol) computed for 5-*d*-2-methyl-thiophene at 110°C are provided in parenthesis.

Functional testing for selected key transition states in the catalytic C–H zincation of 2-methyl-thiophene using ^{Dipp}NacNacZnMe and [DMT][B(C_6F_5)$_4$] was performed (Table S2). For majority of the screened functionals, **TS4$^{P\text{-}ZnMe}$** was consistently calculated to be higher in energy than **TS2^{CHZn}**. This suggests the relative energetic ordering is independent of the chosen functional. Therefore, in order to remain consistent, all further calculations were performed with the B3PW91 functional. This protocol was chosen based on its reliable performance in our previous studies on the related catalytic C–H zincation and C–H borylation with ^{Dipp}NacNacZnH.[4,9]

Table S2: Functional testing for the selected transition states in the catalytic C–H zincation of 2-methyl-thiophene using ^{Dipp}NacNacZnMe and [DMT][B(C_6F_5)$_4$]. Free energies quoted in the table (kcal/mol) were calculated at 110°C.

Species	BP86	BLYP	B3LYP	PBE	PBE0	B97D3	B97D	M06	WB97xD	TPSS	B3PW91
**TS2^{CHZn}**	22.5	23.5	25.3	23.3	25.3	23.6	23.9	24.5	26.0	25.3	25.1
**TS4$^{P\text{-}ZnMe}$**	23.7	26.9	29.1	21.6	25.3	25.9	29.8	31.3	31.0	26.1	26.7

S7.2. Additional energy profiles

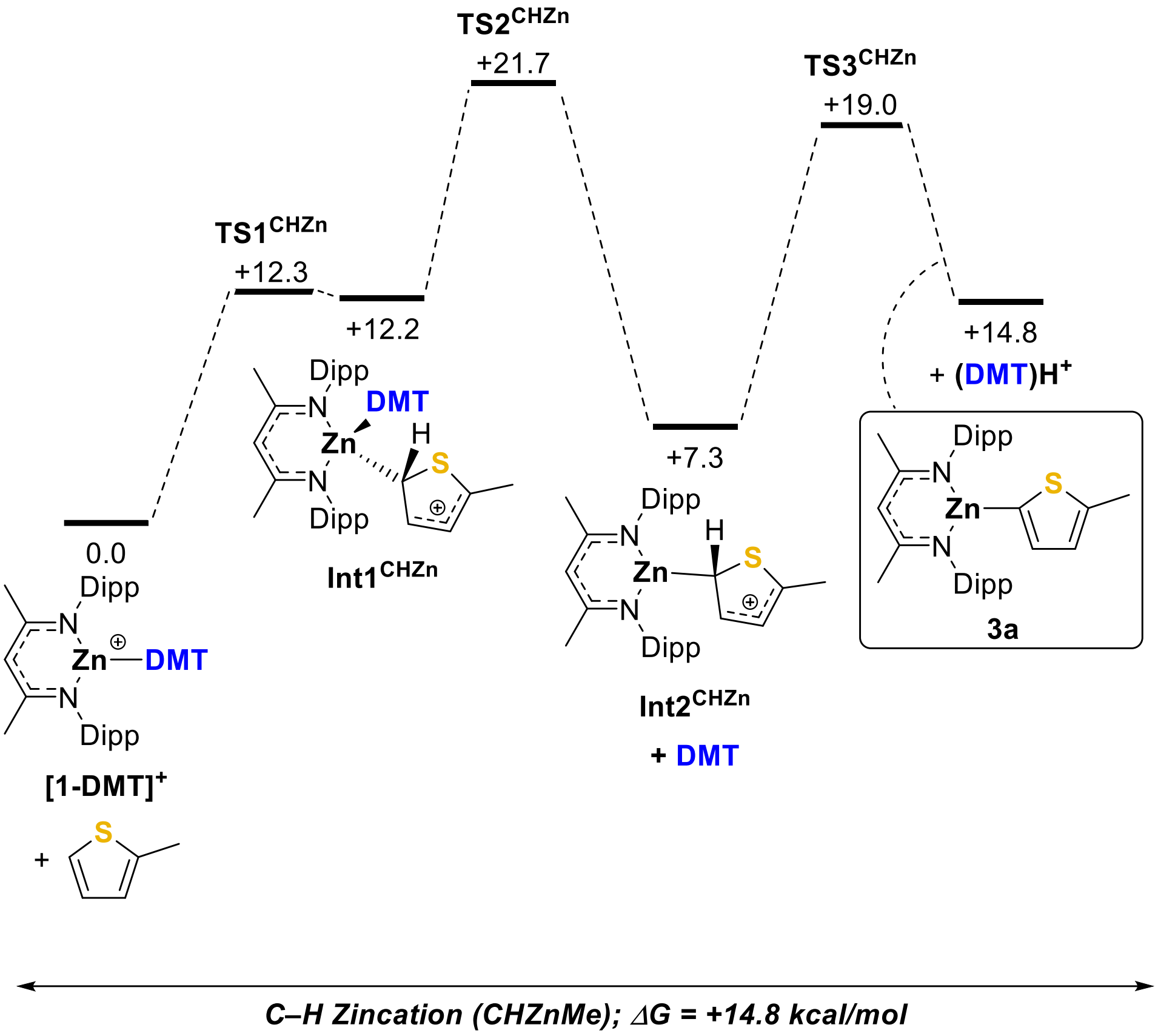

Figure S63: Computed free energy profile (kcal/mol) for the metalation phase of catalytic C–H zincation of 2-methyl-thiophene at 25°C [Method: B3PW91(def2-TZVP, BJD3, PhCl)//B3PW91(Zn: SDD; S: SDD(d); other atoms: 6-31G**)].

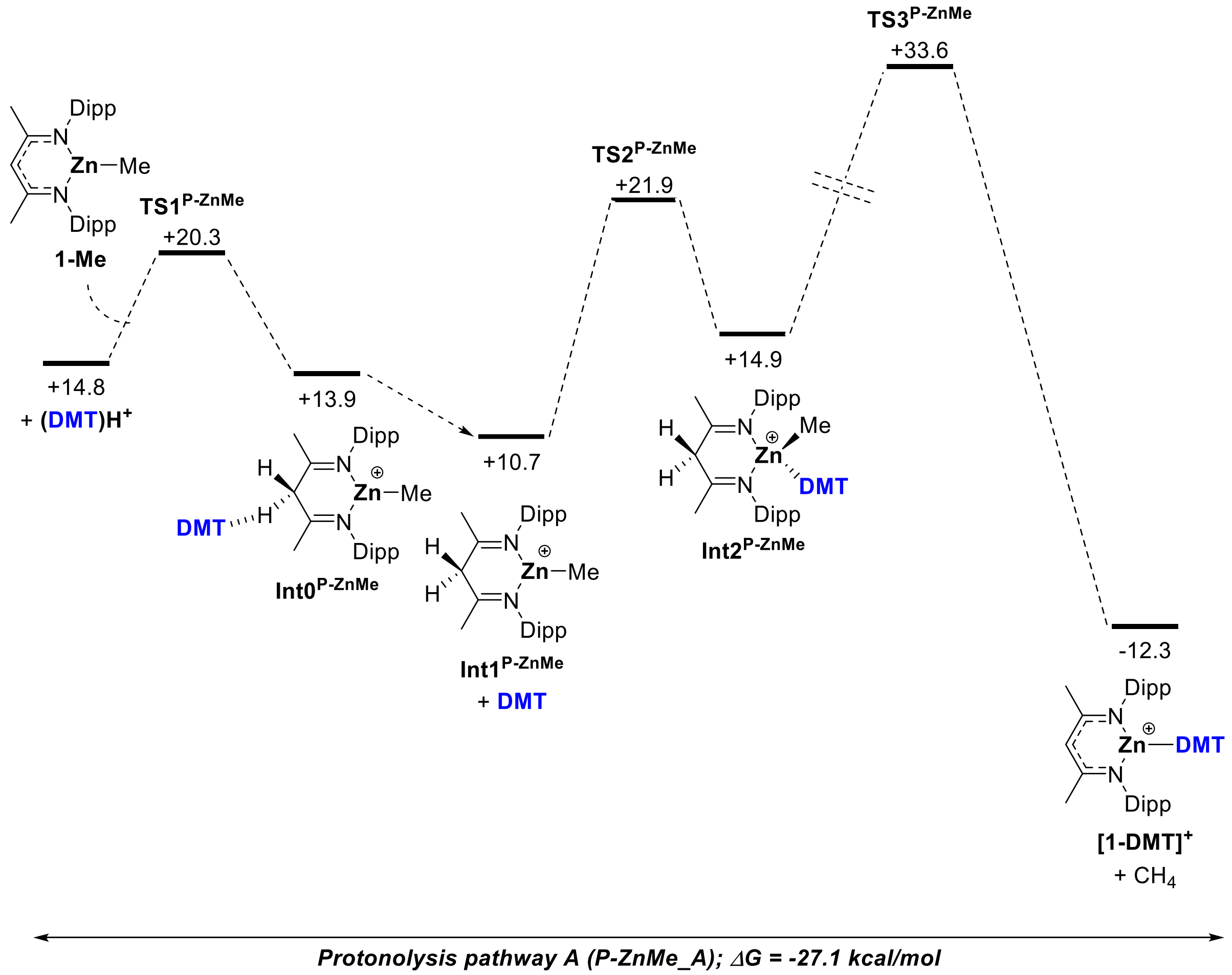

Figure S64: Computed free energy profile (kcal/mol) for the protonolysis phase (pathway A) of the C–H zincation of 2-methyl-thiophene with ^{Dipp}NacNacZnMe proceeding via a ligand assisted pathway at 25°C [Method: B3PW91(def2-TZVP, BJD3, PhCl)//B3PW91(Zn: SDD; S: SDD(d); other atoms: 6-31G**)].

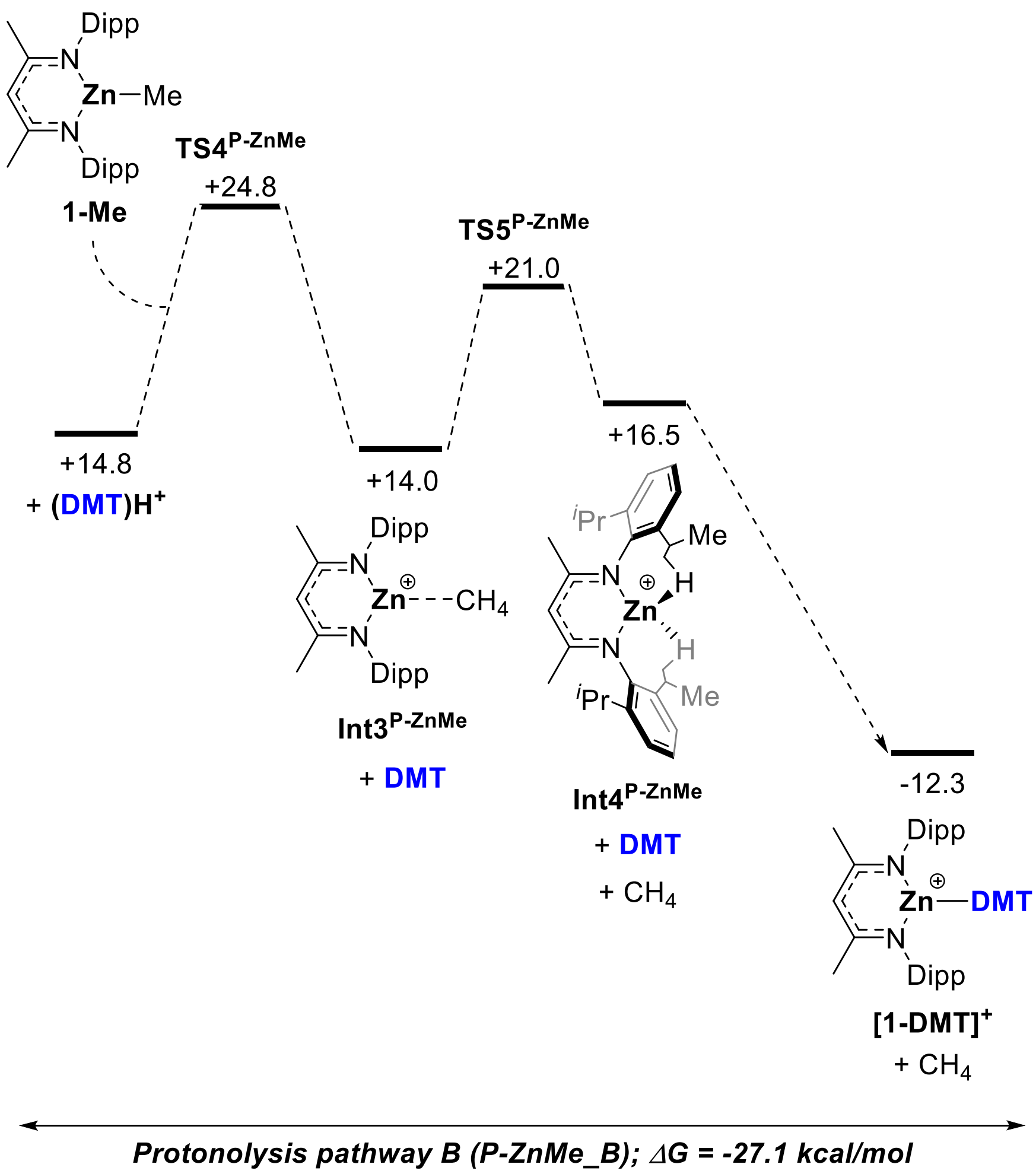

Figure S65: Computed free energy profile (kcal/mol) for the protonolysis phase (pathway B) of the C–H zincation of 2-methyl-thiophene with ^{Dipp}NacNacZnMe proceeding via σ-bond metathesis pathway at 25°C [Method: B3PW91(def2-TZVP, BJD3, PhCl)//B3PW91(Zn: SDD; S: SDD(d); other atoms: 6-31G**)].

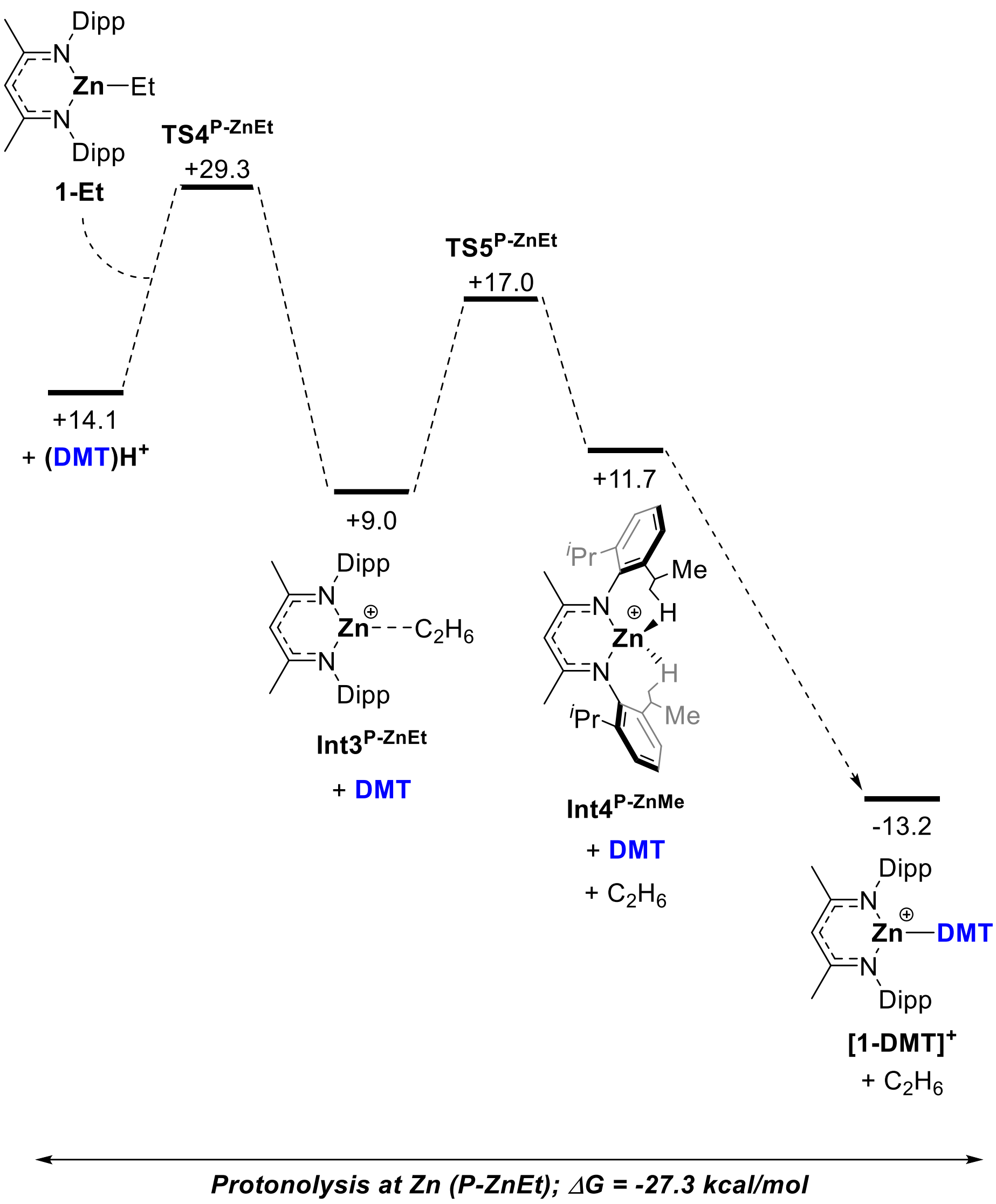

Figure S66: Computed free energy profile (kcal/mol) for the protonolysis phase of the C–H zincation of 2-methyl-thiophene with ^{Dipp}NacNacZnEt proceeding via σ-bond metathesis pathway at 110°C [Method: B3PW91(def2-TZVP, BJD3, PhCl)//B3PW91(Zn: SDD; S: SDD(d); other atoms: 6-31G**)].

S7.3. Determination of theoretical KIE for C–H zincation

S7.3.1. Theoretical KIE calculations

Based on the free energy profile computed for the C–H zincation of 2-methyl-thiophene or 5-*d*-2-methyl-thiophene with ^{Dipp}NacNacZnMe at 110°C (see Figure S62) the relevant energy barriers ($\Delta G^{\ddagger}$) were extracted and respective KIEs were calculated [equation (2)].

$$\frac{k_H}{k_D} = e^{-(\Delta G^{\ddagger}_H - \Delta G^{\ddagger}_D)/RT} \quad (2)$$

Table S3: Energy barriers (kcal/mol) and associated KIEs calculated at 110°C for selected transition states in C–H zincation of 2-methyl-thiophene or 5-*d*-2-methyl-thiophene.

**RDS**	**$\Delta G^{\ddagger}$ (kcal/mol) at 110°C**	**k_H/k_D at 110°C**
$TS2^{CHZn}$	25.1	**0.88**
d-$TS2^{CHZn}$	25.0	
$TS4^{P\text{-}ZnMe}$	26.6	**2.51**
d-$TS4^{P\text{-}ZnMe}$	27.3	

S7.3.2. Determination of equilibrium isotope effect (EIE)

Based on the free energy profile computed for the C–H zincation of 2-methyl-thiophene or 5-*d*-2-methyl-thiophene with ^{Dipp}NacNacZnMe at 110°C (see Figure S62) the relevant energy changes (ΔG) were extracted and respective EIEs, expressed as K_{eq}, were calculated [equation (3)].

$$K_{eq} = e^{-\frac{\delta \Delta G}{RT}} \quad (3)$$

Table S4: Energy changes (kcal/mol) and associated EIEs calculated at 110°C for selected intermediates in C–H zincation of 2-methyl-thiophene or 5-*d*-2-methyl-thiophene.

Intermediate	ΔG (kcal/mol) at 110°C	EIE at 110°C
$INT1^{CHZn}$	+15.9	1.14
d-$INT1^{CHZn}$	+16.0	
$INT2^{CHZn}$	+6.2	1.00
d-$INT2^{CHZn}$	+6.2	
3a + $[DMT(H)]^+$	+14.1	0.59
d-3a + $[DMT(D)]^+$	+13.7	

The product of EIEs and KIE arising from the RDS, **$TS4^{P\text{-}ZnMe}$**, gives an overall KIE of 1.69 at 110°C [equation (4)].

$$\mathrm{KIE} = 1.14 \times 1.00 \times 0.59 \times 2.51 = 1.69 \tag{4}$$

S7.4. Computed Cartesian coordinates (Å) and energies (hartrees) for all species

CH4
B3PW91
SCF = -40.5081092285
SCF (C6H5Cl) = -40.5082715566
SCF (BJD3) = -40.5099786479
SCF (BS2) = -40.5207559181
H (0 K) = -40.463014
H (298 K) = -40.459202
G (298 K) = -40.481370
G (383 K) = -40.487833
Low freq. = 1344.8935
Second freq. = 1344.8936

5
CH4
C 0.00000 0.00000 0.00000
H 0.00000 0.89122 0.63019
H -0.00000 -0.89122 0.63019
H 0.89122 -0.00000 -0.63019
H -0.89122 0.00000 -0.63019

C2H6
B3PW91
SCF = -79.8084474830
SCF (C6H5Cl) = -79.8085921023
SCF (BJD3) = -79.8137957178
SCF (BS2) = -79.8325404266
H (0 K) = -79.733377
H (298 K) = -79.728954
G (298 K) = -79.754794
G (383 K) = -79.762374
Low freq. = 311.4430
Second freq. = 825.7500

8
C2H6
C 0.00000 0.00000 0.76280
C -0.00000 -0.00000 -0.76280
H 0.00000 1.01937 1.16269
H 0.88280 -0.50968 1.16269
H -0.88280 -0.50968 1.16269
H 0.88280 0.50968 -1.16269
H -0.88280 0.50968 -1.16269
H -0.00000 -1.01937 -1.16269

DMT
B3PW91
SCF = -405.392451505
SCF (C6H5Cl) = -405.395908470
SCF (BJD3) = -405.432669780
SCF (BS2) = -405.523891386
H (0 K) = -405.191017
H (298 K) = -405.179474
G (298 K) = -405.228086
G (383 K) = -405.242695
Low freq. = 22.1259
Second freq. = 72.2345

23
DMT
H 2.48607 1.99031 -0.67122
H 3.83398 -1.05959 -0.02431
H 3.83398 1.05959 -0.02431
C 2.76194 -1.23900 0.07792
C 2.76194 1.23900 0.07792
H 2.57986 -1.67232 1.07500
H 2.57986 1.67232 1.07500
N 2.05027 -0.00000 -0.14370
H 2.48607 -1.99031 -0.67122
C -0.07402 1.20208 -0.04848
C 0.66246 0.00000 -0.07606
C -1.46406 1.18988 -0.01764
C -2.19708 0.00000 -0.00071
C -0.07402 -1.20208 -0.04848
C -1.46406 -1.18988 -0.01764
H 0.43592 -2.15865 -0.05524
H -1.99160 -2.14177 -0.00265
C -3.70149 -0.00000 0.06872
H -4.12519 0.88402 -0.41846
H -4.05966 -0.00001 1.10646
H -4.12519 -0.88401 -0.41847
H -1.99160 2.14177 -0.00265
H 0.43592 2.15865 -0.05524

[DMTH]+
B3PW91
SCF = -405.772731399
SCF (C6H5Cl) = -405.836130532
SCF (BJD3) = -405.815448175
SCF (BS2) = -405.897902913
H (0 K) = -405.556370
H (298 K) = -405.544953
G (298 K) = -405.593015
G (383 K) = -405.607479
Low freq. = 30.3062
Second freq. = 51.3724

24
[DMTH]+
H 2.21802 0.23750 2.11422
H 3.76750 0.04538 -1.22047
H 3.76460 0.02373 1.22989
C 2.70266 -0.19144 -1.24403
C 2.69970 -0.21338 1.24689
H 2.56039 -1.27087 -1.27936
H 2.55749 -1.29328 1.26295
N 2.05816 0.33939 0.00548
H 2.22286 0.27461 -2.10436
C -0.20002 1.33265 0.00450
C 0.58133 0.18431 0.00236
C -1.58660 1.20236 -0.00261
C -2.19728 -0.05738 -0.00987
C 0.01369 -1.08576 -0.00752
C -1.37305 -1.19250 -0.01447
H 0.62127 -1.98616 -0.01310
H -1.82353 -2.18080 -0.02537
C -3.69328 -0.19466 0.00920
H -4.18474 0.70560 -0.36656
H -4.04904 -0.36296 1.03242
H -4.02408 -1.04414 -0.59417
H -2.20050 2.09814 -0.00405
H 0.25028 2.32371 0.00835
H 2.22935 1.34857 0.01462

2-methyl-thiophene,2a
B3PW91
SCF = -204.285929660
SCF (C6H5Cl) = -204.288401429
SCF (BJD3) = -204.306656123
SCF (BS2) = -592.304637144
H (0 K) = -204.191145
H (298 K) = -204.184383
G (298 K) = -204.220876
G (383 K) = -204.231701
Low freq. = 95.1333
Second freq. = 230.7174

12
2-methyl-thiophene,2a
C -0.78516 0.22412 -0.00000
C -0.01658 1.35682 0.00003
C 1.38631 1.10015 -0.00003
C 1.67832 -0.23296 -0.00001
S 0.23444 -1.19357 0.00001
C -2.27587 0.10684 -0.00001
H -0.44808 2.35278 0.00004
H 2.14408 1.87607 -0.00006
H 2.64664 -0.71446 -0.00002
H -2.64577 -0.42561 0.88336
H -2.64577 -0.42560 -0.88337
H -2.72422 1.10406 0.00000

NacNacZnMe,1-Me
B3PW91
SCF = -1506.01703908
SCF (C6H5Cl) = -1506.02521306
SCF (BJD3) = -1506.20944546
SCF (BS2) = -3058.63300539
H (0 K) = -1505.343435
H (298 K) = -1505.303336
G (298 K) = -1505.416875
G (383 K) = -1505.451979
Low freq. = 17.5304
Second freq. = 19.8368

77
NacNacZnMe,1-Me
Zn 0.00021 0.03182 -0.74579
N 1.47877 0.00709 0.59201
N -1.47855 0.00752 0.59086
C -1.27239 -0.00314 1.90857
C -0.00044 -0.00396 2.50768
C 1.27203 -0.00343 1.90949
H -0.00086 -0.01131 3.59102
C -2.46183 -0.01808 2.84072
H -3.08085 -0.90355 2.66613
H -3.10555 0.84902 2.66419
H -2.14628 -0.01314 3.88503
C -2.81282 -0.00241 0.07330
C -3.43902 -1.23524 -0.22105
C -3.46014 1.22040 -0.21622
C -4.73096 1.18314 -0.79781
C -5.35745 -0.02208 -1.09122
C -4.70990 -1.21757 -0.80326

C -2.80263 2.56528 0.05764
H -5.23639 2.11750 -1.02880
H -6.34479 -0.02972 -1.54493
H -5.19893 -2.15944 -1.03882
C -2.75766 -2.56946 0.04742
C -2.41654 3.26394 -1.25488
H -1.75716 2.63526 -1.86066
H -1.89988 4.20919 -1.05379
H -3.30411 3.48898 -1.85677
C -3.67937 3.48287 0.92114
H -1.87892 2.37491 0.61345
H -4.60235 3.76630 0.40370
H -3.14240 4.40707 1.16121
H -3.96351 3.00394 1.86364
C -2.33266 -3.24181 -1.26687
H -1.80673 -4.18263 -1.06916
H -1.66865 -2.59509 -1.84830
H -3.20369 -3.46954 -1.89153
C -3.62885 -3.51772 0.88282
H -1.84815 -2.36732 0.62210
H -4.53314 -3.81987 0.34348
H -3.94489 -3.05574 1.82364
H -3.07340 -4.43033 1.12510
C 2.46088 -0.01846 2.84241
H 2.14470 -0.01403 3.88653
H 3.10446 0.84891 2.66661
H 3.08023 -0.90369 2.66776
H 3.29877 3.48598 -1.85775
H 3.14047 4.40893 1.15936
H 1.89475 4.20571 -1.05388
C 3.67852 3.48526 0.91965
C 2.41242 3.26081 -1.25408
H 3.96508 3.00809 1.86231
H 1.75303 2.63007 -1.85775
C 2.80169 2.56507 0.05908
H 1.87915 2.37441 0.61671
H 4.53179 -3.82199 0.33837
H 4.60011 3.76955 0.40020
C 3.62812 -3.52011 0.87888
H 3.94512 -3.06039 1.82049
C 2.81315 -0.00245 0.07491
C 2.75727 -2.56927 0.04610
C 3.46031 1.22059 -0.21396
C 3.43941 -1.23504 -0.22016
C 4.71061 -1.21696 -0.80168
C 4.73142 1.18379 -0.79492
C 5.35824 -0.02124 -1.08849
H 5.19975 -2.15864 -1.03772
H 5.23677 2.11830 -1.02540
H 1.84832 -2.36757 0.62183
H 6.34585 -0.02853 -1.54165
H 3.07197 -4.43272 1.11955
H 3.20126 -3.46558 -1.89500
C 2.33075 -3.23813 -1.26950
H 1.80368 -4.17871 -1.07368
H 1.66743 -2.58918 -1.84927
C 0.00706 0.06735 -2.69985
H 0.91103 -0.39645 -3.10792
H -0.02443 1.09686 -3.07467
H -0.86180 -0.45259 -3.11649

3a
B3PW91
SCF = -1669.81223588
SCF (C6H5Cl) = -1669.82254931
SCF (BJD3) = -1670.03465283
SCF (BS2) = -3610.43032961
H (0 K) = -1669.089536
H (298 K) = -1669.045077
G (298 K) = -1669.168975
G (383 K) = -1669.207334
Low freq. = 16.1431
Second freq. = 18.4574

84
3a
Zn -0.04311 0.07951 0.00003
N -1.59848 -1.13975 0.00025
N 1.36215 -1.29783 0.00015
C 1.08079 -2.60291 0.00029
C -0.22217 -3.13071 0.00042
C -1.46190 -2.46659 0.00040
H -0.27999 -4.21243 0.00054
C 2.21738 -3.59767 0.00026
H 2.85740 -3.45589 0.87635
H 2.85665 -3.45662 -0.87649
H 1.84493 -4.62296 0.00082
C 2.72496 -0.85378 0.00005
C 3.37509 -0.59944 1.22907
C 3.37501 -0.59968 -1.22906
C 4.67653 -0.09008 -1.20100
C 5.32796 0.16503 -0.00013
C 4.67661 -0.08985 1.20083
C 2.69490 -0.84027 -2.56940
H 5.18641 0.11455 -2.13890
H 6.33922 0.56263 -0.00021
H 5.18656 0.11495 2.13865
C 2.69510 -0.83983 2.56951
C 2.38128 0.48703 -3.27682
C 3.51691 -1.75880 -3.48477

H 4.46595 -1.29573 -3.77592
H 2.96249 -1.97373 -4.40479
H 3.75041 -2.71298 -3.00141
C 2.38180 0.48756 3.27691
H 1.85198 0.30654 4.21896
H 1.76153 1.13863 2.65369
H 3.30197 1.03478 3.51052
C 3.51707 -1.75846 3.48481
H 1.74191 -1.34119 2.37286
H 4.46626 -1.29557 3.77575
H 3.75027 -2.71274 3.00149
H 2.96277 -1.97320 4.40495
C -2.69907 -3.33311 0.00048
H -3.31939 -3.12372 -0.87628
H -3.31993 -3.12285 0.87664
H -2.43985 -4.39273 0.00106
H -3.27169 1.40765 -3.50512
H -3.24506 -1.60815 -4.41349
H -1.89507 0.54200 -4.20372
C -3.77558 -1.34506 -3.49191
C -2.41306 0.77020 -3.26543
H -4.10577 -2.27424 -3.01631
H -1.73625 1.35099 -2.63150
C -2.86499 -0.52284 -2.56913
H -1.96965 -1.12156 -2.37312
H -4.67142 -0.78332 3.78017
H -4.67201 -0.78504 -3.77956
C -3.77490 -1.34331 3.49277
H -4.10492 -2.27281 3.01768
C -2.90221 -0.54708 0.00019
C -2.86454 -0.52139 2.56949
C -3.51917 -0.21929 -1.22882
C -3.51896 -0.21859 1.22911
C -4.75610 0.43234 1.20093
C -4.75631 0.43166 -1.20079
C -5.37614 0.75666 0.00004
H -5.23888 0.69425 2.13900
H -5.23926 0.69304 -2.13891
H -1.96915 -1.12007 2.37364
H -6.33711 1.26394 -0.00002
H -3.24426 -1.60580 4.41445
H -3.27141 1.40949 3.50462
C -2.41272 0.77206 3.26510
H -1.89456 0.54438 4.20343
H -1.73609 1.35263 2.63079
H 3.30133 1.03440 -3.51055
H 1.76097 1.13802 -2.65356
H 1.85139 0.30589 -4.21880
H 1.74182 -1.34178 -2.37261
H 1.92490 5.92173 0.88234
S 1.42212 2.96962 -0.00024
C -0.03879 2.01108 -0.00026
C -1.11874 2.87180 -0.00054
C 1.28695 5.80256 -0.00098
C 0.57061 4.48876 -0.00078
C -0.78057 4.25541 -0.00083
H 1.92624 5.92074 -0.88346
H -2.14551 2.51777 -0.00058
H -1.51351 5.05714 -0.00111
H 0.56278 6.62229 -0.00199

NacNacZn[DMT],[1-DMT]+
B3PW91
SCF = -1871.32848514
SCF (C6H5Cl) = -1871.37991677
SCF (BJD3) = -1871.58961374
SCF (BS2) = -3424.05319612
H (0 K) = -1870.484560
H (298 K) = -1870.436322
G (298 K) = -1870.567681
G (383 K) = -1870.608505
Low freq. = 10.7559
Second freq. = 13.6557

96
NacNacZn[DMT],[1-DMT]+
Zn -0.28248 -0.22290 -0.17866
N -2.00247 -0.56926 0.62904
N 0.92414 -1.40113 0.74696
C 0.40712 -2.18597 1.70724
C -0.94913 -2.21747 2.07160
C -2.05125 -1.47303 1.62069
H -1.17810 -2.91352 2.87002
C 1.31798 -3.08428 2.50923
H 2.21928 -3.36244 1.96275
H 0.78759 -3.98879 2.81357
H 1.63340 -2.56792 3.42264
C 2.33949 -1.39225 0.49832
C 3.19042 -0.63428 1.33317
C 2.84079 -2.09918 -0.62197
C 4.21211 -2.02190 -0.88732
C 5.06341 -1.27336 -0.08038
C 4.55253 -0.59053 1.01592
C 1.93797 -2.95656 -1.50214
H 4.62437 -2.55962 -1.73495
H 6.12574 -1.23184 -0.30316
H 5.22488 -0.01366 1.64460
C 2.68583 0.13617 2.54712
C 2.40776 -3.04796 -2.95944
H 2.64482 -2.06923 -3.39002

H 1.63065 -3.51209 -3.57455
H 3.30170 -3.67211 -3.05754
C 1.76769 -4.37385 -0.92980
H 0.94024 -2.49414 -1.50020
H 2.73884 -4.87282 -0.84591
H 1.13483 -4.97814 -1.58812
H 1.30533 -4.36583 0.05957
C 2.69504 1.65194 2.29892
H 2.30718 2.18351 3.17431
H 2.08670 1.92937 1.43273
H 3.71167 2.01395 2.11323
C 3.48222 -0.19867 3.81738
H 1.64599 -0.15492 2.72640
H 4.51115 0.17066 3.75938
H 3.53065 -1.27636 3.99962
H 3.01844 0.27366 4.68924
C -3.34794 -1.70123 2.35792
H -3.39330 -2.71772 2.75283
H -4.22193 -1.51747 1.73256
H -3.40821 -1.01471 3.21041
H -4.72356 -1.41884 -3.45936
H -4.15885 -3.71837 -1.63606
H -3.28440 -2.41754 -3.60281
C -4.42697 -2.79767 -1.10748
C -3.68342 -1.51314 -3.13246
H -4.29619 -2.97744 -0.03769
H -3.13761 -0.65310 -3.53560
C -3.57041 -1.62137 -1.60520
H -2.52627 -1.85899 -1.36335
H -4.39295 2.95831 3.12022
H -5.48978 -2.60404 -1.28725
C -3.71930 2.11759 3.31441
H -4.33906 1.24805 3.55236
C -3.18728 0.14139 0.23166
C -2.78919 1.84539 2.12273
C -3.92670 -0.32803 -0.88053
C -3.55104 1.32591 0.90956
C -4.65727 2.04157 0.43775
C -5.03329 0.41923 -1.29703
C -5.39471 1.59745 -0.65280
H -4.95186 2.95804 0.94138
H -5.62357 0.07408 -2.14056
H -2.07880 1.07200 2.43252
H -6.25554 2.16369 -0.99618
H -3.13217 2.37075 4.20294
H -2.62085 3.90952 1.42280
C -1.97428 3.10335 1.78579
H -1.44898 3.47165 2.67304
H -1.21810 2.90859 1.01651
H -1.64053 2.27728 -1.18668
C -1.30575 1.72289 -2.06263
H -1.19847 2.40351 -2.91434
H -2.06402 0.97529 -2.29089
H 4.82683 4.98932 -1.95090
C 4.39765 4.72222 -0.97797
C 2.40300 1.51036 -1.49012
C 1.08822 1.98412 -1.54758
C 3.45801 2.39505 -1.29983
C 0.85547 3.35116 -1.38313
C 3.24609 3.77052 -1.14808
C 1.92568 4.22252 -1.18021
N -0.01560 1.03220 -1.79538
H 2.62629 0.45195 -1.57713
H 4.46789 1.99693 -1.26112
H -0.14324 3.76765 -1.41565
H 1.71728 5.28177 -1.05565
H 4.08427 5.64988 -0.49248
H 5.19969 4.27863 -0.38123
C 0.28554 0.19336 -2.99479
H -0.54553 -0.49495 -3.16523
H 0.41008 0.83681 -3.87248
H 1.19539 -0.38276 -2.84434

TS1[CHZn]
B3PW91
SCF = -2075.60146632
SCF (C6H5Cl) = -2075.64714236
SCF (BJD3) = -2075.91311696
SCF (BS2) = -4016.34101563
H (0 K) = -2074.661876
H (298 K) = -2074.607018
G (298 K) = -2074.751179
G (383 K) = -2074.796124
Low freq. = -25.4978
Second freq. = 15.6725

108
TS1[CHZn]
Zn 0.12277 -0.01632 0.01204
N 1.53591 -1.10382 -0.85209
N -1.15589 0.34513 -1.42642
C -0.61467 0.27485 -2.65718
C 0.64722 -0.26140 -2.95595
C 1.56096 -1.01085 -2.18536
H 0.89861 -0.22214 -4.01073
C -1.36180 0.81262 -3.85980
H -0.83474 1.69890 -4.23013
H -2.39033 1.09100 -3.63732
H -1.35792 0.08503 -4.67448
C -2.56956 0.59591 -1.29127

C	-3.03612	1.88675	-0.94505
C	-3.48310	-0.46814	-1.51339
C	-4.85224	-0.20027	-1.40102
C	-5.32201	1.06514	-1.07675
C	-4.41603	2.09275	-0.84798
C	-3.05578	-1.87806	-1.91358
H	-5.56409	-1.00044	-1.58050
H	-6.38975	1.25091	-1.00553
H	-4.78885	3.08125	-0.59825
C	-2.10064	3.06728	-0.72629
C	-3.75578	-2.97475	-1.09322
C	-3.31833	-2.14346	-3.40627
H	-4.39015	-2.08462	-3.62375
H	-2.97754	-3.14709	-3.68111
H	-2.81160	-1.42626	-4.05364
C	-2.54296	3.97492	0.43162
H	-1.73941	4.67083	0.69393
H	-2.81766	3.40679	1.32612
H	-3.41007	4.58392	0.15688
C	-1.94509	3.91552	-1.99913
H	-1.10921	2.66516	-0.48536
H	-2.91291	4.32341	-2.30973
H	-1.54574	3.33615	-2.83368
H	-1.26835	4.75720	-1.81749
C	2.61597	-1.72846	-2.99528
H	2.15340	-2.23387	-3.84676
H	3.17170	-2.45876	-2.40875
H	3.32443	-0.99940	-3.40183
H	0.67753	-5.73252	0.92102
H	-0.33583	-4.78653	-1.61788
H	-0.90928	-4.98042	0.83422
C	0.65122	-4.36661	-1.39727
C	0.14999	-4.77785	1.01780
H	0.88479	-3.63688	-2.17488
H	0.25654	-4.44795	2.05543
C	0.66863	-3.74537	0.00994
H	-0.03651	-2.90268	-0.00309
H	6.41369	-0.93376	-0.93447
H	1.38358	-5.17800	-1.46422
C	5.55248	-0.52598	-1.47379
H	5.27238	-1.25547	-2.23678
C	2.48325	-1.93155	-0.14838
C	4.39869	-0.20715	-0.50500
C	2.04532	-3.18628	0.34781
C	3.80854	-1.48560	0.08307
C	4.65108	-2.29427	0.85707
C	2.93396	-3.94940	1.10988
C	4.22450	-3.50698	1.37666
H	5.66953	-1.96555	1.04400
H	2.61586	-4.91200	1.49582
H	3.61254	0.30916	-1.06577
H	4.89842	-4.11523	1.97282
H	5.88644	0.38607	-1.97962
H	5.71259	0.30873	1.16568
C	4.91472	0.76248	0.56886
H	5.32908	1.65807	0.09538
H	4.12799	1.08931	1.25195
H	-4.82106	-3.03880	-1.33612
H	-3.67705	-2.81748	-0.01445
H	-3.31871	-3.95075	-1.32817
H	-1.97526	-1.96551	-1.74681
H	-3.99954	1.16435	4.66630
S	-3.37372	-0.42521	2.10250
C	-2.00076	-1.40575	1.69355
C	-1.34871	-1.84201	2.82282
C	-3.92919	0.08268	4.82148
C	-3.04985	-0.57434	3.80526
C	-1.93651	-1.35249	4.02083
H	-1.86936	-1.73617	0.67128
H	-4.94704	-0.32006	4.79066
H	-0.50225	-2.51842	2.79717
H	-1.56633	-1.58167	5.01453
H	-3.53153	-0.08965	5.82451
H	2.05869	3.06508	3.06472
C	2.15643	3.19289	1.99089
H	-1.00353	2.30922	1.78128
C	-0.38363	1.67365	2.41017
H	-0.02595	2.25715	3.26472
C	1.55967	2.29168	1.10275
N	0.76556	1.15866	1.61563
C	1.59061	0.28303	2.49642
H	1.97308	0.84517	3.35430
H	0.96072	-0.52838	2.86511
H	2.42489	-0.13353	1.93333
H	-0.96871	0.83197	2.77944
H	3.94253	5.64253	-1.46007
H	3.34364	4.95815	2.21646
C	2.88767	4.27111	1.50826
C	3.83155	5.66933	-0.37354
H	4.83283	5.69806	0.06833
C	3.04728	4.49015	0.13261
H	3.33680	6.61099	-0.11097
C	2.44713	3.58150	-0.74084
C	1.71122	2.49479	-0.26565
H	2.55112	3.71813	-1.81371
H	1.26830	1.81852	-0.99507

INT1[CHZn]
B3PW91
SCF = -2075.60194019
SCF (C6H5Cl) = -2075.64450393
SCF (BJD3) = -2075.91594219
SCF (BS2) = -4016.34068856
H (0 K) = -2074.661969
H (298 K) = -2074.606347
G (298 K) = -2074.752979
G (383 K) = -2074.798660
Low freq. = 11.2828
Second freq. = 21.1629

108
INT1[CHZn]
Zn 0.04581 -0.00374 0.19134
N 1.49336 -0.92851 -0.84824
N -1.32188 0.36152 -1.20757
C -0.84940 0.39215 -2.46157
C 0.44854 0.00986 -2.84251
C 1.44090 -0.73456 -2.17017
H 0.64353 0.13269 -3.90313
C -1.71954 0.85598 -3.61046
H -1.27568 1.75557 -4.05031
H -2.74086 1.08270 -3.30882
H -1.74289 0.10140 -4.40061
C -2.74123 0.45170 -0.97605
C -3.31932 1.67040 -0.54086
C -3.55186 -0.69820 -1.16921
C -4.92135 -0.60272 -0.89521
C -5.49365 0.58065 -0.45077
C -4.69367 1.70399 -0.28400
C -3.02746 -2.01920 -1.72459
H -5.55306 -1.47313 -1.04717
H -6.55989 0.63279 -0.25048
H -5.14974 2.63363 0.04172
C -2.51865 2.95855 -0.40910
C -3.43467 -3.23525 -0.87703
C -3.51321 -2.24187 -3.16799
H -4.60232 -2.35361 -3.19489
H -3.07545 -3.15578 -3.58305
H -3.25370 -1.41126 -3.82686
C -2.95977 3.82476 0.78112
H -2.22013 4.60926 0.97133
H -3.09411 3.24390 1.69941
H -3.90925 4.33060 0.57862
C -2.59337 3.80609 -1.69066
H -1.46648 2.68338 -0.27061
H -3.63066 4.08109 -1.90991
H -2.19765 3.27723 -2.55918
H -2.01840 4.73020 -1.56934
C 2.46430 -1.35134 -3.09846
H 1.96286 -2.03526 -3.78950
H 3.24872 -1.89623 -2.57628
H 2.92020 -0.56938 -3.71326
H 0.79013 -5.66594 0.49637
H 0.26059 -4.99863 -2.41462
H -0.76145 -5.12037 -0.12123
C 1.18432 -4.59716 -1.98467
C 0.18727 -4.77386 0.30038
H 1.66489 -3.96978 -2.73764
H -0.02690 -4.30277 1.26253
C 0.88571 -3.82632 -0.68619
H 0.19059 -3.01656 -0.93833
H 5.96965 -0.41615 -1.30938
H 1.85323 -5.44088 -1.78433
C 5.08529 0.22572 -1.38434
H 4.54849 -0.05179 -2.29122
C 2.48046 -1.82339 -0.28832
C 4.22068 0.10147 -0.11668
C 2.16045 -3.19723 -0.13284
C 3.75156 -1.33339 0.11488
C 4.64961 -2.22827 0.70808
C 3.09897 -4.03938 0.47435
C 4.33111 -3.56531 0.90192
H 5.62541 -1.86730 1.01813
H 2.86518 -5.09303 0.59478
H 3.33379 0.72856 -0.26150
H 5.04658 -4.23753 1.36648
H 5.43086 1.25809 -1.50300
H 6.05289 0.27125 1.06929
C 5.03469 0.67335 1.05863
H 5.12300 1.75917 0.95529
H 4.59537 0.45948 2.03680
H -4.51684 -3.39680 -0.90191
H -3.14641 -3.13930 0.17324
H -2.96884 -4.14214 -1.27594
H -1.93237 -1.96989 -1.74727
H -3.17887 0.35419 4.98370
S -2.67382 -0.99089 2.24758
C -1.18022 -1.60070 1.57775
C -0.29250 -1.93016 2.59114
C -2.90335 -0.70425 5.03803
C -2.07288 -1.12337 3.86730
C -0.78756 -1.62686 3.88030
H -1.19783 -2.02802 0.58020
H -3.83179 -1.28149 5.09485
H 0.66641 -2.40220 2.40141
H -0.23288 -1.79204 4.79777
H -2.34949 -0.86361 5.96593

H 1.80338 3.69544 2.72085
C 1.83253 3.70424 1.63526
H -1.13593 2.40080 1.86081
C -0.37981 1.89406 2.45496
H -0.02776 2.57342 3.23880
C 1.32766 2.63048 0.89335
N 0.74045 1.46436 1.57928
C 1.76177 0.80076 2.43095
H 2.20809 1.51181 3.13384
H 1.28186 0.00312 2.99862
H 2.54155 0.37622 1.80456
H -0.81461 1.01371 2.92904
H 3.13858 5.91890 -2.16910
H 2.76986 5.62724 1.58736
C 2.38370 4.80474 0.99061
C 3.04083 6.07670 -1.09240
H 4.03230 6.31461 -0.69380
C 2.45218 4.87369 -0.40798
H 2.41212 6.96126 -0.94042
C 1.94820 3.79289 -1.13333
C 1.39085 2.68320 -0.49562
H 1.98815 3.80877 -2.21911
H 1.01867 1.86919 -1.11325

TS2[CHZn]
B3PW91
SCF = -2075.58656011
SCF (C6H5Cl) = -2075.63541790
SCF (BJD3) = -2075.88918829
SCF (BS2) = -4016.32612414
H (0 K) = -2074.648187
H (298 K) = -2074.592674
G (298 K) = -2074.742168
G (383 K) = -2074.788406
Low freq. = -52.0909
Second freq. = 11.2190

108
TS2[CHZn]
Zn 0.41314 -0.17774 0.27886
N 1.77757 0.62855 -0.87104
N -0.84379 -1.08609 -0.88724
C -0.79896 -0.64747 -2.15579
C 0.17288 0.23274 -2.67019
C 1.40579 0.68973 -2.16046
H 0.02544 0.47429 -3.71776
C -1.84367 -1.09713 -3.15001
H -2.55716 -0.27722 -3.29188
H -2.39566 -1.97296 -2.81132
H -1.39494 -1.31009 -4.12261
C -1.58500 -2.27901 -0.54764
C -2.86247 -2.20267 0.05525
C -0.99533 -3.54166 -0.81538
C -1.70128 -4.69870 -0.47053
C -2.95310 -4.63531 0.12491
C -3.52007 -3.39486 0.37942
C 0.35143 -3.71076 -1.50527
H -1.26423 -5.66955 -0.68489
H -3.48772 -5.54617 0.37816
H -4.50815 -3.34736 0.82673
C -3.59043 -0.89241 0.30016
C 1.28446 -4.64876 -0.72681
C 0.17962 -4.22429 -2.94444
H -0.28133 -5.21764 -2.95186
H 1.15099 -4.30355 -3.44378
H -0.45382 -3.56296 -3.54078
C -4.20917 -0.82238 1.70318
H -4.63627 0.17060 1.87548
H -3.47315 -1.03037 2.48701
H -5.02637 -1.54035 1.82419
C -4.69053 -0.67230 -0.75144
H -2.86721 -0.07614 0.19994
H -5.45866 -1.44999 -0.67676
H -4.29390 -0.69619 -1.76866
H -5.17044 0.29916 -0.60110
C 2.33560 1.26192 -3.20710
H 2.45148 0.55254 -4.03097
H 3.32073 1.50638 -2.81384
H 1.89220 2.16927 -3.63042
H 5.35750 -2.64820 0.27891
H 3.92091 -3.00265 -2.36674
H 3.88682 -3.59386 0.12518
C 4.19472 -1.97080 -2.12328
C 4.26592 -2.58721 0.32005
H 3.76441 -1.31713 -2.88654
H 3.99518 -2.31196 1.34497
C 3.71090 -1.59556 -0.71318
H 2.61752 -1.68360 -0.71610
H 4.00960 4.78892 -1.34770
H 5.28458 -1.88853 -2.19155
C 3.00147 4.37801 -1.46602
H 2.96815 3.86062 -2.42695
C 3.14191 0.91858 -0.48761
C 2.64414 3.45442 -0.28960
C 4.06675 -0.15257 -0.38643
C 3.56017 2.24357 -0.21101
C 4.89711 2.46532 0.13928
C 5.38821 0.12993 -0.02452
C 5.81052 1.42635 0.23327
H 5.22780 3.48096 0.33536

H 6.10491 -0.68272 0.04032
H 1.62456 3.08702 -0.44412
H 6.84415 1.62470 0.50094
H 2.30484 5.22170 -1.51293
H 3.64947 4.75362 1.15300
C 2.68006 4.26458 1.01606
H 1.92406 5.05603 1.00100
H 2.50539 3.63628 1.89459
H 0.89783 -5.67187 -0.69478
H 1.42541 -4.32400 0.30972
H 2.26588 -4.69457 -1.20903
H 0.83361 -2.72910 -1.56341
H -1.66485 -0.09732 5.29901
S -0.25802 -1.76096 3.09651
C 1.06533 -1.27476 2.05081
C 1.82539 -0.27516 2.67769
C -0.64299 -0.41674 5.52904
C 0.16848 -0.58775 4.28656
C 1.28863 0.13592 3.90828
H 1.48012 -2.03276 1.38766
H -0.71091 -1.35304 6.09166
H 2.74635 0.10877 2.24832
H 1.71209 0.92153 4.52393
H -0.18721 0.33660 6.17493
H -4.11996 2.88178 1.70642
C -3.80577 2.90028 0.66836
H -2.63679 1.10328 2.53062
C -1.94845 1.94552 2.59872
H -2.45721 2.77939 3.10988
C -2.48565 2.61179 0.31343
N -1.45483 2.30869 1.27327
C -0.54665 3.45383 1.41522
H -1.05351 4.31603 1.87808
H 0.30101 3.17104 2.04329
H -0.17486 3.76378 0.43984
H -1.09516 1.65553 3.21665
H -6.42220 3.72623 -2.30398
H -5.76439 3.45847 -0.00518
C -4.74504 3.23460 -0.31043
C -5.40826 3.69672 -2.71082
H -5.40413 2.99643 -3.55264
C -4.40317 3.30482 -1.66142
H -5.18643 4.69021 -3.11755
C -3.07673 3.00630 -2.00685
C -2.13793 2.66189 -1.04349
H -2.77569 3.04179 -3.05155
H -1.12200 2.41709 -1.34327

INT2[CHZn]
B3PW91
SCF = -1670.21252981
SCF (C6H5Cl) = -1670.26421089
SCF (BJD3) = -1670.44149696
SCF (BS2) = -3610.82592447
H (0 K) = -1669.477997
H (298 K) = -1669.433243
G (298 K) = -1669.558806
G (383 K) = -1669.597660
Low freq. = 9.7570
Second freq. = 17.1821

85
INT2[CHZn]
Zn 0.07322 0.15342 -0.16610
N 1.65716 0.54262 0.83820
N -1.37567 0.65425 0.97697
C -1.06281 1.19790 2.16431
C 0.25073 1.41092 2.62088
C 1.49875 1.10745 2.04495
H 0.31295 1.87176 3.59908
C -2.17954 1.59410 3.09574
H -2.61627 0.70354 3.56104
H -2.98696 2.09801 2.56015
H -1.81516 2.24582 3.89043
C -2.74023 0.44178 0.58132
C -3.39443 -0.75094 0.96457
C -3.37431 1.39245 -0.25080
C -4.67014 1.11520 -0.69999
C -5.32178 -0.06043 -0.34736
C -4.68567 -0.97967 0.47972
C -2.71618 2.70777 -0.64602
H -5.17997 1.83785 -1.33127
H -6.32844 -0.25466 -0.70597
H -5.20637 -1.89058 0.76131
C -2.73509 -1.78864 1.86346
C -2.63223 2.88087 -2.16981
C -3.44039 3.91014 -0.01925
H -4.47113 3.98380 -0.38111
H -2.92918 4.84309 -0.27813
H -3.47718 3.84004 1.07158
C -2.28295 -3.01480 1.05597
H -1.79116 -3.74753 1.70427
H -1.57614 -2.73551 0.26581
H -3.13855 -3.50699 0.58050
C -3.63778 -2.21761 3.02865
H -1.83576 -1.33881 2.29639
H -4.51415 -2.77511 2.68302
H -3.99966 -1.35694 3.59923

H -3.08741 -2.87088 3.71318
C 2.71938 1.42374 2.87055
H 3.54641 1.77567 2.25094
H 3.06672 0.52112 3.38561
H 2.49342 2.17430 3.62908
H 3.72721 2.69857 -3.00304
H 3.28148 4.58857 -0.62963
H 2.22999 3.57807 -2.70523
C 3.75457 3.62986 -0.39332
C 2.76228 2.64612 -2.48912
H 3.84585 3.56004 0.69424
H 2.20157 1.82134 -2.94308
C 2.93466 2.46630 -0.97369
H 1.93991 2.51168 -0.51519
H 4.78583 -2.94651 2.37163
H 4.76658 3.64453 -0.81113
C 3.94407 -2.36285 2.75783
H 4.35836 -1.48392 3.26075
C 2.95655 0.23184 0.31066
C 2.96215 -1.97410 1.64276
C 3.55215 1.12105 -0.61478
C 3.57465 -0.99294 0.65200
C 4.78975 -1.31210 0.03703
C 4.77172 0.75269 -1.19352
C 5.38674 -0.45352 -0.87862
H 5.27964 -2.24973 0.28444
H 5.25148 1.42705 -1.89746
H 2.10351 -1.48667 2.11596
H 6.33367 -0.71953 -1.33921
H 3.43968 -2.97812 3.50972
H 3.25005 -3.76611 0.42710
C 2.43814 -3.23062 0.93102
H 1.98062 -3.91886 1.64934
H 1.68501 -2.98214 0.17528
H -3.62596 2.95294 -2.62297
H -2.11974 2.04127 -2.65250
H -2.09099 3.79842 -2.42267
H -1.69333 2.70349 -0.25246
H -1.88517 -4.28081 -2.04393
S -1.54883 -1.11466 -2.58895
C -0.03946 -0.26868 -2.24444
C 1.03891 -1.17326 -2.36215
C -1.50114 -3.88574 -2.99137
C -0.74051 -2.62207 -2.76138
C 0.63438 -2.49011 -2.60080
H 0.02449 0.77412 -2.55606
H -2.35341 -3.73527 -3.65870
H 2.07424 -0.85564 -2.27715
H 1.31443 -3.33108 -2.67356
H -0.84623 -4.64224 -3.42974

TS3[CHZn]
B3PW91
SCF = -2075.59548460
SCF (C6H5Cl) = -2075.64211720
SCF (BJD3) = -2075.89378481
SCF (BS2) = -4016.33300298
H (0 K) = -2074.661001
H (298 K) = -2074.605076
G (298 K) = -2074.754995
G (383 K) = -2074.801589
Low freq. = -1078.5003
Second freq. = 13.8172

108
TS3[CHZn]
Zn 0.78867 0.28798 -0.22042
N 1.13550 2.19374 -0.52857
N 2.62345 -0.39070 -0.13360
C 3.65515 0.42714 -0.37431
C 3.52569 1.78639 -0.70491
C 2.39162 2.61161 -0.75101
H 4.46010 2.29533 -0.90923
C 5.05876 -0.11885 -0.29212
H 5.22535 -0.88269 -1.05712
H 5.23127 -0.60227 0.67354
H 5.79774 0.67148 -0.42637
C 2.86170 -1.76927 0.19250
C 2.98908 -2.72143 -0.84475
C 2.90238 -2.16157 1.54950
C 3.01367 -3.52396 1.84503
C 3.09769 -4.47680 0.83674
C 3.09643 -4.07087 -0.49330
C 2.85532 -1.15426 2.69064
H 3.04619 -3.84268 2.88360
H 3.18850 -5.53017 1.08658
H 3.19418 -4.81691 -1.27753
C 3.02830 -2.33039 -2.31645
C 1.57376 -1.29539 3.52394
C 4.09268 -1.25706 3.59532
H 4.13242 -2.21860 4.11741
H 4.07573 -0.47004 4.35656
H 5.02089 -1.15372 3.02549
C 1.75977 -2.77445 -3.05757
H 1.79627 -2.45778 -4.10566
H 0.85908 -2.35061 -2.60453
H 1.65797 -3.86534 -3.04451
C 4.27462 -2.88058 -3.02696
H 3.07174 -1.23785 -2.37285
H 4.25134 -3.97311 -3.09534

H 5.19921 -2.60569 -2.51015
H 4.33081 -2.49132 -4.04873
C 2.66575 4.07232 -1.03179
H 2.94498 4.58950 -0.10733
H 1.80084 4.58906 -1.44847
H 3.50694 4.16944 -1.72170
H -0.59072 3.43353 3.45264
H 2.17674 5.08115 3.16091
H 1.04594 2.93631 3.90563
C 1.37174 5.19726 2.42829
C 0.33743 2.99195 3.07260
H 1.74048 5.82999 1.61555
H 0.12504 1.96586 2.75199
C 0.91881 3.82144 1.91886
H 1.81029 3.29240 1.57077
H -0.11099 4.24441 -4.00181
H 0.56005 5.73844 2.92517
C 0.27742 3.24904 -3.76124
H 1.27744 3.36832 -3.33917
C 0.07555 3.15254 -0.42655
C -0.67031 2.51623 -2.79702
C -0.06068 3.92220 0.75390
C -0.84684 3.28417 -1.49308
C -1.90592 4.18637 -1.34607
C -1.14388 4.80363 0.84898
C -2.06127 4.93827 -0.18598
H -2.61600 4.31394 -2.15714
H -1.26361 5.40407 1.74637
H -0.19175 1.55876 -2.55099
H -2.88744 5.63784 -0.09616
H 0.37642 2.69037 -4.69793
H -2.43076 3.11030 -3.95732
C -1.99117 2.21115 -3.51351
H -1.81939 1.50712 -4.33395
H -2.74138 1.77868 -2.84207
H 1.50561 -2.28626 3.98544
H 0.67866 -1.16020 2.90724
H 1.55007 -0.55117 4.32742
H 2.85483 -0.15122 2.25111
H -0.59725 -5.23039 -0.81442
S -0.68034 -2.45063 0.50292
C -0.91138 -0.81734 -0.16112
C -1.52334 -0.99918 -1.42416
C -1.56862 -4.73080 -0.88508
C -1.41588 -3.24580 -0.83469
C -1.80501 -2.32357 -1.79068
H -1.82868 -0.19684 0.65675
H -2.18664 -5.09596 -0.05819
H -1.78057 -0.15497 -2.05626
H -2.28923 -2.61039 -2.71796
H -2.04146 -5.03167 -1.82255
H -7.53336 -3.77469 0.03326
H -2.12384 -1.21384 2.86401
H -3.22833 1.91005 0.28413
C -3.01909 1.64179 1.31747
C -2.39074 -0.15699 2.81258
C -7.45775 -2.78731 -0.42969
C -6.24920 -2.03816 0.06042
C -5.68887 -2.30089 1.31340
C -5.66133 -1.02753 -0.70884
C -4.56669 -0.30583 -0.24715
C -4.59328 -1.58477 1.79147
C -4.02242 -0.57422 1.01422
N -2.85675 0.17599 1.45642
H -6.06811 -0.79582 -1.68987
H -6.11965 -3.07689 1.94077
H -1.50324 0.43947 3.02628
H -2.08888 2.13137 1.60654
H -4.14631 0.46826 -0.87933
H -4.20855 -1.82615 2.77495
H -8.37774 -2.24169 -0.18840
H -7.43502 -2.92020 -1.51502
H -3.83615 1.99381 1.95503
H -3.15632 0.06777 3.56231

TS4[P-ZnMe]
B3PW91
SCF = -1911.79426198
SCF (C6H5Cl) = -1911.84524217
SCF (BJD3) = -1912.04118893
SCF (BS2) = -3464.53372244
H (0 K) = -1910.909892
H (298 K) = -1910.857614
G (298 K) = -1911.004527
G (383 K) = -1911.049985
Low freq. = -943.1259
Second freq. = 2.4960

101
TS4[P-ZnMe]
C -2.25523 4.73216 0.52751
C -2.19507 4.10734 -0.71223
C -1.12909 3.26915 -1.05468
C -0.10293 3.07007 -0.10329
C -0.14430 3.70414 1.15921
C -1.23580 4.52865 1.44995
N 0.95942 2.14757 -0.39645
C 2.10782 2.56142 -0.95353
C 2.27147 4.02745 -1.26279
C 0.94316 3.51050 2.20741

C	0.42656	2.69088	3.39973
C	-1.11363	2.60914	-2.42674
C	-2.28595	1.63061	-2.59304
Zn	0.77786	0.28202	0.01192
N	2.45282	-0.47958	-0.54744
C	2.69826	-1.88417	-0.38038
C	2.30227	-2.78326	-1.39660
C	2.46177	-4.15359	-1.16677
C	2.99592	-4.63005	0.02461
C	3.38622	-3.73229	1.01129
C	3.25067	-2.35154	0.83408
C	1.70286	-2.31128	-2.71408
C	2.47151	-2.84857	-3.92991
C	3.68627	-1.40845	1.94737
C	5.13922	-1.64905	2.38163
C	3.37159	0.33510	-1.08422
C	4.68712	-0.25217	-1.52606
C	3.19185	1.71939	-1.26417
C	2.74305	-1.50467	3.15645
C	0.21580	-2.68495	-2.81216
C	1.52958	4.84412	2.69328
C	-1.11864	3.64223	-3.56363
H	4.03672	2.22576	-1.71381
H	4.52937	-1.04232	-2.26618
H	5.20616	-0.71567	-0.68150
H	5.33636	0.50891	-1.95918
H	3.81104	-4.10986	1.93746
H	3.11525	-5.69839	0.18069
H	2.16587	-4.85940	-1.93824
H	1.70691	-1.27726	2.88054
H	3.04379	-0.80200	3.94061
H	2.75568	-2.51149	3.58767
H	3.62561	-0.38472	1.56354
H	5.26666	-2.63099	2.84864
H	5.44605	-0.89621	3.11487
H	5.82967	-1.59540	1.53456
H	-0.21909	-2.30621	-3.74302
H	-0.36175	-2.27228	-1.97732
H	0.08007	-3.77173	-2.79905
H	1.77103	-1.21868	-2.74150
H	2.39299	-3.93759	-4.01059
H	3.53544	-2.59798	-3.88065
H	2.06745	-2.42205	-4.85371
H	3.19493	4.21540	-1.81081
H	2.29318	4.61246	-0.33766
H	1.42836	4.40257	-1.84875
H	-0.39821	3.20434	3.90517
H	2.37014	4.66519	3.37153
H	1.22192	2.53102	4.13520
H	1.89165	5.45613	1.86173
H	0.05731	1.70789	3.08582
H	1.75850	2.94312	1.74640
H	-2.04166	4.23118	-3.56723
H	0.78940	5.43748	3.23968
H	-0.28194	4.34226	-3.48165
H	-2.98960	4.27706	-1.43407
H	-1.28428	5.02492	2.41541
H	-0.18565	2.03386	-2.51255
H	-3.09064	5.38200	0.77184
H	-1.04326	3.14151	-4.53442
H	-3.24754	2.15316	-2.55283
H	-2.22803	1.11851	-3.55919
H	-2.29788	0.86881	-1.80598
C	-0.80969	-0.81289	0.89149
H	-1.82058	-1.53178	1.41450
H	-1.35164	-0.60057	-0.03303
H	-0.08839	-1.63254	0.91859
H	-0.79842	-0.05366	1.67574
H	-7.29192	-0.50553	-2.05926
H	-7.68228	-2.23230	-2.03704
C	-7.44158	-1.37166	-1.40797
C	-6.23237	-1.63325	-0.55243
C	-5.50092	-0.57655	0.00584
C	-5.81025	-2.93402	-0.26783
H	-5.79668	0.44858	-0.20157
H	-6.35306	-3.77654	-0.68807
C	-4.39795	-0.80991	0.81834
C	-4.70871	-3.18451	0.54998
H	-2.07339	-3.98175	0.90601
C	-3.99742	-2.12019	1.10147
H	-3.85051	0.03368	1.23030
H	-8.32033	-1.16241	-0.78661
H	-4.42528	-4.21255	0.74631
N	-2.82680	-2.31039	1.94316
C	-2.25926	-3.66727	1.93467
H	-1.31003	-3.64730	2.47576
C	-3.03652	-1.84206	3.33056
H	-3.41025	-0.81808	3.32448
H	-2.92186	-4.38933	2.42330
H	-2.08255	-1.87096	3.86398
H	-3.76061	-2.47985	3.84836

INT3[P-ZnMe]
B3PW91
SCF = -1506.39715862
SCF (C6H5Cl) = -1506.45686876
SCF (BJD3) = -1506.58959061
SCF (BS2) = -3059.01089124
H (0 K) = -1505.712726
H (298 K) = -1505.671193
G (298 K) = -1505.789987
G (383 K) = -1505.826629
Low freq. = 13.3456
Second freq. = 16.6374

78
INT3[P-ZnMe]

C	-5.41811	-0.00007	-1.11929
C	-4.78591	1.20237	-0.82644
C	-3.51557	1.23336	-0.24251
C	-2.88806	0.00004	0.03988
C	-3.51569	-1.23334	-0.24201
C	-4.78601	-1.20245	-0.82599
N	-1.54438	0.00010	0.55942
C	-1.28081	0.00008	1.88017
C	-2.44646	-0.00005	2.83163
C	-2.86014	-2.57632	0.04971
C	-2.47731	-3.30043	-1.25049
C	-2.86003	2.57643	0.04887
C	-2.47863	3.30100	-1.25148
Zn	0.00716	0.00006	-0.48984
N	1.56272	0.00000	0.55143
C	2.90553	-0.00003	0.02908
C	3.53287	1.23318	-0.25274
C	4.80442	1.20243	-0.83376
C	5.43740	-0.00005	-1.12467
C	4.80430	-1.20252	-0.83397
C	3.53276	-1.23326	-0.25294
C	2.87186	2.57511	0.03050
C	3.75619	3.49460	0.88515
C	2.87162	-2.57518	0.03003
C	3.75588	-3.49489	0.88453
C	1.30429	0.00003	1.87370
C	2.47452	0.00007	2.81966
C	0.01326	0.00008	2.44190
C	2.47252	-3.27880	-1.27651
C	2.47276	3.27898	-1.27591
C	-3.74444	-3.47840	0.92315
C	-3.74375	3.47804	0.92340
H	0.07678	-0.94261	-2.35295
H	-1.96905	4.24536	-1.03468
H	-1.81460	2.69502	-1.87879
C	-0.19005	-0.00023	-2.86773
H	-1.25146	-0.00138	-3.11063
H	0.43205	0.00043	-3.76541
H	0.07469	0.94275	-2.35298
H	0.01604	0.00012	3.52432
H	3.10636	-0.87687	2.65008
H	2.14483	0.00018	3.85832
H	3.10643	0.87694	2.64992
H	5.30747	-2.13799	-1.06128
H	6.42551	-0.00006	-1.57521
H	5.30768	2.13788	-1.06091
H	1.81092	-2.65656	-1.89116
H	1.95421	-4.22055	-1.06933
H	3.35212	-3.50809	-1.88653
H	1.95444	-2.38879	0.59973
H	4.66655	-3.78932	0.35370
H	3.21502	-4.41195	1.13864
H	4.06012	-3.01314	1.81864
H	1.95452	4.22073	-1.06855
H	1.81111	2.65689	-1.89065
H	3.35236	3.50832	-1.88590
H	1.95468	2.38868	0.60020
H	4.66687	3.78908	0.35435
H	4.06043	3.01263	1.81915
H	3.21540	4.41164	1.13945
H	-2.11212	0.00125	3.86880
H	-3.07811	-0.87781	2.66575
H	-3.07989	0.87607	2.66401
H	-3.36347	-3.52990	-1.85092
H	-3.20890	-4.39673	1.18386
H	-1.96785	-4.24481	-1.03348
H	-4.03742	-2.98325	1.85385
H	-1.81268	-2.69416	-1.87688
H	-1.93682	-2.38936	0.60922
H	-4.66190	3.77059	0.40448
H	-4.66185	-3.77139	0.40318
H	-4.03543	2.98267	1.85440
H	-5.28913	2.13790	-1.05355
H	-5.28928	-2.13803	-1.05278
H	-1.93615	2.38953	0.60748
H	-6.40521	-0.00012	-1.57205
H	-3.20835	4.39660	1.18358
H	-3.36541	3.53062	-1.85093

TS5[P-ZnMe]
B3PW91
SCF = -1506.38974600
SCF (C6H5Cl) = -1506.44798082
SCF (BJD3) = -1506.58355487
SCF (BS2) = -3059.00272703
H (0 K) = -1505.706195
H (298 K) = -1505.665593
G (298 K) = -1505.779648
G (383 K) = -1505.814915
Low freq. = -40.1034
Second freq. = 17.6238

78
TS5[P-ZnMe]
C -5.33805 0.64069 -1.00298
C -4.42830 1.69457 -1.00703
C -3.16197 1.55209 -0.43738
C -2.83465 0.30876 0.15082
C -3.73807 -0.77198 0.16235
C -4.99266 -0.57443 -0.42656
N -1.50699 0.14550 0.67186
C -1.19993 0.30668 1.97367
C -2.32506 0.60418 2.92867
C -3.38284 -2.13143 0.74633
C -3.14686 -3.15831 -0.37265
C -2.14612 2.68705 -0.46929
C -1.18448 2.53017 -1.65612
Zn 0.03076 -0.08591 -0.36936
N 1.62319 -0.06413 0.62126
C 2.93561 -0.26482 0.07689
C 3.82506 0.81854 -0.06382
C 5.07224 0.56169 -0.64568
C 5.42291 -0.71028 -1.07799
C 4.52029 -1.76263 -0.95132
C 3.26280 -1.56170 -0.38108
C 3.45515 2.24198 0.32605
C 4.52680 2.92251 1.18882
C 2.24014 -2.68573 -0.29441
C 2.84619 -4.09139 -0.22963
C 1.37650 0.01625 1.94274
C 2.54254 -0.09249 2.88917
C 0.09937 0.20583 2.51641
C 1.26366 -2.60697 -1.47693
C 3.16360 3.07883 -0.93038
C -4.44116 -2.64492 1.73276
C -2.77290 4.08527 -0.50291
H -1.74466 -0.68403 -2.62672
H -0.40791 3.30067 -1.65315
H -0.66122 1.54921 -1.65934
C -1.24266 -0.74784 -3.59874
H -1.87036 -0.24319 -4.33525
H -1.12129 -1.79595 -3.87654
H -0.26350 -0.26021 -3.58346
H 0.12317 0.29957 3.59481
H 3.20005 -0.91712 2.60064
H 2.20223 -0.24701 3.91322
H 3.14658 0.82005 2.86185
H 4.80406 -2.75098 -1.29748
H 6.39905 -0.88388 -1.52096
H 5.77919 1.37733 -0.76545
H 0.80316 -1.59683 -1.59820
H 0.44333 -3.32490 -1.38633
H 1.77634 -2.77190 -2.42992
H 1.66420 -2.54394 0.62822
H 3.34213 -4.37133 -1.16431
H 2.06204 -4.83185 -0.04524
H 3.57818 -4.16746 0.57921
H 2.85168 4.09197 -0.65600
H 2.37146 2.63072 -1.54019
H 4.05397 3.16250 -1.56201
H 2.53178 2.20607 0.91439
H 5.45160 3.09206 0.62863
H 4.78240 2.32804 2.07154
H 4.17204 3.89996 1.53014
H -1.94574 0.85624 3.91884
H -2.98979 -0.26058 3.02031
H -2.93569 1.43287 2.55851
H -4.05535 -3.31190 -0.96413
H -4.09928 -3.57093 2.20549
H -2.85361 -4.12624 0.04693
H -4.65056 -1.91842 2.52407
H -2.36003 -2.83301 -1.06201
H -2.44209 -2.02763 1.29828
H -3.28060 4.28782 -1.45123
H -5.38866 -2.86723 1.23207
H -3.49912 4.21390 0.30464
H -4.71366 2.64036 -1.45567
H -5.71028 -1.38962 -0.43320
H -1.55073 2.62705 0.44995
H -6.31903 0.77039 -1.45037
H -1.99705 4.84746 -0.38354
H -1.71771 2.56878 -2.61088

INT4[P-ZnMe]
B3PW91
SCF = -1465.88330881
SCF (C6H5Cl) = -1465.93945546
SCF (BJD3) = -1466.06735907
SCF (BS2) = -3018.48529403
H (0 K) = -1465.245684
H (298 K) = -1465.208971
G (298 K) = -1465.313642
G (383 K) = -1465.346048
Low freq. = 15.8475
Second freq. = 24.4498

73
INT4[P-ZnMe]
C -5.32624 -0.82535 -1.19535
C -5.08814 0.36250 -0.51705
C -3.85357 0.62661 0.08823
C -2.85820 -0.36726 0.00101
C -3.06841 -1.56608 -0.72042
C -4.31722 -1.77822 -1.30482
N -1.55472 -0.15361 0.55690
C -1.27806 -0.21629 1.87345
C -2.39884 -0.52584 2.83054
C -1.93129 -2.56339 -0.89359
C -1.02135 -2.13945 -2.06024
C -3.60649 1.98536 0.72766
C -3.41622 3.05384 -0.36179
Zn -0.00000 -0.00148 -0.47774
N 1.55438 0.15437 0.55656
C 2.85811 0.36700 0.00071
C 3.06927 1.56521 -0.72138
C 4.31835 1.77595 -1.30578
C 5.32662 0.82237 -1.19559
C 5.08761 -0.36478 -0.51636
C 3.85279 -0.62745 0.08900
C 1.93310 2.56348 -0.89527
C 2.38360 4.01164 -1.11265
C 3.60468 -1.98528 0.72999
C 4.71476 -2.40154 1.70454
C 1.27750 0.22038 1.87295
C 2.39840 0.53234 2.82910
C -0.00026 0.00273 2.43390
C 3.41328 -3.05486 -0.35816
C 1.02347 2.14029 -2.06232
C -2.38021 -4.01200 -1.11121
C -4.71676 2.40178 1.70192
H -0.00029 0.00403 3.51735
H 3.06258 1.29473 2.41416
H 3.00787 -0.35736 3.01738
H 2.00539 0.88069 3.78482
H 5.87412 -1.11146 -0.46031
H 6.29587 1.00286 -1.65066
H 4.51252 2.69464 -1.84946
H 2.59478 -2.79507 -1.03852
H 3.18692 -4.02715 0.09153
H 4.31891 -3.16709 -0.96308
H 2.66895 -1.93244 1.29628
H 5.66047 -2.58914 1.18659
H 4.43897 -3.32828 2.21718
H 4.90316 -1.63648 2.46417
H 0.10828 2.73702 -2.11956
H 0.72815 1.05912 -2.03613
H 1.54850 2.20996 -3.02091
H 1.33521 2.55278 0.02433
H 2.89655 4.14620 -2.07002
H 3.06009 4.33384 -0.31643
H 1.51834 4.68138 -1.11160
H -2.00562 -0.87559 3.78568
H -3.06547 -1.28629 2.41605
H -3.00565 0.36543 3.02005
H -1.54509 -2.21207 -3.01934
H -1.51426 -4.68086 -1.10999
H -0.10447 -2.73375 -2.11576
H -3.05654 -4.33492 -0.31515
H -0.72975 -1.05710 -2.03628
H -1.33401 -2.55215 0.02640
H -5.66271 2.58799 1.18389
H -2.89281 -4.14700 -2.06870
H -4.90441 1.63743 2.46244
H -5.87517 1.10869 -0.46174
H -4.51061 -2.69743 -1.84790
H -2.67058 1.93402 1.29377
H -6.29533 -1.00691 -1.65035
H -4.44170 3.32935 2.21344
H -4.32206 3.16461 -0.96666
H -3.19064 4.02683 0.08677
H -2.59759 2.79399 -1.04196

1-Et
B3PW91
SCF = -1545.31170792
SCF (C6H5Cl) = -1545.31959140
SCF (BJD3) = -1545.51213132
SCF (BS2) = -3097.93836965
H (0 K) = -1544.609045
H (298 K) = -1544.567681
G (298 K) = -1544.684367
G (383 K) = -1544.720454
Low freq. = 16.5858
Second freq. = 19.6830

80
1-Et
C 5.33459 -0.02368 -0.92721
C 4.67534 -1.22603 -0.70327
C 3.39816 -1.26125 -0.13479
C 2.77852 -0.03929 0.21270
C 3.44291 1.19099 -0.00127
C 4.71731 1.17098 -0.57501
N 1.43573 -0.04332 0.70841
C 1.20943 -0.00903 2.02330
C 2.38463 -0.00757 2.97386
C 2.79911 2.52667 0.34490
C 2.35181 3.26726 -0.92399
C 2.71683 -2.60448 0.08329
C 2.41593 -3.30233 -1.25115
Zn -0.02125 -0.11262 -0.65138
N -1.52163 0.01253 0.67009
C -2.84755 0.04849 0.13345
C -3.54450 -1.16009 -0.09591
C -4.80529 -1.10118 -0.69727
C -5.37466 0.11091 -1.06842
C -4.67888 1.29188 -0.83929
C -3.41587 1.28799 -0.23994
C -2.95313 -2.51527 0.26524
C -3.87484 -3.33161 1.18234
C -2.68114 2.60504 -0.03551
C -3.51964 3.63235 0.73753
C -1.33327 0.06587 1.98835
C -2.53200 0.16739 2.90314
C -0.06920 0.03662 2.60440
C -2.21483 3.18656 -1.37874
C -2.60278 -3.31666 -0.99756
C 3.71378 3.42025 1.19421
C 3.53523 -3.52371 1.00160
H -0.08276 0.06774 3.68726
H -3.21307 -0.67503 2.75024
H -3.10681 1.07331 2.68649
H -2.22978 0.18828 3.95123
H -5.12321 2.23894 -1.13498
H -6.35503 0.13490 -1.53646
H -5.34849 -2.02461 -0.88195
H -1.57511 2.48058 -1.91718
H -1.64963 4.11279 -1.22581
H -3.06900 3.41780 -2.02504
H -1.78702 2.39638 0.56063
H -4.40606 3.94109 0.17284
H -2.92918 4.53346 0.93619
H -3.86245 3.23521 1.69838
H -2.13045 -4.26953 -0.73323
H -1.91608 -2.76146 -1.64312
H -3.50088 -3.54009 -1.58425
H -2.02100 -2.33567 0.81040
H -4.81084 -3.59971 0.68030
H -4.13508 -2.78141 2.09222
H -3.38519 -4.26480 1.48160
H 2.05319 -0.08409 4.01049
H 2.97119 0.91023 2.86505
H 3.06349 -0.83736 2.75706
H 3.20779 3.49367 -1.56946
H 3.17843 4.32366 1.50591
H 1.86251 4.21406 -0.66900
H 4.06241 2.90629 2.09581
H 1.64829 2.66642 -1.50771
H 1.90055 2.32081 0.93503
H 4.49770 -3.78953 0.55085
H 4.59927 3.74356 0.63640
H 3.74228 -3.05200 1.96741
H 5.15992 -2.16007 -0.97681
H 5.23436 2.11041 -0.75274
H 1.76009 -2.41211 0.57960
H 6.32526 -0.01701 -1.37359
H 2.99210 -4.45600 1.19171
H 3.33885 -3.53639 -1.79344
H 1.88164 -4.24404 -1.08235
H 1.80053 -2.67212 -1.89969
C -0.11067 -0.33301 -2.60702
H -1.05851 0.10132 -2.95108
C 1.04955 0.21497 -3.44598
H -0.21237 -1.41153 -2.79564
H 0.92974 -0.01375 -4.51446
H 1.13300 1.30469 -3.36555
H 2.01507 -0.19744 -3.13358

TS4[P-ZnEt]
B3PW91
SCF = -1951.08289201
SCF (C6H5Cl) = -1951.13223860
SCF (BJD3) = -1951.34495546
SCF (BS2) = -3503.83250055
H (0 K) = -1950.169356
H (298 K) = -1950.115818
G (298 K) = -1950.263279
G (383 K) = -1950.308961
Low freq. = -1094.4797
Second freq. = 7.5377

104
TS4[P-ZnEt]
C 2.16569 4.60485 0.28784
C 1.83797 3.93513 1.46097
C 0.70727 3.11474 1.54033
C -0.09867 2.96923 0.38726
C 0.21125 3.65833 -0.80875
C 1.35158 4.46846 -0.83123
N -1.18899 2.03742 0.40360
C -2.43383 2.41602 0.72454
C -2.68319 3.85773 1.08576
C -0.65208 3.55339 -2.05908
C 0.08595 2.83298 -3.19733
C 0.37845 2.42578 2.85836
C 1.42371 1.35987 3.21995
Zn -0.86216 0.18018 -0.03243
N -2.59831 -0.61929 0.15935
C -2.77673 -2.03158 -0.04457
C -2.69807 -2.90771 1.06168
C -2.76986 -4.28287 0.81969
C -2.92378 -4.78489 -0.46750
C -3.01739 -3.90824 -1.54142
C -2.94857 -2.52320 -1.35773
C -2.51794 -2.40597 2.48838
C -3.53765 -3.01779 3.45945
C -3.06392 -1.60428 -2.56612
C -4.38964 -1.80353 -3.31560
C -3.63334 0.16593 0.49193
C -4.99965 -0.45832 0.61982
C -3.53923 1.54726 0.74604
C -1.87550 -1.78174 -3.52313
C -1.08667 -2.65609 2.98697
C -1.14642 4.92708 -2.53712
C 0.22727 3.43210 4.00925
H -4.47717 2.01913 1.01074
H -5.03218 -1.14107 1.47495
H -5.24255 -1.05372 -0.26436
H -5.76889 0.30186 0.75768
H -3.15128 -4.30543 -2.54406
H -2.97886 -5.85713 -0.63172
H -2.70606 -4.97363 1.65575
H -0.91895 -1.61437 -3.01576
H -1.94561 -1.08017 -4.36127
H -1.84874 -2.79412 -3.93965
H -3.04770 -0.57071 -2.20363
H -4.46131 -2.80720 -3.74705
H -4.47564 -1.08487 -4.13724
H -5.25208 -1.66559 -2.65670
H -0.95946 -2.27695 4.00653
H -0.34616 -2.16304 2.34790
H -0.85332 -3.72613 2.99482
H -2.67738 -1.32265 2.48788
H -3.37866 -4.09249 3.59402
H -4.56581 -2.87862 3.11138
H -3.44846 -2.55064 4.44550
H -3.73061 4.02946 1.33441
H -2.40736 4.51921 0.25925
H -2.06774 4.15347 1.94077
H 0.98019 3.38685 -3.50366
H -1.83685 4.81108 -3.37871
H -0.55905 2.73290 -4.07641
H -1.67018 5.46974 -1.74470
H 0.40237 1.82683 -2.89921
H -1.53392 2.95465 -1.80919
H 1.17411 3.93281 4.23616
H -0.31914 5.55914 -2.87602
H -0.50721 4.20846 3.77545
H 2.46508 4.06158 2.33959
H 1.59916 5.01180 -1.73948
H -0.58376 1.91688 2.73989
H 3.04264 5.24497 0.25083
H -0.10057 2.92130 4.92033
H 2.41882 1.80266 3.33680
H 1.16567 0.86932 4.16413
H 1.49331 0.58075 2.45192
C 1.04138 -0.52411 -0.65294
H 1.30693 0.16047 0.16294
C 1.02648 -2.04632 -0.45237
H 0.69530 -0.08582 -1.59789
H 1.74178 -2.54412 -1.11670
H 1.33167 -2.29171 0.56780
H 0.05289 -2.51329 -0.62122
H 2.32542 -0.34362 -1.07794
H 3.19560 -1.55958 -3.14258
C 4.44161 -1.05836 0.57701
H 3.78458 -0.35791 1.08499
C 3.47195 -0.51365 -3.00038

H 2.68444 0.12097 -3.41397
C 5.24796 -1.90368 1.32855
N 3.59681 -0.21960 -1.56403
H 6.46259 -4.12110 2.40934
H 5.20633 -1.84749 2.41334
C 4.47380 -1.11299 -0.82052
C 6.10704 -2.82715 0.71756
C 5.32095 -2.02563 -1.44663
C 6.99379 -3.72103 1.54045
C 6.12167 -2.87066 -0.67806
H 7.36872 -4.56385 0.95462
H 5.37510 -2.09484 -2.52726
C 3.93753 1.20764 -1.36022
H 3.16760 1.82936 -1.82366
H 6.77503 -3.57608 -1.18453
H 4.40250 -0.30511 -3.53888
H 3.97169 1.43484 -0.29510
H 7.86258 -3.16891 1.91763
H 4.91179 1.43579 -1.80483

INT3[P-ZnEt]
B3PW91
SCF = -1545.70085879
SCF (C6H5Cl) = -1545.75841216
SCF (BJD3) = -1545.90071038
SCF (BS2) = -3098.32582186
H (0 K) = -1544.986654
H (298 K) = -1544.944263
G (298 K) = -1545.064877
G (383 K) = -1545.102112
Low freq. = 15.2460
Second freq. = 16.6853

81
INT3[P-ZnEt]
C 3.55556 -1.23321 -0.20279
C 2.93387 -0.00001 0.09176
C 3.55581 1.23299 -0.20310
C 4.81487 1.20208 -0.81078
C 5.44154 -0.00041 -1.11502
C 4.81463 -1.20269 -0.81049
N 1.60155 0.00019 0.64024
C 1.37116 0.00033 1.96678
C 0.09202 0.00038 2.56133
C -1.21100 0.00023 2.02260
C -2.36048 0.00033 2.99380
C 2.90256 2.57540 0.09576
C 2.48132 3.28463 -1.20075
C 2.90205 -2.57541 0.09639
C 2.48045 -3.28475 -1.19995
N -1.49631 0.00006 0.70675
Zn 0.02998 -0.00009 -0.38346
C -0.03083 0.00026 -2.78548
C -2.84841 -0.00002 0.21043
C -3.48018 1.23318 -0.06286
C -4.75658 1.20244 -0.63358
C -5.39125 -0.00015 -0.92127
C -4.75636 -1.20267 -0.63380
C -3.47996 -1.23327 -0.06307
C -2.82096 2.57535 0.22396
C -3.70077 3.48228 1.09674
C -2.82057 -2.57539 0.22362
C -3.70009 -3.48227 1.09675
C -2.43832 -3.29446 -1.07934
C -2.43836 3.29432 -1.07895
C 3.80449 3.48993 0.93730
C 3.80388 -3.49003 0.93795
C 2.56018 0.00043 2.88927
H -0.02688 -0.93787 -2.18478
H -1.92637 4.23833 -0.86632
H -1.77533 2.68403 -1.70356
C -1.24674 0.00010 -3.70911
H 0.92986 0.00011 -3.30659
H -0.02684 0.93853 -2.18506
H 0.11544 0.00053 3.64351
H 3.18861 -0.87648 2.70734
H 2.25124 0.00055 3.93432
H 3.18860 0.87730 2.70713
H 5.31297 -2.13834 -1.04762
H 6.42001 -0.00056 -1.58616
H 5.31340 2.13757 -1.04814
H 1.80464 -2.66693 -1.80299
H 1.96980 -4.22764 -0.97907
H 3.34923 -3.51383 -1.82546
H 1.99497 -2.38863 0.68188
H 4.70543 -3.78418 0.39154
H 3.26994 -4.40746 1.20511
H 4.12413 -3.00438 1.86467
H 1.97084 4.22766 -0.98009
H 1.80548 2.66687 -1.80380
H 3.35026 3.51345 -1.82616
H 1.99534 2.38891 0.68114
H 4.70618 3.78377 0.39098
H 4.12449 3.00437 1.86415
H 3.27073 4.40754 1.20423
H -2.00844 0.00026 4.02513
H -2.99592 -0.87663 2.83819
H -2.99571 0.87746 2.83826
H -3.32493 -3.52319 -1.67949
H -3.16033 -4.39873 1.35536

H -1.92620 -4.23841 -0.86677
H -3.99332 -2.98930 2.02852
H -1.77554 -2.68418 -1.70422
H -1.89647 -2.38723 0.78123
H -4.61765 3.77867 0.57771
H -4.61718 -3.77859 0.57805
H -3.99439 2.98934 2.02841
H -5.26279 2.13797 -0.85399
H -5.26241 -2.13825 -0.85437
H -1.89702 2.38729 0.78187
H -6.38297 -0.00020 -1.36387
H -3.16105 4.39870 1.35555
H -3.32481 3.52291 -1.67939
H -1.23185 0.88434 -4.35059
H -1.23196 -0.88441 -4.35022
H -2.18231 0.00028 -3.14532

TS5[P-ZnEt]
B3PW91
SCF = -1545.69068831
SCF (C6H5Cl) = -1545.74902864
SCF (BJD3) = -1545.88965627
SCF (BS2) = -3098.31512022
H (0 K) = -1544.977511
H (298 K) = -1544.935950
G (298 K) = -1545.053672
G (383 K) = -1545.090047
Low freq. = -29.8860
Second freq. = 12.7118

81
TS5[P-ZnEt]
C -3.29322 -1.60038 0.30585
C -3.00884 -0.27493 -0.09561
C -3.90820 0.78221 0.14613
C -5.12282 0.47012 0.76857
C -5.43264 -0.82995 1.14491
C -4.51934 -1.85642 0.92031
N -1.72675 -0.02238 -0.68793
C -1.54593 0.14823 -2.01131
C -0.30068 0.39643 -2.63051
C 1.02108 0.48270 -2.14271
C 2.09397 0.87299 -3.12411
C -3.57922 2.23230 -0.17656
C -3.24410 3.00047 1.11246
C -2.25524 -2.69654 0.11339
C -1.22735 -2.65883 1.25391
N 1.39156 0.23259 -0.87202
Zn -0.08897 -0.09674 0.22619
C 2.73873 0.38637 -0.40133
C 3.06415 1.58377 0.27646
C 4.35372 1.71525 0.79410
C 5.28852 0.69298 0.65436
C 4.94421 -0.47914 -0.00573
C 3.66650 -0.66329 -0.54742
C 2.02103 2.67934 0.45696
C 2.60934 4.08890 0.58311
C 3.31433 -1.98086 -1.22225
C 4.33546 -2.38800 -2.29374
C 3.15413 -3.09800 -0.17885
C 1.12650 2.39053 1.67132
C -4.69969 2.94184 -0.94930
C -2.83609 -4.10984 0.00192
C -2.75423 0.08245 -2.90746
C 1.39679 -1.09233 3.37010
H 1.76413 -0.86372 2.35792
H 0.32022 3.12371 1.76625
H 0.64583 1.38782 1.62675
C 2.45671 -0.73899 4.40981
H 1.14959 -2.15835 3.39677
H 0.46928 -0.53397 3.54835
H -0.37737 0.56328 -3.69760
H -3.39062 -0.76493 -2.63821
H -2.46055 -0.00808 -3.95340
H -3.36388 0.98515 -2.79919
H -4.77009 -2.86730 1.22437
H -6.38447 -1.04579 1.62097
H -5.83652 1.26451 0.96594
H -0.79327 -1.64261 1.42333
H -0.39135 -3.34337 1.08360
H -1.69065 -2.89699 2.21671
H -1.72551 -2.49471 -0.82556
H -3.28496 -4.44775 0.94121
H -2.04688 -4.82298 -0.25453
H -3.60140 -4.15978 -0.77756
H -2.95944 4.03286 0.88401
H -2.41919 2.53119 1.65962
H -4.10645 3.03255 1.78630
H -2.68294 2.24772 -0.80616
H -5.60036 3.06205 -0.33922
H -4.98650 2.39488 -1.85289
H -4.37615 3.94365 -1.24845
H 1.66299 1.19195 -4.07302
H 2.76752 0.03127 -3.31338
H 2.70820 1.68330 -2.72060
H 4.09342 -3.27537 0.35514
H 3.99137 -3.28189 -2.82328
H 2.86076 -4.03679 -0.66006
H 4.49203 -1.59619 -3.03292
H 2.39527 -2.84641 0.57006

H 2.34604 -1.85859 -1.72005
H 3.16129 4.22425 1.51859
H 5.31008 -2.62578 -1.85613
H 3.28749 4.31054 -0.24575
H 4.63762 2.62770 1.30804
H 5.68113 -1.27098 -0.10325
H 1.38131 2.68012 -0.43393
H 6.28779 0.81343 1.06212
H 1.80752 4.83329 0.57126
H 1.70505 2.37886 2.60002
H 2.10510 -0.97035 5.41936
H 3.37840 -1.30241 4.23989
H 2.70889 0.32514 4.38180

TS1[P-ZnMe]
B3PW91
SCF = -1911.80158006
SCF (C6H5Cl) = -1911.85261916
SCF (BJD3) = -1912.06057984
SCF (BS2) = -3464.53577504
H (0 K) = -1910.915981
H (298 K) = -1910.864270
G (298 K) = -1911.004814
Low freq. = -1129.4456
Second freq. = 8.5336

101
TS1[P-ZnMe]
C -5.99583 -1.82864 -0.05809
C -4.76011 -1.91259 0.58353
C -3.63067 -2.29796 -0.13604
C -3.75418 -2.59269 -1.49816
C -4.99254 -2.50557 -2.12259
C -6.14105 -2.12448 -1.41547
N -2.30393 -2.38282 0.47109
C -1.76239 -3.76378 0.43730
C -7.48175 -2.06088 -2.09331
C -2.21190 -1.84377 1.84062
C -0.65034 -0.77536 -1.03798
C 0.68884 -1.34537 -0.97410
C 0.91904 -2.55918 -1.83964
C -1.00339 0.60371 -0.71122
C -2.29744 1.08614 -1.31536
N -0.27367 1.38218 0.04199
C -0.66748 2.74875 0.28702
C -0.38234 3.74295 -0.67499
C -0.72992 5.06405 -0.37552
C -1.33404 5.40245 0.82847
C -1.58775 4.41299 1.76958
C -1.25390 3.07727 1.52807
C 0.32896 3.45221 -1.98927
C -0.44848 3.97259 -3.20717
C -1.54403 2.03728 2.59958
C -3.05553 1.85539 2.80831
Zn 1.54451 0.88328 0.79696
N 1.64550 -0.87020 -0.22165
C 2.93926 -1.50837 -0.18714
C 3.22888 -2.39761 0.87046
C 4.50657 -2.96129 0.92947
C 5.47601 -2.65356 -0.01682
C 5.17733 -1.76299 -1.03980
C 3.91529 -1.16997 -1.14896
C 2.21421 -2.74896 1.94880
C 2.64468 -2.19942 3.31720
C 3.67093 -0.15909 -2.26036
C 4.48973 1.11852 -2.01641
C -0.84847 2.37280 3.92651
C 1.75123 4.03423 -1.97141
C 1.95592 -4.26095 2.02619
C 3.97209 -0.73254 -3.65281
H -1.15453 -1.07923 -1.95885
H -2.09403 1.51782 -2.30234
H -2.76418 1.86438 -0.70988
H -2.99574 0.25900 -1.45908
H -2.05112 4.68183 2.71475
H -1.59878 6.43503 1.03570
H -0.51772 5.84377 -1.10143
H 0.22897 2.50013 3.79045
H -1.01042 1.57315 4.65738
H -1.24019 3.29713 4.36261
H -1.13712 1.08053 2.25099
H -3.51860 2.78346 3.15856
H -3.25462 1.08818 3.56545
H -3.56545 1.56960 1.88164
H 2.27847 3.79400 -2.90054
H 2.33720 3.64348 -1.13376
H 1.72738 5.12457 -1.87478
H 0.42229 2.36602 -2.09848
H -0.49634 5.06603 -3.21710
H -1.47783 3.60108 -3.22770
H 0.04442 3.66188 -4.13410
H 1.57562 -3.28524 -1.35637
H 1.41357 -2.24909 -2.76718
H -0.02183 -3.03832 -2.11716
H 3.58311 -2.65669 3.64681
H 1.17595 -4.48340 2.76294
H 1.88696 -2.41520 4.07855
H 1.64700 -4.67340 1.05949
H 2.79995 -1.11718 3.28236
H 1.26731 -2.26273 1.68335

H 5.03695 -0.95441 -3.77473
H 2.85371 -4.80464 2.33605
H 3.42232 -1.65963 -3.84440
H 5.94277 -1.51538 -1.76973
H 4.74704 -3.64981 1.73479
H 2.61157 0.12159 -2.24272
H 6.46328 -3.10127 0.04687
H 3.70027 -0.00991 -4.42901
H 5.56346 0.90515 -2.03490
H 4.28498 1.86337 -2.79205
H 4.26117 1.56496 -1.04353
H -2.57566 -0.81636 1.85729
H -2.78787 -2.45048 2.54592
H -1.16262 -1.85600 2.14146
H -7.91530 -3.06312 -2.19026
H -2.88700 -2.89185 -2.08006
H -5.06727 -2.73780 -3.18169
H -4.70428 -1.67638 1.63988
H -6.86541 -1.52773 0.51986
H -8.18940 -1.44994 -1.52769
H -7.40122 -1.64414 -3.10149
H -0.72563 -3.74733 0.77964
H -2.35292 -4.41392 1.08995
H -1.80075 -4.15615 -0.57793
C 2.77060 1.77419 2.01122
H -1.49750 -1.62343 -0.22078
H 2.70512 2.86124 1.90675
H 2.54000 1.52792 3.05303
H 3.80381 1.46969 1.82046

INT0[P-ZnMe]
B3PW91
SCF = -1911.81745986
SCF (C6H5Cl) = -1911.86791554
SCF (BJD3) = -1912.06477064
SCF (BS2) = -3464.55453400
H (0 K) = -1910.929403
H (298 K) = -1910.876059
G (298 K) = -1911.024437
Low freq. = 9.1033
Second freq. = 9.7044

101
INT0[P-ZnMe]
C -4.36050 1.19124 -1.35982
C -4.95467 1.20254 -0.10080
C -5.26719 -0.00142 0.57124
C -4.95320 -1.20419 -0.10220
C -4.35901 -1.19066 -1.36122
C -4.04434 0.00086 -2.02699
N -5.84699 -0.00248 1.82539
C -6.37189 -1.24504 2.35821
C -3.48696 0.00208 -3.42648
C -6.37365 1.23878 2.35952
C -1.16912 -0.00047 0.47938
C -0.47475 -1.33693 0.45728
C -1.35740 -2.46814 0.89062
C -0.47541 1.33635 0.45779
C -1.35859 2.46694 0.89165
N 0.74531 1.49032 0.07921
C 1.35350 2.80329 0.05163
C 1.42221 3.47885 -1.18396
C 2.05583 4.72464 -1.20729
C 2.61992 5.27057 -0.06066
C 2.56222 4.57026 1.13773
C 1.93295 3.32427 1.22635
C 0.82367 2.91752 -2.46625
C -0.39585 3.73583 -2.91808
C 1.93761 2.58288 2.55604
C 1.31194 3.41313 3.68633
Zn 2.05078 0.00054 -0.46008
N 0.74608 -1.49015 0.07876
C 1.35486 -2.80282 0.05067
C 1.93434 -3.32412 1.22523
C 2.56413 -4.56981 1.13612
C 2.62231 -5.26953 -0.06258
C 2.05820 -4.72329 -1.20906
C 1.42408 -3.47776 -1.18524
C 1.93852 -2.58332 2.55525
C 3.36246 -2.14204 2.92890
C 0.82552 -2.91603 -2.46736
C 1.86683 -2.82113 -3.59163
C 3.36179 2.14219 2.92943
C 1.86499 2.82309 -3.59056
C 1.31326 -3.41439 3.68516
C -0.39393 -3.73428 -2.91948
H -1.88053 -0.00051 -0.36895
H -0.97678 3.43431 0.56621
H -1.41718 2.47200 1.98737
H -2.37566 2.31148 0.51858
H 3.01698 4.99721 2.02690
H 3.10946 6.23891 -0.10236
H 2.10936 5.27440 -2.14246
H 3.81005 1.51895 2.14912
H 3.35584 1.57077 3.86329
H 4.01630 3.00769 3.07375
H 1.33431 1.67403 2.44789
H 1.90720 4.30488 3.90579
H 1.25813 2.82361 4.60724
H 0.30060 3.74797 3.43564

H 1.42847 2.34283 -4.47228
H 2.73907 2.24282 -3.28105
H 2.21557 3.81288 -3.89905
H 0.47331 1.89816 -2.26221
H -0.11272 4.76806 -3.14832
H -1.17153 3.77162 -2.14656
H -0.83504 3.30380 -3.82330
H -1.41552 -2.47408 1.98636
H -0.97537 -3.43514 0.56432
H -2.37467 -2.31275 0.51806
H 4.01741 -3.00726 3.07285
H 1.25905 -2.82527 4.60630
H 3.35617 -1.57105 3.86302
H 0.30213 -3.74972 3.43426
H 3.81042 -1.51821 2.14889
H 1.33476 -1.67474 2.44747
H -0.11073 -4.76643 -3.15001
H 1.90902 -4.30588 3.90431
H -1.16965 -3.77034 -2.14801
H 2.11210 -5.27258 -2.14447
H 3.01894 -4.99698 2.02516
H 0.47507 -1.89678 -2.26297
H 3.11224 -6.23766 -0.10466
H -0.83312 -3.30199 -3.82458
H 2.21741 -3.81078 -3.90056
H 1.43029 -2.34050 -4.47314
H 2.74090 -2.24098 -3.28188
H -5.58168 1.98865 2.46896
H -7.16953 1.67054 1.73447
H -6.78355 1.05497 3.35345
H -4.29049 0.00470 -4.17301
H -5.20734 -2.16082 0.34013
H -4.16528 -2.14213 -1.85317
H -5.20990 2.15834 0.34271
H -4.16794 2.14352 -1.85066
H -2.87177 0.88699 -3.61717
H -2.87495 -0.88442 -3.61988
H -6.78180 -1.06292 3.35245
H -7.16733 -1.67717 1.73285
H -5.57888 -1.99397 2.46660
C 3.86363 0.00088 -1.14005
H -1.84926 -0.00081 1.34057
H 4.40500 0.88973 -0.80457
H 4.40511 -0.88807 -0.80504
H 3.86315 0.00116 -2.23448

INT1[P-ZnMe]
B3PW91
SCF = -1506.41350927
SCF (C6H5Cl) = -1506.46924485
SCF (BJD3) = -1506.60610116
SCF (BS2) = -3059.02252738
H (0 K) = -1505.727905
H (298 K) = -1505.687205
G (298 K) = -1505.803683
Low freq. = 12.3325
Second freq. = 13.4257

78
INT1[P-ZnMe]
C 3.34946 1.27825 -0.31065
C 2.80509 0.02522 0.03542
C 3.45340 -1.19006 -0.26591
C 4.70273 -1.11846 -0.88834
C 5.27370 0.10349 -1.22388
C 4.59666 1.28382 -0.94399
N 1.49154 -0.04069 0.64070
Zn 0.00014 -0.33175 -0.74952
C -0.00040 -0.67515 -2.65358
H -0.88950 -0.24786 -3.12498
C 2.85961 -2.54761 0.08446
C 2.82938 -3.49609 -1.12186
C 2.63110 2.59863 -0.06886
C 2.18497 3.22612 -1.39912
C 1.34143 0.11510 1.90757
C 2.47428 0.37833 2.85500
C 0.00001 0.01674 2.59359
C -1.34139 0.11538 1.90751
C -2.47413 0.37901 2.85498
N -1.49155 -0.04061 0.64067
C -2.80508 0.02550 0.03542
C -3.34913 1.27859 -0.31095
C -4.59636 1.28435 -0.94422
C -5.27375 0.10413 -1.22379
C -4.70308 -1.11788 -0.88797
C -3.45374 -1.18967 -0.26558
C -2.63042 2.59883 -0.06948
C -3.48343 3.58735 0.73881
C -2.86033 -2.54731 0.08505
C -3.60185 -3.19061 1.26764
C -2.83004 -3.49587 -1.12121
C -2.18428 3.22597 -1.39991
C 3.60069 -3.19117 1.26718
C 3.48444 3.58679 0.73952
H -3.44034 0.13411 2.41484
H -2.47868 1.44321 3.12179

H -2.33673 -0.18051 3.78537
H -5.04290 2.23404 -1.22380
H -6.24431 0.13612 -1.70954
H -5.23594 -2.03628 -1.11534
H -1.54477 2.54714 -1.97322
H -1.62846 4.15220 -1.22184
H -3.04694 3.47129 -2.02743
H -1.72410 2.39918 0.51435
H -4.37229 3.89943 0.18206
H -2.90797 4.49090 0.96397
H -3.82463 3.15539 1.68477
H -2.31423 -4.42477 -0.85776
H -2.31322 -3.04612 -1.97387
H -3.83844 -3.76763 -1.44786
H -1.81965 -2.39299 0.39898
H -4.65004 -3.37926 1.01400
H -3.58972 -2.55124 2.15626
H -3.14583 -4.14979 1.53343
H 3.44034 0.13261 2.41500
H 2.33646 -0.18067 3.78564
H 2.47956 1.44266 3.12131
H 3.04762 3.47132 -2.02670
H 2.90920 4.49042 0.96496
H 1.62943 4.15246 -1.22081
H 3.82572 3.15458 1.68532
H 1.54520 2.54756 -1.97248
H 1.72479 2.39909 0.51503
H 4.64891 -3.37997 1.01380
H 4.37327 3.89882 0.18267
H 3.58843 -2.55192 2.15588
H 5.23533 -2.03694 -1.11597
H 5.04343 2.23346 -1.22337
H 1.81888 -2.39309 0.39815
H 6.24425 0.13533 -1.70968
H 3.14443 -4.15030 1.53273
H 3.83780 -3.76822 -1.44817
H 2.31317 -4.42482 -0.85863
H 2.31301 -3.04612 -1.97467
H -0.00011 -0.94953 3.12559
H 0.00008 0.74885 3.41130
H 0.88867 -0.24833 -3.12543
H -0.00074 -1.75227 -2.84687

TS2[P-ZnMe]
B3PW91
SCF = -1911.79852691
SCF (C6H5Cl) = -1911.85280484
SCF (BJD3) = -1912.05974004
SCF (BS2) = -3464.53440238
H (0 K) = -1910.908715
H (298 K) = -1910.857617
G (298 K) = -1910.995164
Low freq. = -38.4121
Second freq. = 8.2891

101
TS2[P-ZnMe]
C 2.36129 4.51222 -0.57069
C 1.27133 3.85124 -1.14139
C 1.41606 2.56833 -1.68514
C 2.68891 1.97463 -1.62243
C 3.76782 2.65171 -1.07082
C 3.63281 3.94044 -0.53570
N 0.33803 1.85429 -2.28967
C 0.63673 1.44203 -3.66679
C 4.81682 4.68079 0.02641
Zn -0.61509 -1.04364 -0.83821
N 0.66761 -1.85151 0.61879
C 2.10655 -1.74265 0.49589
C 2.82733 -0.87779 1.34562
C 4.21101 -0.79010 1.15405
C 4.85861 -1.52310 0.16989
C 4.12528 -2.37088 -0.65186
C 2.74097 -2.50095 -0.51417
C 2.19642 -0.04737 2.45483
C 2.73037 -0.45262 3.83883
C 1.99078 -3.49972 -1.38456
C 2.11642 -4.92243 -0.81404
N -2.10425 -0.66071 0.60016
C -3.02508 0.44516 0.43816
C -4.02855 0.32199 -0.55032
C -4.89455 1.40278 -0.73554
C -4.77581 2.56449 0.01781
C -3.78229 2.65950 0.98185
C -2.88445 1.61141 1.21889
C -4.24069 -0.95531 -1.35242
C -4.56698 -0.69131 -2.82782
C -1.83702 1.78848 2.31017
C -0.93822 3.00322 2.04018
C -2.27113 -1.52598 1.53642
C -3.37030 -1.49310 2.55208
C 0.14206 -2.57642 1.54197
C 0.90857 -3.37613 2.54880

C -1.36496 -2.74077 1.62708
C 2.43953 -3.47391 -2.85095
C 2.41872 1.45367 2.22567
C -5.33896 -1.82434 -0.71670
C -2.48982 1.91886 3.69674
C -0.95661 2.52206 -2.24395
H -1.59781 -3.27273 2.55209
H 1.98064 -3.36972 2.35726
H 0.54212 -4.40900 2.55209
H 0.72114 -2.97602 3.55227
H 4.63878 -2.95239 -1.41084
H 5.93438 -1.44051 0.04645
H 4.78949 -0.13305 1.79657
H 2.42811 -2.45985 -3.26072
H 1.77218 -4.09378 -3.45735
H 3.45015 -3.87490 -2.97694
H 0.92820 -3.23131 -1.37181
H 3.16291 -5.24451 -0.80352
H 1.55244 -5.63434 -1.42569
H 1.74343 -4.98944 0.21282
H 1.93570 2.03202 3.01920
H 2.02476 1.78470 1.26201
H 3.48412 1.70199 2.24561
H 1.11428 -0.22311 2.44875
H 3.79646 -0.22255 3.93167
H 2.61066 -1.52128 4.03854
H 2.20817 0.10162 4.62560
H -3.87927 -2.46313 2.57798
H -4.09620 -0.70534 2.35522
H -2.93777 -1.34232 3.54848
H -5.56349 -0.25689 -2.95519
H -5.47044 -2.75307 -1.28160
H -4.55494 -1.63165 -3.38716
H -5.10900 -2.08916 0.31981
H -3.84471 -0.01209 -3.28968
H -3.30747 -1.52991 -1.32966
H -3.08442 2.83533 3.76599
H -6.29754 -1.29511 -0.71380
H -3.15567 1.08201 3.92554
H -3.70058 3.56641 1.57368
H -5.68109 1.32950 -1.47971
H -1.19369 0.90092 2.32295
H -5.46127 3.39123 -0.14281
H -1.72279 1.96684 4.47651
H -1.52158 3.92846 2.00026
H -0.20423 3.11804 2.84303
H -0.39127 2.90926 1.09839
H -1.23773 2.75116 -1.21368
H -0.98210 3.45623 -2.82975
H -1.71317 1.84874 -2.65481
H 5.41542 5.13349 -0.77328
H 2.83587 0.96777 -2.00175
H 4.73845 2.16174 -1.04774
H 0.31556 4.36201 -1.17316
H 2.21214 5.50939 -0.16284
H 4.50530 5.48717 0.69604
H 5.48126 4.01490 0.58634
H -0.18568 0.83391 -4.04836
H 0.76853 2.31027 -4.33145
H 1.54572 0.84168 -3.70671
C -0.84638 -1.56505 -2.69970
H -1.66294 -3.42579 0.81718
H 0.10094 -1.53211 -3.24071
H -1.23034 -2.59034 -2.74770
H -1.56251 -0.91512 -3.20669

INT2[P-ZnMe]
B3PW91
SCF = -1911.80750783
SCF (C6H5Cl) = -1911.85391566
SCF (BJD3) = -1912.08370405
SCF (BS2) = -3464.54192725
H (0 K) = -1910.916409
H (298 K) = -1910.865166
G (298 K) = -1911.000665
Low freq. = 13.3801
Second freq. = 26.6150

101
INT2[P-ZnMe]
C 1.71369 4.09923 -1.45580
C 0.68855 3.19417 -1.73819
C 0.96440 1.83012 -1.84930
C 2.28287 1.39874 -1.66615
C 3.29611 2.31110 -1.40108
C 3.03599 3.68395 -1.29596
N -0.05087 0.84846 -2.22229
C 0.31494 0.25978 -3.54116
C 4.14543 4.66793 -1.04225
Zn -0.42354 -0.94769 -0.91683
N 0.96575 -1.45525 0.68411
C 2.38697 -1.19214 0.68236
C 2.92928 -0.20333 1.52965
C 4.30747 0.02895 1.45897
C 5.12364 -0.68359 0.59074
C 4.56767 -1.66057 -0.22684
C 3.19786 -1.93945 -0.20166
C 2.10668 0.60329 2.52462
C 2.55317 0.33933 3.97208
C 2.65228 -3.07422 -1.05783

C 3.02691 -4.43867 -0.45553
N -1.97106 -0.57082 0.58563
C -3.05604 0.37344 0.44408
C -4.20516 -0.01615 -0.28054
C -5.22878 0.92620 -0.43187
C -5.12920 2.20169 0.10555
C -3.98463 2.56449 0.80555
C -2.92382 1.67116 0.98515
C -4.39704 -1.40030 -0.89007
C -4.63443 -1.33201 -2.40626
C -1.69495 2.10041 1.77501
C -1.32783 3.57299 1.55448
C -2.03088 -1.46426 1.50907
C -3.10998 -1.57796 2.54347
C 0.46702 -2.21930 1.59090
C 1.24006 -2.88222 2.69016
C -1.00394 -2.57963 1.54742
C 3.11515 -2.99411 -2.51838
C 2.16896 2.10628 2.22064
C -5.55540 -2.15571 -0.21763
C -1.87816 1.83521 3.27848
C -1.39836 1.43495 -2.36643
H -1.22923 -3.23304 2.39236
H 2.31152 -2.70107 2.62117
H 1.05132 -3.96171 2.66691
H 0.87557 -2.52471 3.66040
H 5.21175 -2.23224 -0.88834
H 6.19173 -0.48897 0.56041
H 4.74794 0.78173 2.10645
H 2.88479 -2.02257 -2.96678
H 2.61842 -3.76678 -3.11349
H 4.19328 -3.15514 -2.61702
H 1.56008 -3.00372 -1.06194
H 4.11344 -4.57474 -0.44003
H 2.59594 -5.25217 -1.04824
H 2.66932 -4.54343 0.57340
H 1.56323 2.66255 2.94346
H 1.80833 2.33288 1.21423
H 3.19334 2.48422 2.29903
H 1.05907 0.29495 2.43669
H 3.57323 0.69795 4.14308
H 2.53527 -0.72437 4.22553
H 1.89991 0.86682 4.67492
H -3.62431 -2.53928 2.42672
H -3.84105 -0.77305 2.48331
H -2.65946 -1.59054 3.54242
H -5.56483 -0.80741 -2.64542
H -5.63311 -3.17134 -0.61898
H -4.71053 -2.34122 -2.82321
H -5.42995 -2.22723 0.86631
H -3.82156 -0.81561 -2.92501
H -3.48061 -1.97905 -0.73548
H -2.73234 2.39968 3.66713
H -6.51161 -1.65526 -0.40172
H -2.05549 0.77869 3.49534
H -3.91318 3.56377 1.22194
H -6.12452 0.64710 -0.97926
H -0.84945 1.49253 1.42740
H -5.94021 2.91283 -0.02005
H -0.98938 2.14812 3.83628
H -2.05618 4.24775 2.01527
H -0.35900 3.78980 2.01164
H -1.25875 3.82336 0.49258
H -1.71762 1.90369 -1.43572
H -1.42851 2.17414 -3.17750
H -2.10111 0.63547 -2.60782
H 4.72328 4.84940 -1.95612
H 2.52898 0.34322 -1.72813
H 4.30920 1.94264 -1.26673
H -0.31309 3.58043 -1.88297
H 1.47023 5.15567 -1.37639
H 3.75811 5.63176 -0.70222
H 4.84550 4.29776 -0.28699
H -0.44931 -0.45875 -3.83933
H 0.39037 1.05262 -4.29532
H 1.27118 -0.25741 -3.47661
C -0.88621 -2.47176 -2.11070
H -1.15737 -3.17854 0.63706
H -0.06927 -2.69897 -2.80283
H -1.10221 -3.39723 -1.56440
H -1.77325 -2.25019 -2.71337

TS3[P-ZnMe]
B3PW91
SCF = -1911.77230406
SCF (C6H5Cl) = -1911.81715326
SCF (BJD3) = -1912.05136240
SCF (BS2) = -3464.50854423
H (0 K) = -1910.885283
H (298 K) = -1910.835085
G (298 K) = -1910.967719
Low freq. = -1274.4887
Second freq. = 13.7012

101
TS3[P-ZnMe]
C -0.06139 4.28158 -1.80269
C -0.55016 3.02100 -2.15202
C 0.21652 1.88444 -1.89981
C 1.46365 2.02780 -1.28451

C	1.93992	3.28960	-0.95055
C	1.19079	4.44598	-1.20864
N	-0.19833	0.54023	-2.32904
C	0.70519	0.09924	-3.43106
C	1.72845	5.80839	-0.86701
Zn	-0.18993	-0.87520	-0.75924
N	1.34717	-1.31586	0.50744
C	2.71474	-0.86554	0.49522
C	3.18810	-0.01002	1.51604
C	4.51370	0.43481	1.44138
C	5.35113	0.05887	0.40172
C	4.87052	-0.78448	-0.59319
C	3.55792	-1.26407	-0.57271
C	2.34319	0.45137	2.69581
C	2.92143	-0.03771	4.03385
C	3.09993	-2.24148	-1.64722
C	3.30534	-3.69736	-1.19624
N	-1.69740	-0.80958	0.60855
C	-2.87834	0.01506	0.57717
C	-4.05795	-0.45803	-0.04851
C	-5.14114	0.42312	-0.15363
C	-5.08062	1.71591	0.34628
C	-3.92501	2.15104	0.98440
C	-2.80856	1.32093	1.11524
C	-4.23670	-1.87528	-0.58174
C	-4.52950	-1.90388	-2.08934
C	-1.59389	1.79472	1.89826
C	-1.34236	3.30150	1.78747
C	-1.57597	-1.82506	1.42219
C	-2.60958	-2.20707	2.44199
C	0.96151	-2.28010	1.30619
C	1.89891	-3.05888	2.18451
C	-0.42851	-2.72657	1.29357
C	3.77851	-2.02822	-3.00626
C	2.20047	1.97998	2.71635
C	-5.35728	-2.61758	0.16692
C	-1.73088	1.39251	3.37690
C	-1.58747	0.50003	-2.84317
H	-0.56395	-3.63006	1.88582
H	2.94693	-2.83036	1.99192
H	1.72837	-4.13087	2.04074
H	1.67735	-2.84653	3.23590
H	5.53320	-1.08366	-1.39839
H	6.37642	0.41519	0.36735
H	4.89471	1.08723	2.22192
H	3.76663	-0.98026	-3.32233
H	3.27329	-2.62326	-3.77349
H	4.82330	-2.35454	-2.99359
H	2.02104	-2.09937	-1.78845
H	4.36521	-3.89128	-1.00096
H	2.97510	-4.39266	-1.97539
H	2.75129	-3.92782	-0.28324
H	1.56654	2.29204	3.55262
H	1.75816	2.35506	1.78965
H	3.17173	2.46856	2.84697
H	1.33825	0.02999	2.58796
H	3.89358	0.42320	4.23685
H	3.06508	-1.12130	4.05241
H	2.25357	0.23153	4.85865
H	-3.00460	-3.20329	2.21408
H	-3.43669	-1.49860	2.48278
H	-2.14158	-2.27331	3.42938
H	-5.46979	-1.39449	-2.32295
H	-5.40816	-3.66090	-0.16154
H	-4.62160	-2.93709	-2.43965
H	-5.21029	-2.60874	1.24984
H	-3.74112	-1.42104	-2.67179
H	-3.30419	-2.42447	-0.41939
H	-2.61458	1.86250	3.82155
H	-6.33307	-2.16375	-0.03356
H	-1.83390	0.31106	3.50186
H	-3.89192	3.15500	1.39393
H	-6.05453	0.07967	-0.63116
H	-0.71430	1.27876	1.49261
H	-5.93586	2.37877	0.25288
H	-0.85569	1.71656	3.94937
H	-2.11422	3.88300	2.30228
H	-0.38963	3.55500	2.26045
H	-1.30076	3.63129	0.74606
H	-2.28728	0.82976	-2.07355
H	-1.69579	1.12532	-3.73679
H	-1.82161	-0.52900	-3.12069
H	2.55500	6.08040	-1.53335
H	2.07776	1.15677	-1.06966
H	2.91632	3.37338	-0.48096
H	-1.52649	2.95724	-2.61674
H	-0.67470	5.15447	-2.01013
H	0.95983	6.57914	-0.96138
H	2.11570	5.83945	0.15627
H	0.42522	-0.90481	-3.75823
H	0.61933	0.78997	-4.27767
H	1.73755	0.08814	-3.08701
C	-0.57422	-2.92944	-1.53575
H	-0.50944	-2.86836	-0.08680
H	-0.51478	-2.39062	-2.49595
H	0.19792	-3.70144	-1.60356
H	-1.55845	-3.40662	-1.54952

S8. References

1 M. Stender, R. J. Wright, B. E. Eichler, J. Prust, M. M. Olmstead, H. W. Roesky and P. P. Power, *J. Chem. Soc. Dalton Trans.*, 2001, 3465–3469.
2 M. Garçon, N. W. Mun, A. J. P. White and M. R. Crimmin, *Angew. Chem. Int. Ed.*, 2021, **60**, 6145–6153.
3 M. E. Grundy, L. Sotorrios, M. K. Bisai, K. Yuan, S. A. Macgregor and M. J. Ingleson, *ACS Catal.*, 2023, **13**, 2286–2294.
4 M. Kumar Bisai, J. Łosiewicz, L. Sotorrios, G. S. Nichol, A. P. Dominey, M. J. Cowley, S. P. Thomas, S. A. Macgregor and M. J. Ingleson, *Angew. Chem. Int. Ed.*, 2024, **63**, e202404848.
5 D. Uraguchi, N. Kinoshita, T. Kizu and T. Ooi, *J. Am. Chem. Soc.*, 2015, **137**, 13768–13771.
6 C. A. Reed, *Acc. Chem. Res.*, 2010, **43**, 121–128.
7 A. Vasseur, J. Muzart and J. Le Bras, *Chem. Eur. J.*, 2011, **17**, 12556–12560.
8 J. Prust, A. Stasch, W. Zheng, H. W. Roesky, E. Alexopoulos, I. Usón, D. Böhler and T. Schuchardt, *Organometallics*, 2001, **20**, 3825–3828.
9 M. K. Bisai, J. Łosiewicz, G. S. Nichol, A. P. Dominey, S. P. Thomas, S. A. Macgregor and M. J. Ingleson, *Chem. Sci.*, 2025, **16**, 9255–9263.
10 J. Yu, G. Kehr, C. G. Daniliuc, C. Bannwarth, S. Grimme and G. Erker, *Org. Biomol. Chem.*, 2015, **13**, 5783–5792.
11 A. Prokofjevs, J. W. Kampf and E. Vedejs, *Angew. Chem. Int. Ed.*, 2011, **50**, 2098–2101.
12 M. J. Frisch, G. W. Trucks, H. B. Schlegel, G. E. Scuseria, M. A. Robb, J. R. Cheeseman, G. Scalmani, V. Barone, G. A. Petersson, H. Nakatsuji, X. Li, M. Caricato, A. V. Marenich, J. Bloino, B. G. Janesko, R. Gomperts, B. Mennucci, H. P. Hratchian, J. V. Ortiz, A. F. Izmaylov, J. L. Sonnenberg, D. Williams-Young, F. Ding, F. Lipparini, F. Egidi, J. Goings, B. Peng, A. Petrone, T. Henderson, D. Ranasinghe, V. G. Zakrzewski, J. Gao, N. Rega, G. Zheng, W. Liang, M. Hada, M. Ehara, K. Toyota, R. Fukuda, J. Hasegawa, M. Ishida, T. Nakajima, Y. Honda, O. Kitao, H. Nakai, T. Vreven, K. Throssell, J. J. A. Montgomery, J. E. Peralta, F. Ogliaro, M. J. Bearpark, J. J. Heyd, E. N. Brothers, K. N. Kudin, V. N. Staroverov, T. A. Keith, R. Kobayashi, J. Normand, K. Raghavachari, A. P. Rendell, J. C. Burant, S. S. Iyengar, J. Tomasi, M. Cossi, J. M. Millam, M. Klene, C. Adamo, R. Cammi, J. W. Ochterski, R. L. Martin, K. Morokuma, O. Farkas, J. B. Foresman and D. J. Fox, 2016.
13 A. D. Becke, *J. Chem. Phys.*, 1993, **98**, 5648–5652.
14 D. Andrae, U. Häußermann, M. Dolg, H. Stoll and H. Preuß, *Theor. Chim. Acta*, 1990, **77**, 123–141.
15 W. J. Hehre, R. Ditchfield and J. A. Pople, *J. Chem. Phys.*, 1972, **56**, 2257–2261.

16 P. C. Hariharan and J. A. Pople, *Theor. Chim. Acta*, 1973, **28**, 213–222.

17 A. Höllwarth, M. Böhme, S. Dapprich, A. W. Ehlers, A. Gobbi, V. Jonas, K. F. Köhler, R. Stegmann, A. Veldkamp and G. Frenking, *Chem. Phys. Lett.*, 1993, **208**, 237–240.

18 F. Weigend, *Phys. Chem. Chem. Phys.*, 2006, **8**, 1057–1065.

19 F. Weigend and R. Ahlrichs, *Phys. Chem. Chem. Phys.*, 2005, **7**, 3297–3305.

20 S. Grimme, J. Antony, S. Ehrlich and H. Krieg, *J. Chem. Phys.*, 2010, **132**, 154104.

21 J. Tomasi, B. Mennucci and R. Cammi, *Chem. Rev.*, 2005, **105**, 2999–3094.

22 A. D. Becke, *Phys. Rev. A*, 1988, **38**, 3098–3100.

23 J. P. Perdew, *Phys. Rev. B*, 1986, **33**, 8822–8824.

24 C. Lee, W. Yang and R. G. Parr, *Phys. Rev. B*, 1988, **37**, 785–789.

25 C. Adamo and V. Barone, *J. Chem. Phys.*, 1999, **110**, 6158–6170.

26 S. Grimme, S. Ehrlich and L. Goerigk, *J. Comput. Chem.*, 2011, **32**, 1456–1465.

27 S. Grimme, *J. Comput. Chem.*, 2006, **27**, 1787–1799.

28 Y. Zhao and D. G. Truhlar, *Theor. Chem. Acc.*, 2008, **120**, 215–241.

29 J. D. Chai and M. Head-Gordon, *Phys. Chem. Chem. Phys.*, 2008, **10**, 6615–6620.

30 J. Tao, J. P. Perdew, V. N. Staroverov and G. E. Scuseria, *Phys. Rev. Lett.*, 2003, **91**, 146401.
